# Supplementary material for: Healthcare providers’ perceptions on post abortion intrauterine contraception: A qualitative study in central Uganda
Source: PLoS One. 2024 Dec 5;19(12):e0301748. doi: 10.1371/journal.pone.0301748 (PMC11620438; doi:10.1371/journal.pone.0301748)
Supplement: S1 Annex — (DOCX) [file pone.0301748.s001.docx]

**Annex 1: Background characteristics of study sites**

| **Hospital** | **Location** | **Level of Care** | **No. of beds and population served** | **No. healthcare providers** | **Duration of work** | **cumulative 1^st^ trimester Abortion load in the 3 months (July- Sept 2022** | **Setting** | **Service delivered** |
| --- | --- | --- | --- | --- | --- | --- | --- | --- |
|  | Kampala | National Referral hospital | Serves a population of 4.5 million, with a bed capacity of 900 | 500 | 24 hours a day, 7 days a week. | 484 | Urban | Teaching hospital.  Offers free emergency gynecological services |
| 2. | Kampala | Regional referral hospital | Serves a population of 3 million, Bed capacity of 100. | 356 | 24 hours a day, 7 days a week. | 300 | Peri-urban | Teaching hospital  Offers free gynecology and family planning services |
| 3. | Wakiso | Health Centre IV | Serves a population of 200,000.  Bed capacity of 30. | 28 | 8 hours, 5 days a week | 30 | Peri-urban | Emergency gynecological and family planning services |
| 4. | Mpigi | Hospital | 100 beds. | 20 | 24 hours a day, 7 days a week | 48 | Rural | Offer outpatient services as well Post abortion care (PAC) |
| 5. | Masaka | Hospital | 330 beds | 40 | 24 hours a day, 7 days a week. | 155 | Peri-urban | Offers maternal health services including PAC services |
| 6. | Gombe | Hospital | 100 beds. | 260 | 24 hours a day, 7 days a week. | 76 | Rural | Offers both in-and outpatient services including PAC services. |
| 7. | Nakaseke | Hospital | 100 beds. | 417 | 24 hours a day, 7 days a week. | 50 | Rural | Offers all maternal health services including PAC services. |
| 8. | Luwero | Hospital | 80 beds. | 45 | 24 hours a day, 7 days a week. | 76 | Rural | Offers comprehensive maternal health services |
| 9. | Mityana | Hospital | 100 | 116 | 24 hours a day, 7 days a week. | 100 | Peri-urban | Offers comprehensive maternal health services  including PAC |
| 10. | Mukono | Health Centre IV | 12 | 80 | 24 hours a day, 5 days a week. | 46 | Peri-urban | Offers both in and outpatient services including family planning |
| 11 | Kasanda | Health centre IV | 15 |  | 24 hours a day, 5 days a week | 38 | Rural | Offers both in and outpatient services including family planning |
| 12 | Wakiso | Health centre IV | 40 | 15 | 24 hours a day, 5 days a week | 50 | Peri-urban | Offers  maternal services including PAC |
| 13 | Buikwe | Hospital | 106 | 100 | 24 hours a day, 7 days a week | 100 | Peri-urban | Offers  maternal services including PAC |
| 14 | Kayunga | Regional Referral hospital | 200 | 179 | 24 hours a day, 7 days a week | 212 | Peri-urban | Offers specialist  maternal services including PAC |
| 15 | Wakiso | General hospital | 200 | 155 | 24 hours a day, 7 days a week | 91 | Peri-urban | Offers specialist  maternal services including PAC |
| 16 | Kasanda | Health centre III | 20 | 30 | 24 hours a day, 5 days a week | 60 | Rural | Offers  maternal services including PAC, family planning services |
