# Supplementary material for: Healthcare providers’ perceptions on post abortion intrauterine contraception: A qualitative study in central Uganda
Source: PLoS One. 2024 Dec 5;19(12):e0301748. doi: 10.1371/journal.pone.0301748 (PMC11620438; doi:10.1371/journal.pone.0301748)
Supplement: S1 File — (PDF) [file pone.0301748.s002.pdf]

MAKERERE UNIVERSITY

P.O. Box 7072, Kampala, Uganda
Fax: 256 41 4532591

Phone: +256-414-534361
e-mail: obsgyn@chs.mak.ac.ug

EFFECTIVENESS, ACCEPTABILITY AND UPTAKE OF EARLY VERSUS STANDARD INTRAUTERINE CONTRACEPTION FOLLOWING PROVISION OF FIRST TRIMESTER MEDICAL POST ABORTION CARE IN CENTRAL UGANDA:

PRINCIPAL INVESTIGATOR:

DR. HERBERT KAYIGA, MBChB, MPH, MMED OBS/GYN
PhD Student - Makerere University College of Health Sciences (MakCHS)

SUPERVISORS:

ASSOC. PROF JOSAPHAT BYAMUGISHA, MD, PhD
Department of Obs/Gyn, Makerere University College of Health Sciences

DR. OTHMAN KAKAIRE, MD, PhD
Senior Lecturer, Department of Obs/Gyn, Makerere University College of Health Sciences

PROF KRISTINA GEMZELL-DANIELSSON, MD, PhD
Karolinska Institutet and Karolinska University Hospital

ASSOC. PROF NAZARIUS.M. TUMWESIGYE, MSc. PhD
Department of Epidemiology and Biostatistics, Makerere University College of Health Sciences

A PhD PROPOSAL SUBMITTED TO THE SOMREC, MAKERERE UNIVERSITY, KAMPALA.

MARCH 2022

DOCTORAL COMMITTEE

ASSOC. PROF ANNETTEE OLIVIA NAKIMULI, MD, PhD
Dean, Makerere University College of Health Sciences

DR. JOSEPH RUJUMBA, MA, PhD
Lecturer, Department of Paediatrics and Child Health, Makerere University College of Health Sciences

ASSOC. PROF JOSAPHAT BYAMUGISHA, MD, PhD
Department of Obs/Gyn, Makerere University College of Health Sciences

DR. OTHMAN KAKAIRE, MD, PhD
Senior Lecturer, Department of Obs/Gyn, Makerere University College of Health Sciences

PROF KRISTINA GEMZELL-DANIELSSON, MD, PhD
Karolinska Institutet and Karolinska University Hospital

ASSOC. PROF NAZARIUS.M. TUMWESIGYE, MSc. PhD
Department of Epidemiology and Biostatistics, Makerere University College of Health Sciences

TABLE OF CONTENTS

DECLARATION **6**

ACKNOWLEGEMENT **5**

LIST OF ABBREVIATIONS **6**

OPERATIONAL DEFINITIONS **7**

ABSTRACT **8**

CHAPTER ONE: **10**

1.0 BACKGROUND **10**

1.2 PROBLEM STATEMENT: **13**

1.3 SIGNIFICANCE: **14**

1.4 JUSTIFICATION: **15**

1.5 HYPOTHESIS **15**

1.6 RESEARCH QUESTIONS: 16

1.7 General objective: 16

1.7.1 Specific objectives: 16

1.8 CONCEPTUAL FRAME WORK **18**

CHAPTER TWO: **19**

2.1 LITERATURE REVIEW **19**

CHAPTER THREE: METHODS: **27**

3.1 Study sites 27

3.2 SUB-STUDY I: UPTAKE AND FACTORS ASSOCIATED WITH UTILIZATION OF POST ABORTION INTRAUTERINE CONTRACEPTION AFTER FIRST TRIMESTER MEDICAL MANAGEMENT OF INCOMPLETE ABORTION IN CENTRAL UGANDA 34

3.3 SUB-STUDY 2: EFFECTIVENESS OF EARLY VERSUS STANDARD INSERTION OF POST ABORTION INTRAUTERINE CONTRACEPTION AFTER FIRST TRIMESTER MEDICAL MANAGEMENT OF INCOMPLETE ABORTION IN CENTRAL UGANDA: A NON–INFERIORITY RANDOMIZED CLINICAL TRIAL 40

3.4 SUB-STUDY 3: HEALTHCARE PROVIDERS' PERCEPTIONS TOWARDS POST ABORTION INTRAUTERINE CONTRACEPTION AFTER FIRST TRIMESTER MEDICAL MANAGEMENT OF ABORTIONS IN UGANDA: A QUALITATIVE STUDY 54

3.5 SUB-STUDY 4: WOMEN AND THEIR SPOUSES' PERCEPTIONS TOWARDS POST ABORTION INTRAUTERINE CONTRACEPTION AFTER FIRST TRIMESTER MEDICAL MANAGEMENT OF INCOMPLETE ABORTIONS IN UGANDA: A QUALITATIVE STUDY 60

3.7 Study limitations ... 66
3.8 Dissemination of study findings: ... 67
3.9 International and National research collaboration: ... 67
4.0 WORK PLAN: ... 69
5.0 REFERENCES: ... 70
APPENDIX I: BUDGET ... 77
APPENDIX II: BUDGET JUSTIFICATION: ... 79
APPENDIX III: INFORMED CONSENT FOR RCT ... 81
APPENDIX IV: INFORMED ASSENT FOR RCT ... 90
APPENDIX V: INFORMED CONSENT FOR UPTAKE OF POST ABORTION IUC ... 100
APPENDIX VI: INFORMED ASSENT FOR UPTAKE OF POST ABORTION IUC ... 108
APPENDIX VII: HEALTH WORKERS' CONSENT FOR INTERVIEW ... 116
APPENDIX VIII: IN DEPTH INTERVIEW CONSENT FORM FOR WOMEN ... 122
APPENDIX IX: IN DEPTH INTERVIEW ASSENT FORM FOR EMANCIPATED MINORS ... 129
APPENDIX X: IN DEPTH INTERVIEW CONSENT FORM FOR SPOUSES ... 135
APPENDIX XI: IN DEPTH INTERVIEW ASSENT FORM FOR EMANCIPATED MINORS ... 142
APPENDIX XII: LUGANDA CONSENT RCT ... 149
APPENDIX XIII: LUGANDA CONSENT FOR UPTAKE ... 159
APPENDIX XIV: LUGANDA CONSENT FOR HEALTHCARE PROVIDERS ... 168
EKIWANDIIKO KY'OKUKKIRIZA (EKIWA OLUKUSA) EKY'ABASAWO ... 168
APPENDIX XV: LUGANDA CONSENT FOR QUALITATIVE STUDY AMONG COUPLES ... 175
APPENDIX XVI: LUGANDA CONSENT FOR SPOUSES ... 184
APPENDIX XVII: DATA COLLECTION TOOL ... 192
APPENDIX XVIII: QUALITATIVE HEALTH WORKERS' INTERVIEW GUIDE: ... 202
Interview Guide questions: ... 202
APPENDIX XIX: QUALITATIVE WOMEN AND THEIR SPOUSES' INTERVIEW GUIDE: ... 204
Interview Guide questions: ... 205
APPENDIX XX: ELIGIBILITY SCREENING ... 206
APPENDIX XXI: CASE REPORT FORM (CRF) ... 207
APPENDIX XXII: POST-TRIAL ACCESS: TRIAL CLOSE AND CONTINUATION ACCESS MANUAL ... 299
APPENDIX XXIII: COVID 19 RISK MANAGEMENT PLAN ... 305

ACKNOWLEGEMENT

I extend my gratitude to the Department of Obstetrics and Gynaecology of Makerere University College of Health Sciences, for all the help offered to me in critiquing my study right from the concept level. I'm humbled by all the support from my supervisors; Prof Josaphat Byamugisha, Dr. Othman Kakaire, Prof Nazarius M. Tumwesigye, and Prof Kristina Gemzell-Danielsson. Without your guidance, I wouldn't have made it this far.

I'm so thankful to my Doctoral committee; Assoc. Prof Annette Nakimuli, and Dr. Joseph Rujjumba for offering their full support in this PhD journey.

I would like to appreciate my wife Dr. Pauline Amuge Kayiga, and daughters Callista, Elizabeth and Kayla for being patient with me in this course. Finally, I appreciate the Almighty God for giving me the grace to come this far with the PhD training.

LIST OF ABBREVIATIONS:

**DMPA**	Injectable Depo-Medroxyprogesterone
**IUC**	Intrauterine Contraception
**IUD**	Intrauterine Device
**IUS**	Intrauterine system
**FIGO**	International Federation of Obstetricians and Gynecologists
**LARCs**	Long-Acting Reversible Contraceptives
**LMIC**	Low- and Middle-income countries
**PAC**	Post Abortion Care
**mPAC**	Medical Post Abortion Care
**SOMREC**	School of Medicine Ethics Review Committee
**UNCST**	Uganda National Council of Science and Technology
**WHO**	World Health Organization

OPERATIONAL DEFINITIONS:

Acceptability: A woman's perception, experience and satisfaction towards post abortion intrauterine contraception (whether she would recommend the post abortion intrauterine contraception to a friend or if it would be her future choice when availed to her in the same circumstances).

Discontinuation: The stoppage to use Intra Uterine contraception (IUC) on a clinical basis or participant's choice by six months post insertion

Early IUC: Insertion of an IUC within one week after medical Post Abortion Care

Effectiveness: The proportion of women who will conceive while using early or standard IUC within one year of insertion.

Expulsion: The passage of an IUC spontaneously out of the uterus or when any portion of the IUC is visible in the cervical canal or the vagina.

First trimester abortion: Pregnancy loss within 12 weeks of amenorrhea

Intrauterine Contraception: Insertion of a copper or Levonorgestrel releasing device in the uterus after medical management of a first trimester incomplete abortion.

Medical PAC: The use of Misoprostol to treat a first trimester incomplete abortion

Post abortion care (PAC): The comprehensive management of women presenting with an incomplete abortion.

Pain: The visual perception of discomfort by the patient from the insertion of the speculum to the point on IUC insertion on a scale of ten

Pelvic infection: The presence of purulent par vaginal discharge, with adnexal tenderness, cervical motion tenderness, fever of $38^{o}C$ or more during or after a miscarriage or IUC insertion.

Standard IUC: Insertion of an IUC 2-4 weeks after medical PAC

Uptake: The utilization of IUC at or after medical post abortion care

ABSTRACT

Background: Unintended pregnancies continue to cause a public health threat in Low- and Middle-Income countries yet with restrictive abortion laws in these settings. Over 40% of these unintended pregnancies end up as unsafe abortions leading to significant maternal morbidity and mortality. With ovulation occurring between 5-10 days after first trimester abortion, 47% of the women conceive shortly afterwards. This could be because nearly 50% of the women never return for post abortion follow up visits.

This study seeks to investigate uptake, acceptability, continued use and effectiveness of early insertion of Intrauterine contraception (IUC) (within one week after medical Post abortion care (mPAC)) compared to Standard IUC insertion (between 2-4 weeks post PAC after first trimester incomplete abortion), in Uganda with the ultimate aim of increasing the uptake of IUC post 1st trimester medical management of incomplete abortion.

Specific Objectives:

1. To determine the proportion of women who take up IUC after mPAC for 1st trimester incomplete abortion.
2. To compare the expulsion rates at six months between early versus standard IUC insertions post mPAC treatment for first trimester incomplete abortion.
3. To compare the IUC continuation rates at six months between early versus standard IUC insertion post mPAC treatment for first trimester incomplete abortion.
4. To explore the women and their spouses' perception on Long-Acting Reversible Contraceptives (LARC) and IUC following mPAC treatment.
5. To explore the Healthcare providers' perception on LARC and IUC following mPAC treatment.

Methods: This study will be conducted this study in 5 health facilities within central Uganda. The first sub-study will evaluate the uptake of intrauterine contraception and the predictors of the uptake in a cross-sectional study design of 650 participants. Health workers’ perception towards post abortion intrauterine contraception will be explored to understand the barriers and facilitators towards their recommendation of IUC. About 20-30 in depth interviews among healthcare providers, will be conducted until data saturation is obtained.

A Non-inferiority RCT of 1,000 participants will be used to determine the expulsion rates, and continued use at six months of early versus standard intrauterine contraception after first trimester medical management of incomplete abortion. To understand the perception of the women and their spouses towards post abortion intrauterine contraception, 20-30 in depth interviews will be conducted. Baseline participant characteristics will be analysed using descriptive statistics. Categorical variables such as uptake of a contraceptive method and adherence to the method will be analysed using a Chi-square with the level of significance at <0.05. For continuous variables, statistical tests such as Fisher's exact test, t-test, ANOVA and Wilcoxon-Mann-Whitney test will be used as appropriate. Binary logistic regression model will be used for factors associated with the dependant variable uptake of intrauterine contraception with p-value set <0.05 as level of significance. Intention to treat and per protocol analysis will be used for the RCTs. Risk ratios and confidence intervals will be used to describe associations.

Study utility: The rationale for this study is that a greater proportion of women undergoing medical post abortion care for incomplete abortion will receive their intended post abortion IUC within the first days after the PAC treatment compared with routine insertion at a scheduled follow up visit 2-4 weeks later. Though there could be higher expulsion rates in the early versus the standard IUC insertion, continued use at 6 months following abortion will be higher in the early insertion group as compared to the standard insertion group. This will help women avoid unplanned and unwanted pregnancy following medical treatment of incomplete abortion.

CHAPTER ONE:

1.0 BACKGROUND:

Unintended pregnancies are a global public health threat especially in Low-and Middle-income countries (LMIC) (1). More than a third of the 182 million pregnancies in LMIC countries between 2009 and 2019, were unintended (1). This has been attributed mainly to the high unmet need for contraception especially in Sub-Saharan Africa (2). This unmet need is driven by knowledge gaps in contraception, inaccessibility to modern contraceptive methods especially in rural areas, minimal resource allocation for family planning services and limited skills among healthcare providers (3). As a result, over 40% of such unintended pregnancies in LMICs end up as induced abortions, 97% of which are unsafe (4, 5). In face of restrictive abortion laws, poverty, gender inequality in LMIC countries(6), a significant proportion of these induced abortions end up as unsafe leading to 11% of the maternal mortality that would have otherwise been preventable (6, 7, 8). The World Health Organization (WHO) estimated nearly half of the 56 million abortions that occurred globally between 2010-2014, were unsafe (7). Unsafe abortion contributing 460 deaths per 100,000 live births on the African Continent as compared to 30 deaths per 100,000 live births in high-resource countries (9). Women who survive deaths from unsafe abortions especially the adolescents end up living with physical and psychological complications such as secondary infertility, chronic pelvic pain, pelvic inflammatory disease or sepsis(10, 11).

Uganda has one of the fastest growing populations worldwide from 5 million in 1948 to 44.27 million in 2018 and highest maternal mortality rates (of 336 per 100,000 live births) globally (State of Uganda Population report 2018). According to the Uganda Demographic survey (12) 2016, 78% of the population are 30 years or younger. Uganda also has one of the highest fertility rates in the region at 5.4 children per woman yet with a high dependency ratio of 103 (13). By 17 years, adolescents are sexually active with 25% of these adolescents conceiving in the process(13). With a high unmet need for contraception at 28% among married women and 32% among unmarried sexually active women, 56% of the pregnancies in Uganda are unintended translating in 1,036,000 unintended pregnancies which could be averted with the use of modern contraceptive methods(14, 15). These unintended pregnancies are even higher among teenagers; with seven out of ten young women not intending to conceive but not using any effective contraceptive method(15). Uganda's contraceptive uptake of 30% is also lower than that of its neighbors; Kenya (46%), Rwanda (52%)

and Tanzania (34%) calling for measures to scale up the low uptake(16). Singh et al (17) reported of the 314,304 women who undergo unsafe abortions each year in Uganda, about 128,682 experience complications with only 85,000 obtaining care. About ten percent of the maternal mortality in Uganda follows complications of unsafe abortions in face of restrictive abortion laws (13, 17).

WHO recommends early post abortion contraception since fertility returns from as early as five to ten days after first trimester abortions(18, 19, 20), and yet over 50% of these women resume sexual intercourse within two weeks(21). This reduces on missed opportunities of using an effective family planning method(22), since women undergoing abortion are at increased risk for subsequent unintended pregnancies and repeat abortions(23). Prior studies have shown that about 42% of the women scheduled for post abortion contraception never return for follow up visits for reasons such as; long distances to travel and other social demands predisposing them to unintended pregnancies (24, 25, 26, 27, 28, 29, 30).

Being a signatory to the Sustainable Development Goals (SDGs), one of the interventions to improve maternal health in Uganda is provision of Post Abortion Care (13, 31). The Ministry of Health (MoH) estimates that for every one dollar spent on family planning, six dollars are saved on maternal health expenditure. With the uptake of contraception, 28% of the maternal deaths can be averted (13). Post Abortion care model (32, 33, 34, 35, 36, 37, 38) has been adopted to reduce harmful consequences of unsafe abortion. It has five elements: 1) treatment for complications due to spontaneous or induced abortion; 2) post abortion contraceptive counselling and provision; 3) counselling to respond to women's emotional and physical needs; 4) linkages to reproductive or other health services; and 5) community and service provider partnership.

The use of Long-Acting reversible contraceptives (LARC) such as implants, intrauterine contraceptives has been found to be safe and effective immediately after an induced or spontaneous abortion (20, 39, 40, 41, 42, 43). Short acting contraceptive methods such as oral contraceptives and injectable Depo-Medroxyprogesterone (DMPA) and condoms, are user-dependent and have high discontinuation rates. It's to this end that the International Federation of Obstetricians and

Gynecologists (FIGO) recommends use of LARCs as they are cost effective in reducing maternal mortality and morbidity yet with higher continuation rates(25, 44, 45, 46, 47, 48, 49).

Whereas WHO recommends use of early intrauterine contraception after manual vacuum aspiration for incomplete abortion, placement of intrauterine contraception after medical management with Misoprostol has been between 2-4 weeks post abortion (20, 25, 50), since there's barely any published evidence on its safety on early insertion(51, 52, 53). This follows the notion that complete expulsion of the products of conception is expected by 2-4 weeks after medical management of incomplete abortion. This complete expulsion is usually confirmed using clinical evaluation, with ultrasonography reserved for cases where clinical findings are inconclusive(54). With a contraceptive uptake of intrauterine devices of only 4.1% in Uganda, this study aims at scaling up the utilization of intrauterine contraception immediately after first trimester medical management of incomplete abortion. Uganda lacks a policy on the ideal time to have intrauterine contraception after medical management of incomplete abortions The practice in Uganda has been placement of intrauterine contraception immediately after surgical evaluation after incomplete abortions or 2-4 weeks after medical management of incomplete abortion(55).

Early post abortion IUC fronts the advantages of having the women motivated to take on the method, the assurance that they are not pregnant, their cervixes are open hence lesser pain at insertion, yet with higher continuation rates and patient satisfaction than with the standard insertion between 2-4 weeks after medical PAC. There's also no need for back contraception with early IUC as implemented for standard IUC (51, 56) .

It's against this background that this study seeks to determine uptake, acceptability, continued use and effectiveness of early insertion of IUC (within one week after medical PAC) compared to Standard IUC insertion (at 2-4 weeks post PAC after first trimester incomplete abortion), in Central Uganda with the ultimate aim of increasing the uptake of PAC services.

1.2 PROBLEM STATEMENT:

In face of an overwhelmed health system in Uganda, even with PAC services being freely available in 90% of the health facilities, a significant proportion of women who undergo medical or surgical evacuations don’t return for post abortion follow up visits. This then turns out to be a missed opportunity to take up an effective family planning method despite evidence of safety and effectiveness of intrauterine contraception a week after medical management of incomplete abortion (12, 24, 29, 30, 48, 50). Fertility for most women returns from as early as five days to ten days after first trimester abortions [19-21]. With more than 50% of the women resuming sexual intercourse within two weeks after first trimester pregnancy losses [22], the risk of early repeat pregnancies is high. Early repeat pregnancies are associated with adverse maternal and fetal outcomes such as repeat miscarriages, preterm labour, preeclampsia and fetal demise (57).

LARC methods are underutilized in sub-Saharan Africa (SSA) and Uganda in particular(58) [8]. Uganda’s pattern of low LARC use is manifested by the low IUC use of 0.5 % [8]. This can be compared to a current 30% user rate in Sweden [19].

Whereas the WHO recommends use of intrauterine contraception after immediately manual vacuum aspiration following incomplete abortion, placement of intrauterine contraception after medical management with Misoprostol has been between 2-4 weeks post abortion (20, 25, 50), as there’s barely any published evidence on the safety of early post abortion contraception after medical management for incomplete abortion(51, 52, 53). Prior studies have reported higher expulsion rates with early post abortion contraception after medical Post abortion care though with high continuation rates (51, 59, 60). Other studies have reported no difference in the expulsion rates, perforation, or discontinuation rates between early versus standard post abortion intrauterine contraceptive insertion (24, 61, 62, 63).

According to Sääv et al., [18], the early insertion of IUC within nine days of medical abortion was as effective as IUC insertion at 3-4 weeks after medical abortion treatment with similar expulsion rates. This study is yet to determine whether similar outcomes can be observed following post abortion care with Misoprostol for incomplete abortion. The low current use and low ever use of LARC in SSA is a manifestation that women in the region lack access to and/or knowledge about these methods [3]. Although LARC methods are safe, acceptable, with high satisfaction and continuation rates, yet with very low failure rates (58) there are several obstacles to their

utilization, and especially to IUC. The key barriers include; lack of widespread training of providers, coupled with lack of enduring provider competence and confidence, lack of consistent supply of methods, equipment, materials and space, lack of knowledge/interest on the part of potential users, provider bias and circumstances that favor provision of short-acting methods (e.g., time constraints and long client queues) even for women with long-term needs(58, 64, 65, 66). The rationale for this study is that a greater proportion of women undergoing medical PAC will receive their intended post abortion IUC if it is inserted within the first days after the PAC treatment compared with routine insertion at a scheduled follow up visit 2-4 weeks later.

1.3 SIGNIFICANCE:

Provision of accessible, safe and effective PAC and adequate contraceptive counselling and provision are critical in promoting maternal survival(67, 68). Post abortion contraceptive counselling and uptake is important to prevent repeat pregnancy, especially in sub Saharan Africa where access to health care is limited (32). Early IUC insertions post first trimester medical abortion management avails the opportunity of utilizing the limited human resource in low resource setting (69).

Prior studies have shown higher continuation rates and patient satisfaction with early IUC than with the standard insertion between 2-4 weeks after medical PAC(30). There’s also no need for back up contraception with early IUC as implemented for standard IUC (51, 56).

If shown to be successful, early IUC insertion will help women avoid unplanned and unwanted pregnancy following medical treatment of incomplete abortion, thereby averting the 18% preventable maternal mortality from unsafe abortion in Uganda. The study findings will guide in formulation of policies on the ideal time frame to insert IUC after medical PAC for first trimester incomplete abortion both locally and internationally as most of the randomized trials have been done in Europe and US mostly with induced medical abortions (30, 48, 70).The study findings will ultimately enable women enjoy their right of planning their pregnancies and families.

1.4 JUSTIFICATION:

Uganda has a low LARC use of 0.5%, despite having a national policy on LARC (14, 71). With return of fertility for most women from as early as five days to two weeks after first trimester abortions, it's advisable that early post abortion contraception is instituted to reduce on missed opportunity of using an effective family planning method(22). Uganda lacks a policy on the ideal timing for placement of intrauterine contraception after medical management of first trimester incomplete abortions. Undertaking this study in Uganda will therefore aid in formulating evidence based practices on when IUC can be instituted after medical management of first trimester incomplete abortion thereby improving the quality of Post abortion care given to women(72).

Knowing the safety profile on when to institute IUC after medical PAC would reduce on the unintended pregnancies and their associated morbidity and mortality.

It's against this background that this study seeks to determine the effectiveness, continued use, uptake and acceptability of early insertion of IUC (within week after medical PAC) compared to standard IUC insertion (at 2-4 weeks post PAC) in Central Uganda with the ultimate aim of increasing the uptake of PAC services after incomplete abortion.

1.5 HYPOTHESIS

1.5.1 Hypothesis 1:

1.5.1.1 Null hypothesis:

Early Insertion of IUC (within one week) has higher expulsion rates than standard insertion (at 2-4 weeks) after medical management among women with first trimester incomplete abortion at six months within a non-inferiority margin of 5%.

1.5.1.2 Alternate hypothesis:

Early Insertion of IUC (within one week) has similar expulsion rates as standard insertion (at 2-4 weeks) after medical management among women with first trimester incomplete abortion at six months within a non-inferiority margin of 5%.

1.5.2. Hypothesis 2:

1.5.2.1 Null hypothesis:

Early Insertion of IUC (within one week) has higher continued use as compared to the standard insertion (at 2-4 weeks after medical management among women with first trimester incomplete abortion) at six months within a non-inferiority margin of 5%.

1.5.2.2 Alternative hypothesis:

Early Insertion of IUC (within one week) has similar continued use as standard insertion (at 2-4 weeks) after medical management among women with first trimester incomplete abortion at six months within a non-inferiority margin of 5%.

1.6 RESEARCH QUESTIONS:

1. What proportion of women take up Intrauterine Contraception after 1st trimester incomplete abortion?
2. What are the predictors of the Intrauterine Contraception uptake after 1st trimester incomplete abortion? Is there any difference in the expulsion rates at six months between early versus standard IUC insertions post mPAC treatment following incomplete abortion?
3. Are there any differences in the IUC expulsion and continuation rates at six months between early versus standard IUC insertion post mPAC treatment following incomplete abortion?
4. What are the women and their spouses' perceptions on LARC and IUC following mPAC treatment for incomplete abortion?
5. What are the Healthcare providers' perceptions on long-acting Reversible contraceptives (intrauterine contraception) following mPAC treatment for incomplete abortion?

1.7 General objective:

- To determine whether early insertion of IUC (within one week after medical PAC) has similar effectiveness, continued use, and acceptability as standard IUC insertion (at 2-4 weeks post medical PAC) among women managed for first trimester incomplete abortion.

1.7.1 Specific objectives:

1. To determine the proportion of women who take up IUC after 1st trimester incomplete abortion.
2. To determine the factors associated with the post abortion intrauterine contraception uptake after 1st trimester incomplete abortion.

3. To compare the expulsion rates at six months between early versus standard IUC insertions among women managed with mPAC treatment for first trimester incomplete abortion.
4. To compare the IUC continuation rates at six months between early versus standard IUC insertion among women managed with mPAC treatment for first trimester incomplete abortion.
5. To explore the Healthcare providers' perception on IUC following mPAC treatment for first trimester incomplete abortion.
6. To explore the women and their spouses' perception on IUC following mPAC treatment for first trimester incomplete abortion.

1.8 CONCEPTUAL FRAME WORK

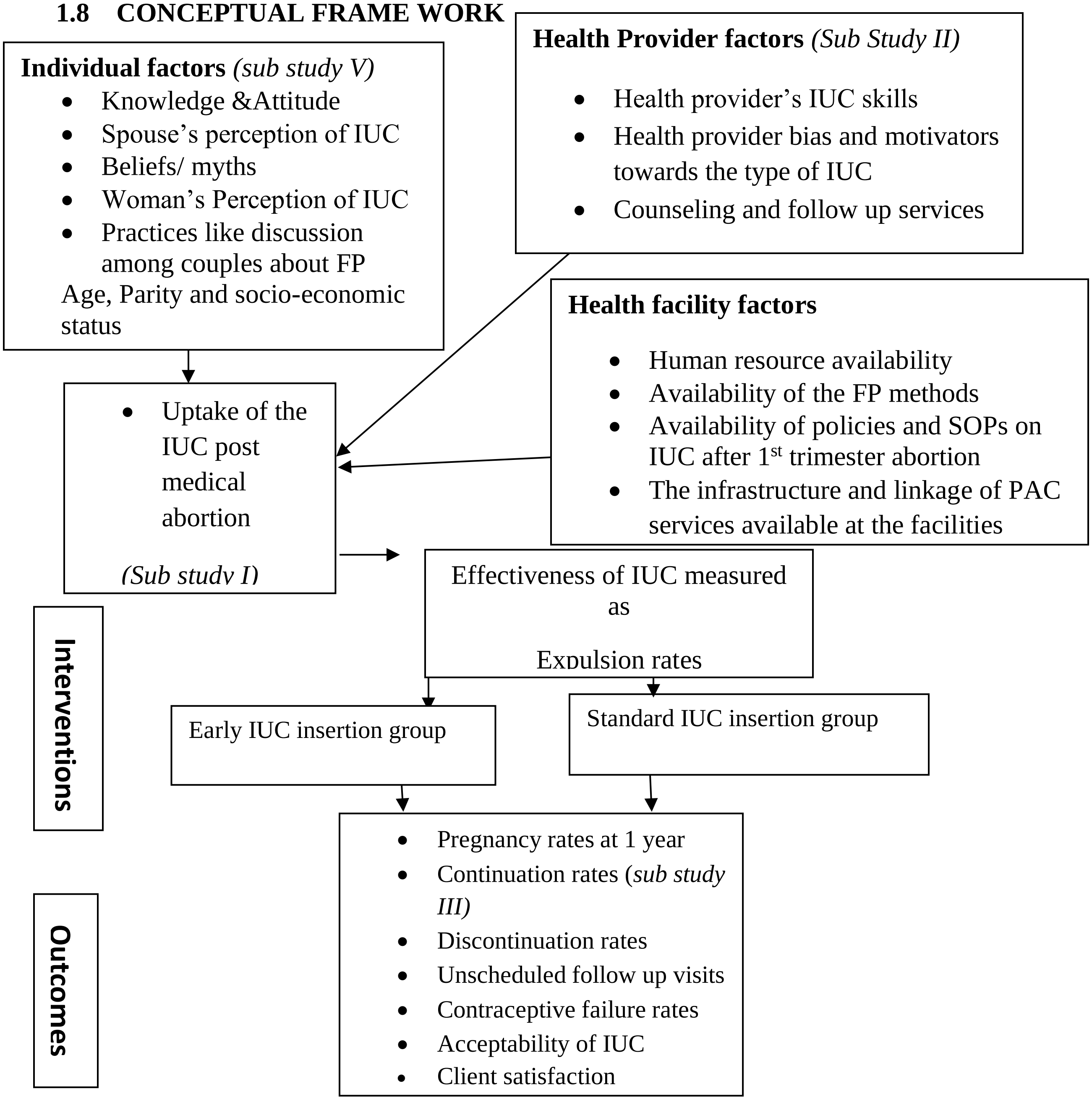

Fig 1: Healthcare providers' and patient characteristics that influence contraceptive decision making and uptake. Sub-study 1 *focuses on establishing the uptake of post abortion contraception and the factors that determine the uptake after medical management for incomplete abortion*. Sub-study II seeks *to determine the expulsion and continuation rates between early versus standard insertion six months after abortion IUC after first trimester medical management of incomplete abortion*. Sub-study *III explores the women's and their spouses' perception towards* post abortion IUC after first trimester medical management of incomplete abortion. *Sub-study IV seeks to explore the health providers' perceptions towards post abortion contraception* as this is key in *policy formulation and implementation.*

CHAPTER TWO:

2.1 LITERATURE REVIEW:

2.1.0 Epidemiology:

2.1.1 Burden of Unintended pregnancies:

Globally, 222 million women have an unmet need for contraception, with 43.8 million pregnancies being unintended. This results in 21.6 million unsafe abortions which ultimately end up leading to 11% of maternal deaths (70, 73). These maternal deaths would otherwise have been prevented with effective family planning methods (74). According to the World Health Organization, the higher proportions of unsafe abortion occur in Africa and Asia (75) contributing to 97% of the global burden (7, 76). This burden of unsafe abortions is driven by, restrictive laws (6), knowledge gaps in contraception, inaccessibility to modern contraceptive methods especially in rural areas, minimal resource allocation for family planning services and limited skills among healthcare providers (3). As a result of these factors, the case fatality rate from unsafe abortions in Africa is 460 deaths per 100,000 live births as compared to 30 deaths per 100,000 live births in high income countries (7).

About five million women who undergo unsafe abortion end up with chronic disability (56), infertility, pelvic inflammatory disease, and psychological issues like Anxiety (10) which deprives them of realizing their sexual and reproductive health rights on the number and spacing of their children (77). According ipas, 38-68% of the burden of women with complications from unsafe abortions are adolescents yet even this could be an underestimate as many don’t show up for treatment (78).

2.1.2 Burden of unsafe Abortions in Uganda:

Uganda has a high unmet need for contraception of 30% and a high fertility rate of 5.4 children per woman (72) which in turn leads to 56% of the pregnancies being unintended (15). The Uganda laws are restrictive in regards to abortions with the only window of deviation in cases of incest or to save the woman’s life (79). This pushes many Ugandan women with unwanted and unintended pregnancies to unsafe abortions “that are either performed by a person lacking the necessary skills or in an environment that does not conform to minimal medical standards” (80). Singh et al (17) reported of the 314,304 women who undergo unsafe abortions each year in Uganda, about 128,682

experience complications with only 85,000 obtaining care. About 28% and 18% of the maternal mortality occur among adolescents and other Ugandan women from complications of unsafe abortions in face of restrictive abortion laws (13). Mbonye (31) reported that growing burden of unsafe abortions in Uganda is driven by use of ineffective contraceptive methods, knowledge gaps on the available options, and inaccessibility of family planning services to a significant proportion of Ugandan women.

2.2 Return of fertility after first trimester abortion and Repeat pregnancy:

The return of fertility for most women after first trimester pregnancy losses is as early as five days to ten days after first trimester abortions (18, 19, 20). Evidence shows that more than 50% of the women resume sexual intercourse within two weeks after first trimester pregnancy losses (21). It's therefore advisable that early post abortion contraception is instituted. As this would reduce on missed opportunity of using an effective family planning method(22). Women undergoing abortion are at increased risk of subsequent unintended pregnancies. Repeat abortions account for 40-47% of all abortions (23, 43). Prior studies have shown that about 42% of the women scheduled for post abortion contraception never return for follow up visits for reasons like; long distances to travel, financial constraints and other social demands predisposing them to unintended pregnancies (24, 25, 26, 27, 28, 29, 30). There is also evidence that early repeat pregnancies are associated with adverse maternal and fetal outcomes such as repeat miscarriages, preterm labour, preeclampsia and fetal demise (57).

2.3 Description of Abortions:

Abortion refers to a pregnancy loss prior to viability which can either be spontaneous or induced. Though the true incidence of spontaneous abortion is hard to determine, it's thought that 15% of clinically evident pregnancies and 60% of chemical pregnancies end up as spontaneous abortions. The cause of most cases of spontaneous abortion is unknown. However, abnormal karyotype account for about 50% of first trimester spontaneous abortions. Other causes of spontaneous abortion are infections, anatomic defects, endocrine factors, immunologic factors and maternal systemic diseases (51).

There are different classifications of abortion.

Threatened abortion: This type occurs in presence of uterine bleeding with or without lower abdominal pain and the cervical os is closed. The management is usually conservative though

progesterone derivatives (like Duphaston), Salbutamol and some tocolytics (like Nifedipine) have been used to manage the condition.

Missed abortion: There is retention of the dead embryo or fetus prior to viability. It could present with slight uterine bleeding, regression of pregnancy symptoms. Missed abortion mimics threatened abortion. However, ultrasonography would confirm a missed abortion when fetal heart activities are not demonstrated.

Inevitable abortion: The diagnosis is made when there is dilatation of the cervix and intrauterine bleeding without expulsion of any products of conception (51).

Incomplete abortion: Sometimes spontaneous abortions could be incomplete. In these instances, uterine bleeding is usually associated with lower abdominal pains, the cervical os is open with some retention of products of conception. It may be possible to see products of conception at the cervical os.

Complete abortion: Complete abortion occurs when all the products of conception are expelled and the cervical os is closed. Due to the closed os, it may be difficult to diagnose but, ultrasonography may aid in confirming the diagnosis.

Induced abortion: Induced abortion may be medical or surgical, and medications used to induce abortion include Mifepristone, Methotrexate and Misoprostol. Surgically-induced abortion could be done with the manual vacuum or electric aspirator.

2.4 Medical Management of First Trimester abortions:

Medical management which involves the use of medication to induce or complete an abortion, has comparable effectiveness when compared to surgical aspiration for first trimester abortions(11). Evidence shows that medical management is 95% effective in achieving a complete abortion especially when given prior 63 days of gestation (81), though it has also been reported to effective even after 63 days of gestation (82). It gives the notion of being more natural, private, doesn’t require anesthesia and safer to the women as compared to the surgical management (83, 84). Women feel more involved in their management unlike in the surgical interventions where the clinicians do most of procedures with the patients taking the back seat (11). Products of conception are expelled within hours of taking the medications such as Misoprostol, with more than three quarters of the women completing the abortions within the first 24 hours (54).

Medical management however demands longer duration to achieve complete abortions and can at times give women more cramping and par vaginal bleeding as compared to the surgical options (83). The commonly used drug for medical management of incomplete abortion in Africa is Misoprostol.

In Africa, the uptake of medical management is about 40% as compared to the 19% in Asia(32) with benefits of being used in far to reach health facilities and communities where surgical management for incomplete abortions are not readily available.

2.5 Infection rates after medical management of abortions:

A systematic review of 65 studies by Shannon shows that pelvic infections are rare after medical management with only 0.9% of infections being reported(85). Medical management is therefore as safe as surgical management of first trimester incomplete abortion.

2.6 Choice of drugs for medical management:

The World Health Organization recommends Misoprostol in the medical management of incomplete abortions (86) in Africa.

2.7 Route of Administration, dose of Misoprostol in Medical management:

The incidence of adverse effects follows the dose and route of administration of Misoprostol. Lesser gastrointestinal side effects follow the vaginal as compared to the oral, buccal or sublingual routes (87, 88, 89). The vaginal, buccal and sublingual routes have a quicker onset as compared to the rectal or oral routes with efficacy rates exceeding the 92% approved by FDA for gestation age up to 63 days of gestation (87, 90).

According to the updated FIGO recommendations for incomplete abortion 2017, sublingual Misoprostol 400 mcg or oral Misoprostol 600 mcg administered as a single dose has been found to be very effective(91).

Whether singleton or multiple gestation, the doses and regimens of Misoprostol are the same according to Hayes et al (92) for incomplete abortion.

2.8 Safety margin of medical management of abortion:

On the overall, less than 1% of the patients managed with medication might require emergency curettage or blood transfusion for heavy bleeding according to prior studies (93, 94, 95, 96).

2.9 Antibiotic use in medical management of abortion:

According to Sawaya in a meta-analysis on the use of prophylactic antibiotics in abortion care, more than half of the post abortion endometritis could be prevented with the antibiotic use (97). Use of single dose Doxycycline and Metronidazole along with medical management of incomplete abortion reduces infection rates by 75% (98, 99, 100, 101).

2.10 Pain control in medical management of abortion:

Prior to discharging patients after medical management for incomplete abortions, they should be given appropriate analgesia. Non-steroid Anti-inflammatory drugs appear to be superior to Acetaminophen in pain management in medical abortion (102). Ibuprofen doesn't appear to interrupt with the effects of Misoprostol(103). Narcotics can also be used whenever appropriate in medical abortion.

2.11 Description of Post Abortion Contraception:

2.11.1 Early post abortion IUC insertion:

Post abortion IUC insertion could be early (immediate) or at an interval (standard or delayed). When an IUC is inserted the same day or within a week after an induced abortion or complete spontaneous miscarriage, it is termed as early IUC insertion. Insertion of an IUC immediately after an abortion has several potential advantages. The woman is known not to be pregnant and it avoids repeat unintended pregnancies despite return to sexual activity and of ovulation. For example, many clinicians refuse to insert an IUC in a woman who is not menstruating (25).

After an abortion, a woman's motivation to use contraception may be high, and for women who have limited access to a clinician, post abortion care may provide a unique opportunity to address a woman's need for contraception (68, 104, 105). In addition, insertion of an IUD immediately after abortion may avoid discomfort related to insertion, and any bleeding from the insertion will be disguised as the expected bleeding after abortion (69).

Less than a third of women who intend to have an IUD after abortion may actually have one inserted (25). Insertion of an IUD immediately after a pregnancy loss carries concerns over expulsion of the IUD due to dilated cervix and risk of perforation may be increased due to softening of the myometrium (24, 60). Other studies have reported no difference in the expulsion rates,

perforation, or discontinuation rates between early versus standard post abortion intrauterine contraceptive insertion (24, 61, 62, 63, 106).

Another potential concern is pelvic inflammatory disease (PID), particularly when post abortion IUD insertion is done after a clandestine or unsafe abortion which increases the risk of upper genital tract infection compared with interval insertion(107). A systematic review of 65 studies by Shannon however disputes this showing that pelvic infections are rare after medical management with only 0.9% of infections being reported(85). Other studies have also reported that the Pelvic inflammatory rates are similar between early and standard IUC insertion (108, 109, 110). Insertion after a septic abortion is however a contraindication to IUC insertion (40, 51).

2.11.2 Interval or Standard post abortion IUC insertion:

In the USA, IUC insertion is routinely done at 10–14 days at the time of post medical abortion follow-up (111). The timeframe for interval insertion is between two to six weeks after medical management of abortion (30, 51, 111). Standard post abortion IUD insertion allows time for the woman to come to terms with the loss of a wanted pregnancy after a spontaneous miscarriage and affords a woman who had an induced abortion the opportunity to be sure of her choice of contraceptive since a visit is required post abortion(30). However, observational studies have reported that 40% of clients do not return for insertion after opting for interval IUD insertion (112) and that the additional visit is a barrier to delayed IUD insertion (23). The reasons for failing to honor their follow up appointments include long distances to travel to the health facilities, no social support, financial constraints and also health facilities are so crowded (12, 24, 29, 30, 48, 50).

2.12 Mechanism of Action of Post Abortion Intrauterine Devices:

2.12.1 Copper IUDs:

Intrauterine contraceptive devices act locally on the endometrium causing inflammatory reaction. Copper-IUDs are thought to have spermicidal actions, and their effect on the endometrium interferes with normal development of ova or fertilization of ova. It also promotes phagocytosis of sperm cells and inhibits the movement of sperm cells from the vagina to the fallopian tubes where fertilization occurs. It is also thought that the inflammatory changes of the endometrium may prevent implantation of the embryo should fertilization occur (113).

2.12.2 Levonorgestrel Releasing Devices:

Hormone-impregnated IUDs release the progestin into the maternal circulation daily. The Levonorgestrel acts primarily by thickening the cervical mucus, thereby impeding the ascent of sperm cells. It also inhibits ovulation, especially following insertion when the woman's serum concentration is relatively high. For instance, Levonorgestrel-20(LNG-20) IUD causes anovulation in approximately 10-15% of cycles and changes the endometrium to reduce the likelihood of implantation (113).

2.13 Early versus Standard insertion:

While the mechanism of action of IUDs is irrespective of whether insertion is early or standard, early IUD insertion would afford the woman an opportunity to have an effective contraceptive method earlier, before resumption of sexual activities. This would prevent an unplanned pregnancy and the need for a repeat induced abortion, or space the interval between a spontaneous miscarriage and the next planned pregnancy (30, 40, 51, 76).

2.14 Factors Associated with the Uptake of Post Abortion Contraception:

A number of patient and health system factors have been highlighted in prior studies however their applicability to our setting is yet to be ascertained. Such factors that influence the uptake of post abortion contraception include; Younger age, Misconception about IUD uptake among nulliparous women, prior induced abortion, consent from partner on the choice of contraception, prior use of IUDs, peer influence, cost of contraception, quality of contraceptive service delivery, difficulty in contacting the service providers, logistics challenges, planned pregnancy, stock-outs of supplies and materials. There are social, legal, religious restrictions, limited skills from health workers, knowledge gaps on contraceptive options, lack of administrative coordination between the abortion and contraceptive service delivery (32, 78, 114)

2.15 Relevance of studying Early versus Standard Post Abortion Contraception:

Whereas the WHO recommends use of intrauterine contraception immediately after manual vacuum aspiration following incomplete abortion, placement of intrauterine contraception after medical management with Misoprostol has been between 2-4 weeks (20, 25, 50), as there's barely any published evidence on the safety of early post abortion contraception after medical abortion(51, 52, 53) especially in low resource settings where access to surgical management is not assured in a number of facilities. An effective post abortion contraceptive, that is long-lasting

and convenient like intrauterine contraception needs to be instituted as soon as possible. The IUDs, being similar to sterilization in terms of contraceptive efficacy meet these criteria (112, 115), yet simpler, less expensive, and promptly reversible. Less than one woman out of 100 with typical use of IUDs becomes pregnant in the first year of using IUDs (40).

In face of restrictive abortion laws in Uganda, this study seeks to determine the uptake, acceptability effectiveness, and continued use, of early insertion of IUC (within week after medical PAC) compared to Standard IUC insertion (at 2-4 weeks post PAC in first trimester incomplete abortion) in Uganda with the ultimate aim of increasing the uptake of PAC services. Determining the healthcare providers' and patients' perception towards Post abortion contraception will as well help policy makers to identify facilitators and barriers of the uptake of IUC as they formulate effective and feasible policies to mitigate the burden of unsafe abortions. The study findings might enable more women locally and internationally to access post abortion contraception thereby preventing unwanted and unintended pregnancies.

CHAPTER THREE: METHODS:

3.1 Study sites:

The study will be implemented in 5 health facilities in Central Uganda. The central region was selected due to the high abortion rate compared to the national average (62 vs. 39 per 1,000 live deliveries) and the accompanying huge case load of women treated for abortion complications (17, 116, 117). The study sites will include Entebbe hospital, Mpigi HC IV, Masaka hospital, Gombe hospital, Nakaseke hospital, Luweero HC IV, Mengo hospital, Naguru hospital, Kawempe National referral hospital, Kawolo hospital, Kiganda HC IV, Wakiso HC IV, Kasangati HC IV, and Kayunga hospital. We shall also seek referrals from faith-based hospitals that don't offer modern contraceptives like Lubaga hospital, and St Francis hospital Nsambya. We shall also work with Kampala City Authority health facilities to enable us access and refer women with first trimester miscarriages to our study centres.

The 5 sites were purposively selected because of the high patient loads and that prior family planning studies have been conducted successively before in these settings. The infrastructures for family planning research are already in existence at these sites.

All the study sites are located within 1-2 hours from Kampala, the capital city of Uganda. The selected study sites are either public or private-not–for-profit health facilities, equipped to provide comprehensive emergency obstetric services in rural, periurban and urban areas. The facilities identified for inclusion in the trial have sufficient staff to establish a group of 250 midwives and 80 physicians and sufficient caseload between 50-150 cases a month (See Table 1).

Routinely at the health facilities, women presenting with symptoms and signs of incomplete abortion are triaged at the emergency ward/OPD. History taking and physical examination are conducted by the attending health providers. Pregnancy dating is done by calculating the weeks of gestation from the first day of the last menstrual period or by ultrasound. Laboratory examinations are usually undertaken if the patient is found anemic or has other co-morbidities like fever. Other investigations are performed based on the symptoms and clinical examination findings.

Details of the health facilities are shown in table 1.

Table 1: Caseload per facility (three months) and staffing July- September 2021

District	**Facility***	**Location**	**Nature of Health facility**	**Bed capacity**	**Uptake of PAC/IUDs**	**Other Contraceptive method**	**cumulative 1st trimester Abortion load in the 3 months (July-Sept 2021**	**Total abortions recorder**
Kampala	China-Uganda Friendship hospital	Located on Naguru Road, on Naguru Hill.<br><br>It is found in Nakawa Division; Kampala District. The hospital is about four kilometers (2 miles), by road, east of the central business district of Kampala.	Public hospital	100				
Wakiso	Entebbe Hospital	Central business centre of Entebbe.<br><br>Its 44 Km by	Public<br><br>(General hospital)	200			32	59

		road Southwest of Mulago Hospital						
Mpigi	Mpigi HC IV	38 km southwest of Mulago Hospital	Public<br><br>(General hospital)	100		02	72	8
Masaka	Masaka Hospital	Found in the central business centre of Masaka town.<br><br>132 kn southwest of Mulago Hospital	Public (Regional referral hospital)	400		-	155	3
Gombe	Gombe Hospital	Found in the Central business centre of Gombe.<br><br>It’s found 70 km southwest of Mulago Hospital	Public<br><br>(General hospital)	260		11	76	257
Nakaseke	Nakaseke Hospital	Found in Luweero Triangle<br><br>Located 65Km Northwe	Public (General hospital)	120		22	50	82

		st of Mulago hospital						
Luweero	Luweero HC IV	Found in Luweero town.<br><br>It's located 60km north of Mulago Hospital	Public (Health Centre IV)		-	08	76	11
Mityana	Mityana Hospital	Located in the Central Business Centre of Mityana.<br><br>It's found 69 km west of Mulago Hospital	Public<br><br>(General hospital)	100		22	52	26
Mukono	Mukono HC IV	It's found along Kampala-Jinja highway, 20km East of Mulago hospital.	Public (Health Centre IV) mainly on an out-patient basis	12		10	36	9
Kassanda	Kiganda HC IV	It's located 56 km East of Mubende, 110 km west	Pulbic (Health centre IV) serving a rural		9	38	52	-

		of Mulago hospital	population					
Wakiso	Wakiso HC IV	Found along Kampala-Busunju Express road<br>It's 29 km Northwest of Mulago Hospital	Public (Health Centre IV) serving a population of 20,000	-		-	-	-
Wakiso	Kasangati HC IV	It's found along Kampala-Gayaza road 13km Northeast of Mulago hospital<br>It's a teaching hospital for Makerere University	Public (Health Centre IV) serving a population of about 150,000	40		-	-	-
Kampala	Kawempe National Referral Hospital	Its located 12 km along Gulu-Kampala	Public (National referral hospital also a teaching hospital	900	40	-	484	-

		highway. Serves a population of 4.5 million,	for Makerere University).					
Buikwe	Kawolo Hospital	It's found along Kampala-Jinja Highway, 50 km east of Mulago Hospital	Public (General hospital)	106		-	-	-
Kayunga	Kayunga Hospital	It's found 67.5 km northeast of Mulago hospital along	Public (Regional referral hospital)	200		212	212	-
	**Total**					**95**	**71**	**105**

**Source - Health facility records July-September 2021*

3.1.2 Overall Study design:

Upon consenting to participate in the study, women managed with medical management for first trimester incomplete abortion, will be recruited. The participants will be given information on the available post abortion contraceptive methods. Those who opt to take on intrauterine contraception with no contraindications will be recruited into a cross sectional study (sub study 1) that will have 650 participants to understand the factors that influenced their decision to take up post abortion IUC after medical management of 1st trimester incomplete abortion. Findings of the sub study will be used to inform the main RCT study and possibly give insights on how to improve participant retention in the RCT (sub study 2). The main study will be a non-inferiority RCT that will seek to determine the expulsion and continuation rates of "Early IUC insertion" (within one week after

mPAC) and “Standard IUC insertion” (between 2-4 weeks after mPAC) for 1st trimester incomplete abortion in Central Uganda at six months. To answer the objective on the expulsion rate, and the second objective of the RCT, continuation rates by six months after “Early vs. Standard IUC insertion, 1,000 participants will be recruited. To understand the barriers and facilitators influencing the acceptability of post abortion IUC among the couples and healthcare providers in Central Uganda, 20-30 in depth interviews will be conducted among women with or without their spouses, using the IUC from the main study (Sub study 3). About 20-30 in depth interviews among healthcare providers of different cadres involved in the main study will be conducted to explore their perceptions on post abortion IUC after medical management of 1st trimester incomplete abortion (sub study 4). We anticipate achieving data saturation by the end of these in-depth interviews. More of the details in regards to the study designs, sample size calculation for the different sub studies will be found in the individual sub study write up.

3.2 SUB-STUDY I: UPTAKE AND FACTORS ASSOCIATED WITH UTILIZATION OF POST ABORTION INTRAUTERINE CONTRACEPTION AFTER FIRST TRIMESTER MEDICAL MANAGEMENT OF INCOMPLETE ABORTION IN CENTRAL UGANDA

3.2.1 Objectives:

1. To determine the proportion of women who take up Intrauterine Contraception after medical management of 1st trimester incomplete abortion.
2. To determine the factors associated with the intrauterine Contraception uptake after medical management of 1st trimester incomplete abortion.

3.2.2 Study Design:

This will be a cross sectional study in which the proportion of women opting to take up post abortion intrauterine contraception after medical management of first trimester abortion will be determined out of the total population of women who will have received medical management for 1st trimester incomplete abortion over the study period at the 15 study centres.

3.2.3 Study design Justification:

There has been a relatively low uptake of intrauterine contraception locally of 0.5%(71, 72). A cross sectional study design is feasible, doesn’t require a lot of time and wouldn’t disrupt the RCT process yet availing us with the data snapshot on post abortion contraception. The findings would in turn help to streamline patient retention in the RCTs as addressing the barriers to the post abortion contraceptive uptake could improve the follow up in the main study.

3.2.4 Participant recruitment:

The criteria for identifying study participants with incomplete abortion will be based on experience of any of the following conditions: a confirmation of pregnancy by a urine HCG, ascertaining the gestation age by ultrasound scan or from the first day of the last menstrual period, history of lower abdominal pain, or cramps, par vaginal bleeding, before 12 weeks of gestation. The research assistants will identify and approach the potential participants willing to undertake medical management for first trimester incomplete abortion from the emergency gynaecology units. Post abortion contraceptive counseling will be offered to all women. Interested individuals in using post

abortion contraception will be given all the required information on intrauterine contraception to enable them make an informed decision to participate in the study.

3.2.5: Data collection process:

Upon consenting to participate in the study, a detailed patient history and examination to confirm an incomplete abortion will be obtained. The choice of contraception chosen will be recorded. Those participants who opt for post abortion intrauterine contraceptive within four weeks of medical management of first trimester incomplete abortion will be interviewed in detail to establish the factors that influence their choice using interviewer administered questionnaires that will be in English or Luganda. Data will also be retrieved from the patients' records. In cases of under or over documentation in the patients' records, both the participants and the care team will be consulted for clarity.

3.2.5.1 Inclusion criteria:

- Women 15 years and above having taken medical management for first trimester incomplete abortion within the past four weeks and are willing to participate in the study will be recruited.

3.2.5.2 Exclusion criteria:

- Participants too sick to participate in the study.
- Participants confirmed to have uterine anomalies like bicornuate uterus as documented from prior medical records will be excluded.

3.2.6 Sampling procedure:

All eligible women with first trimester incomplete abortion managed medically and willing to use post abortion contraception meeting the inclusion criteria will be recruited upon giving informed consent at the different study sites. With a sample size of 900, we shall randomly select 60 participants from each of the 15 health facilities. A pretested standard questionnaire will be administered by the trained staff on participants' socio-demographic characteristics, contraceptive choices and predictors of the post abortion intrauterine contraceptive uptake.

3.2.7: Procedure for Intrauterine contraception placement:

Women will be free to decide between the Cu-IUD (Nova T, Bayer AG, Berlin, Germany) and the LNG-IUS (Mirena H, Bayer AG, Berlin, Germany).

Medical PAC will be performed according to the evidence-based guidelines. In our study, women

with first trimester incomplete abortion diagnosed on history taking and clinical examination, will be given Misoprostol 400 mcg sublingually as a single dose(91). Prior to this, two Ibuprofen 200 mg tablets every 8 hours for three days and two Paracetamol 500mg tablets will be taken orally for three days(102). In case additional analgesia will be needed, it will be availed to the patients. As prophylaxis against post abortion endometritis, two Metronidazole 200mg tablets taken orally every 8 hours for five days and one Doxycycline 100mg tablet twice daily for five days will be given to all participants (21, 98, 100, 101). After receiving the medical PAC for incomplete abortion, the participants will go home unless if they have medical reasons for hospitalization. For the early insertion group, the intervention will be within a week of medical PAC while the standard group, the IUC will be inserted between 2-4 weeks after medical PAC.

3.2.8.1 Steps of the IUD insertion:

The following standardized protocol for IUC insertion will be used for early as well as standard insertion after medical PAC for first trimester incomplete abortion. The woman will be placed in supine position with her legs in stirrups. A speculum will be inserted into the vagina and a tenaculum placed on the cervix to straighten the uterus. A sound will be inserted when thought appropriate into the uterus to measure the length to the fundus and thereafter the IUC will be placed at the fundus of the uterus. Threads will be cut at 3 centimeters length. The woman will put a vertical line on a VAS (10cm horizontal line) to indicate pain before insertion, at tenaculum and sound placement, IUC insertion, and before discharge.

3.2.9 Sample size calculation:

Using a study that assessed post abortion contraceptive uptake among young women in 10 countries in South Asia and Africa, the overall uptake of IUDs was 11%(118), using OpenEpi, we shall need 190 participants to answer this objective.

3.2.9.1: Sample size calculation for factors associated with Post Abortion Intrauterine Contraception after first Trimester Medical PAC (secondary outcomes):

Using a study by Makenzius (37), that looked at the "***Contraceptive uptake in Post Abortion Care: Secondary outcomes from a Randomized trial in Kisumu, Kenya***", Age groups of 21–25 (OR: 2.35; p< 0.029) was independent associated with contraceptive uptake in Post abortion care. Using OpenEpi info sample size calculator, with OR of 2.35, power of 80%, two -sided confidence level

of 95% and level of significance of <0.05, we get a sample size for associated factors of 642 participants.

Calculating for sample size adjusting for clustering,

Sample size = Calculated Sample size * Design Effect (DE)

$DE = 1 + (n - 1)\,\rho$

Where n = number of participants per cluster and ρ is the Intra-cluster coefficient (119)

According to Rowe(120) in "***Design effects and intra-class correlation coefficients from a health facility cluster survey in Benin"*** *the* median ICC was observed at 0.2 (After adjustment for participant and cluster level characteristics) and Design effect of 1.4 were reported from their analysis of 46 health facility surveys.

Considering the sample size calculation of 642,

For 15 clusters (units), average cluster size = 642/15 = 43

Applying the formula, we get DE = 1.4

Overall sample size = 43 * 1.4 *15 = 890

With each of the clusters have 60 participants

We shall need a sample size of 900 participants, to determine both our primary and secondary objectives for the study

.

Sample Size:X-Sectional, Cohort, & Randomized Clinical Trials			
Two-sided significance level(1-alpha):			95
Power(1-beta, % chance of detecting):			80
Ratio of sample size, Unexposed/Exposed:			1
Percent of Unexposed with Outcome:			5
Percent of Exposed with Outcome:			11
Odds Ratio:			2.4
Risk/Prevalence Ratio:			2.2
Risk/Prevalence difference:			6

	Kelsey	Fleiss	Fleiss with CC
Sample Size - Exposed	321	320	352
Sample Size-Nonexposed	321	320	352
Total sample size:	642	640	704

References
Kelsey et al., Methods in Observational Epidemiology 2nd Edition, Table 12-15
Fleiss, Statistical Methods for Rates and Proportions, formulas 3.18 &3.19
CC = continuity correction
Results are rounded up to the nearest integer.
Print from the browser menu or select, copy, and paste to other programs.

3.2.9 Primary outcome: Uptake of intrauterine contraception after medical PAC for first trimester incomplete abortion.

3.2.9: Secondary outcomes: Post abortion sepsis, duration of bleeding, repeat pregnancies will also be assessed

3.2.9.10: Predictor variables: Socio-demographic characteristics like Age, parity, socio-economic status, education background, peer pressure, misconceptions surrounding post abortion contraception, health facility factors like availability of the contraceptive mix, coordination of the family planning services, healthcare providers' skills of IUC insertion will be assessed in relation to the uptake of the IUC after mPAC for 1st trimester incomplete abortion.

3.2.10 Analysis plan:

Baseline participant characteristics will be analysed using descriptive statistics like mean, standard deviation and proportions. Uptake of the IUC will be determined by the computing the proportion of women who leave with IUC as their contraceptive method out of the women who receive

medical management for first trimester incomplete abortion expressed as a percentage. Categorical variables such as level of education, socio-demographic background, parity and the post abortion Intrauterine contraception will be analysed using a Chi-square with the level of significance at <0.05. For continuous variables, Fisher's exact test, ANOVA will be used as appropriate. Multilevel logistic regression model will be used for factors associated with the uptake of intrauterine contraception with p value set <0.05 as level of significance. Odds ratios and confidence intervals will be used to describe associations between the contraceptive uptake and the different variables.

3.2.11 Quality Control:

3.2.11.1 Staff training and recruitment:

Nurse-midwives familiar with the local hospital settings will be enrolled for research training for three days. They will be trained on how to identify potential participants. They will be also trained on participant recruitment while observing the research ethics in accordance to the Declaration of Helsinki(121). Thirty research assistants will be then selected to collect the data in 15 study sites over the study period. The nurse-midwives will also be trained on how to identify emergencies like severe haemorrhage following the abortion, need for blood transfusion, genital infections like septic abortion and the procedures to undertake so as to inform the obstetric team on duty so that the affected participants obtain timely emergency care to save their lives.

A pilot study will be carried out to pretest and modify the data collection tools. Completed data collection tools will be checked for completeness and thereafter edited, coded and entered on same day of collection. Data will be backed up. The database will be password protected and the participants' records will be kept under limited access in a lockable cabin. The research materials will be kept under restricted access by only authorized staff for patient confidentiality and privacy.

3.3 SUB-STUDY 2: EFFECTIVENESS OF EARLY VERSUS STANDARD INSERTION OF POST ABORTION INTRAUTERINE CONTRACEPTION AFTER FIRST TRIMESTER MEDICAL MANAGEMENT OF INCOMPLETE ABORTION IN CENTRAL UGANDA: A NON–INFERIORITY RANDOMIZED CLINICAL TRIAL

3.3.1 Study design:

This will be a Randomized Clinical Trial to investigate IUC expulsion rates and continued use at six months, of early insertion of IUC (within one week after a medical PAC) compared with standard insertion (at 2-4 weeks post PAC) among women managed for first trimester incomplete abortion in Central Uganda.

3.3.2: Rationale for study:

When an IUC is inserted the same day or within a week after an abortion, it is termed as early IUC insertion. Early IUC insertion after an abortion has several potential advantages. The woman is known not to be pregnant and it avoids repeat unintended pregnancies even if resumption of sexual activity or ovulation could be within two weeks of the evacuation. Hesitancy of many clinicians to insert an IUC in a woman who is not menstruating (25) could be prevented with the early insertion of the IUDs. After an abortion, a woman's motivation to use contraception may be high, and for women who have limited access to a clinician, post abortion care may provide a unique opportunity to address a woman's need for contraception (68, 104, 105). In addition, insertion of an IUD immediately after abortion may avoid discomfort related to insertion, and any bleeding from the insertion will be disguised as the expected bleeding after abortion (69). Less than a third of women who intend to have an IUD after abortion may actually have one inserted (25).
There are concerns however with the insertion of an IUD immediately after a pregnancy loss such as increased chances of expulsion of the IUD due to dilated cervix and risk of perforation may be increased due to softening of the myometrium (24, 60).

Standard post abortion IUD insertion (2-4 weeks after first trimester abortion) allows time for the woman to come to terms with the loss of a wanted pregnancy after a spontaneous miscarriage and affords a woman who had an abortion the opportunity to be sure of her choice of contraceptive since a visit is required post abortion(30). However, observational studies have reported that 40% of clients do not return for insertion after opting for interval IUD insertion (112) and that the

additional visit is a barrier to standard IUD insertion (23). The reasons for failing to honor their follow up appointments include long distances to travel to the health facilities, no social support, financial constraints and also health facilities are so crowded (12, 24, 29, 30, 48, 50).

It's to this end that this study seeks to determine the ideal time of insertion of intrauterine contraception after medical management of first trimester incomplete abortion to guide in formulation of evidence-based policies to improve the contraceptive uptake in Uganda and beyond.

3.3.3 Objectives:

- To compare the expulsion rates at six months between early IUC and standard IUD insertions among women managed with mPAC after first trimester incomplete abortion.
- To compare the IUC continuation rates at six months between early IUC and standard IUD insertion among women managed with mPAC after first trimester incomplete abortion.

3.3.4 Hypothesis 1:

3.3.4.1 Null hypothesis:

Early Insertion of IUC (within one week) has higher expulsion rates than standard insertion (at 2-4 weeks) after medical management among women with first trimester incomplete abortion at six months within a non-inferiority margin of 5%.

3.3.4.2 Alternate hypothesis:

Early Insertion of IUC (within one week) has similar expulsion rates as standard insertion (at 2-4 weeks) after medical management among women with first trimester incomplete abortion at six months within a non-inferiority margin of 5%.

3.3.5 Hypothesis 2:

3.3.5.1 Null hypothesis:

Early Insertion of IUC (within one week) has higher continued use as compared to the standard insertion (at 2-4 weeks) after medical management among women with first trimester incomplete abortion) at six months within a non-inferiority margin of 5%.

3.3.5.2 Alternative hypothesis:

Early Insertion of IUC (within one week) has similar continued use as standard insertion (at 2-4 weeks) after medical management among women with first trimester incomplete abortion at six months within a non-inferiority margin of 5%.

Study Profile (122):

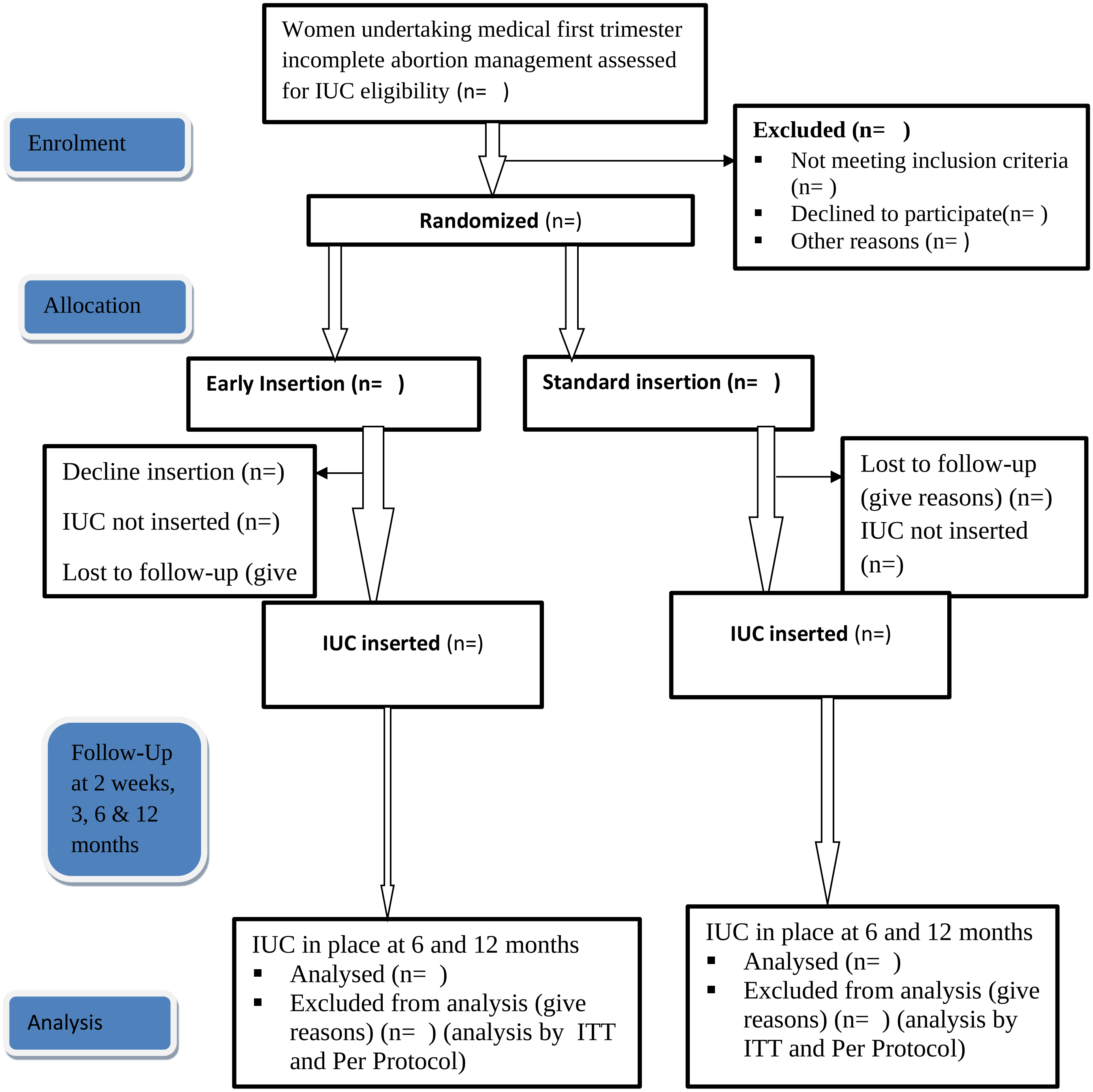

Fig 2: Randomization and follow up of study participants in the "Early" and "standard" insertion of post abortion intrauterine contraception after first trimester medical management for incomplete abortion in a ratio of 1:1.

3.3.6 Study Population:

Women with first trimester incomplete abortion undergoing medical management will receive written and oral information about the study from the attending physician according to the

principles of the Helsinki Declaration and have an opportunity to ask questions. If the woman agrees to participate and is deemed eligible, she will sign informed consent in the presence of the attending physician at that consultation. This procedure will not differ between study sites. The FIGO guidelines on medical PAC will be followed (www.figo.org).

3.3.6.1 Inclusion criteria:

- Women 15 years and above, eligible for medical management of first trimester incomplete abortion, staying within ten kilometers from the health facility, opting for post abortion IUC with no intention of conceiving within one year, able and willing to comply with the planned follow up.

3.3.6.2 Exclusion criteria:

- Contraindications for medical PAC or IUC such as patients with known allergies to copper or Levonorgestrel IUDs, patients with unsafe or septic abortions, confirmed cervical cancer or with suspicious Pap smear cytology results demanding more work up, hemoglobin level below 9g/dl, active genital tract infections, coagulopathies, known uterine anomalies, and suspected ectopic pregnancies will be excluded. Women with mental health issues that make it hard for them to comprehend the study protocols will be offered the standard of care but will be excluded from the study.

3.3.7 Sample size and calculated power:

3.3.7.1 Justification of power calculation:

It is assumed that IUC placement following medical PAC can be compared to that following medical management of incomplete abortion with regard to the main outcome (expulsion rates at six months post medical PAC, no significant difference expected between groups) and safety (123). A non-inferiority RCT study design will be used. With an assumed 95% “success” (non-expelled IUDs) in both groups with a power of 90 percent and an alpha of 0.05 we would need to randomize 500 women in each group.

Choosing the bigger sample size and 20 percent loss to follow up which is commonly seen in studies on abortion. Thus, we will randomize 1,000 women. Analysis will be presented as intention to treat for all randomized women as well as per protocol for women completing the six months follow up. Follow up at two weeks, three, six and 12 months will be performed by visit or telephone.

3.3.7 Variables and measures:

3.3.7.1 Primary outcome measures:

- Expulsion rates at six months following insertion in both groups evaluated by follow up.
- Continued use of IUC at six months post insertion evaluated at follow-up at two weeks then three- and six-months post abortion (complete, partial or no expulsion). The exact day of expulsion will be noted to determine the risk of expulsion as a function of time (post insertion).

- **3.3.7.2 Secondary outcome measures**:
- Ease of insertion according to health care provider (judged as easy, moderate, or very difficult).
- Pain at time of insertion (women will indicate the pain before insertion, at placement of tenaculum, sound and IUC by putting a vertical mark on a 10 cm long horizontal line with a Visual Analogue Scale (VAS) from 0, indicating no pain to 10 indicating worst imaginable pain.
- Post abortion bleeding (number of days of fresh bleeding and spotting after the abortion)
- Complications (adverse events (AE) and serious AE) bleeding requiring any treatment, uterine perforations and cervical tears, infection requiring treatment with antibiotics, hospitalization for any reason, surgical procedures due to heavy bleeding, incomplete abortions, prolonged bleeding or patient request,
- Pregnancies occurring during the 12-month follow-up (planned and unplanned, wanted and unwanted, pregnancy outcomes- ectopic, miscarriage, abortion, molar, or kept pregnancy),
- Discontinuation rates at 6 and 12 months will also be determined.

3.3.7.3 Predictor variable:

Timing of the IUC insertion after medical management of 1st trimester incomplete abortion will be assessed on its impact on the expulsion and continuation rates of the IUC at six months

3.3. 8 Randomization for study:

The randomization sequence will be generated using STATA 12 software package in a ratio of 1:1 allocation using permuted block size of 4, which will be varied randomly. The investigators will not participate in randomization process. The randomization will be performed by the Biostatistician. This process will be accomplished before the study starts. The group assignments will be kept in sealed envelopes, which will only be opened by the study nurse after informing and

recruiting the participants. The randomization list will remain with the Biostatistician throughout the study period. The investigators and research assistants will not access the randomization list. Intermittent check-ups that ensure that the intervention procedures are adhered to, will be routinely ensured.

3.3.9 Allocation concealment:

Sequentially numbered, identical, opaque, sealed envelopes prepared by staff otherwise not involved in the trial to conceal the pre-specified intrauterine contraception allocation from clinicians, research personnel, and participants will be used. The randomization sequence will be generated by a biostatistician not affiliated with the trial. After screening, women who will be eligible for IUC will be randomized to receive early or standard insertion of IUC by a different team from the one that ensured allocation.

3.3.10 Blinding:

In this study, we shall have no blinding because the continuation and expulsion rates following "Early or standard intrauterine contraception" after medical management of first trimester incomplete abortions are objective. The participants will make a choice on whether to take up copper or Levonorgestrel intrauterine devices from the outset. The study therefore will be an open label design.

3.3.11 Choice of Study Design:

The study will be performed as a randomized, controlled, non-inferiority trial. It is known that socio-demographic factors such as parity and age may influence the choice of contraception and the rate of continued use. To eliminate these confounders the randomized design is non-inferior.

3.3.12 Trial process and data collection:

3.3.12.1 Enrolment:

All women with incomplete first trimester abortions managed medically with Misoprostol opting for a post abortion IUC will be invited to be included in the study at the initial outpatient consultation. The women will receive detailed oral and written information regarding the study. An informed consent will be signed by the research assistant and the woman before randomization and any other study related activity.

3.3.12.2 Allocation and treatment:

Designated study nurses and doctors will be responsible for recruiting and examining study participants at the outpatient clinic. Eligible women who choose to participate in the study and

who sign an informed consent form will be randomized into either:
Group 1; early insertion (insertion within 1 week of PAC) or Group 2; standard insertion (follow-up and insertion at 2-4 weeks post abortion)

Women will be free to decide between the Cu-IUD (Nova T, Bayer AG, Berlin, Germany) and the LNG-IUS (Mirena H, Bayer AG, Berlin, Germany).
Medical PAC will be performed according to the evidence-based guidelines. In our study, women with first trimester incomplete abortion diagnosed on history taking and clinical examination, will be given Misoprostol 400 mcg sublingually as a single dose(91). Prior to this, two Ibuprofen 200 mg tablets every 8 hours for three days and two Paracetamol 500mg tablets will be taken orally for three days(102). In case additional analgesia will be needed, it will be availed to the patients. As prophylaxis against post abortion endometritis, two Metronidazole 200mg tablets taken orally every 8 hours for five days and one Doxycycline 100mg tablet twice daily for five days will be given to all participants (21, 98, 100, 101).

After receiving the medical PAC for incomplete abortion, the participants will go home unless if they have medical reasons for hospitalization. For the early insertion group, the intervention will be within a week of medical PAC while the standard group, the IUC will be inserted between 2-4 weeks after medical PAC. All efforts such as phone calls, text messages and home visits will be underway to reach out to the participants in case they fail to return to the health facility for the different interventions. Transport reimbursements will be given to encourage the participants to make it for the scheduled visits.

Evidence has it that routine evaluation of retained products of conception after medical management of incomplete abortions using ultrasonography can lead to erroneous diagnosis of incomplete abortions often leading to unnecessary interventions that could even be harmful to the women (124, 125, 126). Ultrasonography also has a poor predictive value in estimating the risk of expulsion of intrauterine contraception after medical management of abortion (30). It is against this backdrop that ultrasonography will only be used in cases where incomplete expulsion of products of conception is suspected from the patient history and clinical examination despite the single Misoprostol dose of 400 mcg (127, 128). After the Misoprostol treatment, if there will be

no complete expulsion, the nurse-midwife or doctor on duty will surgically evacuate the uterus. Women who will be identified to develop clinical presentation suggestive of sepsis will be managed as per the clinical guidelines. All of such participants will however be analysed by the Intention-To-Treat protocol.

All women will be encouraged to abstain from unprotected sexual intercourse for two weeks prior to insertion of the post abortion intrauterine contraceptive to prevent any unintended pregnancies. All costs pertaining to the IUDs, the post abortion care, all the required investigations outside the standard of care offered by the public facilities for the study participants will be met by the research team.

3.3.13 Procedure:

The following standardized protocol for IUC insertion will be used for early as well as standard insertion after medical PAC for first trimester incomplete abortion. The woman will be in supine position with her legs in stirrups. A speculum will be inserted into the vagina and a tenaculum placed on the cervix to straighten the uterus. A sound will be inserted when thought appropriate into the uterus to measure the length to the fundus and thereafter the IUC will be placed at the fundus of the uterus. Threads will be cut at 3 centimeters length. The woman will put a vertical line on a VAS (10cm horizontal line) to indicate pain before insertion, at tenaculum and sound placement, IUC insertion, and before discharge.

3.3.14 Follow up:

All participants will be followed up for at least one year. Follow up visits will be scheduled at two weeks, three, six and twelve months of IUC insertion. At each scheduled visit, the participants will undertake standardized history and physical examination including general, abdominal and pelvic examinations. If a participant misses a scheduled visit by two weeks she will be contacted by phone, reminded of the missed visit and rescheduling of her appointment done. We shall also request for contacts for the participants' next of kin at recruitment as backup in case the participants' contacts are inaccessible on the scheduled visits. Efforts will be made to get directions to the participants' homes at recruitment into the study. Home visits will be made for participants who will be lost to follow up. At every visit, the women will be assessed clinically for possible development of PID. They will be asked about development of complications and whether they

received any medication from other sources. Information about IUD removal, IUD expulsion, heavy or prolonged menstrual bleeding or missed menstrual periods and hormonal side effects related to progesterone will be ascertained. Women will be requested to keep notes on the menstrual changes on all follow up visits. The acceptability of the IUD will be assessed using a Likert scale. At the last visit, participants will be assessed on their level of satisfaction with the IUC, and whether they would recommend a friend to use IUD, if they would use the IUD in the future or whether they would wish to continue using the IUC.

3.3.15 Analysis plan:

The primary outcomes, expulsion rates and continued use rates of IUC at six months will be analyzed by a generalized estimating equation with trial center treated as a random factor. Independent variables between the Early vs. Standard insertion groups will be assessed to determine whether they are similar. The database will contain information on demographic variables (age, gravidity, parity and mode of delivery, number of medical abortions), which will be analyzed by Mann Whitney U-test. Length and weight of women will be calculated into BMI and compared by Student´s t-test. Previous contraception will be compared by proportions and Fisher´s exact test or Chi^2-test depending on number of cases.

Type of IUC inserted will be analyzed by proportions and Chi^2-test for the two different types of IUC used (IUCs available in Uganda), sound measurement will be analyzed by Chi^2-test, ease of insertion according to inserter (judged as easy, moderately difficult or very difficult) will be analyzed by Chi^2-test, patients' experience of insertion will be analyzed by Student´s t-test (women will indicate the pain on a VAS, from 0, (no pain) to 10 (worst imaginable pain). The result will be noted as millimeters and entered into the CRF), bleeding and spotting total number of days will be analyzed using Cox regression analysis and visualized by Kaplan Meier curves. Expulsion rates (yes, partial expulsion or no, on which day post insertion) will be analyzed using Chi^2-test or Fischer´s exact test depending on cases. Expulsion as a function of time will be analyzed using Cox regression analysis and visualized by Kaplan Meier curves. Extraction of IUC for any other reason and subsequent contraceptive method prescribed will be shown as proportions and analyzed by Chi^2-test. Surgical intervention and reason for this will be analyzed by proportions and Chi^2-test. If the woman would recommend IUC as post abortion contraception and if she would

recommend “Early” or “standard” insertion of IUC to a friend will be analyzed by Chi^2-test (asked at discharge, three and six month follow up).

In addition, multivariate regressions that explore influence of parameters on outcome measures and interactions between various covariates and primary and secondary outcomes will be performed using logistic regression analysis for dichotomous parameters and after dichotomization of continuous parameters. Interaction variables may be introduced if appropriate. An intention to treat analysis as well as per protocol analysis will be performed.

3.3. 16 Participant Retention in the study:

The research team will ensure that the participants are comprehensively informed of the follow up schedule with every effort made to have the follow up visits during working hours from Monday to Friday every week. For those who find it very hard to adhere to this schedule, a window will be availed over the weekend. A phone call and text message will always be used to remind the participants at least 72 hours prior to their scheduled visits. Two contacts of next of kin will be required of the participants in addition to their personal mobile phone numbers. These contacts will always be used in case the patients’ contacts are unavailable. Transport reimbursements will be given to all study participants at every follow up visit. Participants who make it to the end of the study will also be given a token of appreciation in monetary terms. This will be disclosed to them at the end of the study. Participants who fail to make it for the follow up visit will also be called to identify any reasons for failure to honor their appointments. We shall review and analysis the reasons for non-adherence continuously and use the recommendations from the audits to minimize the loss to follow up. A readily available contact of a study nurse will be offered to all participants over 24 hours every week to address any issues that they will have with the study intervention or process. This will ensure timely response to the participants’ concerns thereby creating a bond between the participants and the study team.

A participant will be regarded as loss to follow up if they fail to honor their appointments after one month of every attempt to have them come back fails. A phone call and text message will be sent out twice a week for the whole month.

3.3.17 Data security:

All databases will be accessed only by the personnel directly involved in the study. The consents and the questionnaires will be kept separately in a lockable research office. Study participants will not be identified by name in the final report.

3.3.18 Data Monitoring:

A multidisciplinary committee comprising of a pharmacist, gynecologist, social worker and one member of the IRB will form the Data Safety and Monitoring Board (DSMB). This committee will report to the steering committee which will comprise of the Doctoral committee. To avoid conflict of interest, the DSMB will have no funders or any of the members involved in the implementation of the research. They will have access to the study findings and will report to the steering committee, interim analysis results with recommendations on whether to halt or continue with the study. Measures to ensure cross-validation of the data entry for any errors, on-site evaluation will also be ensured in the study. Quality assurance will be observed by two members of the research team to ensure completeness of all the data collected.

3.3.19 Trial Auditing:

A committee of experts in the study to oversee the data control process will be constituted. This committee will work with monitors, trial coordinators and investigators. The committee will have a responsibility of educating; supporting and helping the study team resolve any issues which will emerge in the study. The study will have two monitors who will be responsible in ensuring that the protocol is adhered to in regards to the quality data entry, adverse events documentation, report writing and submission. They will work with the study coordinators to ensure that the right information is collected from the participants and rightly entered in the database. The monitors will also work with the coordinators to ensure that they can do timely communication, and query response of all events with trial coordinating centre. They will ensure that the coordinators learn how to trouble shoot and resolve the issues that arise in the process. The monitors will also work with the coordinators to ensure that all the required documents like IRB clearance and correspondence forms, case report forms, patient consent forms, study agreements, adverse events forms, study protocols, institution clearance forms and all source documents like patient charts are filed in a binder that can easily be retrieved whenever needed. The monitors will notify the teams at least a fortnight prior to their visit to ensure timely preparations. They will ensure that all the 5 study sites adhere to the protocol requirements.

3.3.20 Modifications in the Protocol:

In cases of any amendments in the study objectives, major administrative changes, sample size, potential benefits or safety concerns, the UNCST and SOMREC IRBs will be notified by the study Principal Investigator. Minor amendments like changes in the administration that don't affect the running of the study will also be communicated at the discretion of the steering committee as a memorandum to the IRBs.

3.3.21 Confidentiality:

All study related information will be kept secure at the study sites. Patient data will be kept in password protected databases. Patient files and records will be kept in lockable cabins with limited access. Only the steering committee and DSMB will have full access to the information. Patients will be assured that their information will never be shared with any third parties without their written consent. Patient identifiers will never be kept with the study findings to ensure confidentiality in the study. The research team will sign confidentiality forms to ensure that they conform to keeping the participants' confidentiality at all times during and after the study.

3.3.22 Stopping rules:

An interim analysis of results will be performed when 50 percent of women have been recruited. If expulsion rates exceed 20 percent, the study will be stopped. As recommended by Korn (129), when the Hazard ratio equals or exceeds the preselected non-inferiority margin for our study of 5% at 50 percent recruitment, DSMB will stop the study. This will enable us avoid harm to the study participants.

3.3.23 Adverse Events:

Any untoward medical occurrence without regard to the possible causality in a study participant, in our study will be defined as an adverse event. All adverse events will be recorded from the point the participants sign the consent forms to discharge from the hospital. In the event that the participants get the adverse event after signing the consent form but before receiving the post abortion contraception, the adverse event won't be registered as following the intervention.

All serious adverse events will be reported within seven working days to the SOMREC IRB. In this study, any untoward medical occurrence leading to life threatening conditions like death, permanent disability, prolonged hospitalization or significant hazard as regarded by the Safety Monitoring committee, as reported by the investigations to be casually related with post abortion intrauterine contraception, will be regarded as "serious adverse events". Any serious adverse

events that occur however in the participants after being discontinued from the study won't be regarded unless if the investigators are compelled to the notion that the occurrence was related to the post abortion intrauterine contraception. The study personnel will document the circumstances surrounding the events and the reasons for discontinuing the study intervention.

Within 24 hours of occurrence of the adverse events, the investigators will set out to establish the relation of the events whether unexplained or unexpected based on the documentation in the consent process or protocol and post abortion intrauterine devices. The adverse events will as well be evaluated in perspective with the participant's prior medical condition, medications and prior clinical course before placement of the post abortion intrauterine contraception.

Uterine perforation, need for exploratory laparotomy after post abortion intrauterine device insertions, need for blood transfusion following severe hemorrhage, hospitalization over 72 hours, anaphylactic reaction after IUD insertion, life threatening sepsis, or deaths will be regarded as severe adverse events. Participants with adverse events will be managed immediately by the care teams at the different facilities as per the standard operating procedures. Those who need referral services will be given the best care at the expense of the research team.

3.3.24 Quality control:

The Principal Investigator will carry out a trial run for a month to pre-test all study instruments, streamline the process of enrolment, allocation and follow up at the health facilities prior to starting data collection. All research assistants involved in the study will undergo training on research conduct prior to the study. All health workers participating in the study will undergo a standard five-day course in post abortion care and key areas to be covered include: making a diagnosis, uterine evacuation with Misoprostol and surgical methods, adjunct services to be provided, contraceptive counselling and provision, intrauterine contraception and the other available family planning options. A coordination team will be set up to supervise the study and offer support. All filled or recorded data will be checked on a daily basis for completeness. A Data safety monitoring board will be formed to monitor patient safety during the implementation of the RCT.

The trials will be registered at ClinicalTrials.gov and reported in accordance with CONSORT guidelines.

3.3.25: Post-trial access plan:

The steps that will be followed at the end of the trial are highlighted in Appendix XXII. The plan details processes like how feedback about the study findings will be given to the participants, the steps that will be followed to close out the different study sites. Plans to ensure accountability and stewardship after the trial and measures that will be followed to transition participants into the existing care are highlighted in the Appendix. Since both the early and standard groups will receive the post abortion intrauterine contraception after medical management of first trimester incomplete abortion, in the event that one intrauterine device (copper or Levonorgestrel) is found to be superior to the other, the study participants will be offered first priority to change as they may so wish without incurring any financial costs.

3.4 SUB-STUDY 3: HEALTHCARE PROVIDERS' PERCEPTIONS TOWARDS POST ABORTION INTRAUTERINE CONTRACEPTION AFTER FIRST TRIMESTER MEDICAL MANAGEMENT OF ABORTIONS IN UGANDA: A QUALITATIVE STUDY

3.4.1 Study Design:

This will be a qualitative study in which healthcare providers of different cadres offering family planning services at the different 15 health facilities will be interviewed on their perception towards Post Abortion intrauterine contraception. We shall use the grounded theory (a methodological approach that generates theory from data that has been systematically collected and analyzed using comparative analysis) (130) and inductive thematic approaches(131) to explore the perceptions of healthcare providers as they offer post abortion intrauterine contraception after medical management of incomplete abortions. We shall conduct in depth interviews among healthcare providers of different cadres offering post abortion contraception while observing the COVID-19 infection prevention protocols.

3.4.2 Aim:

This study will seek to explore the perceptions of Healthcare providers towards the provision of IUC after medical management of first trimester incomplete abortion with particular emphasis on barriers and facilitators of the IUC.

3.4.3 Research questions:

1. How do Healthcare providers perceive post abortion LARC provision, IUD in particular?
2. What are the most important barriers to post abortion LARC provision and uptake?
3. What are the most important facilitators to post abortion LARC provision and uptake?

3.4.4 Participant recruitment and sampling:

Prior to participant recruitment, permission from the SOMREC institutional review board will be sought. After obtaining permission, from the different hospital administrators, healthcare providers providing family planning services based on their availability and convenience will be approached. Emphasis will be made so that healthcare providers both male and female, of different cadres offering family planning services at the different facilities especially in Post abortion care will be

included in the study. These will include obstetricians/gynecologists, nurse midwives, and medical doctors, in the family planning clinics. The selected healthcare providers will then be given two contacts of the Principal investigator and the research team. All participants will be sanitized, and will have COVID-19 symptom screening prior to the interviews. All the in depth interviews will be administered in English, the official language used in Uganda.

3.4.5 Inclusion criteria:

All Healthcare providers actively involved in family planning services in Post Abortion care at any of the 15 selected health facilities during the study period who will consent to participate in the study will be recruited.

3.4.6 Exclusion Criteria:

- Healthcare providers offering in family planning services at the 15 selected health facilities who might be on leave or inaccessible physically during the study period will be excluded.

3.4.7 Sample size and sampling procedure:

In this study 20-30 in-depth interviews will be conducted among with healthcare providers offering family planning services at the 15 different health facilities(119). Healthcare providers involved in provision of family planning especially in the post abortion care at the different health facilities will be purposively identified. Thematic analysis will be used to generate themes and subthemes. We plan to do the purposive sampling, data collection and analysis concurrently so as to achieve data saturation. When no new themes or codes will be arising, we shall conduct 2-3 more interviews to ensure saturation(132).

3.4.8 Staff training and recruitment:

Two researchers familiar with the local hospital settings with expertise in qualitative research will be enrolled for research training for three days. They will be trained on how to identify and interview potential clients. They will be also trained on participant recruitment while observing the research ethics in accordance to the Declaration of Helsinki(121). Two field note takers fluent in English will also be recruited.

3.4.9 Data collection:

After obtaining informed consent from the participants, 20-30 healthcare providers will have in depth interviews conducted by research assistants during the study period. All interviews will be administered in English. Two note takers will capture the participants' non-verbal expressions with their consent in addition to the field notes. After ascertaining data saturation with no new emerging themes or codes from the analysis, we shall stop the data collection (133, 134). The interviews will last between 45 to 90 minutes. The interviews will capture the participant socio-demographic information, the way they perceive post abortion contraception after medical management of first trimester incomplete abortion, the facilitators and barriers to uptake of Post abortion intrauterine contraception using open ended questions. Where further clarity will be needed, more specific questions will be raised by the interviewers so that all the required information is obtained. All of the interviews will be tape recorded and transcribed verbatim immediately thereafter. Transcription accuracy will be ensured at the end of the interviews by the Principal investigator and one Administrator. Field notes, audio files and the transcripts will be compared for congruency.

3.4.10 Quality control:

Two interviewers and two note takers will be trained prior to the data collection. A pilot study will be carried out with four healthcare providers to pretest and modify the interview guide. The interviews will be tape recorded and transcribed verbatim immediately after the interviews. The

transcriptions will be compared with field notes throughout the study period. Data will be backed up. The research materials will be kept under restricted access by only authorized staff for patient confidentiality and privacy.

3.4.11 Data analysis:

Data analysis, sampling and collection will be conducted concurrently in the pilot until data saturation is achieved. This will be done so that insights from the data analysis will be used to make the required adjustments in the interview guide. When no new themes or codes will be arising from the new data collected, we shall confirm data saturation there by halting further data collection. This will aid in the data collection and also test the credibility of the emerging themes in the subsequent interviews.

All interviews will be transcribed fully and assessed for accuracy by the research team. The research team will listen to interviews several times. The participants' voices will be later on transcribed using the "listen N write" software word-for-word. To ensure trustworthiness and credibility, two independent researchers will read and review the content (interview transcripts, and field notes word-for-word, line-for-line) several times. Field notes and interview transcripts from each of the interviews will be assessed individually and later integrated to strengthen the data analysis and dependability of the study findings.

Using the Social constructivism worldview, (that explores the notion of collaboration within healthcare providers to accomplish the provision of post abortion contraception after first trimester medical management of incomplete abortion, using cultural, contextual, situational analysis constructs, and social interaction as opposed to their individual cognitive abilities to solve IUC insertion challenges) as reported by Crotty (135) with the inductive thematic approach (a content

analysis approach that generates theories from the available dataset with no prior predetermined theory) (131), the research team will take the following steps to generate themes. The steps will include; data will be prepared by typing out the interviews, thereafter using sentences, phrases or paragraphs, will be used to generate meaning units from the context of the participants' voices. We shall then convert the concepts generated into codes (text coding) using semantic tags. The primary codes will then be generated and meaning units shortened to formulate 'compressed meaning units'. We shall later revise the text codes comparing similarities and differences between the codes thereby integrating what appears as similar codes. We shall then critically look at all the transcript steps and codes and classifying them based on their relationships or differences. We shall ensure reliability of the codes, and then revise the classes. We shall keep comparing the codes from the data generated. Similar codes will be put into subcategories and these subcategories will be later put into the main themes.

In cases of disagreements, the research team will discuss until an agreement is reached. We shall thereafter identify themes reflecting on the depth. We shall later compare with other classes so as to delimit the theories towards intrauterine contraception after medical management of first trimester incomplete abortion. We intend to achieve conceptual congruency by making efforts to ensure a fit from the gaps surrounding post abortion intrauterine contraception, the use of context specific methods and ensuring rigorous data collection and analysis to understand the barriers and facilitators of the post abortion intrauterine contraceptive uptake among the healthcare providers (136).

3.4.12 Rigor:

To ensure rigor in the data collection, Guba and Lincoln criteria(137), that will include, data credibility, confirmability, transferability and dependability will be used. We shall observe data credibility by ensuring checks by two members of the research team to accept codes from the transcription, and peer debriefing from the senior researchers. We shall ensure long term involvement of the research team with the healthcare providers. Data dependability will be ensured by having two members of the research team devoted to continuous reading through the transcripts to ensure ongoing comparison of the key information being generated during the data collection and analysis processes. Dependability will be observed by the stringent coding procedure and inter-coder corroboration. Two independent researchers will analyze the data to ensure investigator triangulation. Data transferability will be observed by ensuring that participants' statements are captured with barely any modifications made yet ensuring a rich, thick description of the study process by the research team. Thorough checks of procedures and results will be emphasized to improve the dependability and transferability of the data(138). Confirmability will be observed by comparing the results to other evidence and field notes by an external investigator (139)who will read and compare the study results with the field notes and memos. We shall ensure that the coordinators of the interviews or discussions do not participate in the analysis but will critique the results from the analysis and ensured that these results will conform to their expectations from the discussions. Field notes, and transcripts will be available for ongoing analysis by independent investigators.

3.5 SUB-STUDY 4: WOMEN AND THEIR SPOUSES' PERCEPTIONS TOWARDS POST ABORTION INTRAUTERINE CONTRACEPTION AFTER FIRST TRIMESTER MEDICAL MANAGEMENT OF INCOMPLETE ABORTIONS IN UGANDA: A QUALITATIVE STUDY

3.5.1 Study Design:

This will be a qualitative study in which women enrolled in the main study ("comparing expulsion rates, effectiveness, continued use, and acceptability of Early versus Standard IUC insertion (at 2-4 weeks post medical management of first trimester incomplete abortion) with or without their spouses will be interviewed on their perceptions towards Post Abortion intrauterine contraception. We shall use the grounded theory and inductive thematic approaches(131) to explore the perceptions of women with or without their spouses (female sexual workers, those with multiple sexual partners, those with rape and defilement issues) towards post abortion intrauterine contraception. We shall conduct in depth interviews while observing the COVID-19 infection prevention protocols.

3.5.2 Study Aim:

To explore the perceptions of women and their spouses on post abortion contraceptive uptake and use, LARC in particular. Specifically we want to gain a deeper understanding of post abortion fertility planning and contraceptive use, as well as how women who have chosen to use an IUC, navigate socio-cultural factors and gender norms and power relations that discourage use of the IUC.

3.5.3 Research questions:

1. What are the women's and their spouses' perceptions surrounding LARC and the IUD in particular?
2. Considering the many socio-cultural beliefs and practices, gender norms and power relations that discourage modern contraceptive use, how do women who have chosen to initiate mPAC IUD assert their agency and negotiate use?
3. How do current social and gender norms including norms about sexuality and reproduction influence reproductive agency in relation to contraceptive use?

3.5.4 Participants:

In-depth interviews (IDIs) will be held with women with or without their spouses (female sexual workers, those with multiple sexual partners, those with rape and defilement issues) of varying ages, who have continued or discontinued their IUC use past the six months' follow up. Potential participants will be invited to participate at the six months follow up visit. A total of 20 to 30 women and men will be interviewed. Each interview will take around 45 to 90 minutes and be recorded. We shall purposely sample couples who opt to take on IUC after medical post abortion treatment, those who continue or discontinue their intrauterine contraception. We shall also seek to determine the power dynamics involved in the decision making to either take on or decline IUC as we discuss with the individual spouses in the in depth interviews. An inductive thematic analysis to identify the specific phenomenon behind uptake of IUC will be used.

3.5.5 Participant recruitment and sampling:

Prior to participant recruitment, permission from the different hospital institutional review boards will be sought. After obtaining permission, the different nurses in charge of the participant recruitment in the study will seek to meet the women who will have accepted post abortion intrauterine contraception and their spouses based on their availability and convenience. We shall purpose to meet couples that have continued or discontinued post abortion intrauterine contraception at six months. The selected women and their spouses will then be given two contacts of the Principal investigator and the research team to establish rapport even before the interviews. We plan to do the purposive sampling, data collection and analysis concurrently so as to achieve data saturation. When no new themes or codes will be arising, we shall conduct 2-3 more interviews to ensure saturation(132). We shall purposively interview 20-30 women with or without their spouses using the post abortion intrauterine contraception at the 15 selected health facilities using in depth interviews till we achieve data saturation. We shall conduct some of the in depth interviews with the women alone and others with the spouse alone to see if more themes can emerge with the power dynamics that could be in existence in the different relationships.

COVID-19 prevention protocols will be adhered to prior to the interviews (see Appendix XXII). All the in depth interviews will be administered in English, or Luganda (the commonest local language used in Uganda).

3.5.6 Inclusion criteria:

- Women with or without their spouses above 15 years old, eligible for medical management of first trimester incomplete abortion, using post abortion IUC, able and willing to participate in the study.

3.5.7 Exclusion criteria:

- Women who obtain post abortion care after 1st trimester abortion but opt to use other contraception other than IUC will be excluded.

3.5.8 Staff training and recruitment:

Two researchers familiar with the local hospital settings with expertise in qualitative research will be enrolled for research training for three days. They will be trained on how to identify and interview potential participants. They will also be trained on participant recruitment while observing the research ethics in accordance to the Declaration of Helsinki(121). Two field note takers will also be recruited fluent in English and Luganda.

3.5.9 Data collection:

After obtaining informed consent from the participants, 20-30 women with or without their spouses will have in depth interviews by research assistants during the study period. All interviews will be administered in either English or Luganda. Two note takers will capture the participants' non-verbal expressions with their consent in addition to the field notes. When no new themes or codes will be arising from the new data collected, we shall confirm data saturation (133, 134). The interviews will last between 45 to 90 minutes. The interviews will capture the participant socio-demographic information, the way they perceive post abortion contraception after medical management of first trimester incomplete abortion, the facilitators and barriers to uptake of Post abortion intrauterine contraception using open ended questions. Where further clarity will be needed, more specific questions will be raised by the interviewers so that all the required information is obtained. All of the interviews will be tape recorded and transcribed verbatim immediately thereafter. Transcription accuracy will be ensured at the end of the interviews by the Principal investigator and one Administrator. Field notes, audio files and the transcript will be compared for congruency. The Luganda transcripts will be translated to English immediately after the interviews or at least within 24 hours of data collection to ensure that the content and context of the transcripts are not lost with time.

3.5.10 Quality control:

Two interviewers and two note takers will be trained prior to the data collection. A pilot study will carried out with four couples using post abortion intrauterine contraception to pretest and modify the interview guide. The interviews will be tape recorded and transcribed verbatim immediately after the interviews. The transcriptions will be compared with field notes throughout the study period. Data will be backed up. The research materials will be kept under restricted access by only authorized staff for patient confidentiality and privacy.

3.5.11 Data analysis:

All interviews will be transcribed fully and assessed for accuracy by the research team. The research team will listen to interviews several times. The participants' voices will be later on transcribed using the "listen N write" software word-for-word. To ensure trustworthiness and credibility, two independent researchers will read and review the content (interview transcripts, audio files and field notes word-for-word, line-for-line) several times. Field notes and interview transcripts from each of the interviews will be assessed individually and later integrated to strengthen the data analysis and dependability of the study findings.

Using the Social constructivism worldview as reported by Crotty (135) with the inductive thematic approach (131), the research team will take the following steps; data will be prepared by typing out the interviews, thereafter using sentences, phrases or paragraphs, will be used to generate meaning units from the context of the participants' voices. We shall then convert the concepts generated into codes (text coding) using semantic tags. The primary codes will then be generated and meaning units shortened to formulate 'compressed meaning units'. We shall later revise the text codes comparing similarities and differences between the codes thereby integrating what appears as similar codes. We shall then critically look at all the transcript steps and codes and classifying them based on their relationships or differences. We shall ensure reliability of the codes, and then revise the classes. We shall keep comparing the codes from the data generated. Similar codes will be put into subcategories and these subcategories will be later put into the main themes.

In cases of disagreements, the research team will discuss until an agreement is reached. We shall thereafter identify themes reflecting on the depth. We shall later compare with other classes so as to delimit the theories and achieve conceptual congruency(136).

3.5.12 Rigor:

To ensure rigor in the data collection we shall use Guba and Lincoln criteria(137), that will include, data credibility, confirmability, transferability and dependability. We shall observe data credibility by ensuring checks by two members of the research team to accept codes from the transcription, and peer debriefing from the senior researchers. Long term involvement of the research team with the healthcare providers will be ensured. Data dependability will be ensured by having two members of the research team devoted to continuous reading through the transcripts to ensure ongoing comparison of the key information being generated during the data collection and analysis processes. Dependability will be observed by the stringent coding procedure and inter-coder corroboration. Two independent researchers will analyze the data to ensure investigator triangulation. Data transferability will be observed by ensuring that participants' statements are captured with barely any modifications made yet ensuring a rich, thick description of the study process by the research team. Thorough checks of procedures and results will be emphasized to improve the dependability and transferability of the data(138). Confirmability will be observed by comparing the results to other evidence and field notes by an external investigator (139)who will read and compare the study results with the field notes and memos. We shall ensure that the coordinators of the interviews or discussions do not participate in the analysis but will critique the results from the analysis and ensured that these results will conform to their expectations from the discussions. Field notes, and transcripts will be available for ongoing analysis by independent investigators.

3.6 Ethical Considerations:

Ethical approvals will be obtained from SOMREC, UNCST, and the Ministry of Health of Uganda. Administrative permissions will be sought from the department of Obstetrics and Gynaecology at Makerere University College of Health Sciences, the directors of the different implementing health units and Nongovernmental organizations like Marie Stopes Uganda. The trials will be registered at ClinicalTrials.gov and reported in accordance with CONSORT guidelines. We shall obtain informed consent from the all participants prior to enrolment into the study.

So as to minimize transmission of COVID-19 to the study participants and study team, a comprehensive risk management plan that will be followed in the study is highlighted in Appendix XXII The plan details how the infection prevention protocols will be followed in the study from the point the participants walk into the different study sites, through the consenting process, examination rooms to exiting the different health facilities where the study will be conducted. Measure like hand hygiene, appropriate use of personal protective equipment, waste management and how persons with suspected COVID-19 will be handled in the study are described in detail in the risk management plan.

3.6.1 Consenting process:

Women with first trimester incomplete abortion undergoing medical management, their spouses and healthcare providers will receive written and oral information about the study from the research team according to the principles of the Helsinki Declaration and have an opportunity to ask questions in a local language they are conversant in or English. If the eligible participant agrees to participate in the study, he or she will sign informed consent in the presence of the attending physician or research assistant prior enrolment into the study. This procedure will not differ between study sites.

Benefits and risks involved in the study will be communicated to the participants. All patients will receive the standard clinical care that is evidence based irrespective of whether they participate in the study or not. Those with medical conditions or emergencies like unsafe abortion, mental conditions or any other emergencies will be guided by the research team to the appropriate care teams at the different health facilities for best available medical care irrespective of whether they consent to participate in the study or not.

3.6.1.1 Participants' Autonomy: The participants will be availed all the information about the study processes. Women with first trimester incomplete abortions who opt for medical management will be recruited into the study after giving both an oral and written consent. The participants will be encouraged to ask questions in the language they feel so free to express themselves. Participants' confidentiality and privacy will be observed at all times during and after the study. Healthcare providers of various cadres offering family planning services especially in post abortion care will also be invited to participate in the study.

3.6.1.2 Beneficence: Participants in the study will receive a closer follow up for medical management of incomplete abortion as compared to those receiving routine clinical care. They will receive post abortion contraceptive counseling. The intervention will prevent unintended pregnancies, a benefit to the participants. As a public health contribution, this study might aid in formulation of a post abortion contraception policy after first trimester incomplete abortions that is currently missing. Participants will be compensated for their time and transport. Participants will be reassured that participating in the study is voluntary and that they can opt out of the study without it compromising the quality of care they will be receiving. Participants in need of other services like gender based violence will be referred to the responsible bodies. Those who develop complications like sepsis, marked hemorrhage, retained products of conception despite the recommended Misoprostol dosage will be managed surgically.

3.6.1.3 Justice: In this study all eligible participants will be recruited randomly irrespective of their ethnicity or socioeconomic status. We anticipate that the benefits post abortion contraception after first trimester incomplete abortions outweigh the harms that can arise from unintended pregnancies and the associated morbidies and mortality.

3.6.1.4 Non-maleficence: In our study, Misoprostol and the contraceptive devices (copper and Levonorgestrel releasing IUDs) are already registered by the National Drug Authority and readily available on the market. There's barely any harm using the products for their purposes in the study as they are within the National Clinical guidelines. There's minimal harm to the participants in this regard. We shall ensure participants' privacy and confidentiality at all times.

All participants' data (audiotapes, records, transcripts and notes) will be kept in a secure place that will be lockable and accessible only to personnel directly involved in the study. Study participants will not be identified by names in the final report.

3.7 Study limitations

- Different study sites will have variable patient caseloads however this will be addressed by using proportionate sampling.
- The number of Misoprostol doses to achieve complete abortion might affect the outcomes of the study as one would anticipate the more doses of Misoprostol, the more uterine contractions hence more expulsion of the IUDs. We shall keep track of all participants

especially those who might need more than one dose of Misoprostol to complete the expulsion of products of conception. This will enable us to assess whether the number of doses of Misoprostol has any influence on the expulsion rates. Participants will receive Non-steroidal Anti-inflammatory drugs like Ibuprofen which will inhibit prostaglandin effects on the endometrium that could lead to expulsion of the IUDs as pain management.

- Health Providers' individual attitude and experiences of recommending surgical over medical management of abortions could bias their recommendation of the study interventions. We shall conduct CMEs prior to the commencing the study in all the study sites.
- Misoprostol causes chills, rigors and fevers. Differentiating between Misoprostol side effects and sepsis might be a challenge but we shall carry out investigations to differentiate the two occurrences as post abortion sepsis is a contraindication for IUD insertion.

3.8 Dissemination of study findings:

Dissemination of the findings will be made as described below:

1) First to the women who will take part in the research together with their spouses, then to their local community through local media outlets.
2) Health care providers of the participating health facilities through dissemination meetings.
3) Ministry of health through presentation at the Maternal Child Health Technical Working Group to inform policy makers on required policy changes.
4) Publications in international scientific peer reviewed journals and presentation at both local and international conferences will further be used to disseminate the result within two years after completion of the study.

3.9 International and National research collaboration:

Professor Kristina Gemzell Danielsson, is head of the research group at the WHO-collaborating centre at the Department of Obstetrics and Gynaecology, Karolinska Institutet and Karolinska University Hospital and has extensive experience from conducting randomized controlled trials within Human Reproduction and implementing safe abortion and PAC worldwide. She is on the supervisory committee for the study and will use her expertise in ensuring the smooth running of the study.

Associate Professor Josaphat Byamugisha, MD and PhD from Makerere University and Karolinska Institutet, and previous head of the Department of Obstetrics and Gynaecology at Makerere University will be the principal supervisor in Uganda.

Associate Professor Nazarius Mbona Tumwesigye, PhD, lectures Biostatistics and Demography at the school of public health, Makerere University College of Health Sciences. His experience in Biostatistics will enrich the quality of write ups from the study findings. He will also have a supervisory role on this project.

Dr. Othman Kakaire, MD, PhD, senior lecturer and post doc at Makerere has solid experience from conducting randomised controlled trials in the Ugandan context. He will also be a supervisor on the project.

Dr. Herbert Kayiga, **MD, MPH and PhD scholar**, Makerere University will conduct this work for his PhD and will be the Principal Investigator.

Co-workers in Sweden and Uganda will be; **Amanda Cleeve**, **MSc, PhD**, Registered Nurse midwife and Postdoc researcher will use her expertise in the field contraception to enrich the study. **Emelie Looft- Ttrgardh,** is also a PhD student on the project, Karolinska Institutet.

Data analysis and publications will be performed in collaboration between researchers from Uganda and Sweden.

3.10 Timeline:

My goal is to complete the study within three years. I'll have presented the concept first in the Department of Obstetrics and Gynaecology. After my concept approval, I'm seeking to present the edited version of the proposal to the SOMREC for ethical clearance. With their ethic clearance, I'll seek the final ethical clearance from the Uganda National Scientific and Ethical Council. I anticipate that getting all these ethical clearances might take me about six months to one year. Data collection will be over one year. The outcomes will be measured up to one year. I plan to dedicate the six months for writing and submission for presentation and publication. Please see the table below summarizing the activities.

Given the volume of the abortions in the listed health facilities and my familiarity with the people and clinical setting of these health facilities, I believe this is a feasible goal. I plan to use the skill set and knowledge that I acquire from this research opportunity primarily in research to conduct future studies and to begin to apply for larger com(140)petitive funding.

4.0 WORK PLAN:

	May 2020	June 2021	July 2021	Aug 2021	Sept 2021	Oct 2021	Nov 2021	Dec 2021	Jan 2022	Feb 2022	Mar 2022	April 2022	May 2022	June 2022	July 2022	A 2
Obs Dept	X															
SOMREC						X	X									

IRB		X	X													
Uganda Regist								X	X							
Pretest Data Coll Tools											X					
Data Coll.											X	X	X	X	X	X
Data Analysis															X	X
Writing Manusc Prep.															X	X

5.0 REFERENCES:

References

1. Bearak J, Popinchalk A, Ganatra B, Moller A-B, Tunçalp Ö, Beavin C, et al. Unintended pregnancy and abortion by income, region, and the legal status of abortion: estimates from a comprehensive model for 1990–2019. The Lancet Global Health. 2020;8(9):e1152-e61.
2. Hubacher D, Mavranezouli I, McGinn E. Unintended pregnancy in sub-Saharan Africa: magnitude of the problem and potential role of contraceptive implants to alleviate it. Contraception. 2008;78(1):73-8.

3. Blumenthal P, Voedisch A, Gemzell-Danielsson K. Strategies to prevent unintended pregnancy: increasing use of long-acting reversible contraception. Human reproduction update. 2010;17(1):121-37.
4. Finer LB, Henshaw SK. Disparities in rates of unintended pregnancy in the United States, 1994 and 2001. Perspectives on sexual and reproductive health. 2006;38(2):90-6.
5. Sedgh G, Henshaw S, Singh S, Åhman E, Shah IH. Induced abortion: estimated rates and trends worldwide. The Lancet. 2007;370(9595):1338-45.
6. Sedgh G, Bearak J, Singh S, Bankole A, Popinchalk A, Ganatra B, et al. Abortion incidence between 1990 and 2014: global, regional, and subregional levels and trends. The Lancet. 2016;388(10041):258-67.
7. Ganatra B, Gerdts C, Rossier C, Johnson Jr BR, Tunçalp Ö, Assifi A, et al. Global, regional, and subregional classification of abortions by safety, 2010–14: estimates from a Bayesian hierarchical model. The Lancet. 2017;390(10110):2372-81.
8. Alfirevic Z, Milan SJ, Livio S. Caesarean section versus vaginal delivery for preterm birth in singletons. Cochrane Database of Systematic Reviews. 2012(6).
9. Ahmed S, Li Q, Liu L, Tsui AO. Maternal deaths averted by contraceptive use: an analysis of 172 countries. The Lancet. 2012;380(9837):111-25.
10. Ǻhman E, Shah IH. New estimates and trends regarding unsafe abortion mortality. International Journal of Gynecology & Obstetrics. 2011;115(2):121-6.
11. Min L, Singh K. Termination of Pregnancy: A Review of its Methodology and Post-Abortion Care. Obstet Gynecol Int J. 2017;6(5):000220.
12. Uganda Demographic Health Survey U. Health Survey 2011 Kampala, Uganda. Calverton, Maryland, USA: Uganda Bureau of Statistics (UBOS) and ICF International Inc. 2012.
13. Uganda MoH. State of Uganda Population Report 2018. 2018:96-101.
14. Uganda Demographic Survey 2016.
15. Vlassoff M, Sundaram A, Bankole A, Remez L, Mugisha F. Benefits of meeting the contraceptive needs of Ugandan women. Issues in brief (Alan Guttmacher Institute). 2009(4):1-8.
16. Andi JR, Wamala R, Ocaya B, Kabagenyi A. Modern contraceptive use among women in Uganda: An analysis of trend and patterns (1995-2011). Etude de la population africaine= African population studies. 2014;28(2):1009.
17. Singh S, Prada E, Mirembe F, Kiggundu C. The incidence of induced abortion in Uganda. International family planning perspectives. 2005:183-91.
18. Schreiber CA, Sober S, Ratcliffe S, Creinin MD. Ovulation resumption after medical abortion with mifepristone and misoprostol. Contraception. 2011;84(3):230-3.
19. Lähteenmäki P, Luukkainen T. Return of ovarian function after abortion. Clinical endocrinology. 1978;8(2):123-32.
20. Cameron I, Baird D. The return to ovulation following early abortion: a comparison between vacuum aspiration and prostaglandin. European Journal of Endocrinology. 1988;118(2):161-7.
21. Boesen HC, RØrbye C, NØrgaard M, Nilas L. Sexual behavior during the first eight weeks after legal termination of pregnancy. Acta obstetricia et gynecologica Scandinavica. 2004;83(12):1189-92.
22. Singh S, Prada E, Mirembe F, Kiggundu C. The incidence of induced abortion in Uganda. International family planning perspectives. 2005;31(4).
23. McNicholas C, Hotchkiss T, Madden T, Zhao Q, Allsworth J, Peipert JF. Immediate postabortion intrauterine device insertion: continuation and satisfaction. Women's Health Issues. 2012;22(4):e365-e9.
24. Gillett PG, Lee NH, Yuzpe AA, Cerskus I. A comparison of the efficacy and acceptability of the Copper-7 intrauterine device following immediate or delayed insertion after first-trimester therapeutic abortion. Fertility and sterility. 1980;34(2):121-4.

25. Stanek AM, Bednarek PH, Nichols MD, Jensen JT, Edelman AB. Barriers associated with the failure to return for intrauterine device insertion following first-trimester abortion. Contraception. 2009;79(3):216-20.
26. Davis A, Ramos ME, Rosario L, Westhoff C. Timing of copper intrauterine device insertion after medical abortion: a randomized controlled trial. Obstetrics & Gynecology. 2011;118(3):623-8.
27. Paul M, Iyengar SD, Essén B, Gemzell-Danielsson K, Iyengar K, Bring J, et al. Does mode of follow-up influence contraceptive use after medical abortion in a low-resource setting? Secondary outcome analysis of a non-inferiority randomized controlled trial. BMC public health. 2016;16(1):1-13.
28. Chong E, Frye LJ, Castle J, Dean G, Kuehl L, Winikoff B. A prospective, non-randomized study of home use of mifepristone for medical abortion in the US. Contraception. 2015;92(3):215-9.
29. Grossman D, Ellertson C, Grimes DA, Walker D. Routine follow-up visits after first-trimester induced abortion. Obstetrics & Gynecology. 2004;103(4):738-45.
30. Sääv I, Stephansson O, Gemzell-Danielsson K. Early versus delayed insertion of intrauterine contraception after medical abortion—a randomized controlled trial. PLoS One. 2012;7(11):e48948.
31. Mbonye AK, Asimwe J, Kabarangira J, Nanda G, Orinda V. Emergency obstetric care as the priority intervention to reduce maternal mortality in Uganda. International Journal of Gynecology & Obstetrics. 2007;96(3):220-5.
32. Benson J, Andersen K, Brahmi D, Healy J, Mark A, Ajode A, et al. What contraception do women use after abortion? An analysis of 319,385 cases from eight countries. Global public health. 2018;13(1):35-50.
33. Pereira IB, Carvalho RM, Graça LM. Intra-abortion contraception with etonogestrel subdermal implant. European Journal of Obstetrics & Gynecology and Reproductive Biology. 2015;185:33-5.
34. Fabic MS, Choi Y, Bongaarts J, Darroch JE, Ross JA, Stover J, et al. Meeting demand for family planning within a generation: the post-2015 agenda. The Lancet. 2015;385(9981):1928-31.
35. Kabiru CW, Ushie BA, Mutua MM, Izugbara CO. Previous induced abortion among young women seeking abortion-related care in Kenya: a cross-sectional analysis. BMC pregnancy and childbirth. 2016;16(1):1-10.
36. Maina BW, Mutua MM, Sidze EM. Factors associated with repeat induced abortion in Kenya. BMC Public Health. 2015;15(1):1-8.
37. Makenzius M, Oguttu M, Klingberg-Allvin M, Gemzell-Danielsson K, Odero TM, Faxelid E. Post-abortion care with misoprostol–equally effective, safe and accepted when administered by midwives compared to physicians: a randomised controlled equivalence trial in a low-resource setting in Kenya. BMJ open. 2017;7(10).
38. Cleeve A, Oguttu M, Ganatra B, Atuhairwe S, Larsson EC, Makenzius M, et al. Time to act—comprehensive abortion care in East Africa. The Lancet Global Health. 2016;4(9):e601-e2.
39. Aiken A, Lohr P, Aiken C, Forsyth T, Trussell J. Contraceptive method preferences and provision after termination of pregnancy: a population-based analysis of women obtaining care with the British Pregnancy Advisory Service. BJOG: An International Journal of Obstetrics & Gynaecology. 2017;124(5):815-24.
40. Organization WH, Health WHOR. Medical eligibility criteria for contraceptive use: World Health Organization; 2010.
41. Kalyanwala S, Acharya R, Francis Zavier A. Adoption and continuation of contraception following medical or surgical abortion in Bihar and Jharkhand, India. International Journal of Gynecology & Obstetrics. 2012;118:S47-S51.
42. Rocca CH, Puri M, Harper CC, Blum M, Dulal B, Henderson JT. Postabortion contraception a decade after legalization of abortion in Nepal. International Journal of Gynecology & Obstetrics. 2014;126(2):170-4.

43. Heikinheimo O, Gissler M, Suhonen S. Age, parity, history of abortion and contraceptive choices affect the risk of repeat abortion. Contraception. 2008;78(2):149-54.
44. Shaw D. The FIGO initiative for the prevention of unsafe abortion. International Journal of Gynecology & Obstetrics. 2010;110(Supplement):S17-S9.
45. Winner B, Peipert JF, Zhao Q, Buckel C, Madden T, Allsworth JE, et al. Effectiveness of long-acting reversible contraception. New England Journal of Medicine. 2012;366(21):1998-2007.
46. Cameron S, Glasier A, Chen Z, Johnstone A, Dunlop C, Heller R. Effect of contraception provided at termination of pregnancy and incidence of subsequent termination of pregnancy. BJOG: An International Journal of Obstetrics & Gynaecology. 2012;119(9):1074-80.
47. Pohjoranta E, Mentula M, Gissler M, Suhonen S, Heikinheimo O. Provision of intrauterine contraception in association with first trimester induced abortion reduces the need of repeat abortion: first-year results of a randomized controlled trial. Human Reproduction. 2015;30(11):2539-46.
48. Roberts H, Silva M, Xu S. Post abortion contraception and its effect on repeat abortions in Auckland, New Zealand. Contraception. 2010;82(3):260-5.
49. Trussell J. Contraceptive failure in the United States. Contraception. 2011;83(5):397-404.
50. Mondragón y Kalb M, Ortega AA, Velazquez JM, Olavarrieta CD, Rodríguez JV, Becker D, et al. Patient characteristics and service trends following abortion legalization in Mexico City, 2007–10. Studies in family planning. 2011;42(3):159-66.
51. Okusanya BO, Oduwole O, Effa EE. Immediate postabortal insertion of intrauterine devices. Cochrane Database of Systematic Reviews. 2014(7).
52. Timonen H, Luukkainen T. Immediate postabortion insertion of the copper-T (TCu-200) with eighteen months follow-up. Contraception. 1974;9(2):153-60.
53. Moussa A. Evaluation of postabortion IUD insertion in Egyptian women. Contraception. 2001;63(6):315-7.
54. Pyne S, Nagpal A, Barua A. CommonHealth is a coalition for reproductive health and safe Abortion. It is a membership based network of individuals and organisations across 22 states in India working in the area of maternal health, reproductive health and safe abortion access for women and communities.
55. FP2020. FP2020 Core Indicator Summary Sheet: 2019-2020 Annual Progress Report. 2020 24th September 2021.
56. Obstetricians RCo, Gynaecologists. The care of women requesting induced abortion: RCOG press; 2011.
57. Conde-Agudelo A, Belizan J, Breman R, Brockman S, Rosas-Bermudez A. Effect of the interpregnancy interval after an abortion on maternal and perinatal health in Latin America. International Journal of Gynecology & Obstetrics. 2005;89:S34-S40.
58. Adedini SA, Omisakin OA, Somefun OD. Trends, patterns and determinants of long-acting reversible methods of contraception among women in sub-Saharan Africa. PloS one. 2019;14(6):e0217574.
59. Steenland MW, Tepper NK, Curtis KM, Kapp N. Intrauterine contraceptive insertion postabortion: a systematic review. Contraception. 2011;84(5):447-64.
60. Bednarek PH, Creinin MD, Reeves MF, Cwiak C, Espey E, Jensen JT. Immediate versus delayed IUD insertion after uterine aspiration. New England Journal of Medicine. 2011;364(23):2208-17.
61. Grimes DA, Lopez LM, Schulz KF, Van Vliet HA, Stanwood NL. Immediate post-partum insertion of intrauterine devices. Cochrane Database of Systematic Reviews. 2010(5).
62. Pakarinen P, Toivonen J, Luukkainen T. Randomized comparison of levonorgestrel-and copper-releasing intrauterine systems immediately after abortion, with 5 years' follow-up. Contraception. 2003;68(1):31-4.

63. Suvisaari J, Lähteenmäki P. Detailed analysis of menstrual bleeding patterns after postmenstrual and postabortal insertion of a copper IUD or a levonorgestrel-releasing intrauterine system. Contraception. 1996;54(4):201-8.
64. Hasselback L, Dicko M, Viadro C, Ndour S, Ndao O, Wesson J. Understanding and addressing contraceptive stockouts to increase family planning access and uptake in Senegal. BMC health services research. 2017;17(1):1-8.
65. Ochako R, Izugbara C, Okal J, Askew I, Temmerman M. Contraceptive method choice among women in slum and non-slum communities in Nairobi, Kenya. BMC women's health. 2016;16(1):1-13.
66. Secura GM, Allsworth JE, Madden T, Mullersman JL, Peipert JF. The Contraceptive CHOICE Project: reducing barriers to long-acting reversible contraception. American journal of obstetrics and gynecology. 2010;203(2):115. e1-. e7.
67. Leonard AH, Winkler J. A quality of care framework for abortion care. Advances in Abortion Care. 1991;1(1):1-4.
68. Wolf M, Benson J. Meeting women's needs for post-abortion family planning: Report of a Bellagio Technical Working Group. International Journal of Gynecology & Obstetrics. 1994;45(Supplement):S3-S23.
69. Sääv I. Misoprostol as a family planning drug: use in pregnant and non-pregnant women: Inst för kvinnors och barns hälsa/Dept of Women's and Children's Health; 2014.
70. Say L, Chou D, Gemmill A, Tunçalp Ö, Moller A-B, Daniels J, et al. Global causes of maternal death: a WHO systematic analysis. The Lancet global health. 2014;2(6):e323-e33.
71. Ali M, Folz R, Farron M. Expanding choice and access in contraception: an assessment of intrauterine contraception policies in low and middle-income countries. BMC Public Health. 2019;19(1):1-6.
72. Uganda Bureau of Statistics I. Uganda demographic and health survey 2016: key indicators report. UBOS, and Rockville Maryland; 2017.
73. Tumlinson K, Pence BW, Curtis SL, Marshall SW, Speizer IS. Quality of care and contraceptive use in urban Kenya. International perspectives on sexual and reproductive health. 2015;41(2):69.
74. Santhya K. Changing family planning scenario in India: An overview of recent evidence. 2003.
75. Organization WH. Unsafe abortion incidence and mortality: global and regional levels in 2008 and trends during 1990-2008. World Health Organization; 2012.
76. Organization WH, Staff WHO, UNAIDS. Safe abortion: technical and policy guidance for health systems: World Health Organization; 2003.
77. Darroch JE, Singh S. Trends in contraceptive need and use in developing countries in 2003, 2008, and 2012: an analysis of national surveys. The Lancet. 2013;381(9879):1756-62.
78. Corbett MR, Turner KL. Essential elements of postabortion care: origins, evolution and future directions. International family planning perspectives. 2003;29(3):106-11.
79. United Nations DoEaSA, Population Division. . World population policies 2009. New York: United Nations; Report No. ST/ESA/SER.A/293). 2010. .
80. Organization WH. Safe abortion: technical and policy guidance for health systems: World Health Organization; 2012.
81. Pazol K, Creanga AA, Zane SB, Burley KD, Jamieson DJ. Abortion surveillance—United States, 2009. Morbidity and Mortality Weekly Report: Surveillance Summaries. 2012;61(8):1-44.
82. Hamoda H, Ashok PW, Flett GM, Templeton A. A randomised controlled trial of mifepristone in combination with misoprostol administered sublingually or vaginally for medical abortion up to 13 weeks of gestation. BJOG: An International Journal of Obstetrics & Gynaecology. 2005;112(8):1102-8.
83. Ho PC. Women's perceptions on medical abortion. Contraception. 2006;74(1):11-5.
84. Macnaughton H, Nothnagle M, Early J. Mifepristone and Misoprostol for Early Pregnancy Loss and Medication Abortion. American Family Physician. 2021;103(8):473-80.

85. Shannon C, Brothers LP, Philip NM, Winikoff B. Infection after medical abortion: a review of the literature. Contraception. 2004;70(3):183-90.
86. Bulletins—Gynecology P. Medication Abortion Up to 70 Days of Gestation. Contraception. 2020;102(4):225-36.
87. Kulier R, Kapp N, Gülmezoglu AM, Hofmeyr GJ, Cheng L, Campana A. Medical methods for first trimester abortion. Cochrane database of systematic reviews. 2004(1).
88. Wiebe E, Dunn S, Guilbert E, Jacot F, Lugtig L. Comparison of abortions induced by methotrexate or mifepristone followed by misoprostol. Obstetrics & Gynecology. 2002;99(5):813-9.
89. Von Hertzen H, Honkanen H, Piaggio G, Bartfai G, Erdenetungalag R, Gemzell-Danielsson K, et al. WHO multinational study of three misoprostol regimens after mifepristone for early medical abortion. I: Efficacy. BJOG: an international journal of obstetrics and gynaecology. 2003;110(9):808-18.
90. Spitz IM, Bardin CW, Benton L, Robbins A. Early pregnancy termination with mifepristone and misoprostol in the United States. New England Journal of Medicine. 1998;338(18):1241-7.
91. Morris JL, Winikoff B, Dabash R, Weeks A, Faundes A, Gemzell-Danielsson K, et al. FIGO's updated recommendations for misoprostol used alone in gynecology and obstetrics. 2017.
92. Creinin MD, Grossman DA. Medication Abortion Up to 70 Days of Gestation: ACOG Practice Bulletin, Number 225. Obstetrics & Gynecology. 2020;136(4):e31-e47.
93. Aubeny E, Peyron R, Turpin CL, Renault M, Targosz V, Silvestre L, et al. Termination of early pregnancy (up to 63 days of amenorrhea) with mifepristone and increasing doses of misoprostol [corrected]. International journal of fertility and menopausal studies. 1995;40:85-91.
94. Schaff EA, Eisinger SH, Stadalius LS, Franks P, Gore BZ, Poppema S. Low-dose mifepristone 200 mg and vaginal misoprostol for abortion. Contraception. 1999;59(1):1-6.
95. Winikoff B, Sivin I, Coyaji KJ, Cabezas E, Bilian X, Sujuan G, et al. Safety, efficacy, and acceptability of medical abortion in China, Cuba, and India: a comparative trial of mifepristone-misoprostol versus surgical abortion. American journal of obstetrics and gynecology. 1997;176(2):431-7.
96. Ashok P, Penney G, Flett G, Templeton A. An effective regimen for early medical abortion: a report of 2000 consecutive cases. Human reproduction (Oxford, England). 1998;13(10):2962-5.
97. Sawaya GF, Grady D, Kerlikowske K, Grimes DA. Antibiotics at the time of induced abortion: the case for universal prophylaxis based on a meta-analysis. Obstetrics & Gynecology. 1996;87(5):884-90.
98. Hemsell DL. Prophylactic antibiotics in gynecologic and obstetric surgery. Reviews of infectious diseases. 1991;13(Supplement_10):S821-S41.
99. Brewer C. Prevention of infection after abortion with a supervised single dose of oral doxycycline. British medical journal. 1980;281(6243):780.
100. Larsson P-G, Platz-Christensen J-J, Thejls H, Forsum U, Påhlson C. Incidence of pelvic inflammatory disease after first-trimester legal abortion in women with bacterial vaginosis after treatment with metronidazole: a double-blind, randomized study. American journal of obstetrics and gynecology. 1992;166(1):100-3.
101. Heisterberg L, Petersen K. Metronidazole prophylaxis in elective first trimester abortion. Obstetrics and gynecology. 1985;65(3):371-4.
102. Livshits A, Machtinger R, David LB, Spira M, Moshe-Zahav A, Seidman DS. Ibuprofen and paracetamol for pain relief during medical abortion: a double-blind randomized controlled study. Fertility and sterility. 2009;91(5):1877-80.
103. Creinin MD, Shulman T. Effect of nonsteroidal anti-inflammatory drugs on the action of misoprostol in a regimen for early abortion. Contraception. 1997;56(3):165-8.
104. Mahomed K, Healy J, Tandom S. Family planning counselling--a priority for post abortion care. The Central African journal of medicine. 1997;43(7):205-7.
105. McLaurin KE, Senanayake P. Post-abortion family planning: Reversing a legacy of neglect. The Lancet. 1993;342(8879):1099-100.

106. Sivin I, Tatum HJ. Four years of experience with the TCu 380A intrauterine contraceptive device. Fertility and sterility. 1981;36(2):159-63.
107. Regulation WHOTFoIDfF. IUD insertion following spontaneous abortion: a clinical trial of the TCu 220C, Lippes Loop D, and Copper 7. Studies in Family Planning. 1983:109-14.
108. Farley TMM RP, Meirik O, Rosenberg MJ, Chen JH.;. IUDs and pelvic inflammatory disease. Lancet. 1992;340(8813):248–9.
109. Sinei S, Schulz K, Lamptey P, Grimes D, Mati J, Rosenthal S, et al. Preventing IUCD-related pelvic infection: the efficacy of prophylactic doxycycline at insertion. BJOG: An International Journal of Obstetrics & Gynaecology. 1990;97(5):412-9.
110. Walsh T, Grimes D, Frezieres R, Nelson A, Bernstein L, Coulson A, et al. Randomised controlled trial of prophylactic antibiotics before insertion of intrauterine devices. The Lancet. 1998;351(9108):1005-8.
111. Gemzell-Danielsson K, Kallner HK. Post abortion contraception. Women's Health. 2015;11(6):779-84.
112. Goodman S, Hendlish SK, Reeves MF, Foster-Rosales A. Impact of immediate postabortal insertion of intrauterine contraception on repeat abortion. Contraception. 2008;78(2):143-8.
113. Burkman RT. New York UM-HC. Contraception & Family Planning. In: Decherney AH, Nathan L, Goodwin TM, Laufer N editor (s). CurrentDiagnosis&Treatment. Obstetrics&Gynecology 10th Edition 2007;579–597.
114. Solo J, Billings DL, Aloo-Obunga C, Ominde A, Makumi M. Creating linkages between incomplete abortion treatment and family planning services in Kenya. Studies in family planning. 1999;30(1):17-27.
115. Grimes DA, Mishell DR. Intrauterine contraception as an alternative to interval tubal sterilization. Contraception. 2008;77(1):6-9.
116. Prada E, Atuyambe LM, Blades NM, Bukenya JN, Orach CG, Bankole A. Incidence of Induced Abortion in Uganda, 2013: New Estimates Since 2003. PLoS ONE. 2016;11(11):e0165812.
117. Hussain R. Unintended pregnancy and abortion in Uganda. 2013.
118. Benson J, Andersen K, Healy J, Brahmi D. What factors contribute to postabortion contraceptive uptake by young women? A program evaluation in 10 countries in Asia and sub-Saharan Africa. Global Health: Science and Practice. 2017;5(4):644-57.
119. Niccolai LM, Hansen CE, Credle M, Shapiro ED. Parents' recall and reflections on experiences related to HPV vaccination for their children. Qualitative health research. 2016;26(6):842-50.
120. Rowe AK, Lama M, Onikpo F, Deming MS. Design effects and intraclass correlation coefficients from a health facility cluster survey in Benin. International Journal for Quality in Health Care. 2002;14(6):521-3.
121. Association GAotWM. World Medical Association Declaration of Helsinki: ethical principles for medical research involving human subjects. The Journal of the American College of Dentists. 2014;81(3):14.
122. Piaggio G, Elbourne DR, Pocock SJ, Evans SJ, Altman DG. Reporting of noninferiority and equivalence randomized trials: extension of the CONSORT 2010 statement. JAMA. 2012;308(24):2594-604.
123. Trussell J, Vaughan B. Contraceptive failure, method-related discontinuation and resumption of use: results from the 1995 National Survey of Family Growth. Family planning perspectives. 1999:64-93.
124. Suhonen S, Heikinheimo O, Tikka M, Haukkamaa M. The learning curve is rapid in medical termination of pregnancy—first-year results from the Helsinki area. Contraception. 2003;67(3):223-7.
125. Pohjoranta E, Suhonen S, Mentula M, Heikinheimo O. Intrauterine contraception after medical abortion: factors affecting success of early insertion. Contraception. 2017;95(3):257-62.

126. Reeves M, Fox M, Lohr P, Creinin MD. Endometrial thickness following medical abortion is not predictive of subsequent surgical intervention. Ultrasound in Obstetrics and Gynecology. 2009;34(1):104-9.
127. Rossi B, Creinin MD, Meyn LA. Ability of the clinician and patient to predict the outcome of mifepristone and misoprostol medical abortion. Contraception. 2004;70(4):313-7.
128. Creinin MD, Grossman DA. Medical management of first-trimester abortion. Contraception. 2014;89(3):148-61.
129. Korn E, Freidlin B. Interim monitoring for non-inferiority trials: minimizing patient exposure to inferior therapies. Annals of Oncology. 2018;29(3):573-7.
130. Chun Tie Y, Birks M, Francis K. Grounded theory research: A design framework for novice researchers. SAGE open medicine. 2019;7:2050312118822927.
131. Thomas DR. A general inductive approach for analyzing qualitative evaluation data. American journal of evaluation. 2006;27(2):237-46.
132. Jackson D, Daly J, Davidson P, Elliott D, Cameron-Traub E, Wade V, et al. Women recovering from first-time myocardial infarction (MI): a feminist qualitative study. Journal of advanced nursing. 2000;32(6):1403-11.
133. Patton MQ. Qualitative evaluation and research methods: SAGE Publications, inc; 1990.
134. Speziale HS, Streubert HJ, Carpenter DR. Qualitative research in nursing: Advancing the humanistic imperative: Lippincott Williams & Wilkins; 2011.
135. Crotty M, Crotty MF. The foundations of social research: Meaning and perspective in the research process: Sage; 1998.
136. Glaser BG. The constant comparative method of qualitative analysis. Social problems. 1965;12(4):436-45.
137. Lincoln YS, Guba EG. But is it rigorous? Trustworthiness and authenticity in naturalistic evaluation. New directions for program evaluation. 1986;1986(30):73-84.
138. Hsieh H-F, Shannon SE. Three approaches to qualitative content analysis. Qualitative health research. 2005;15(9):1277-88.
139. Palinkas LA, Horwitz SM, Green CA, Wisdom JP, Duan N, Hoagwood K. Purposeful sampling for qualitative data collection and analysis in mixed method implementation research. Administration and policy in mental health and mental health services research. 2015;42(5):533-44.
140. Singh S, Prada E, Mirembe F, Kiggundu CJIfpp. The incidence of induced abortion in Uganda. 2005:183-91.

APPENDIX I: BUDGET:

**Budget item**	**No of units**	**Unit cost (USD)**	**Frequency**	**Total (USD)**
**(A) Institutional Fees**				
1. Makerere University Fees	1	2740	3	8220

2. Makerere University College of Health Sciences Higher Degrees REC (IRB) fee	1	500	1	500
3. National Drug Authority fees	1	500	1	500
3. Uganda National Counsel for Science and Technology (UNCST) Fees	1	300	1	300
**Sub-total**				**9520**
**(B) Personnel**				
4. Research Assistants (30) – Quantitative	30	50	24	36000
Stipend for PI	1	3000	36	108,000
5. Data Clerk	2	2500	1	5000
**Sub-total**				**149000**
**(C) Qualitative Studies**				
Tape Recorder	1	800	1	800
Note taker and Transcription	1	1500	1	1500
Participant's token, snacks and transport costs	1	1000	1	1000
**Sub-total**				**3300**
**(D) Quantitative Studies**				**0**
Administrative costs and motivation for the 16 units		5500	1	5500
6. District entry meeting and identification of Research Assistants	1	2000	1	2000
7. Training data collectors	1	4000	1	4000
8. Training Health providers	1	10000	1	10000
9. Participants costs	1	10000	1	10000
10. Communication	1	2000	1	2000

11. Travel cost for follow up by PI and Coordinator	8	150	25	30000
12. Data Analysis	1	3000	1	3000
13. Study Coordinator	2	1000	30	60000
14. Data tool transportation	1	1200	1	1200
**Sub-total**				**119000**
**(E) Materials and consumables**				
15. Misoprostol	12000	0.5	1	6000
16. MVA Syringes	5	60	6	1800
17. Medical supplies	1	1000	1	1000
18. Computer	1	1500	1	1500
19. Printer	1	150	1	150
20. Scanner	1	100	1	100
21. Photocopier	1	250	1	250
22. Stationary	1	5000	1	5000
23. Protocol production	1	1000	1	1000
24. Filing Cabinets	4	200	1	800
**Sub-total**				**17,600**
**(F)Other Costs**				
25. Courses Attended	1	3000	4	12000
26. International Conferences	1	3000	2	6000
27. PhD Defense	1	4000	1	4000
Sub-total				22000
28. Administrative fees (15%)	1	46893	1	46893
**Total**				**360000**

APPENDIX II: BUDGET JUSTIFICATION:

1. Fees will be paid to Makerere University for Tuition, Registration, library and Examinations. I'll try to seek a tuition waiver from the University though.

2. A fee of $500 will be paid to the MakCHS Higher Degrees' Research and Ethics Committee for registration and yearly renewal of the protocol.

3. A fee of $300 will be paid to Uganda National Council of Science and Technology for the clearance of the research.

4. Thirty research assistants (two at each site) will be paid a nominal fee of 4 USD per questionnaire filled for each of the 5 visits.

5. Two data clerks will be needed to do double data entry. Each data entrant will spend one week per month to enter the data.

(C)Qualitative study costs cover: transport costs, payment for key informants, refreshments and professional fee for the social scientist

6. District entry meetings will be made for introduction and selection of Research Assistants.

7. Data collectors will be trained on how to collect data.

8. Providers will also be trained in both medical and surgical management of incomplete abortion.

9. Participants cost covers their transport refund per visit

10. Communication covers airtime for calling participants who haven't turned up on the scheduled dates as well as interaction between the PI, coordinator and RAs.

11. Travel costs for follow up cover vehicle hire and accommodation for the PI to monitor study activities

12. Covers costs for statistical analysis

13. Two study coordinators and two monitors will spend 100% time at USD1000 per month for 2 yrs.

14. Costs for transportation of filled questionnaires

15. Misoprostol will be purchased to treat the study patients with incomplete abortion.

16. MVA syringes will be used to manage participants that come back with incomplete abortion

17. Medical supplies include prescribed antibiotics, gloves, antiseptics, cotton, gauze, disinfectants and investigations (scan) that may not be available at the facility.

18-24. This cost will be used to prepare the research questionnaires and other study tools, securely store the data.

22. Stationary includes: reams of paper, log books, markers, punch machine, stapler, pens etc.

25. The cost to cover courses to attend will include payment for the course and travel.

26. This cost will be for attending at least 2 international conferences to share the research work.

27. PhD Defense costs cover transport, hotel hire and meals for the opponent.

28. Administrative fees in terms of incentives like tea will be issues, 3 USD
for every new patient recruited and USD

APPENDIX III: INFORMED CONSENT FOR RCT

MAKERERE UNIVERSITY COLLEGE OF HEALTH SCIENCES

SCHOOL OF MEDICINE RESEARCH AND ETHICS COMMITTEE (SOM-REC)

Title of the proposed study:

EFFECTIVENESS OF EARLY VERSUS STANDARD INTRAUTERINE CONTRACEPTION FOLLOWING PROVISION OF FIRST TRIMESTER MEDICAL POST ABORTION CARE IN CENTRAL UGANDA: A NON–INFERIORITY RANDOMIZED CLINICAL TRIAL

Investigator:

DR. HERBERT KAYIGA,
Department of Obstetrics and Gynecology,
Makerere University College of Health Services,
P.O. Box 7062, Kampala.
0777855063. Email: hkayiga@gmail.com

Co-Investigators:

ASSOC. PROF JOSAPHAT BYAMUGISHA,
Makerere University College of Health Sciences,
Department of Obs/Gyn,
P.O. Box 7062, Kampala.
0772580330. Email: jbyamugisha@gmail.com

DR. OTHMAN KAKAIRE
Makerere University College of Health Sciences,
Department of Obs/Gyn,
P.O. Box 7062, Kampala.
0772514616, Email: kakaireothman@gmail.com

ASSOC. PROF NAZARIUS .M. TUMWESIGYE
Makerere University College of Health Sciences,
Department of Epidemiology and Biostatistics
P.O. Box 7062, Kampala.
0782447771, Email: naz@musph.ac.ug

PROF KRISTINA GEMZELL-DANIELSSON
Karolinska Institutet and Karolinska University Hospital
Email: kristina.gemzell@ki.se

Background and rationale for the study:

This study seeks to determine the ideal time to have the intrauterine contraception inserted after medical management of an incomplete abortion within the first three months of conception in Central Uganda. Currently the practice has been to have intrauterine contraceptive (copper or Levonorgestrel releasing) devices inserted between 2-4 weeks after expulsion of the products of

conception. Unfortunately, many women rarely return to have the devices inserted for various reasons. This exposes them to unintended pregnancies as the ability to conceive returns as early as five to ten days after the pregnancies loss.

It is to this end that we request to interview you on the choice you have taken to use a uterine device as a means to prevent unwanted pregnancy after losing your previous pregnancy for which you were given Misoprostol tablets to put under your tongue to enable you expel the contents which hadn't come out fully. Your views will help us to know if inserting this method after your miscarriage is as safe and effective when put within one week of expelling the products of conception as when inserted 2-4 weeks later which has been the standard of care. We also seek to understand why you opted for the intrauterine device over the other methods of contraception and whether you would recommend this type of contraception to a friend in future. This will enable us to make evidence based recommendations on when to have post abortion intrauterine contraception after medical management of first trimester miscarriages.

A description of sponsors of the research project and the organizational affiliation of the researchers:

I am Dr. Herbert Kayiga, a practicing Obstetrician/Gynaecologist, also a Lecturer in the department of Obstetrics and Gynaecology, Makerere University College of Health Sciences, a researcher in the study "*Acceptability, Effectiveness and Uptake of Early versus Standard Post Abortion Intrauterine Contraception after First Trimester Medical Post Abortion Care for Incomplete Abortions in Central Uganda*". Associate Professor Josaphat Byamugisha, Associate Professor Nazarius Tumwesigye, and Dr. Othman Kakaire are also affiliated to Makerere University College of Health Sciences. They are my supervisors on this study project. Professor Kristina Gemzell-Danielsson, is co-investigator based at Karolinska Institutet and Karolinska University Hospital, Sweden.

This study will be conducted in 15 health facilities that will include Entebbe hospital, Mpigi HC IV, Masaka hospital, Gombe hospital, Nakaseke hospital, Luweero HC IV, Mengo hospital, Naguru hospital, Kawempe National referral hospital, Kawolo hospital, Wakiso HC IV, Kasangati HC IV, Kiganda Health centre IV and Kayunga hospital.

The study will be partly funded through collaboration between Makerere University and Karolinska Institutet and Karolinska University Hospital through Prof Kristina Gemzell-Danielsson. Makerere University has offered a tuition waiver while Karolinska Institutet, will offer some of the research funds.

Purpose:

In this study, we are investigating to know whether when you have an intrauterine device inserted in you within one week of using medical management (Misoprostol tablets) to complete the products of conception within three months of conception, is as effective as when inserted 2-4 weeks later which has been the standard of care. Your ability to conceive after a pregnancy loss within 12 weeks is high yet we don't want you to carry a pregnancy which is unwanted or unintended.

The estimated duration the research participant will take to in the research project:

We plan to take about 30 minutes in the questionnaire administered interviews. We shall require you to return for your follow up visits at 2 weeks, 3, 6, and 12 months.

How the device works:

You have a choice to make of the type of intrauterine device you would like to have inserted in you. The copper IUD can help you not to conceive for about ten years. You can however request any of the healthcare providers to help remove it at one time you would like to conceive or use any other method. This particular type of intrauterine device works by making the environment within the uterus unfavorable for conception by releasing some material called copper ions. The effect of the copper ions can make you to have heavy periods, more than the usual cramps during periods. Usually these symptoms can subside within six months. Though it can help you prevent conception 92% of the times, there's an eight percent chance of conception while using the method. The method works immediately following its insertion after medical management of first trimester incomplete abortions.

The Levonorgestrel intrauterine device can help you delay conception for five years once it is inserted. It works by releasing small amounts of a hormone Levonorgestrel that inhibits your ability to release an egg that can be fertilized. The method also makes your cervical mucus thick and in so doing, prevents sperms from swimming through. Though 99% of the times, you can't

conceive while using the method, there is one percent chance of conceiving while using the method.

Procedures:

We shall have you divided into two groups (one where the uterine device is inserted within one week) or (between 2-4 weeks) after medical management of incomplete abortion within the first three months of conception. By opting to participate in the study, we shall put you in any of the groups as will be determined by the research team. You might choose the type of intrauterine device to use either copper T or Levonorgestrel releasing IUDs but you might not choose on when to have it placed as per the requirements of this study.

After taking your medical history and having conducted a clinical examination on you, once found eligible to participate in the study, we shall insert the intrauterine device into your uterus. You will be made to lie facing up with your legs in stirrups. A speculum will be inserted into the vagina and an instrument (tenaculum) will be placed on your cervix to straighten the uterus. Another instrument (uterine sound) will be inserted when thought appropriate into your uterus to measure its length and thereafter the intrauterine device of your choice will be placed into the uterus. Threads will be cut at 3 centimeters length. You will asked to score on a vertical line on a VAS chart (10cm horizontal line) to indicate pain before insertion, at tenaculum and sound placement, IUC insertion, and before discharge.

On the follow up visits, we shall take your clinical history. We shall do a clinical examination which in addition to the general examiniation, will include a speculum examination to ascertain whether your intrauterine device is still in the right place. We shall ask you about the bleeding patterns since the insertion, any genital infections and your level of satisfaction with the intrauterine device of your choice.

Who will participate in the study?

You will be interviewed just like 2076 other participants to determine whether intrauterine contraception within one week is as effective as the standard insertion at 2-4 weeks after medical management of incomplete first trimester abortions.

Risks/Discomforts:

Both the copper T and Levonorgestrel releasing IUDs are already in use in Uganda for family planning. While using the intrauterine devices, the copper IUD, can make you have heavy or painful periods. There's a risk of bleeding in between your periods. These symptoms can however subside within six months or can be managed medically with drugs like Ibuprofen and other drugs. There's also an eight percent chance of conceiving while using the method. While using the Levonorgestrel IUD, there might be reduced menstrual flow or at times not seeing any menstrual loss. There's also a one percent risk of conceiving while using the method. Though the device works locally, sometimes the Levonorgestrel hormone can get into your circulation and this can alter your mood. None of the IUDs can prevent you from contracting HIV or any other sexually transmitted infections especially when you engage in unprotected sex.

In the event that you experience any of the following; severe infection following evacuation of the uterine contents, need for surgery (exploratory laparotomy) after post abortion intrauterine device insertions, need for blood transfusion following heavy (severe) hemorrhage, hospitalization over 72 hours, severe unusual reaction (anaphylactic reaction) after IUD insertion, or deaths for you or your spouse, these will be regarded as severe adverse events. Participants with adverse events will be managed immediately by the care teams at the different facilities as per the standard operating procedures. Those who need referral services will be given the best care at the expense of the research team. We shall request you to inform the Principal investigator or research team as soon as the events occur so that timely interventions can be undertaken.

Benefits:

Participants in the study will receive a closer follow up for their medical management of first trimester incomplete abortion as compared to the ones undertaking routine clinical care. They will receive post abortion contraceptive counseling. Using the copper or Levonorgestrel intrauterine devices enables you not to conceive for the duration of time you will use the devices. The Levonorgestrel intrauterine devices help you not to have heavy or painful periods. The copper intrauterine device helps you delay conception up to ten years while the Levonorgestrel intrauterine device helps you delay conception for up to five years while using the method. As a public health contribution, this study will aid in formulation of a post abortion contraception policy after first trimester abortions that is currently missing.

We anticipate that the benefits post abortion contraception after first trimester abortions outweigh the harms that can arise from unintended pregnancies and the associated complications.

The research team will take you through all the available family planning options, their efficacy, effectiveness and benefits of the options over the other methods. In the event that the choice you have taken as a contraceptive device is found to be inferior to the other device (copper or Levonorgrel) inserted after medical management of first trimester incomplete abortion, you will have the first priority to change the device without any financial costs to you

Confidentiality:

Patient data will be kept in password protected databases. All participants' data (audiotapes, records, transcripts and notes) will be kept in a secure place that will be lockable and accessible only to personnel directly involved in the study. Study participants will be identified by pseudonyms in the final report. The research team will sign confidentiality forms to ensure that they conform to keeping the participants' confidentiality at all times during and after the study.

As the primary investigator and the local Research Ethics Committee (REC) and Uganda National Council for Science and Technology (UNCST) will have access however to your private information that identifies the research participants by name to ensure that your safety is ensured throughout the study period.

Alternatives:

You have a right to decline to participate in the study or withdraw from it at any stage of questioning without it affecting the quality of care offered to you while you are in hospital. In case you opt for an alternative other than medical management of first trimester incomplete abortion, you will be given the alternative of surgical evacuation to remove the retained products of conception. Other family planning methods other than intrauterine contraception will also be availed to you for your informed decision on what is ideal for you.

Cost:

The costs of the medications like Misoprostol, antibiotics and pain killers during the medical management and the intrauterine devices will be covered by the study team. Any of the adverse effects that might occur to you during the study will be covered by the study team.

Compensation for participation in the study:

You will be given 30,000/= (Thirty thousand Uganda shillings) as a compensation for your time to participate in the study. In cases of any permanent damage or any major injuries during the study, the study participants will have their medical bills covered by the research team.

Reimbursement:

Study participants will be compensated for their time, and will have transport refund and will have their meals covered on their follow up visits. If you make it to the end of the study, we shall give you a token of appreciation on your last follow up visit at the discretion of the Principal investigators.

Questions about the study:

A readily available contact of a study nurse will be offered to all participants over 24 hours every week to address any issues that they will have with the study intervention or process. This will ensure timely response to the participants' concerns. The contact of the Principal investigator, School of Medicine and Ethics committee chairman and Secretary of the Uganda National Science and Technology committee are availed in this consent form.

Questions about participants rights:

If there are any questions you would like to ask in regards to the study procedure, follow up, any inquiries about the study materials or any concerns while in the study, you can contact Dr. Herbert Kayiga whose contact is listed below. In case you have concerns about the ethical conduct, any dissatisfaction with the study, please contact the chair of the Ethics review board or the National Council of Science and Technology secretary whose contacts are listed below. The contacts are:

1. **Dr. Herbert Kayiga**
   Department of Obstetrics and Gynecology,
   Makerere University College of Health Services,
   P.O. Box 7062, Kampala.
   0777855063.
   Email: hkayiga@gmail.com
2. **Prof Ponsiano Ocama**
   Chairperson:
   SOMREC (School of Medicine Research & Ethics Committee)
   0772421190
   Email: ponsiano.ocama@gmail.com
3. **Dr. Peter Ndemere**
   Executive Secretary- UNCST
   +256414705500

Email: info@uncst.go.ug

Statement of voluntariness:

You have a right to decline to participate in the study or withdraw from it at any stage of questioning without it affecting the quality of care offered to you while you are in hospital.

Dissemination of results:

You will be given feedback on the progress or the findings of the study. Any new information that affects the study or data that has clinical relevance to research participants (including incidental findings) will be made available to you and/or your health care providers.

Ethical approval:

Ethical approvals for this study have been obtained from SOMREC, UNCST, and the Ministry of Health of Uganda. Administrative permissions have also been sought from the directors of the different implementing health units and the department of Obstetrics and Gynaecology, Makerere University College of Health Sciences. The trials was also registered at ClinicalTrials.gov and reported in accordance with CONSORT guidelines.

STATEMENT OF CONSENT/ASSENT

.... has described to me what is going to be done, the risks, the benefits involved and my rights regarding this study. I understand that my decision to participate in this study will not alter my or my child's usual medical care. In the use of this information, my identity will be concealed. I am aware that I may withdraw at any time. I understand that by signing this form, I do not waive any of my legal rights but merely indicate that I have been informed about the research study in which I am voluntarily agreeing to participate. A copy of this form will be provided to me.

Name ………………………Signature/thumb print of participant …………………Date ……

Name ………………………Signature of parent/guardian for minors (If applicable)…Date ……..............................

Name………………………Signature of witness (if applicable)……………Date…………….

Name ……………………….Signature of interviewer/Person obtaining informed consent ……………………Date ………………….

APPENDIX IV: INFORMED ASSENT FOR RCT

MAKERERE UNIVERSITY COLLEGE OF HEALTH SCIENCES

SCHOOL OF MEDICINE RESEARCH AND ETHICS COMMITTEE (SOM-REC)

Title of the proposed study:

EFFECTIVENESS OF EARLY VERSUS STANDARD INTRAUTERINE CONTRACEPTION FOLLOWING PROVISION OF FIRST TRIMESTER MEDICAL POST ABORTION CARE IN CENTRAL UGANDA: A NON–INFERIORITY RANDOMIZED CLINICAL TRIAL

Investigator:
DR. HERBERT KAYIGA,
Department of Obstetrics and Gynecology,
Makerere University College of Health Services,
P.O. Box 7062, Kampala.
0777855063. Email: hkayiga@gmail.com
Co-Investigators:
ASSOC. PROF JOSAPHAT BYAMUGISHA,
Makerere University College of Health Sciences,
Department of Obs/Gyn,
P.O. Box 7062, Kampala.
0772580330. Email: jbyamugisha@gmail.com
DR. OTHMAN KAKAIRE
Makerere University College of Health Sciences,
Department of Obs/Gyn,
P.O. Box 7062, Kampala.
0772514616, Email: kakaireothman@gmail.com
ASSOC. PROF NAZARIUS .M. TUMWESIGYE
Makerere University College of Health Sciences,
Department of Epidemiology and Biostatistics
P.O. Box 7062, Kampala.
0782447771, Email: naz@musph.ac.ug
PROF KRISTINA GEMZELL-DANIELSSON
Karolinska Institutet and Karolinska University Hospital
Email: kristina.gemzell@ki.se

Background and rationale for the study:

This study seeks to determine the ideal time to have the intrauterine contraception inserted after medical management of an incomplete abortion within the first three months of conception in Central Uganda. Currently the practice has been to have intrauterine contraceptive (copper or Levonorgestrel releasing) devices inserted between 2-4 weeks after expulsion of the products of conception. Unfortunately, many women rarely return to have the devices inserted for various

reasons. This exposes them to unintended pregnancies as the ability to conceive returns as early as five to ten days after the pregnancies loss.

It is to this end that we request to interview you on the choice you have taken to use a uterine device as a means to prevent unwanted pregnancy after losing your previous pregnancy for which you were given Misoprostol tablets to put under your tongue to enable you expel the contents which hadn't come out fully. Your views will help us to know if inserting this method after your miscarriage is as safe and effective when put within one week of expelling the products of conception as when inserted 2-4 weeks later which has been the standard of care. We also seek to understand why you opted for the intrauterine device over the other methods of contraception and whether you would recommend this type of contraception to a friend in future. This will enable us to make evidence based recommendations on when to have post abortion intrauterine contraception after medical management of first trimester miscarriages.

A description of sponsors of the research project and the organizational affiliation of the researchers:

I am Dr. Herbert Kayiga, a practicing Obstetrician/Gynaecologist, also a Lecturer in the department of Obstetrics and Gynaecology, Makerere University College of Health Sciences, a researcher in the study "*Acceptability, Effectiveness and Uptake of Early versus Standard Post Abortion Intrauterine Contraception after First Trimester Medical Post Abortion Care for Incomplete Abortions in Central Uganda*". Associate Professor Josaphat Byamugisha, Associate Professor Nazarius Tumwesigye, and Dr. Othman Kakaire are also affiliated to Makerere University College of Health Sciences. They are my supervisors on this study project. Professor Kristina Gemzell-Danielsson, is co-investigator based at Karolinska Institutet and Karolinska University Hospital, Sweden.

This study will be conducted in 15 health facilities that will include Entebbe hospital, Mpigi HC IV, Masaka hospital, Gombe hospital, Nakaseke hospital, Luweero HC IV, Mengo hospital, Naguru hospital, Kawempe National referral hospital, Kawolo hospital, Wakiso HC IV, Kasangati HC IV, Kiganda Health centre IV and Kayunga hospital.

The study will be partly funded through collaboration between Makerere University and Karolinska Institutet and Karolinska University Hospital through Prof Kristina Gemzell-

Danielsson. Makerere University has offered a tuition waiver while Karolinska Institutet, will offer some of the research funds.

Purpose:

In this study, we are investigating to know whether when you have an intrauterine device inserted in you within one week of using medical management (Misoprostol tablets) to complete the products of conception within three months of conception, is as effective as when inserted 2-4 weeks later which has been the standard of care. Your ability to conceive after a pregnancy loss within 12 weeks is high yet we don't want you to carry a pregnancy which is unwanted or unintended.

The estimated duration the research participant will take to in the research project:

We plan to take about 30 minutes in the questionnaire administered interviews. We shall require you to return for your follow up visits at 2 weeks, 3, 6, and 12 months.

How the device works: You have a choice to make of the type of intrauterine device you would like to have inserted in you. The copper IUD can help you not to conceive for about ten years. You can however request any of the healthcare providers to help remove it at one time you would like to conceive or use any other method. This particular type of intrauterine device works by making the environment within the uterus unfavorable for conception by releasing some material called copper ions. The effect of the copper ions can make you to have heavy periods, more than the usual cramps during periods. Usually these symptoms can subside within six months. Though it can help you prevent conception 92% of the times, there's an eight percent chance of conception while using the method. The method works immediately following its insertion after medical management of first trimester incomplete abortions.

The Levonorgestrel intrauterine device can help you delay conception for five years once it is inserted. It works by releasing small amounts of a hormone Levonorgestrel that inhibits your ability to release an egg that can be fertilized. The method also makes your cervical mucus thick and in so doing, prevents sperms from swimming through. Though 99% of the times, you can't conceive while using the method, there is one percent chance of conceiving while using the method.

Procedures:

We shall have you divided into two groups (one where the uterine device is inserted within one week) or (between 2-4 weeks) after medical management of incomplete abortion within the first three months of conception. By opting to participate in the study, we shall put you in any of the groups as will be determined by the research team. You might choose the type of intrauterine device to use either copper T or Levonorgestrel releasing IUDs but you might not choose on when to have it placed as per the requirements of this study.

After taking your medical history and having coducted a clinical examination on you, once found eligible to participate in the study, we shall insert the intrauterine device into your uterus. You will be made to lie facing up with your legs in stirrups. A speculum will be inserted into the vagina and an instrument (tenaculum) will be placed on your cervix to straighten the uterus. Another instrument (uterine sound) will be inserted when thought appropriate into your uterus to measure its length and thereafter the intrauterine device of your choice will be placed into the uterus. Threads will be cut at 3 centimeters length. You will asked to score on a vertical line on a VAS chart (10cm horizontal line) to indicate pain before insertion, at tenaculum and sound placement, IUC insertion, and before discharge.

On the follow up visits, we shall take your clinical history. We shall do a clinical examination which in addition to the general examiniation, will include a speculum examination to ascertain whether your intrauterine device is still in the right place. We shall ask you about the bleeding patterns since the insertion, any genital infections and your level of satisfaction with the intrauterine device of your choice.

Who will participate in the study?

You will be interviewed just like 2082 other participants to determine whether intrauterine contraception within one week is as effective as the standard insertion at 2-4 weeks after medical management of incomplete first trimester abortions.

Risks/Discomforts: Both the copper T and Levonorgestrel releasing IUDs are already in use in Uganda for family planning. While using the intrauterine devices, the copper IUD, can make you have heavy or painful periods. There’s a risk of bleeding in between your periods. These symptoms can however subside within six months or can be managed medically with drugs like Ibuprofen and other drugs. There’s also an eight percent chance of conceiving while using the method. While

using the Levonorgestrel IUD, there might be reduced menstrual flow or at times not seeing any menstrual loss. There's also a one percent risk of conceiving while using the method. Though the device works locally, sometimes the Levonorgestrel hormone can get into your circulation and this can alter your mood. None of the IUDs can prevent you from contracting HIV or any other sexually transmitted infections especially when you engage in unprotected sex.

In the event that you experience any of the following; severe infection following evacuation of the uterine contents, need for surgery (exploratory laparotomy) after post abortion intrauterine device insertions, need for blood transfusion following heavy (severe) hemorrhage, hospitalization over 72 hours, severe unusual reaction (anaphylactic reaction) after IUD insertion, or deaths for you or your spouse, these will be regarded as severe adverse events. Participants with adverse events will be managed immediately by the care teams at the different facilities as per the standard operating procedures. Those who need referral services will be given the best care at the expense of the research team. We shall request you to inform the Principal investigator or research team as soon as the events occur so that timely interventions can be undertaken.

Benefits:

Participants in the study will receive a closer follow up for their medical management of first trimester incomplete abortion as compared to the ones undertaking routine clinical care. They will receive post abortion contraceptive counseling. Using the copper or Levonorgestrel intrauterine devices enables you not to conceive for the duration of time you will use the devices. The Levonorgestrel intrauterine devices help you not to have heavy or painful periods. The copper intrauterine device helps you delay conception up to ten years while the Levonorgestrel intrauterine device helps you delay conception for up to five years while using the method. As a public health contribution, this study will aid in formulation of a post abortion contraception policy after first trimester abortions that is currently missing.

We anticipate that the benefits post abortion contraception after first trimester abortions outweigh the harms that can arise from unintended pregnancies and the associated complications.

The research team will take you through all the available family planning options, their efficacy, effectiveness and benefits of the options over the other methods. In the event that the choice you have taken as a contraceptive device is found to be inferior to the other device (copper or Levonorgrel) inserted after medical management of first trimester incomplete abortion, you will have the first priority to change the device without any financial costs to you

Confidentiality:

Patient data will be kept in password protected databases. All participants' data (audiotapes, records, transcripts and notes) will be kept in a secure place that will be lockable and accessible only to personnel directly involved in the study. Study participants will be identified by pseudonyms in the final report. The research team will sign confidentiality forms to ensure that they conform to keeping the participants' confidentiality at all times during and after the study.

As the primary investigator and the local Research Ethics Committee (REC) and Uganda National Council for Science and Technology (UNCST) will have access however to your private information that identifies the research participants by name to ensure that your safety is ensured throughout the study period.

Alternatives:

You have a right to decline to participate in the study or withdraw from it at any stage of questioning without it affecting the quality of care offered to you while you are in hospital. In case you opt for an alternative other than medical management of first trimester incomplete abortion, you will be given the alternative of surgical evacuation to remove the retained products of conception. Other family planning methods other than intrauterine contraception will also be availed to you for your informed decision on what is ideal for you.

Cost:

The costs of the medications like Misoprostol, antibiotics and pain killers during the medical management and the intrauterine devices will be covered by the study team. Any of the adverse effects that might occur to you during the study will be covered by the study team.

Compensation for participation in the study:

You will be given 30,000/= (Thirty thousand Uganda shillings) as a compensation for your time to participate in the study. In cases of any permanent damage or any major injuries during the study, the study participants will have their medical bills covered by the research team.

Reimbursement:

Study participants will be compensated for their time, and will have transport refund and will have their meals covered on their follow up visits. If you make it to the end of the study, we shall give you a token of appreciation on your last follow up visit at the discretion of the Principal investigators.

Questions about the study:

A readily available contact of a study nurse will be offered to all participants over 24 hours every week to address any issues that they will have with the study intervention or process. This will ensure timely response to the participants' concerns. The contact of the Principal investigator, School of Medicine and Ethics committee chairman and Secretary of the Uganda National Science and Technology committee are availed in this consent form.

Questions about participants rights:

If there are any questions you would like to ask in regards to the study procedure, follow up, any inquiries about the study materials or any concerns while in the study, you can contact Dr. Herbert Kayiga whose contact is listed below. In case you have concerns about the ethical conduct, any dissatisfaction with the study, please contact the chair of the Ethics review board or the National Council of Science and Technology secretary whose contacts are listed below The contacts are:

1. **Dr. Herbert Kayiga**
   Department of Obstetrics and Gynecology,
   Makerere University College of Health Services,
   P.O. Box 7062, Kampala.
   0777855063.
   Email: hkayiga@gmail.com
2. **Prof Ponsiano Ocama**
   Chairperson:
   SOMREC (School of Medicine Research & Ethics Committee)
   0772421190
   Email: ponsiano.ocama@gmail.com
3. **Dr. Peter Ndemere**
   Executive Secretary- UNCST
   +256414705500
   Email: info@uncst.go.ug

Statement of voluntariness:

You have a right to decline to participate in the study or withdraw from it at any stage of questioning without it affecting the quality of care offered to you while you are in hospital.

Dissemination of results:

You will be given feedback on the progress or the findings of the study. Any new information that affects the study or data that has clinical relevance to research participants (including incidental findings) will be made available to you and/or your health care providers.

Ethical approval:

Ethical approvals for this study have been obtained from SOMREC, UNCST, and the Ministry of Health of Uganda. Administrative permissions have also been sought from the directors of the different implementing health units and the department of Obstetrics and Gynaecology, Makerere University College of Health Sciences. The trials was also registered at ClinicalTrials.gov and reported in accordance with CONSORT guidelines.

STATEMENT OF ASSENT

.... has described to me what is going to be done, the risks, the benefits involved and my rights regarding this study. I understand that my decision to participate in this study will not alter my or my child's usual medical care. In the use of this information, my identity will be concealed. I am aware that I may withdraw at any time. I understand that by signing this form, I do not waive any of my legal rights but merely indicate that I have been informed about the research study in which I am voluntarily agreeing to participate. A copy of this form will be provided to me.

Name ………………………Signature/thumb print of participant …………………Date ……

Name ………………………Signature of parent/guardian for minors (If applicable)…Date ……..............................

Name………………………Signature of witness (if applicable)……………Date…………….

Name ……………………….Signature of interviewer/Person obtaining informed consent ……………………Date ………………….

APPENDIX V: INFORMED CONSENT FOR UPTAKE OF POST ABORTION IUC

MAKERERE UNIVERSITY COLLEGE OF HEALTH SCIENCES

SCHOOL OF MEDICINE RESEARCH AND ETHICS COMMITTEE (SOM-REC)

Title of the proposed study:

UPTAKE AND FACTORS ASSOCIATED WITH UTILIZATION OF POST ABORTION INTRAUTERINE CONTRACEPTION AFTER FIRST TRIMESTER MEDICAL MANAGEMENT OF INCOMPLETE ABORTION IN CENTRAL UGANDA

Investigator:

DR. HERBERT KAYIGA,
Department of Obstetrics and Gynecology,
Makerere University College of Health Services,
P.O. Box 7062, Kampala.
0777855063. Email: hkayiga@gmail.com

Co-Investigators:

ASSOC. PROF JOSAPHAT BYAMUGISHA,
Makerere University College of Health Sciences,
Department of Obs/Gyn,
P.O. Box 7062, Kampala.
0772580330. Email: jbyamugisha@gmail.com

DR. OTHMAN KAKAIRE
Makerere University College of Health Sciences,
Department of Obs/Gyn,
P.O. Box 7062, Kampala.
0772514616, Email: kakaireothman@gmail.com

ASSOC. PROF NAZARIUS .M. TUMWESIGYE
Makerere University College of Health Sciences,
Department of Epidemiology and Biostatistics
P.O. Box 7062, Kampala.
0782447771, Email: naz@musph.ac.ug

PROF KRISTINA GEMZELL-DANIELSSON
Karolinska Institutet and Karolinska University Hospital
Email: kristina.gemzell@ki.se

Background and rationale for the study:

After first trimester miscarriages, the return of fertility is within two weeks. Unfortunately majority of the women never return to the health facilities for post abortion contraception thereafter. This leads to many of these women getting unintended and unwanted pregnancies thereafter that could have adverse pregnancy outcomes. In Uganda, post abortion contraception is widely available but the uptake has remained so low at 0.5%. The reasons for the low uptake and the drivers of this low contraception uptake are yet to be explored.

This study seeks to determine the proportion of women opting to take up post abortion intrauterine contraception out of the total population of women receiving post abortion care services over the study period at the 15 study centres. Such women will include those managed with Misoprostol tablets to complete the products of conception after a first trimester incomplete miscarriage. This study seeks also to identify the barriers and facilitators of the choice to take on post abortion intrauterine contraception to better inform policy makers on how to formulate context specific evidence based guidelines on post abortion contraception.

A description of sponsors of the research project and the organizational affiliation of the researchers:

I am Dr. Herbert Kayiga, a practicing Obstetrician/Gynaecologist, also a Lecturer in the department of Obstetrics and Gynaecology, Makerere University College of Health Sciences, a researcher in the main study "*Acceptability, Effectiveness and Uptake of Early versus Standard Post Abortion Intrauterine Contraception after First Trimester Medical Post Abortion Care for Incomplete Abortions in Uganda*". Associate Professor Josaphat Byamugisha, Associate Professor Nazarius Tumwesigye, and Dr. Othman Kakaire are also affiliated to Makerere University College of Health Sciences. They are my supervisors on this study project. Professor Kristina Gemzell-Danielsson, is co-investigator based at Karolinska Institutet and Karolinska University Hospital, Sweden.

This study will be conducted in Entebbe hospital, Mpigi HC IV, Masaka hospital, Gombe hospital, Nakaseke hospital, Luweero HC IV, Mengo hospital, Naguru hospital, Kawempe National referral hospital, Kawolo hospital, Wakiso HC IV, Kasangati HC IV, Kiganda Health Centre IV and Kayunga hospital.

The study will be partly funded through collaboration between Makerere University and Karolinska Institutet and Karolinska University Hospital through Prof Kristina Gemzell-Danielsson. Makerere University has offered a tuition waiver while Karolinska Institutet, will offer some of the research funds.

Purpose:

In this study, we are going to interview you on the choice you have taken to use a uterine device as a means to prevent unwanted pregnancy after losing your previous pregnancy for which you were given Misoprostol tablets to put under your tongue to enable you expel the contents which hadn’t come out fully. We also seek to understand why you opted for the intrauterine device over the other methods of contraception and whether you would recommend this type of contraception to a friend in future. This study also will seek to identify the barriers and facilitators of the choice to take on post abortion intrauterine contraception to better inform policy makers on how to formulate context specific evidence based guidelines on post abortion contraception.

The estimated duration the research participant will take to in the research project:

We plan to take about 30-45 minutes in the questionnaire administered interviews.

How the device works:

You have a choice to make of the type of intrauterine device you would like to have inserted in you. The copper IUD can help you not to conceive for about ten years. This particular type of intrauterine device works by making the environment within the uterus unfavorable for conception by releasing some material called copper ions. The effect of the copper ions can make you to have heavy periods, more than the usual cramps during periods. Usually these symptoms can subside within six months. The method works immediately following its insertion after medical management of first trimester incomplete abortions.

The Levonorgestrel intrauterine device can help you delay conception for five years once it is inserted. It works by releasing small amounts of a hormone Levonorgestrel that inhibits your ability to release an egg that can be fertilized. The method also makes your cervical mucus thick and in so doing, prevents sperms from swimming through.

Procedures:

You will be interviewed on the reasons behind your choice and whether you would recommend the method to your friends or even use the method again in future. We shall use the information you give us to identify the ideal time to insert the uterine devices after incomplete abortion/miscarriage within the first 12 weeks to prevent future unwanted and intended pregnancies. This is so because the ability to get another pregnancy is restored within two weeks after the expulsion of products of conception.

Who will participate in the study?

We plan to also interview 738 other women like you on the reasons behind your choice and whether you would recommend the method to your friends or even use the method again in future. We'll use the information you give us to understand barriers and facilitators of your choice to insert the uterine devices after pregnancy losses within the first 12 weeks to prevent future unwanted and intended pregnancies.

Risks/Discomforts: Both the copper T and Levonorgestrel releasing IUDs are already in use in Uganda for family planning. While using the intrauterine devices, the copper IUD, can make you have heavy or painful periods. There's a risk of bleeding in between your periods. These symptoms can however subside within six months or can be managed medically with drugs like Ibuprofen and other drugs. There's also an eight percent chance of conceiving while using the method. While using the Levonorgestrel IUD, there might be reduced menstrual flow or at times not seeing any menstrual loss. There's also a one percent risk of conceiving while using the method. Though the device works locally, sometimes the Levonorgestrel hormone can get into your circulation and this can alter your mood, cause breast tenderness. None of the IUDs can prevent you from contracting HIV or any other sexually transmitted infections especially when you engage in unprotected sex.

In the event that you experience any of the following; severe infection following evacuation of the uterine contents, need for surgery (exploratory laparotomy) after post abortion intrauterine device insertions, need for blood transfusion following heavy (severe) hemorrhage, hospitalization over 72 hours, severe unusual reaction (anaphylactic reaction) after IUD insertion, or deaths for you or your spouse, these will be regarded as severe adverse events. Participants with adverse events will be managed immediately by the care teams at the different facilities as per the standard operating procedures. Those who need referral services will be given the best care at the expense of the

research team. We shall request you to inform the Principal investigator or research team as soon as the events occur so that timely interventions can be undertaken.

Benefits:

Participants in the study will receive a closer follow up for their medical management of first trimester incomplete abortion as compared to the ones undertaking routine clinical care. They will receive post abortion contraceptive counseling. The intervention will prevent unintended pregnancies a benefit to the participants. As a public health contribution, this study will aid in formulation of a post abortion contraception policy after first trimester abortions that is currently missing.

We anticipate that the benefits post abortion contraception after first trimester abortions outweigh the harms that can arise from unintended pregnancies and the associated complications.

The research team will take you through all the available family planning options, their efficacy, effectiveness and benefits of the options over the other methods. In the event that the choice you have taken as a contraceptive device is found to be inferior to the other device (copper or Levonorgrel) inserted after medical management of first trimester incomplete abortion, you will have the first priority to change the device without any financial costs to you

Confidentiality:

Patient data will be kept in password protected databases. All participants' data (audiotapes, records, transcripts and notes) will be kept in a secure place that will be lockable and accessible only to personnel directly involved in the study. Study participants will be identified by pseudonyms in the final report. The research team will sign confidentiality forms to ensure that they conform to keeping the participants' confidentiality at all times during and after the study.

As the primary investigator and the local Research Ethics Committee (REC) and Uganda National Council for Science and Technology (UNCST) will have access however to your private information that identifies the research participants by name to ensure that your safety is ensured throughout the study period.

Alternatives:

You have a right to decline to participate in the study or withdraw from it at any stage of questioning without it affecting the quality of care offered to you while you are in hospital. In case you opt for an alternative other than medical management of first trimester incomplete abortion,

you will be given the alternative of surgical evacuation to remove the retained products of conception. Other family planning methods other than intrauterine contraception will also be availed to you for your informed decision on what is ideal for you.

Cost:

The costs of the medications like Misoprostol, antibiotics and pain killers during the medical management and the intrauterine devices will be covered by the study team. Any of the adverse effects that might occur to you during the study will be covered by the study team.

Compensation for participation in the study:

You will be given 30,000/= (Thirty thousand Uganda shillings) as a compensation for your time to participate in the study. In cases of any permanent damage or any major injuries during the study, the study participants will have their medical bills covered by the research team.

Reimbursement:

Study participants will be compensated for their time, and will have transport refund.

Questions about the study:

A readily available contact of a study nurse will be offered to all participants over 24 hours every week to address any issues that they will have with the study intervention or process. This will ensure timely response to the participants' concerns. The contact of the Principal investigator, School of Medicine and Ethics committee chairman and Secretary of the Uganda National Science and Technology committee are availed in this consent form.

Questions about participants rights:

If there are any questions you would like to ask in regards to the study procedure, any inquiries about the study materials or any concerns while in the study, you can contact Dr. Herbert Kayiga whose contact is listed below. In case you have concerns about the ethical conduct, any dissatisfaction with the study, please contact the chair of the Ethics review board or the National Council of Science and Technology secretary whose contacts are listed below

The contacts are:

1. **Dr. Herbert Kayiga**
   Department of Obstetrics and Gynecology,
   Makerere University College of Health Services,
   P.O. Box 7062, Kampala.
   0777855063.
   Email: hkayiga@gmail.com

2. **Prof Ponsiano Ocama**
Chairperson:
SOMREC (School of Medicine Research & Ethics Committee)
0772421190
Email: ponsiano.ocama@gmail.com
3. **Dr. Peter Ndemere**
Executive Secretary- UNCST
+256414705500
Email: info@uncst.go.ug

Statement of voluntariness:

You have a right to decline to participate in the study or withdraw from it at any stage of questioning without it affecting the quality of care offered to you while you are in hospital.

Dissemination of results:

You will be given feedback on the progress or the findings of the study. Any new information that affects the study or data that has clinical relevance to research participants (including incidental findings) will be made available to you and/or your health care providers.

Ethical approval: Ethical approvals for this study have been obtained from School of Medicine Research and Ethics Committee, Uganda National Council of Science and Technology, and the Ministry of Health of Uganda. Administrative permissions have also been sought from the directors of the different implementing health units..

STATEMENT OF CONSENT/ASSENT

.... has described to me what is going to be done, the risks, the benefits involved and my rights regarding this study. I understand that my decision to participate in this study will not alter my usual medical care. In the use of this information, my identity will be concealed. I am aware that I may withdraw at any time. I understand that by signing this form, I do not waive any of my legal rights but merely indicate that I have been informed about the research study in which I am voluntarily agreeing to participate. A copy of this form will be provided to me.

Name ………………………Signature/thumb print of participant …………………Date ……

Name ………………………Signature of parent/guardian for minors (If applicable)…Date ……...............................

Name……………………Signature of witness (if applicable)……………Date…………….

Name ……………………….Signature of interviewer/Person obtaining informed consent ……………………Date ………………….

APPENDIX VI: INFORMED ASSENT FOR UPTAKE OF POST ABORTION IUC

MAKERERE UNIVERSITY COLLEGE OF HEALTH SCIENCES

SCHOOL OF MEDICINE RESEARCH AND ETHICS COMMITTEE (SOM-REC)

Title of the proposed study:

UPTAKE AND FACTORS ASSOCIATED WITH UTILIZATION OF POST ABORTION INTRAUTERINE CONTRACEPTION AFTER FIRST TRIMESTER MEDICAL MANAGEMENT OF INCOMPLETE ABORTION IN CENTRAL UGANDA

Investigator:

DR. HERBERT KAYIGA,
Department of Obstetrics and Gynecology,
Makerere University College of Health Services,
P.O. Box 7062, Kampala.
0777855063. Email: hkayiga@gmail.com

Co-Investigators:

ASSOC. PROF JOSAPHAT BYAMUGISHA,
Makerere University College of Health Sciences,
Department of Obs/Gyn,
P.O. Box 7062, Kampala.
0772580330. Email: jbyamugisha@gmail.com

DR. OTHMAN KAKAIRE
Makerere University College of Health Sciences,
Department of Obs/Gyn,
P.O. Box 7062, Kampala.
0772514616, Email: kakaireothman@gmail.com

ASSOC. PROF NAZARIUS .M. TUMWESIGYE
Makerere University College of Health Sciences,
Department of Epidemiology and Biostatistics
P.O. Box 7062, Kampala.
0782447771, Email: naz@musph.ac.ug

PROF KRISTINA GEMZELL-DANIELSSON
Karolinska Institutet and Karolinska University Hospital
Email: kristina.gemzell@ki.se

Background and rationale for the study:

After first trimester miscarriages, the return of fertility is within two weeks. Unfortunately majority of the women never return to the health facilities for post abortion contraception thereafter. This leads to many of these women getting unintended and unwanted pregnancies thereafter that could have adverse pregnancy outcomes. In Uganda, post abortion contraception is widely available but the uptake has remained so low at 0.5%. The reasons for the low uptake and the drivers of this low contraception uptake are yet to be explored.

This study seeks to determine the proportion of women opting to take up post abortion intrauterine contraception out of the total population of women receiving post abortion care services over the study period at the 15 study centres. Such women will include those managed with Misoprostol tablets to complete the products of conception after a first trimester incomplete miscarriage. This study seeks also to identify the barriers and facilitators of the choice to take on post abortion intrauterine contraception to better inform policy makers on how to formulate context specific evidence based guidelines on post abortion contraception.

A description of sponsors of the research project and the organizational affiliation of the researchers:

I am Dr. Herbert Kayiga, a practicing Obstetrician/Gynaecologist, also a Lecturer in the department of Obstetrics and Gynaecology, Makerere University College of Health Sciences, a researcher in the main study "*Acceptability, Effectiveness and Uptake of Early versus Standard Post Abortion Intrauterine Contraception after First Trimester Medical Post Abortion Care for Incomplete Abortions in Uganda*". Associate Professor Josaphat Byamugisha, Associate Professor Nazarius Tumwesigye, and Dr. Othman Kakaire are also affiliated to Makerere University College of Health Sciences. They are my supervisors on this study project. Professor Kristina Gemzell-Danielsson, is co-investigator based at Karolinska Institutet and Karolinska University Hospital, Sweden.

This study will be conducted in Entebbe hospital, Mpigi HC IV, Masaka hospital, Gombe hospital, Nakaseke hospital, Luweero HC IV, Mengo hospital,, Naguru hospital, Kawempe National referral hospital, Kawolo hospital, Wakiso HC IV, Kasangati HC IV, Kiganda Health Centre IV and Kayunga hospital.

The study will be partly funded through collaboration between Makerere University and Karolinska Institutet and Karolinska University Hospital through Prof Kristina Gemzell-Danielsson. Makerere University has offered a tuition waiver while Karolinska Institutet, will offer some of the research funds.

Purpose:

In this study, we are going to interview you on the choice you have taken to use a uterine device as a means to prevent unwanted pregnancy after losing your previous pregnancy for which you were given Misoprostol tablets to put under your tongue to enable you expel the contents which hadn't come out fully. We also seek to understand why you opted for the intrauterine device over the other methods of contraception and whether you would recommend this type of contraception to a friend in future. This study also will seek to identify the barriers and facilitators of the choice to take on post abortion intrauterine contraception to better inform policy makers on how to formulate context specific evidence based guidelines on post abortion contraception.

The estimated duration the research participant will take to in the research project:

We plan to take about 30-45 minutes in the questionnaire administered interviews.

How the device works:

You have a choice to make of the type of intrauterine device you would like to have inserted in you. The copper IUD can help you not to conceive for about ten years. This particular type of intrauterine device works by making the environment within the uterus unfavorable for conception by releasing some material called copper ions. The effect of the copper ions can make you to have heavy periods, more than the usual cramps during periods. Usually these symptoms can subside within six months. The method works immediately following its insertion after medical management of first trimester incomplete abortions.

The Levonorgestrel intrauterine device can help you delay conception for five years once it is inserted. It works by releasing small amounts of a hormone Levonorgestrel that inhibits your ability to release an egg that can be fertilized. The method also makes your cervical mucus thick and in so doing, prevents sperms from swimming through.

Procedures:

You will be interviewed on the reasons behind your choice and whether you would recommend the method to your friends or even use the method again in future. We shall use the information you give us to identify the ideal time to insert the uterine devices after incomplete abortion/miscarriage within the first 12 weeks to prevent future unwanted and intended pregnancies. This is so because the ability to get another pregnancy is restored within two weeks after the expulsion of products of conception.

Who will participate in the study?

We plan to also interview 900 other women like you on the reasons behind your choice and whether you would recommend the method to your friends or even use the method again in future. We'll use the information you give us to understand barriers and facilitators of your choice to insert the uterine devices after pregnancy losses within the first 12 weeks to prevent future unwanted and intended pregnancies.

Risks/Discomforts: Both the copper T and Levonorgestrel releasing IUDs are already in use in Uganda for family planning. While using the intrauterine devices, the copper IUD, can make you have heavy or painful periods. There's a risk of bleeding in between your periods. These symptoms can however subside within six months or can be managed medically with drugs like Ibuprofen and other drugs. There's also an eight percent chance of conceiving while using the method. While using the Levonorgestrel IUD, there might be reduced menstrual flow or at times not seeing any menstrual loss. There's also a one percent risk of conceiving while using the method. Though the device works locally, sometimes the Levonorgestrel hormone can get into your circulation and this can alter your mood, cause breast tenderness. None of the IUDs can prevent you from contracting HIV or any other sexually transmitted infections especially when you engage in unprotected sex.

In the event that you experience any of the following; severe infection following evacuation of the uterine contents, need for surgery (exploratory laparotomy) after post abortion intrauterine device insertions, need for blood transfusion following heavy (severe) hemorrhage, hospitalization over 72 hours, severe unusual reaction (anaphylactic reaction) after IUD insertion, or deaths for you or your spouse, these will be regarded as severe adverse events. Participants with adverse events will be managed immediately by the care teams at the different facilities as per the standard operating procedures. Those who need referral services will be given the best care at the expense of the

research team. We shall request you to inform the Principal investigator or research team as soon as the events occur so that timely interventions can be undertaken.

Benefits:

Participants in the study will receive a closer follow up for their medical management of first trimester incomplete abortion as compared to the ones undertaking routine clinical care. They will receive post abortion contraceptive counseling. The intervention will prevent unintended pregnancies a benefit to the participants. As a public health contribution, this study will aid in formulation of a post abortion contraception policy after first trimester abortions that is currently missing.

We anticipate that the benefits post abortion contraception after first trimester abortions outweigh the harms that can arise from unintended pregnancies and the associated complications.

The research team will take you through all the available family planning options, their efficacy, effectiveness and benefits of the options over the other methods. In the event that the choice you have taken as a contraceptive device is found to be inferior to the other device (copper or Levonorgrel) inserted after medical management of first trimester incomplete abortion, you will have the first priority to change the device without any financial costs to you.

Confidentiality:

Patient data will be kept in password protected databases. All participants' data (audiotapes, records, transcripts and notes) will be kept in a secure place that will be lockable and accessible only to personnel directly involved in the study. Study participants will be identified by pseudonyms in the final report. The research team will sign confidentiality forms to ensure that they conform to keeping the participants' confidentiality at all times during and after the study.

As the primary investigator and the local Research Ethics Committee (REC) and Uganda National Council for Science and Technology (UNCST) will have access however to your private information that identifies the research participants by name to ensure that your safety is ensured throughout the study period.

Alternatives:

You have a right to decline to participate in the study or withdraw from it at any stage of questioning without it affecting the quality of care offered to you while you are in hospital. In case you opt for an alternative other than medical management of first trimester incomplete abortion,

you will be given the alternative of surgical evacuation to remove the retained products of conception. Other family planning methods other than intrauterine contraception will also be availed to you for your informed decision on what is ideal for you.

Cost:

The costs of the medications like Misoprostol, antibiotics and pain killers during the medical management and the intrauterine devices will be covered by the study team. Any of the adverse effects that might occur to you during the study will be covered by the study team.

Compensation for participation in the study:

You will be given 30,000/= (Thirty thousand Uganda shillings) as a compensation for your time to participate in the study. In cases of any permanent damage or any major injuries during the study, the study participants will have their medical bills covered by the research team.

Reimbursement:

Study participants will be compensated for their time, and will have transport refund.

Questions about the study:

A readily available contact of a study nurse will be offered to all participants over 24 hours every week to address any issues that they will have with the study intervention or process. This will ensure timely response to the participants' concerns. The contact of the Principal investigator, School of Medicine and Ethics committee chairman and Secretary of the Uganda National Science and Technology committee are availed in this consent form.

Questions about participants rights:

If there are any questions you would like to ask in regards to the study procedure, any inquiries about the study materials or any concerns while in the study, you can contact Dr. Herbert Kayiga whose contact is listed below. In case you have concerns about the ethical conduct, any dissatisfaction with the study, please contact the chair of the Ethics review board or the National Council of Science and Technology secretary whose contacts are listed below

The contacts are:

1. **Dr. Herbert Kayiga**
   Department of Obstetrics and Gynecology,
   Makerere University College of Health Services,
   P.O. Box 7062, Kampala.
   0777855063.
   Email: hkayiga@gmail.com

2. **Prof Ponsiano Ocama**
Chairperson:
SOMREC (School of Medicine Research & Ethics Committee)
0772421190
Email: ponsiano.ocama@gmail.com
3. **Dr. Peter Ndemere**
Executive Secretary- UNCST
+256414705500
Email: info@uncst.go.ug

Statement of voluntariness:

You have a right to decline to participate in the study or withdraw from it at any stage of questioning without it affecting the quality of care offered to you while you are in hospital.

Dissemination of results:

You will be given feedback on the progress or the findings of the study. Any new information that affects the study or data that has clinical relevance to research participants (including incidental findings) will be made available to you and/or your health care providers.

Ethical approval:

Ethical approvals for this study have been obtained from SOMREC, UNCST, and the Ministry of Health of Uganda. Administrative permissions have also been sought from the directors of the different implementing health units..

STATEMENT OF ASSENT

.... has described to me what is going to be done, the risks, the benefits involved and my rights regarding this study. I understand that my decision to participate in this study will not alter my usual medical care. In the use of this information, my identity will be concealed. I am aware that I may withdraw at any time. I understand that by signing this form, I do not waive any of my legal rights but merely indicate that I have been informed about the research study in which I am voluntarily agreeing to participate. A copy of this form will be provided to me.

Name ………………………Signature/thumb print of participant …………………Date ……

Name ………………………Signature of parent/guardian for minors (If applicable)…Date ……...............................

Name………………………Signature of witness (if applicable)……………Date…………….

Name ……………………….Signature of interviewer/Person obtaining informed consent ……………………Date ………………….

APPENDIX VII: HEALTH WORKERS' CONSENT FOR INTERVIEW
MAKERERE UNIVERSITY COLLEGE OF HEALTH SCIENCES

SCHOOL OF MEDICINE RESEARCH AND ETHICS COMMITTEE (SOM-REC)

Title of the proposed study:

"HEALTHCARE PROVIDERS' PERCEPTIONS TOWARDS POST ABORTION INTRAUTERINE CONTRACEPTION AFTER FIRST TRIMESTER MEDICAL MANAGEMENT OF INCOMPLETE ABORTION IN UGANDA: A QUALITATIVE STUDY"

Investigator:

DR. HERBERT KAYIGA,
Department of Obstetrics and Gynecology,
Makerere University College of Health Services,
P.O. Box 7062, Kampala.
0777855063. Email: hkayiga@gmail.com

Co-Investigators:

ASSOC. PROF JOSAPHAT BYAMUGISHA,
Makerere University College of Health Sciences,
Department of Obs/Gyn,
P.O. Box 7062, Kampala.
0772580330. Email: jbyamugisha@gmail.com

DR. OTHMAN KAKAIRE
Makerere University College of Health Sciences,
Department of Obs/Gyn,
P.O. Box 7062, Kampala.
0772514616, Email: kakaireothman@gmail.com

ASSOC. PROF NAZARIUS .M. TUMWESIGYE
Makerere University College of Health Sciences,
Department of Epidemiology and Biostatistics
P.O. Box 7062, Kampala.
0782447771, Email: naz@musph.ac.ug

PROF KRISTINA GEMZELL-DANIELSSON
Karolinska Institutet and Karolinska University Hospital
Email: kristina.gemzell@ki.se

Background and rationale for the study:

This study seeks to determine the ideal time to have the intrauterine contraception inserted after medical management of an incomplete abortion within the first three months of conception in Central Uganda. Currently the practice has been to have intrauterine contraceptive (copper or

Levonorgestrel releasing) devices inserted between 2-4 weeks after expulsion of the products of conception. Unfortunately, many women rarely return to have the devices inserted for various reasons. This exposes them to unintended pregnancies as the ability to conceive returns as early as five to ten days after the pregnancies loss.

It is to this end that we request to interview you on your perception as a healthcare provider towards placement of uterine devices as a means to prevent unwanted pregnancies after medical management of first trimester incomplete abortion. Your views will help us in formulation of evidence based guidelines in regards to post abortion contraception and determining the ideal time for placement of intrauterine devices after medical management of first trimester incomplete abortions. We also seek to understand the barriers and facilitators among healthcare providers towards recommendation of post abortion contraception especially the intrauterine devices.

A description of sponsors of the research project and the organizational affiliation of the researchers:

I am Dr. Herbert Kayiga, a practicing Obstetrician/Gynaecologist, also a Lecturer in the department of Obstetrics and Gynaecology, Makerere University College of Health Sciences, a researcher in the main study "*Acceptability, Effectiveness and Uptake of Early versus Standard Post Abortion Intrauterine Contraception after First Trimester Medical Post Abortion Care for Incomplete Abortions in Central Uganda*". Associate Professor Josaphat Byamugisha, Associate Professor Nazarius Tumwesigye, and Dr. Othman Kakaire are also affiliated to Makerere University College of Health Sciences. They are my supervisors on this study project. Professor Kristina Gemzell-Danielsson, is co-investigator based at Karolinska Institutet and Karolinska University Hospital, Sweden.

This study will be conducted in Entebbe hospital, Mpigi HC IV, Masaka hospital, Gombe hospital, Nakaseke hospital, Luweero HC IV, Mengo hospital, Naguru hospital, Kawempe National referral hospital, Kawolo hospital, Wakiso HC IV, Kasangati HC IV, Kiganda Health Centre IV and Kayunga hospital.

The study will be partly funded through collaboration between Makerere University and Karolinska Institutet and Karolinska University Hospital through Prof Kristina Gemzell-

Danielsson. Makerere University has offered a tuition waiver while Karolinska Institutet, will offer some of the research funds.

Purpose:

In this study, we are going to interview you on your perception towards post abortion contraception after first trimester medical management of incomplete abortion. Your views will help us know whether early insertion (within one week) is as effective as standard insertion (between 2-4 weeks after first trimester medical management of incomplete abortion). If proven to be as effective, then we would prevent unwanted pregnancies among women with first trimester pregnancy losses as the return to fertility occurs within two weeks. Your views towards post abortion contraception will enable us identify barriers and facilitators among healthcare providers towards post abortion contraception thereby guiding us in formulating context specific evidence based guidelines on post abortion intrauterine contraception.

The estimated duration the research participant will take to in the research project:

You will be interviewed alone just like 20-30 health workers like you though might be of different cadres. We plan to take about 45-90 minutes during the interview.

Procedure:

You will be interviewed about on the barriers and facilitators of post abortion intrauterine contraception after first trimester medical management of incomplete abortion.

Who will participate in the study?

We are planning on interviewing healthcare providers involved in family planning service delivery on the barriers and facilitators of post abortion intrauterine contraception after first trimester medical management of incomplete abortion. We interview 20-30 health workers of different cadres using in depth interviews lasting about 45-90 minutes.

Risks/Discomforts: We anticipate minimal risk to you in this study.

Benefits:

There are no direct benefits to you but the information gathered will help us to make informed recommendations on when to insert intrauterine devices after pregnancy losses within the first 12

weeks especially after using medical treatment to complete the expulsion of the products of conception.

Confidentiality:

Participants' data will be kept in password protected databases. All participants' data (audiotapes, records, transcripts and notes) will be kept in a secure place that will be lockable and accessible only to personnel directly involved in the study. Study participants will be identified by pseudonyms in the final report. The research team will sign confidentiality forms to ensure that they conform to keeping the participants' confidentiality at all times during and after the study.

As the primary investigator and the local Research Ethics Committee (REC) and Uganda National Council for Science and Technology (UNCST) will have access however to your private information that identifies the research participants by name to ensure that your safety is ensured throughout the study period.

Alternatives:

You have a right to decline to participate in the study or withdraw from it at any stage of questioning without it affecting your employment status at the health facility or with the research team.

Cost:

There are no direct costs involving you in this study but should they arise in any ways, the research team will cover them.

Compensation for participation in the study:

You will be given 30,000/= (Thirty thousand Uganda shillings) as a compensation for your time to participate in the study.

Reimbursement:

Study participants will be compensated for their time.

Questions about the study:

The contact of the Principal investigator, School of Medicine and Ethics committee chairman and Secretary of the Uganda National Science and Technology committee are availed in this consent form.

Questions about participants rights:

If there are any questions you would like to ask in regards to the study procedure, any inquiries about the study materials or any concerns while in the study, you can contact Dr. Herbert Kayiga whose contact is listed below. In case you have concerns about the ethical conduct, any dissatisfaction with the study, please contact the chair of the Ethics review board or the National Council of Science and Technology secretary whose contacts are listed below

The contacts are:

1. **Dr. Herbert Kayiga**
   Department of Obstetrics and Gynecology,
   Makerere University College of Health Services,
   P.O. Box 7062, Kampala.
   0777855063.
   Email: hkayiga@gmail.com
2. **Prof Ponsiano Ocama**
   Chairperson:
   SOMREC (School of Medicine Research & Ethics Committee)
   0772421190
   Email: ponsiano.ocama@gmail.com
3. **Dr. Peter Ndemere**
   Executive Secretary- UNCST
   +256414705500
   Email: info@uncst.go.ug

Statement of voluntariness:

You have a right to decline to participate in the study or withdraw from it at any stage of questioning without it affecting your relationship with the research team or your employers.

Dissemination of results:

You will be given feedback on the progress or the findings of the study. Any new information that affects the study or data that has clinical relevance to research participants (including incidental findings) will be made available to you as health care providers.

Ethical approval:

Ethical approvals for this study have been obtained from SOMREC, UNCST, and the Ministry of Health of Uganda. Administrative permissions have also been sought from the directors of the different implementing health units.

STATEMENT OF CONSENT/ASSENT

.... has described to me what is going to be done, the risks, the benefits involved and my rights regarding this study. I understand that my decision to participate in this study will not alter my employment status or the relationship I have with the research team. In the use of this information, my identity will be concealed. I am aware that I may withdraw at any time. I understand that by signing this form, I do not waive any of my legal rights but merely indicate that I have been informed about the research study in which I am voluntarily agreeing to participate. A copy of this form will be provided to me.

Name ………………………Signature/thumb print of participant …………………Date ……

Name ………………………Signature of parent/guardian for minors (If applicable)…Date ……...............................

Name………………………Signature of witness (if applicable)……………Date…………….

Name ……………………….Signature of interviewer/Person obtaining informed consent ……………………Date ………………….

APPENDIX VIII: IN DEPTH INTERVIEW CONSENT FORM FOR WOMEN

MAKERERE UNIVERSITY COLLEGE OF HEALTH SCIENCES

SCHOOL OF MEDICINE RESEARCH AND ETHICS COMMITTEE (SOM-REC)

Title of the proposed study:

WOMEN AND THEIR SPOUSES' PERCEPTIONS TOWARDS POST ABORTION INTRAUTERINE CONTRACEPTION AFTER FIRST TRIMESTER MEDICAL MANAGEMENT OF INCOMPLETE ABORTIONS IN CENTRAL UGANDA: A QUALITATIVE STUDY

Investigator:

DR. HERBERT KAYIGA,
Department of Obstetrics and Gynecology,
Makerere University College of Health Services,
P.O. Box 7062, Kampala.
0777855063. Email: hkayiga@gmail.com

Co-Investigators:

ASSOC. PROF JOSAPHAT BYAMUGISHA,
Makerere University College of Health Sciences,
Department of Obs/Gyn,
P.O. Box 7062, Kampala.
0772580330. Email: jbyamugisha@gmail.com

DR. OTHMAN KAKAIRE
Makerere University College of Health Sciences,
Department of Obs/Gyn,
P.O. Box 7062, Kampala.
0772514616, Email: kakaireothman@gmail.com

ASSOC. PROF NAZARIUS .M. TUMWESIGYE
Makerere University College of Health Sciences,
Department of Epidemiology and Biostatistics
P.O. Box 7062, Kampala.
0782447771, Email: naz@musph.ac.ug

PROF KRISTINA GEMZELL-DANIELSSON
Karolinska Institutet and Karolinska University Hospital
Email: kristina.gemzell@ki.se

Background and rationale for the study:

This study seeks to explore the perceptions of women with or without their spouses using post abortion intrauterine contraception after medical management of first trimester incomplete abortions. Currently the practice has been to have intrauterine contraceptive (copper or Levonorgestrel releasing) devices inserted between 2-4 weeks after expulsion of the products of conception. Unfortunately, many women rarely return to have the devices inserted for various reasons. This exposes them to unintended pregnancies as the ability to conceive returns as early as five to ten days after the pregnancies loss.

It is to this end that we request to interview you on the choice you have taken to use a uterine device as a means to prevent unwanted pregnancy after losing your previous pregnancy for which you were given tablets to put under your tongue to enable you expel the contents which hadn't come out fully. Your views will help us to know if inserting this method after your miscarriage is as safe and effective when put within one week of expelling the products of conception as when inserted 2-4 weeks later which has been the standard of care. We also seek to understand why you opted for the intrauterine device over the other methods of contraception and whether you would recommend this type of contraception to a friend in future. This will enable us to make evidence based recommendations on when to have post abortion intrauterine contraception after medical management of first trimester miscarriages.

A description of sponsors of the research project and the organizational affiliation of the researchers:

I am Dr. Herbert Kayiga, a practicing Obstetrician/Gynaecologist, also a Lecturer in the department of Obstetrics and Gynaecology, Makerere University College of Health Sciences, a researcher in the main study "*Acceptability, Effectiveness and Uptake of Early versus Standard Post Abortion Intrauterine Contraception after First Trimester Medical Post Abortion Care for Incomplete Abortions in Uganda*". Associate Professor Josaphat Byamugisha, Associate Professor Nazarius Tumwesigye, and Dr. Othman Kakaire are also affiliated to Makerere University College of Health Sciences. They are my supervisors on this study project. Professor Kristina Gemzell-Danielsson, is co-investigator based at Karolinska Institutet and Karolinska University Hospital, Sweden.

This study will be conducted in Entebbe hospital, Mpigi HC IV, Masaka hospital, Gombe hospital, Nakaseke hospital, Luweero HC IV, Mengo hospital, , Naguru hospital, Kawempe National referral hospital, Kawolo hospital, Wakiso HC IV, Kasangati HC IV, Kiganda Health Centre IV and Kayunga hospital.

The study will be partly funded through collaboration between Makerere University and Karolinska Institutet and Karolinska University Hospital through Prof Kristina Gemzell-Danielsson. Makerere University has offered a tuition waiver while Karolinska Institutet, will offer some of the research funds.

Purpose:

In this study, we are going to interview you on the choice you have taken to use a uterine device as a means to prevent unwanted pregnancy after losing your previous pregnancy for which you were given tablets to put under your tongue to enable you expel the contents which hadn't come out fully. Your views will help us to know if inserting this method after your miscarriage is as safe and effective when put within one week of expelling the products of conception as when inserted 2-4 weeks later which has been the standard of care. We also seek to understand why you opted for the intrauterine device over the other methods of contraception and whether you would recommend this type of contraception to a friend in future. We seek also to know how you navigated around barriers like cultural norms to take on the post abortion intrauterine contraception.

The estimated duration the research participant will take to in the research project:

We plan to take about 45-90 minutes interviewing you alone or with your spouse in regards to the choice of post abortion contraception you have opted to use after medical managemnent of your first trimester miscarriage.

Procedures:

You will be interviewed on the reasons behind your choice and whether you would recommend the method to your friends or even use the method again in future. We shall use the information you give us to identify the ideal time to insert the uterine devices after incomplete abortion/miscarriage within the first 12 weeks to prevent future unwanted and intended pregnancies. This is so because the ability to get another pregnancy is restored within two weeks

after the expulsion of products of conception. You will be interviewed alone or with your spouse as you will judge most appropriately.

Who will participate in the study?

The study will also entail interviewing 20-30 women or as couples that opt to take on post abortion intrauterine contraception using in depth interviews to understand the barriers and facilitators to the uptake of their family planning options.

Risks/Discomforts: Both the copper T and Levonorgestrel releasing IUDs are already in use in Uganda for family planning. While using the intrauterine devices, the copper IUD, can make you have heavy or painful periods. There’s a risk of bleeding in between your periods. These symptoms can however subside within six months or can be managed medically with drugs like Ibuprofen and other drugs. There’s also an eight percent chance of conceiving while using the method. While using the Levonorgestrel IUD, there might be reduced menstrual flow or at times not seeing any menstrual loss. There’s also a one percent risk of conceiving while using the method. Though the device works locally, sometimes the Levonorgestrel hormone can get into your circulation and this can alter your mood. None of the IUDs can prevent you from contracting HIV or any other sexually transmitted infections especially when you engage in unprotected sex.

In the event that you experience any of the following severe infection following evacuation of the uterine contents, need for surgery (exploratory laparotomy) after post abortion intrauterine device insertions, need for blood transfusion following heavy (severe) hemorrhage, hospitalization over 72 hours, severe unusual reaction (anaphylactic reaction) after IUD insertion, or deaths for you or your spouse, these will be regarded as severe adverse events. Participants with adverse events will be managed immediately by the care teams at the different facilities as per the standard operating procedures. Those who need referral services will be given the best care at the expense of the research team.We shall request you to inform the Principal investigator or research team as soon as the events occur so that timely interventions can be undertaken.

In this particular study, there will be minimal risk anticipated during the interview.

Benefits:

Participants in the study will receive a closer follow up for their medical management of first trimester incomplete abortion as compared to the ones undertaking routine clinical care. They will receive post abortion contraceptive counseling. The intervention will prevent unintended pregnancies a benefit to the participants. As a public health contribution, this study will aid in

formulation of a post abortion contraception policy after first trimester abortions that is currently missing.

We anticipate that the benefits post abortion contraception after first trimester abortions outweigh the harms that can arise from unintended pregnancies and the associated complications.

The research team will take you through all the available family planning options, their efficacy, effectiveness and benefits of the options over the other methods. In the event that the choice you have taken as a contraceptive device is found to be inferior to the other device (copper or Levonorgrel) inserted after medical management of first trimester incomplete abortion, you will have the first priority to change the device without any financial costs to you

Confidentiality:

Patient data will be kept in password protected databases. All participants' data (audiotapes, records, transcripts and notes) will be kept in a secure place that will be lockable and accessible only to personnel directly involved in the study. Study participants will be identified by pseudonyms in the final report. The research team will sign confidentiality forms to ensure that they conform to keeping the participants' confidentiality at all times during and after the study.

As the primary investigator and the local Research Ethics Committee (REC) and Uganda National Council for Science and Technology (UNCST) will have access however to your private information that identifies the research participants by name to ensure that your safety is ensured throughout the study period.

Alternatives:

You have a right to decline to participate in the study or withdraw from it at any stage of questioning without it affecting the quality of care offered to you while you are in hospital. In case you opt for an alternative other than medical management of first trimester incomplete abortion, you will be given the alternative of surgical evacuation to remove the retained products of conception. Other family planning methods other than intrauterine contraception will also be availed to you for your informed decision on what is ideal for you.

Cost:

The costs of the medications like Misoprostol, antibiotics and pain killers during the medical management and the intrauterine devices will be covered by the study team. Any of the adverse effects that might occur to you during the study will be covered by the study team.

Compensation for participation in the study:

You will be given 30,000/= (Thirty thousand Uganda shillings) as a compensation for your time to participate in the study. In cases of any permanent damage or any major injuries during the study, the study participants will have their medical bills covered by the research team.

Reimbursement:

Study participants will be compensated for their time, and will have transport refund.

Questions about the study:

A readily available contact of a study nurse will be offered to all participants over 24 hours every week to address any issues that they will have with the study intervention or process. This will ensure timely response to the participants' concerns. The contact of the Principal investigator, School of Medicine and Ethics committee chairman and Secretary of the Uganda National Science and Technology committee are availed in this consent form.

Questions about participants rights:

If there are any questions you would like to ask in regards to the study procedure, any inquiries about the study materials or any concerns while in the study, you can contact Dr. Herbert Kayiga whose contact is listed below. In case you have concerns about the ethical conduct, any dissatisfaction with the study, please contact the chair of the Ethics review board or the National Council of Science and Technology secretary whose contacts are listed below

The contacts are:

1. **Dr. Herbert Kayiga**
   Department of Obstetrics and Gynecology,
   Makerere University College of Health Services,
   P.O. Box 7062, Kampala.
   0777855063.
   Email: hkayiga@gmail.com
2. **Prof Ponsiano Ocama**
   Chairperson:
   SOMREC (School of Medicine Research & Ethics Committee)
   0772421190
   Email: ponsiano.ocama@gmail.com
3. **Dr. Peter Ndemere**
   Executive Secretary- UNCST
   +256414705500
   Email: info@uncst.go.ug

Statement of voluntariness:

You have a right to decline to participate in the study or withdraw from it at any stage of questioning without it affecting the quality of care offered to you while you are in hospital.

Dissemination of results:

You will be given feedback on the progress or the findings of the study. Any new information that affects the study or data that has clinical relevance to research participants (including incidental findings) will be made available to you and/or your health care providers.

Ethical approval:

Ethical approvals for this study have been obtained from SOMREC, UNCST, and the Ministry of Health of Uganda. Administrative permissions have also been sought from the directors of the different implementing health units. The trials was also registered at ClinicalTrials.gov and reported in accordance with CONSORT guidelines.

STATEMENT OF CONSENT/ASSENT

.... has described to me what is going to be done, the risks, the benefits involved and my rights regarding this study. I understand that my decision to participate in this study will not alter my usual medical care. In the use of this information, my identity will be concealed. I am aware that I may withdraw at any time. I understand that by signing this form, I do not waive any of my legal rights but merely indicate that I have been informed about the research study in which I am voluntarily agreeing to participate. A copy of this form will be provided to me.

Name ………………………Signature/thumb print of participant …………………Date ……

Name ………………………Signature of parent/guardian for minors (If applicable)…Date ……..............................

Name………………………Signature of witness (if applicable)……………Date…………….

Name ……………………….Signature of interviewer/Person obtaining informed consent ……………………Date ………………….

APPENDIX IX: IN DEPTH INTERVIEW ASSENT FORM FOR EMANCIPATED MINORS

MAKERERE UNIVERSITY COLLEGE OF HEALTH SCIENCES

SCHOOL OF MEDICINE RESEARCH AND ETHICS COMMITTEE (SOM-REC)

Title of the proposed study:

WOMEN AND THEIR SPOUSES' PERCEPTIONS TOWARDS POST ABORTION INTRAUTERINE CONTRACEPTION AFTER FIRST TRIMESTER MEDICAL MANAGEMENT OF INCOMPLETE ABORTIONS IN CENTRAL UGANDA: A QUALITATIVE STUDY

Investigator:

DR. HERBERT KAYIGA,
Department of Obstetrics and Gynecology,
Makerere University College of Health Services,
P.O. Box 7062, Kampala.
0777855063. Email: hkayiga@gmail.com

Co-Investigators:

ASSOC. PROF JOSAPHAT BYAMUGISHA,
Makerere University College of Health Sciences,
Department of Obs/Gyn,
P.O. Box 7062, Kampala.
0772580330. Email: jbyamugisha@gmail.com

DR. OTHMAN KAKAIRE
Makerere University College of Health Sciences,
Department of Obs/Gyn,
P.O. Box 7062, Kampala.
0772514616, Email: kakaireothman@gmail.com

ASSOC. PROF NAZARIUS .M. TUMWESIGYE
Makerere University College of Health Sciences,
Department of Epidemiology and Biostatistics
P.O. Box 7062, Kampala.
0782447771, Email: naz@musph.ac.ug

PROF KRISTINA GEMZELL-DANIELSSON
Karolinska Institutet and Karolinska University Hospital
Email: kristina.gemzell@ki.se

Background and rationale for the study:

This study seeks to explore the perceptions of women with or without their spouses using post abortion intrauterine contraception after medical management of first trimester incomplete abortions. Currently the practice has been to have intrauterine contraceptive (copper or Levonorgestrel releasing) devices inserted between 2-4 weeks after expulsion of the products of conception. Unfortunately, many women rarely return to have the devices inserted for various reasons. This exposes them to unintended pregnancies as the ability to conceive returns as early as five to ten days after the pregnancies loss.

It is to this end that we request to interview you on the choice you have taken to use a uterine device as a means to prevent unwanted pregnancy after losing your previous pregnancy for which you were given tablets to put under your tongue to enable you expel the contents which hadn't come out fully. Your views will help us to know if inserting this method after your miscarriage is as safe and effective when put within one week of expelling the products of conception as when inserted 2-4 weeks later which has been the standard of care. We also seek to understand why you opted for the intrauterine device over the other methods of contraception and whether you would recommend this type of contraception to a friend in future. This will enable us to make evidence based recommendations on when to have post abortion intrauterine contraception after medical management of first trimester miscarriages.

A description of sponsors of the research project and the organizational affiliation of the researchers:

I am Dr. Herbert Kayiga, a practicing Obstetrician/Gynaecologist, also a Lecturer in the department of Obstetrics and Gynaecology, Makerere University College of Health Sciences, a researcher in the main study "*Acceptability, Effectiveness and Uptake of Early versus Standard Post Abortion Intrauterine Contraception after First Trimester Medical Post Abortion Care for Incomplete Abortions in Uganda*". Associate Professor Josaphat Byamugisha, Associate Professor Nazarius Tumwesigye, and Dr. Othman Kakaire are also affiliated to Makerere University College of Health Sciences. They are my supervisors on this study project. Professor Kristina Gemzell-Danielsson, is co-investigator based at Karolinska Institutet and Karolinska University Hospital, Sweden.

This study will be conducted in Entebbe hospital, Mpigi HC IV, Masaka hospital, Gombe hospital, Nakaseke hospital, Luweero HC IV, Mengo hospital, , Naguru hospital, Kawempe National referral hospital, Kawolo hospital, Wakiso HC IV, Kasangati HC IV, Kiganda Health Centre IV and Kayunga hospital.

The study will be partly funded through collaboration between Makerere University and Karolinska Institutet and Karolinska University Hospital through Prof Kristina Gemzell-Danielsson. Makerere University has offered a tuition waiver while Karolinska Institutet, will offer some of the research funds.

Purpose:

In this study, we are going to interview you on the choice you have taken to use a uterine device as a means to prevent unwanted pregnancy after losing your previous pregnancy for which you were given tablets to put under your tongue to enable you expel the contents which hadn't come out fully. Your views will help us to know if inserting this method after your miscarriage is as safe and effective when put within one week of expelling the products of conception as when inserted 2-4 weeks later which has been the standard of care. We also seek to understand why you opted for the intrauterine device over the other methods of contraception and whether you would recommend this type of contraception to a friend in future. We seek also to know how you navigated around barriers like cultural norms to take on the post abortion intrauterine contraception.

The estimated duration the research participant will take to in the research project:

We plan to take about 45-90 minutes interviewing you alone or with your spouse in regards to the choice of post abortion contraception you have opted to use after medical managemnent of your first trimester miscarriage.

Procedures:

You will be interviewed on the reasons behind your choice and whether you would recommend the method to your friends or even use the method again in future. We shall use the information you give us to identify the ideal time to insert the uterine devices after incomplete abortion/miscarriage within the first 12 weeks to prevent future unwanted and intended pregnancies. This is so because the ability to get another pregnancy is restored within two weeks

after the expulsion of products of conception. You will be interviewed alone or with your spouse as you will judge most appropriately.

Who will participate in the study?

The study will also entail interviewing 20-30 women or as couples that opt to take on post abortion intrauterine contraception using in depth interviews to understand the barriers and facilitators to the uptake of their family planning options.

Risks/Discomforts: Both the copper T and Levonorgestrel releasing IUDs are already in use in Uganda for family planning. While using the intrauterine devices, the copper IUD, can make you have heavy or painful periods. There's a risk of bleeding in between your periods. These symptoms can however subside within six months or can be managed medically with drugs like Ibuprofen and other drugs. There's also an eight percent chance of conceiving while using the method. While using the Levonorgestrel IUD, there might be reduced menstrual flow or at times not seeing any menstrual loss. There's also a one percent risk of conceiving while using the method. Though the device works locally, sometimes the Levonorgestrel hormone can get into your circulation and this can alter your mood. None of the IUDs can prevent you from contracting HIV or any other sexually transmitted infections especially when you engage in unprotected sex.

In the event that you experience any of the following severe infection following evacuation of the uterine contents, need for surgery (exploratory laparotomy) after post abortion intrauterine device insertions, need for blood transfusion following heavy (severe) hemorrhage, hospitalization over 72 hours, severe unusual reaction (anaphylactic reaction) after IUD insertion, or deaths for you or your spouse, these will be regarded as severe adverse events. Participants with adverse events will be managed immediately by the care teams at the different facilities as per the standard operating procedures. Those who need referral services will be given the best care at the expense of the research team.We shall request you to inform the Principal investigator or research team as soon as the events occur so that timely interventions can be undertaken.

In this particular study, there will be minimal risk anticipated during the interview.

Benefits:

Participants in the study will receive a closer follow up for their medical management of first trimester incomplete abortion as compared to the ones undertaking routine clinical care. They will receive post abortion contraceptive counseling. The intervention will prevent unintended pregnancies a benefit to the participants. As a public health contribution, this study will aid in

formulation of a post abortion contraception policy after first trimester abortions that is currently missing.

We anticipate that the benefits post abortion contraception after first trimester abortions outweigh the harms that can arise from unintended pregnancies and the associated complications.

The research team will take you through all the available family planning options, their efficacy, effectiveness and benefits of the options over the other methods. In the event that the choice you have taken as a contraceptive device is found to be inferior to the other device (copper or Levonorgrel) inserted after medical management of first trimester incomplete abortion, you will have the first priority to change the device without any financial costs to you

Confidentiality:

Patient data will be kept in password protected databases. All participants' data (audiotapes, records, transcripts and notes) will be kept in a secure place that will be lockable and accessible only to personnel directly involved in the study. Study participants will be identified by pseudonyms in the final report. The research team will sign confidentiality forms to ensure that they conform to keeping the participants' confidentiality at all times during and after the study.

As the primary investigator and the local Research Ethics Committee (REC) and Uganda National Council for Science and Technology (UNCST) will have access however to your private information that identifies the research participants by name to ensure that your safety is ensured throughout the study period.

Alternatives:

You have a right to decline to participate in the study or withdraw from it at any stage of questioning without it affecting the quality of care offered to you while you are in hospital. In case you opt for an alternative other than medical management of first trimester incomplete abortion, you will be given the alternative of surgical evacuation to remove the retained products of conception. Other family planning methods other than intrauterine contraception will also be availed to you for your informed decision on what is ideal for you.

Cost:

The costs of the medications like Misoprostol, antibiotics and pain killers during the medical management and the intrauterine devices will be covered by the study team. Any of the adverse effects that might occur to you during the study will be covered by the study team.

Compensation for participation in the study:

You will be given 30,000/= (Thirty thousand Uganda shillings) as a compensation for your time to participate in the study. In cases of any permanent damage or any major injuries during the study, the study participants will have their medical bills covered by the research team.

Reimbursement:

Study participants will be compensated for their time, and will have transport refund.

Questions about the study:

A readily available contact of a study nurse will be offered to all participants over 24 hours every week to address any issues that they will have with the study intervention or process. This will ensure timely response to the participants' concerns. The contact of the Principal investigator, School of Medicine and Ethics committee chairman and Secretary of the Uganda National Science and Technology committee are availed in this consent form.

Questions about participants rights:

If there are any questions you would like to ask in regards to the study procedure, any inquiries about the study materials or any concerns while in the study, you can contact Dr. Herbert Kayiga whose contact is listed below. In case you have concerns about the ethical conduct, any dissatisfaction with the study, please contact the chair of the Ethics review board or the National Council of Science and Technology secretary whose contacts are listed below

The contacts are:

1. **Dr. Herbert Kayiga**
   Department of Obstetrics and Gynecology,
   Makerere University College of Health Services,
   P.O. Box 7062, Kampala.
   0777855063.
   Email: hkayiga@gmail.com
2. **Prof Ponsiano Ocama**
   Chairperson:
   SOMREC (School of Medicine Research & Ethics Committee)
   0772421190
   Email: ponsiano.ocama@gmail.com
3. **Dr. Peter Ndemere**
   Executive Secretary- UNCST
   +256414705500
   Email: info@uncst.go.ug

Statement of voluntariness:

You have a right to decline to participate in the study or withdraw from it at any stage of questioning without it affecting the quality of care offered to you while you are in hospital.

Dissemination of results:

You will be given feedback on the progress or the findings of the study. Any new information that affects the study or data that has clinical relevance to research participants (including incidental findings) will be made available to you and/or your health care providers.

Ethical approval:

Ethical approvals for this study have been obtained from SOMREC, UNCST, and the Ministry of Health of Uganda. Administrative permissions have also been sought from the directors of the different implementing health units. The trials was also registered at ClinicalTrials.gov and reported in accordance with CONSORT guidelines.

STATEMENT OF ASSENT

.... has described to me what is going to be done, the risks, the benefits involved and my rights regarding this study. I understand that my decision to participate in this study will not alter my usual medical care. In the use of this information, my identity will be concealed. I am aware that I may withdraw at any time. I understand that by signing this form, I do not waive any of my legal rights but merely indicate that I have been informed about the research study in which I am voluntarily agreeing to participate. A copy of this form will be provided to me.

Name ………………………Signature/thumb print of participant …………………Date ……

Name ………………………Signature of parent/guardian for minors (If applicable)…Date ……...............................

Name………………………Signature of witness (if applicable)……………Date…………….

Name ……………………….Signature of interviewer/Person obtaining informed consent ……………………Date ………………….

APPENDIX X: IN DEPTH INTERVIEW CONSENT FORM FOR SPOUSES

MAKERERE UNIVERSITY COLLEGE OF HEALTH SCIENCES

SCHOOL OF MEDICINE RESEARCH AND ETHICS COMMITTEE (SOM-REC)

Title of the proposed study:

WOMEN AND THEIR SPOUSES' PERCEPTIONS TOWARDS POST ABORTION INTRAUTERINE CONTRACEPTION AFTER FIRST TRIMESTER MEDICAL MANAGEMENT OF INCOMPLETE ABORTIONS IN CENTRAL UGANDA: A QUALITATIVE STUDY

Investigator:

DR. HERBERT KAYIGA,
Department of Obstetrics and Gynecology,
Makerere University College of Health Services,
P.O. Box 7062, Kampala.
0777855063. Email: hkayiga@gmail.com

Co-Investigators:

ASSOC. PROF JOSAPHAT BYAMUGISHA,
Makerere University College of Health Sciences,
Department of Obs/Gyn,
P.O. Box 7062, Kampala.
0772580330. Email: jbyamugisha@gmail.com

DR. OTHMAN KAKAIRE
Makerere University College of Health Sciences,
Department of Obs/Gyn,
P.O. Box 7062, Kampala.
0772514616, Email: kakaireothman@gmail.com

ASSOC. PROF NAZARIUS .M. TUMWESIGYE
Makerere University College of Health Sciences,
Department of Epidemiology and Biostatistics
P.O. Box 7062, Kampala.
0782447771, Email: naz@musph.ac.ug

PROF KRISTINA GEMZELL-DANIELSSON
Karolinska Institutet and Karolinska University Hospital
Email: kristina.gemzell@ki.se

Background and rationale for the study:

This study seeks to explore the perceptions of women with or without their spouses using post abortion intrauterine contraception after medical management of first trimester incomplete abortions. Currently the practice has been to have intrauterine contraceptive (copper or Levonorgestrel releasing) devices inserted between 2-4 weeks after expulsion of the products of conception. Unfortunately, many women rarely return to have the devices inserted for various

reasons. This exposes them to unintended pregnancies as the ability to conceive returns as early as five to ten days after the pregnancies loss.

It is to this end that we request to interview you on the choice your spouse has taken to use a uterine device as a means to prevent unwanted pregnancy after losing her previous pregnancy for which she was given tablets to put under her tongue to enable her expel the contents which hadn't come out fully. Your views will help us to know if inserting this method after the miscarriage is as safe and effective when put within one week of expelling the products of conception as when inserted 2-4 weeks later which has been the standard of care. We also seek to understand why you opted for the intrauterine device over the other methods of contraception and whether you would recommend this type of contraception to a friend in future. This will enable us to make evidence based recommendations on when to have post abortion intrauterine contraception after medical management of first trimester miscarriages.

A description of sponsors of the research project and the organizational affiliation of the researchers:

I am Dr. Herbert Kayiga, a practicing Obstetrician/Gynaecologist, also a Lecturer in the department of Obstetrics and Gynaecology, Makerere University College of Health Sciences, a researcher in the main study "*Acceptability, Effectiveness and Uptake of Early versus Standard Post Abortion Intrauterine Contraception after First Trimester Medical Post Abortion Care for Incomplete Abortions in Uganda*". Associate Professor Josaphat Byamugisha, Associate Professor Nazarius Tumwesigye, and Dr. Othman Kakaire are also affiliated to Makerere University College of Health Sciences. They are my supervisors on this study project. Professor Kristina Gemzell-Danielsson, is co-investigator based at Karolinska Institutet and Karolinska University Hospital, Sweden.

This study will be conducted in Entebbe hospital, Mpigi HC IV, Masaka hospital, Gombe hospital, Nakaseke hospital, Luweero HC IV, Mengo hospital,, Naguru hospital, Kawempe National referral hospital, Kawolo hospital, Wakiso HC IV, Kasangati HC IV, Kiganda Health Centre IV and Kayunga hospital.

The study will be partly funded through collaboration between Makerere University and Karolinska Institutet and Karolinska University Hospital through Prof Kristina Gemzell-

Danielsson. Makerere University has offered a tuition waiver while Karolinska Institutet, will offer some of the research funds.

Purpose:

In this study, we are going to interview you on the choice your spouse has taken to use a uterine device as a means to prevent unwanted pregnancy after losing the previous pregnancy for which she were given Misoprostol tablets to put under her tongue to enable her expel the contents which hadn't come out fully. Your views will help us to know if inserting this method after the miscarriage is as safe and effective when put within one week of expelling the products of conception as when inserted 2-4 weeks later which has been the standard of care. We also seek to understand why you opted for the intrauterine device over the other methods of contraception and whether you would recommend this type of contraception to a friend in future. We seek also to know how you navigated around barriers like cultural norms to take on the post abortion intrauterine contraception.

The estimated duration the research participant will take to in the research project:

We plan to take about 45-90 minutes interviewing you alone or with your spouse in regards to the choice of post abortion contraception you have opted to use after medical managemnent of your first trimester miscarriage.

Procedures:

You will be interviewed on the reasons behind your choice and whether you would recommend the method to your friends or even use the method again in future. We shall use the information you give us to identify the barriers and facilitators of post abortion intrauterine contraception among couples. Such information will enable us guide in formulation of context specific evidence based guidelines on post abortion contraception. You will be interviewed alone or with your spouse as you will judge most appropriately.

Who will participate in the study?

The study will also entail interviewing 20-30 couples that opt to take on post abortion intrauterine contraception using in depth interviews to understand the barriers and facilitators to the uptake of their family planning options.

Risks/Discomforts: Both the copper T and Levonorgestrel releasing IUDs are already in use in Uganda for family planning. While using the intrauterine devices, the copper IUD, can make your

spouse to have heavy or painful periods. There's a risk of bleeding in between her periods. These symptoms can however subside within six months or can be managed medically with drugs like Ibuprofen and other drugs. There's also an eight percent chance of conceiving while using the method. While using the Levonorgestrel IUD, there might be reduced menstrual flow or at times not seeing any menstrual loss. There's also a one percent risk of conceiving while using the method. Though the device works locally, sometimes the Levonorgestrel hormone can get into her circulation and this can alter her mood. None of the IUDs can prevent you from contracting HIV or any other sexually transmitted infections especially when you engage in unprotected sex.

In this particular study, there will be minimal risk anticipated during the interview.

Benefits:

The intervention your spouse is using will prevent unintended pregnancies. As a public health contribution, this study will aid in formulation of a post abortion contraception policy after first trimester abortions that is currently missing.

We anticipate that the benefits post abortion contraception after first trimester abortions outweigh the harms that can arise from unintended pregnancies and the associated complications.

The research team will take you through all the available family planning options, their efficacy, effectiveness and benefits of the options over the other methods. In the event that the choice you or your spouse have taken as a contraceptive device is found to be inferior to the other device (copper or Levonorgrel) inserted after medical management of first trimester incomplete abortion, you will have the first priority to change the device without any financial costs to you

Confidentiality:

Patient data will be kept in password protected databases. All participants' data (audiotapes, records, transcripts and notes) will be kept in a secure place that will be lockable and accessible only to personnel directly involved in the study. Study participants will be identified by pseudonyms in the final report. The research team will sign confidentiality forms to ensure that they conform to keeping the participants' confidentiality at all times during and after the study.

As the primary investigator and the local Research Ethics Committee (REC) and Uganda National Council for Science and Technology (UNCST) will have access however to your private information that identifies the research participants by name to ensure that you and your spouse's safety is ensured throughout the study period.

Alternatives:

You have a right to decline to participate in the study or withdraw from it at any stage of questioning without it affecting the quality of care offered to you or your spouse while you are in hospital. Other family planning methods other than intrauterine contraception will also be availed to you and your spouse for your informed decision on what is ideal for you.

Cost:

The costs of the medications like Misoprostol, antibiotics and pain killers during the medical management and the intrauterine devices will be covered by the study team. Any of the adverse effects that might occur to you or your spouse during the study will be covered by the study team.

Compensation for participation in the study:

You will be given 30,000/= (Thirty thousand Uganda shillings) as a compensation for your time to participate in the study. In cases of any permanent damage or any major injuries during the study, the study participants will have their medical bills covered by the research team.

Reimbursement:

Study participants will be compensated for their time, and will have transport refund.

Questions about the study:

A readily available contact of a study nurse will be offered to all participants over 24 hours every week to address any issues that they will have with the study intervention or process. This will ensure timely response to the participants' concerns. The contact of the Principal investigator, School of Medicine and Ethics committee chairman and Secretary of the Uganda National Science and Technology committee are availed in this consent form.

Questions about participants rights:

If there are any questions you would like to ask in regards to the study procedure, any inquiries about the study materials or any concerns while in the study, you can contact Dr. Herbert Kayiga whose contact is listed below. In case you have concerns about the ethical conduct, any dissatisfaction with the study, please contact the chair of the Ethics review board or the National Council of Science and Technology secretary whose contacts are listed below

The contacts are:

1. **Dr. Herbert Kayiga**

Department of Obstetrics and Gynecology,
Makerere University College of Health Services,
P.O. Box 7062, Kampala.
0777855063.
Email: hkayiga@gmail.com

2. **Prof Ponsiano Ocama**

Chairperson:
SOMREC (School of Medicine Research & Ethics Committee)
0772421190
Email: ponsiano.ocama@gmail.com

3. **Dr. Peter Ndemere**

Executive Secretary- UNCST
+256414705500
Email: info@uncst.go.ug

Statement of voluntariness:

You have a right to decline to participate in the study or withdraw from it at any stage of questioning without it affecting the quality of care offered to you or spouse while you are in hospital.

Dissemination of results:

You will be given feedback on the progress or the findings of the study. Any new information that affects the study or data that has clinical relevance to research participants (including incidental findings) will be made available to you and/or your health care providers.

Ethical approval:

Ethical approvals for this study have been obtained from SOMREC, UNCST, and the Ministry of Health of Uganda. Administrative permissions have also been sought from the directors of the different implementing health units.

STATEMENT OF CONSENT/ASSENT

.... has described to me what is going to be done, the risks, the benefits involved and my rights regarding this study. I understand that my decision to participate in this study will not alter my spouse's medical care. In the use of this information, my identity will be concealed. I am aware that I may withdraw at any time. I understand that by signing this form, I do not waive any of my legal rights but merely indicate that I have been informed about the research study in which I am voluntarily agreeing to participate. A copy of this form will be provided to me.

Name ………………………Signature/thumb print of participant …………………Date ……

Name ………………………Signature of parent/guardian for minors (If applicable)…Date ……...............................

Name………………………Signature of witness (if applicable)……………Date…………….

Name ……………………….Signature of interviewer/Person obtaining informed consent ……………………Date ………………….

APPENDIX XI: IN DEPTH INTERVIEW ASSENT FORM FOR EMANCIPATED MINORS

MAKERERE UNIVERSITY COLLEGE OF HEALTH SCIENCES

SCHOOL OF MEDICINE RESEARCH AND ETHICS COMMITTEE (SOM-REC)

Title of the proposed study:

WOMEN AND THEIR SPOUSES' PERCEPTIONS TOWARDS POST ABORTION INTRAUTERINE CONTRACEPTION AFTER FIRST TRIMESTER MEDICAL MANAGEMENT OF INCOMPLETE ABORTIONS IN CENTRAL UGANDA: A QUALITATIVE STUDY

Investigator:

DR. HERBERT KAYIGA,
Department of Obstetrics and Gynecology,
Makerere University College of Health Services,
P.O. Box 7062, Kampala.
0777855063. Email: hkayiga@gmail.com

Co-Investigators:

ASSOC. PROF JOSAPHAT BYAMUGISHA,
Makerere University College of Health Sciences,
Department of Obs/Gyn,
P.O. Box 7062, Kampala.
0772580330. Email: jbyamugisha@gmail.com

DR. OTHMAN KAKAIRE
Makerere University College of Health Sciences,
Department of Obs/Gyn,
P.O. Box 7062, Kampala.
0772514616, Email: kakaireothman@gmail.com

ASSOC. PROF NAZARIUS .M. TUMWESIGYE
Makerere University College of Health Sciences,
Department of Epidemiology and Biostatistics
P.O. Box 7062, Kampala.
0782447771, Email: naz@musph.ac.ug

PROF KRISTINA GEMZELL-DANIELSSON
Karolinska Institutet and Karolinska University Hospital
Email: kristina.gemzell@ki.se

Background and rationale for the study:

This study seeks to explore the perceptions of women with or without their spouses using post abortion intrauterine contraception after medical management of first trimester incomplete abortions. Currently the practice has been to have intrauterine contraceptive (copper or Levonorgestrel releasing) devices inserted between 2-4 weeks after expulsion of the products of conception. Unfortunately, many women rarely return to have the devices inserted for various reasons. This exposes them to unintended pregnancies as the ability to conceive returns as early as five to ten days after the pregnancies loss.

It is to this end that we request to interview you on the choice your spouse has taken to use a uterine device as a means to prevent unwanted pregnancy after losing her previous pregnancy for which she was given tablets to put under her tongue to enable her expel the contents which hadn't come out fully. Your views will help us to know if inserting this method after the miscarriage is as safe and effective when put within one week of expelling the products of conception as when inserted 2-4 weeks later which has been the standard of care. We also seek to understand why you opted for the intrauterine device over the other methods of contraception and whether you would recommend this type of contraception to a friend in future. This will enable us to make evidence based recommendations on when to have post abortion intrauterine contraception after medical management of first trimester miscarriages.

A description of sponsors of the research project and the organizational affiliation of the researchers:

I am Dr. Herbert Kayiga, a practicing Obstetrician/Gynaecologist, also a Lecturer in the department of Obstetrics and Gynaecology, Makerere University College of Health Sciences, a researcher in the main study "*Acceptability, Effectiveness and Uptake of Early versus Standard Post Abortion Intrauterine Contraception after First Trimester Medical Post Abortion Care for Incomplete Abortions in Uganda*". Associate Professor Josaphat Byamugisha, Associate Professor Nazarius Tumwesigye, and Dr. Othman Kakaire are also affiliated to Makerere University College of Health Sciences. They are my supervisors on this study project. Professor Kristina Gemzell-Danielsson, is co-investigator based at Karolinska Institutet and Karolinska University Hospital, Sweden.

This study will be conducted in Entebbe hospital, Mpigi HC IV, Masaka hospital, Gombe hospital, Nakaseke hospital, Luweero HC IV, Mengo hospital, Naguru hospital, Kawempe National referral hospital, Kawolo hospital, Wakiso HC IV, Kasangati HC IV, Kiganda Health Centre IV and Kayunga hospital.

The study will be partly funded through collaboration between Makerere University and Karolinska Institutet and Karolinska University Hospital through Prof Kristina Gemzell-Danielsson. Makerere University has offered a tuition waiver while Karolinska Institutet, will offer some of the research funds.

Purpose:

In this study, we are going to interview you on the choice your spouse has taken to use a uterine device as a means to prevent unwanted pregnancy after losing the previous pregnancy for which she were given Misoprostol tablets to put under her tongue to enable her expel the contents which hadn't come out fully. Your views will help us to know if inserting this method after the miscarriage is as safe and effective when put within one week of expelling the products of conception as when inserted 2-4 weeks later which has been the standard of care. We also seek to understand why you opted for the intrauterine device over the other methods of contraception and whether you would recommend this type of contraception to a friend in future. We seek also to know how you navigated around barriers like cultural norms to take on the post abortion intrauterine contraception.

The estimated duration the research participant will take to in the research project:

We plan to take about 45-90 minutes interviewing you alone or with your spouse in regards to the choice of post abortion contraception you have opted to use after medical managemnent of your first trimester miscarriage.

Procedures:

You will be interviewed on the reasons behind your choice and whether you would recommend the method to your friends or even use the method again in future. We shall use the information you give us to identify the barriers and facilitators of post abortion intrauterine contraception among couples. Such information will enable us guide in formulation of context specific evidence based guidelines on post abortion contraception. You will be interviewed alone or with your spouse as you will judge most appropriately.

Who will participate in the study?

The study will also entail interviewing 20-30 couples that opt to take on post abortion intrauterine contraception using in depth interviews to understand the barriers and facilitators to the uptake of their family planning options.

Risks/Discomforts: Both the copper T and Levonorgestrel releasing IUDs are already in use in Uganda for family planning. While using the intrauterine devices, the copper IUD, can make your spouse to have heavy or painful periods. There's a risk of bleeding in between her periods. These symptoms can however subside within six months or can be managed medically with drugs like

Ibuprofen and other drugs. There's also an eight percent chance of conceiving while using the method. While using the Levonorgestrel IUD, there might be reduced menstrual flow or at times not seeing any menstrual loss. There's also a one percent risk of conceiving while using the method. Though the device works locally, sometimes the Levonorgestrel hormone can get into your circulation and this can alter your mood. None of the IUDs can prevent you from contracting HIV or any other sexually transmitted infections especially when you engage in unprotected sex.

In this particular study, there will be minimal risk anticipated during the interview.

Benefits:

The intervention your spouse is using will prevent unintended pregnancies. As a public health contribution, this study will aid in formulation of a post abortion contraception policy after first trimester abortions that is currently missing.

We anticipate that the benefits post abortion contraception after first trimester abortions outweigh the harms that can arise from unintended pregnancies and the associated complications.

The research team will take you through all the available family planning options, their efficacy, effectiveness and benefits of the options over the other methods. In the event that the choice you or your spouse have taken as a contraceptive device is found to be inferior to the other device (copper or Levonorgrel) inserted after medical management of first trimester incomplete abortion, you will have the first priority to change the device without any financial costs to you.

Confidentiality:

Patient data will be kept in password protected databases. All participants' data (audiotapes, records, transcripts and notes) will be kept in a secure place that will be lockable and accessible only to personnel directly involved in the study. Study participants will be identified by pseudonyms in the final report. The research team will sign confidentiality forms to ensure that they conform to keeping the participants' confidentiality at all times during and after the study.

As the primary investigator and the local Research Ethics Committee (REC) and Uganda National Council for Science and Technology (UNCST) will have access however to your private information that identifies the research participants by name to ensure that you and your spouse's safety is ensured throughout the study period.

Alternatives:

You have a right to decline to participate in the study or withdraw from it at any stage of questioning without it affecting the quality of care offered to you or your spouse while you are in hospital. Other family planning methods other than intrauterine contraception will also be availed to you and your spouse for your informed decision on what is ideal for you.

Cost:

The costs of the medications like Misoprostol, antibiotics and pain killers during the medical management and the intrauterine devices will be covered by the study team. Any of the adverse effects that might occur to you or your spouse during the study will be covered by the study team.

Compensation for participation in the study:

You will be given 30,000/= (Thirty thousand Uganda shillings) as a compensation for your time to participate in the study. In cases of any permanent damage or any major injuries during the study, the study participants will have their medical bills covered by the research team.

Reimbursement:

Study participants will be compensated for their time, and will have transport refund.

Questions about the study:

A readily available contact of a study nurse will be offered to all participants over 24 hours every week to address any issues that they will have with the study intervention or process. This will ensure timely response to the participants' concerns. The contact of the Principal investigator, School of Medicine and Ethics committee chairman and Secretary of the Uganda National Science and Technology committee are availed in this consent form.

Questions about participants rights:

If there are any questions you would like to ask in regards to the study procedure, any inquiries about the study materials or any concerns while in the study, you can contact Dr. Herbert Kayiga whose contact is listed below. In case you have concerns about the ethical conduct, any dissatisfaction with the study, please contact the chair of the Ethics review board or the National Council of Science and Technology secretary whose contacts are listed below

The contacts are:

1. **Dr. Herbert Kayiga**
   Department of Obstetrics and Gynecology,
   Makerere University College of Health Services,

P.O. Box 7062, Kampala.
0777855063.
Email: hkayiga@gmail.com

2. **Prof Ponsiano Ocama**
Chairperson:
SOMREC (School of Medicine Research & Ethics Committee)
0772421190
Email: ponsiano.ocama@gmail.com

3. **Dr. Peter Ndemere**
Executive Secretary- UNCST
+256414705500
Email: info@uncst.go.ug

Statement of voluntariness:

You have a right to decline to participate in the study or withdraw from it at any stage of questioning without it affecting the quality of care offered to you or spouse while you are in hospital.

Dissemination of results:

You will be given feedback on the progress or the findings of the study. Any new information that affects the study or data that has clinical relevance to research participants (including incidental findings) will be made available to you and/or your health care providers.

Ethical approval:

Ethical approvals for this study have been obtained from SOMREC, UNCST, and the Ministry of Health of Uganda. Administrative permissions have also been sought from the directors of the different implementing health units.

STATEMENT OF ASSENT

.... has described to me what is going to be done, the risks, the benefits involved and my rights regarding this study. I understand that my decision to participate in this study will not alter my spouse's medical care. In the use of this information, my identity will be concealed. I am aware that I may withdraw at any time. I understand that by signing this form, I do not waive any of my legal rights but merely indicate that I have been informed about the research study in which I am voluntarily agreeing to participate. A copy of this form will be provided to me.

Name ………………………Signature/thumb print of participant …………………Date ……
Name ………………………Signature of parent/guardian for minors (If applicable)…Date ……..............................

Name………………………Signature of witness (if applicable)……………Date…………….
Name ……………………….Signature of interviewer/Person obtaining informed consent ……………………Date ………………….

APPENDIX XII: LUGANDA CONSENT RCT

EBYONGERWAKO: EKIWANDIIKO KY'OKUKKIRIZA NGA OTEGEDDE (EKIWA OLUKUSA) EKYA RCT

MAKERERE UNIVERSITY COLLEGE OF HEALTH SCIENCES

SCHOOL OF MEDICINE RESEARCH AND ETHICS COMMITTEE (SOM-REC)

Omutwe gw'okunoonyereza okugenda okukolebwa:

EFFECTIVENESS OF EARLY VERSUS STANDARD INTRAUTERINE CONTRACEPTION FOLLOWING PROVISION OF FIRST TRIMESTER MEDICAL POST ABORTION CARE IN CENTRAL UGANDA: A NON–INFERIORITY RANDOMIZED CLINICAL TRIAL

Omunoonyereza:

DR. HERBERT KAYIGA,
Department of Obstetrics and Gynecology,
Makerere University College of Health Services,
P.O. Box 7062, Kampala.
0777855063. Email: hkayiga@gmail.com

Abakolera awamu n'omunoonyereza:

ASSOC. PROF JOSAPHAT BYAMUGISHA,
Makerere University College of Health Sciences,
Department of Obs/Gyn,
P.O. Box 7062, Kampala.
0772580330. Email: jbyamugisha@gmail.com

DR. OTHMAN KAKAIRE
Makerere University College of Health Sciences,
Department of Obs/Gyn,
P.O. Box 7062, Kampala.
0772514616, Email: kakaireothman@gmail.com

ASSOC. PROF NAZARIUS .M. TUMWESIGYE
Makerere University College of Health Sciences,
Department of Epidemiology and Biostatistics
P.O. Box 7062, Kampala.
0782447771, Email: naz@musph.ac.ug

PROF KRISTINA GEMZELL-DANIELSSON
Karolinska Institutet and Karolinska University Hospital
Email: kristina.gemzell@ki.se

Ebyafaayo n'ensonga ezikwata ku kunoonyereza:

Okunoonyereza kuno kunoonya okuzuula ekiseera ekirungi eky'okussibwamu akaweta akaziyiza olubuto mu nnabaana oluvannyuma lw'okufuna obujjanjabi bw'olubuto olutavuddemu bulungi mu myezi esatu egisooka egy'okufuna olubuto mu bitundu bya Uganda eya Wakati. Mu kiseera kino enkola ebadde yakuteekamu kaweta akaziyiza olubuto mu nnabaana (akavaamu ekirungo kya Copper oba Levonorgestrel) wakati wa wiiki 2-4 oluvannyuma lw'okuggibwamu kw'ebiva mu kufuna olubuto. Ebyembi, abakazi bangi tebatera kukomawo kussibwamu buweta olw'ensonga eziwerako. Kino kibateeka mu kabi ak'okufuna embuto ezitali ŋŋenderere kubanga obusobozi

bw'okufuna olubuto bukomawo mangu mu nnaku nga ttaano ku kkumi oluvannyuma lw'okuvaamu embuto.

N'olw'ekyo kye tuva tusaba okukubuuza ebikwata ku kweroboza kw'okoze okukozesa akaweta akassibwa mu nnabaana nga engeri y'okuziyiza embuto ezitayagalwa oluvannyuma lw'okuvaamu olubuto lw'obadde nalwo nga oweereddwa amakerenda ga Misoprostol ogateeke wansi w'olulimi lwo okukusobozesa okuggyamu ebintu ebyali bitavuddeeyo bulungi. Ebirowoozo byo bigenda kutusobozesa okumanya oba okuteekamu enkola eno oluvannyuma lw'okuvaamu olubuto tekirina kabi era nga kikola bulungi nga essiddwamu mu bbanga lya wiiki emu ey'okuggibwamu kw'ebiva mu kufuna olubuto nga bw'essibwamu oluvannyuma lwa wiiki 2-4 nga y'ebadde enzijjanjaba eya bulijjoe. Era tunoonya okutegeera lwaki wasazeewo okuteekebwamu akaweta mu nnabaana mu kifo ky'enkola endala ez'okuziyiza okufuna olubuto era oba nga owagira mukwano gwo akozese enkola ey'ekika kino okuziyiza olubuto mu kiseera eky'omu maaso. Kino kigenda kutusobozesa okuwa amagezi nga twesigama ku bujulizi ku kiseera eky'okussibwamu akaweta akaziyiza olubuto mu nnabaana oluvannyuma lw'okuvaamu olubuto oluvannyuma lw'okufuna obujjanjabi mu myezi esatu egisooka nga embuto zivuddemu.

Ebikwata ku bawagira omulimu gw'okunoonyereza n'ebitongole ebikolagana n'abanoonyereza:

Nze Dr. Herbert Kayiga, nkola ku by'Okuzaalisa/endwadde z'abakyala (Obstetrician/Gynaecologist), era ndi Musomesa mu kitongole ky'Ebyokuzaalisa n'Endwadde z'abakyala (Obstetrics and Gynaecology), ekya Makerere University College of Health Sciences, omunoonyereza mu kunoonyereza okukulu "*Okukkirizika, n'Enkola n'Enkozesa y'Akaweta akaziyiza olubuto mu nnabaana nga bukyali oluvannyuma lw'okuvaamu olubuto mu kugerageranya n'enkozesa y'akaweta akaziyiza olubuto mu nnabaana eya bulijjo oluvannyuma lw'okufuna obujjanjabi bw'embuto ezitavuddemu bulungi mu myezi esatu egisooka egy'okufuna olubuto mu bitundu bya Uganda eya Wakati" ("Acceptability, Effectiveness and Uptake of Early versus Standard Post Abortion Intrauterine Contraception after First Trimester Medical Post Abortion Care for Incomplete Abortions in Central Uganda").* Associate Professor Josaphat Byamugisha, Associate Professor Nazarius Tumwesigye, ne Dr. Othman Kakaire nabo bakolaganira wamu ne Makerere University College of Health Sciences. Bebannuŋŋamya ku mulimu guno ogw'okunoonyereza. Professor Kristina Gemzell-Danielsson, akolera wamu nange

mu kunoonyereza ng’asinziira ku kitongole kya Karolinska Institutet ne Karolinska University Hospital, Sweden.

Okunoonyereza kuno kugenda kukolebwa mu bifo ewajjanjabirwa kkumi na bitaano (15) ebigenda okubeeramu eddwaliro lya Entebbe hospital, ne Mpigi HC IV, ne Masaka hospital, ne Gombe hospital, ne Nakaseke hospital, ne Luweero HC IV, ne Mengo hospital, , ne Naguru hospital, ne Kawempe National referral hospital, ne Kawolo hospital, ne Wakiso HC IV, ne Kasangati HC IV, ne Kiganda Health Centre IV ne Kayunga hospital.

Okunoonyereza kugenda kussibwamu ekitundu ku ssente okuyita mu nkolagana wakati wa Makerere University ne Karolinska Institutet ne Karolinska University Hospital okuyita mu Prof Kristina Gemzell-Danielsson. Makerere University esazeeko ku bisale by’essomero ate Karolinska Institutet, egenda kuwaayo ezimu ku ssente z’okunoonyereza.

Ekigendererwa:

Mu kunoonyereza kuno, tunoonyereza okumanya oba bw’oba nga ossiddwamu akaweta mu nnabaana mu bbanga lya wiiki emu nga okozesezza mu ngeri ey’obujjanjabi (amakerenda ga Misoprostol) okumalamu ebiva mu kufuna olubuto mu myezi esatu egy’okufuna olubuto, kikola bulungi okufaanana ne bwe kassibwamu oluvannyuma lwa wiiki 2-4 nga bwe bubadde obujjanjabi obwa bulijjo. Obusobozi bwo obw’okufuna olubuto oluvannyuma lw’okuvaamu olubuto mu bbanga erya wiiki kkumi na bbiri buli waggulu so nga ate tetwagala wettike lubuto lw’otayagala oba lw’otagenderedde.

Ekiseera ekiteeberezebwa ayeetabye mu kunoonyereza ky’ajja okutwala ng’ali mu kunoonyereza:

Tuteekateeka okutwala eddakiika nga 30-45 mu bibuuzo ebibuuzibwa. Tujja kwetaaga okomewo olw’okukyala kwo okw’okulondoolwa ku wiiki 2, 3, 6, ne ku myezi 12.

Engeri akaweta gye kakolamu:

Olina okweroboza ku kika ky’akaweta akassibwa mu nnabaana k’oyagala bakuteekemu. Akaweta aka copper IUD akassibwa mu nnabaana kasobola okukuyamba obutafuna lubuto okumala emyaka nga kkumi. Wabula osobola okusaba omusawo yenna akuyambe okukaggyamu lw’oba oyagadde okufuna olubuto oba okukozesa enkola endala yonna. Ekika kino kyennyini eky’akaweta akassibwa mu nnabaana kikola nga kireetera embeera eri mu nnabaana okuba nga tesobozesa kufuna lubuto nga kivaamu ekirungo ekiyitibwa copper ions. Copper ions kye zikola kisobola okukuleetera okulwa mu nsonga, okusinga ku kulumizibwa mu lubuto okwa bulijjo nga oli mu

nsonga. Bulijjo obubonero buno busobola okukendeera mu bbanga lya myezi mukaaga. Newankubadde nga kisobola okukuyamba okuziyiza okufuna olubuto n'ebitundutundu kyenda mu bibiri ku buli kikumi (92%) ku mirundi, waliwo omukisa gwa bitundutundu munaana ku buli kikumi ogw'okufun olubuto nga okozesa enkola eno. Enkola eno ekolerawo mangu oluvannyuma lw'okugiteekamu nga omaze okufuna obujjanjabi obw'olubuto olutavuddemu bulungi mu myezi esatu egisooka egy'okufuna olubuto.

Akaweta aka Levonorgestrel akassibwa mu nnabaana kasobla okukuyamba okulwawo okufuna olubuto okumala emyaka ettaano nga kamaze okussibwamu. Kakola nga kayita mu kufulumya ekirungo ekitonotono ekya Levonorgestrel ekiziyiza obusobozi bwo obw'okufulumya eggi erisobola okuwakisibwa. Enkola eno era ereetera amazzi go agabeera ku mumwa gwa nnabaana okubeera amakwafu ng'eminyira era mu kukola bwe gatyo, gaziyiza amazzi g'omusajja okuyitamu. Newankubadde nga ebitundutundu kyenda mu mwenda ku buli kikumi (99%) ku mirundi, tosobolakufuna lubuto nga okozesa enkola eno, waliwo omukisa gwa katundutundu kamu ku buli kikumi ogw'okufuna olubuto nga okozesa enkola eno.

Ebigobererwa mu kunoonyereza:

Tugenda kubagabanyamu ebibinja bibiri (ekimu nga akaweta kagenda kussibwamu mu wiiki emu) oba (wakati wa wiiki 2-4) nga omaze okufuna obujjanjabi bw'olubuto olutavuddemu bulungi mu bbanga ery'emyezi esatu egisooka egyokufuna olubuto. Mu kusalawo okwetaba mu kunoonyereza, tugenda kukuteeka mu kimu ku bibinja nga bwe kinasalibwawo abakola ku kunoonyereza. Oyinza okweroboza ekika ky'akaweta akassibwa mu nnabaana ak'okukozesa oba aka copper T oba akavaamu ekirungo kya Levonorgestrel obussibwa mu nnabaana (IUDs) wabula oyina obutasalawo ku kiseera mwe kanateekebwamu okusinziira ku byetaago by'okunoonyereza kuno.

Oluvannyuma lw'okuwandiika ebyafaayo by'obujjanjabi bwo n'okukukebera obulungi, bw'osangibwa nga osaanidde okwetaba mu kunoonyereza kuno, tugenda kuteekamu akaweta mu nnabaana wo. Ogenda kugalamizibwa nga otunudde waggulu nga amagulu go nga gali ku byuma ebigawanirira waggulu. Ekyuma ekiyitibwa kijja kussibwa mu bitundu eby'ekyama era ekyuma (tenaculum) kijja kuteekebwa ku mumwa gwa nnabaana okugolola nnabaana. Ekyuma ekirala (uterine sound) kijja kussibwa mu nnabaana wo bwe kinalowoozebwa nga kisaanidde okupima obuvanvu bwe noluvannyuma lw'ekyo akaweta ke weerobozza kagenda kujja kussibwa mu nnabaana. Obuwuzi bujja kusalibwa ku buwanvu bwa ssentimita ssatu (3 centimeters). Ogenda

kusabibwa okulamba ku layini entereevu eri ku kipande ekiyitibwa VAS chart (layini ya ssentimita kkumi (10cm horizontal line)) okulaga obulumi nga tebannaba kussaamu kaweta, nga bateekamu ekyuma kya tenaculum n’ekya sound, okuteekamu akaweta ka IUC, era nga tonnaba kusiibulwa.

Ku kukyala okw’okulondoolwa, tujja kuwandiika ebyafaayo by’obujjanjabi bwo. Tujja kukukebera okwongereza ku kukebera okwa bulijjo, tugenda kwongerako okukebera n’ekyuma kya speculum okulaba oba akaweta akakussiddwamu kakyali mu kifo ekituufu. Tugenda kukubuuza ku ngeri gy’ovaamu omusaayi okuva lwe baateekamu akaweta, obulwadde bwonna obw’ebitundu eby’ekyama n’ekigero kyo eky’obumtivu ku kaweta akassibwa mu nnabaana ke weeroboza.

Ani agenda okwetaba mu kunoonyereza?

Ogenda kubuuzibwa ebibuuzo okufaanana n’abeetabyemu abalala 2082 okusalawo oba akaweta akassibwa mu nnabaana okuziyiza olubuto mu bbanga erya wiiki emu kakola nga bwe kassibwamu bulijjo ku wiiki 2-4 oluvannyuma lw’okufuna obujjanjabi bw’embuto ezitavuddemu bulungi mu myezi esatu egisooka egy’okufuna olubuto.

Obuzibu/Okutawaanyizibwa: Obuweta obussibwa mu nnabaana (IUDs) aka copper T n’akavaamu ekirungo kya Levonorgestrel bukozesebwa mu Uganda mu nkola ey’ekizaala gumba (140). Mu kukozesa obuweta obussibwa mu nnabaana, akaweta ka copper IUD, kasobola okukuleetera okulwa mu nsonga oba okulumizibwa nga oli mu nsonga. Waliwo obuzibu bw’okuvaamu omusaayi wakati mu nsonga zo. Wabula obubonero buno busobola okukendeera mu bbanga lya myezi mukaaga oba busobola okujjanjabwa n’eddagala nga n’eddagala eddala. Era waliwo omukisa gwa butundutundu munaana ku buli kikumi ogw’okufuna olubuto nga okozesa enkola eno. Mu kukoesa akaweta ka Levonorgestrel IUD, wayinza okubeerawo okukendeera mu mirundi gy’ogenda mu nsonga oba ebiseera ebimu obutalaba kugenda mu nsonga kwonna. Era waliwo obuzibu bwa katundutundu kamu ku buli kikumi obw’okufuna olubuto nga okozesa enkola eno. Newankubadde nga akaweta kakola mu ngeri eya bulijjo, ebiseera ebimu ekirungo kya Levonorgestrel kisobola okugenda mu musaayi gwo era kino kisobola okukyusa embeera yo. Tewali n’emu kun kola eya IUDs esobola okukuziyiza obutafuna kawuka ka siriimu (HIV) oba endwadde endala zonna ez’ekikaba naddala nga weetaba mu kikolwa eky’okwegatta nga tolina bukuumi.

Singa ofuna ekimu ku bino; okulwala okw'amaanyi oluvannyuma lw'okuggyamu ebibadde mu nnabaana, nga wetaaga okulongoosebwa (okusala mu lubuto okunoonyereza (exploratory laparotomy)) oluvannyuma lw'okuteekamu akaweta mu nnabaana nga omaze okuvaamu olubuto, nga wetaaga okussibwako eccupa y'omusaayi oluvannyuma lw'okuvaamu omusaayi okw'amaanyi, okuweebwa ekitanda okumala essaawa 72, okuyisibwa obubi okw'amaanyi okutali kwa bulijjo (anaphylactic reaction) oluvannyuma lw'okuteekamu akaweta ka IUD, oba okufa kwo oba okw'omwagalwa wo, bino bigenda kutwalibwa nga okuyisibwa obubi okw'amaanyi ennyo.Abeetabyemu abafunye okuyisibwa obubi bagenda kujjanjabibwa mangu abakola ku by'okujjanjaba abali ku malwaliro ag'enjawulo okusinziira ku nkola egobererwa bulijjo. Abo abetaaga okufuna obuweereza obw'okusindikibwa ewalala bagenda kuweebwa obujjanjabi obulungi nga busasulirwa abanoonyereza. Tugenda kukusaba otegeeze Omunoonyereza omukulu oba abakola ku kunoonyereza amangu ddala ng'ebintu ebyo bibaddewo wasobole okubeerawo ekikolebwa okutereeza embeera mu kiseera ekituufu.

Emiganyulo:

Abeetaba mu kunoonyereza abagenda kulondoolwa nnyo mu kujjanjabibwa kwabwe nga tebavuddemu bulungi lubuto mu myezi esatu egisooka egy'okufuna olubuto mu kugerageranya n'abo abafuna obujjanjabi obwa bulijjo. Bagenda kufuna okubudaabudibwa kw'okuziyiza okufuna olubuto nga bamaze okuvaamu olubuto. Okukozesa obuweta aka copper oba Levonorgestrel obussibwa mu nnabaana kikusobozesa obutafuna lubuto okumala ekiseera nga okukozesa obuweta. Obuweta bwa Levonorgestrel obussibwa mu nnabaana bukuyamba obutafuna kulwa mu nsonga oba okulumizibwa nga oli mu nsonga. Akaweta ka copper akassibwa mu nnabaana kakuyamba okulwawo okufuna olubuto okutuuka ku myaka kkumi ate akaweta ka Levonorgestrel akassibwa mu nnabaana kakuyamba okulwawo okufuna olubuto okutuuka ku myaka ettaano nga okozesa enkola eno. Mu ngeri y'okubaako kye tukola eri obulamu bw'abantu, okunoonyereza kuno kugenda kuyamba mu kukola enkola ey'okuziyiza olubuto ng'omaze okuvaamu olubuto oluvannyuma lw'emyezi esatu egisooka egy'okufuna olubuto nga embuto zivuddemu etaliiwo mu kiseera kino.

Tusuubira nti emiganyulo gy'okuziyiza okufuna olubuto nga lumaze okuvaamu oluvannyuma lw'emyezi esatu egisooka egy'okufuna olubuto nga embuto zivuddemu gisinga obuzibu obuyinza okuva mu kufuna embuto ezitagenderddwa n'ebizibu ebigenderako.

Abakola ku kunoonyereza bagenda kukunnyonnyola ku nkola eziriwo ez'ekizaala gumba, amaanyi gaazo, enkola yaazo n'emiganyulo gy'enkola eziriwo mu kugerageranya n'enkola endala.

Okukuuma ebyama:

Ebikwata ku mulwadde ebikuŋŋaanyizibwa bigenda kukuumibwa mu kompyuta ezirimu paasiwaadi. Ebikwata ku beetabyemu byonna (entambi z'amaloboozi, ebiwandiiko, kkopi ezissiddwa mu buwandiike n'obubaluwa) bigenda kuterekebwa mu kifo ekikuumibwa ekigenda okuggalwa era nga kituukibwamu abantu bokka abali obutereevu mu kunoonyereza. Abeetabye mu kunoonyereza bagenda kuyitbwa amannya agatali gaabwe mu lipooti esembayo. Abakola ku kunoonyereza bagenda kuteeka omukono ku biwandiiko by'okukuuma ebyama okukakasa nti banywerera ku kukuuma ebyama by'abetabyemu ekiseera kyonna mu kunoonyereza n'oluvannyuma lw'okunoonyereza.

Olw'okuba nga omunoonyereza omukulu n'Akakiiko akavunaanyizibwa ku by'Empisa mu kunoonyereza ak'omu kitundu (Research Ethics Committee (REC)) ne Uganda National Council for Science and Technology (UNCST) bagenda kulaba obubaka bwo obw'ekyma obukukwatako obwawula abeetabye mu kunoonyereza okusinziira ku linnya okukakasa nti okuumibwa obutafuna kabi okumalako ekiseera kyonna eky'okunoonyereza.

Okweroboza okulala:

Olina eddembe okugaana okwetaba mu kunoonyereza oba okukuvaamu ku kiseera kyonna eky'okubuuzibwa awatali kukosa mutindo gwa bujjanjabi obukuweebwa nga oli mu ddwaliro. Singa werobozako engeri endala mu kifo ky'obujjanjabi bw'olubuto olutavuddemu bulungi mu myezi esatu egisooka, ogenda kuweebwa engeri endala ey'okulongoosebwa baggyemu ebintu ebisigaddemu ebiva mu kufuna olubuto. Enkola endala ez'ekizaala gumba (140) nga oggyeeko ey'okussibwamu akaweta mu nnabaana akaziyiza olubuto nazo zijja kukuweebwa osalewo nga otegedde ku kisinga okukukolera.

Ebisale ebisasulwa:

Ebisale by'eddagala nga Misoprostol, eddagala eriziyiza okukula kw'obuwuka n'okubusaanyawo (antibiotics) n'eddagala erikendeeza obulumi mu kiseera eky'okujjanjabibwa n'obuweta obussibwa mu nnabaana bigenda kusasulwa abakola ku kunoonyereza. Obuzibu bwonna obutasuubirwa nga buva ku bikozesebwa mu kunoonyereza obuyinza okukutuukako mu kunoonyereza bugenda kusasulirwa abakola ku kunoonyereza.

Okuliyirirwa olw'okwetaba mu kunoonyereza:

Ogenda kuweebwa 30,000/= (siringisi za Uganda emitwalo esatu) nga okuliyirirwa olw'obudde bwo okwetaba mu kunoonyereza. Singa wabeera okukosebwa okw'olubeerera kwonna oba ebisago eby'amaanyi byonna nga okunoonyereza kugenda mu maaso, ebisale by'obujjanjabi bw'abeetaba mu kunoonyereza bigenda kusasulwa abakola ku kunoonyereza.

Okuddiza:

Abeetaba mu kunoonyereza bonna bagenda kuliyirirwa olw'obudde bwabwe era bagenda kuddizibwawo ssente z'entambula era emmere yabwe egenda kusasulwa mu kukyala kwabwe okw'okulondoolwa. Singa otuuka ku nkomerero y'okunoonyereza, tugenda kukuwa akasiimo ku kukyala kwo okw'okulondoolwa okusembayo nga Abanoonyereza abakulu basazeewo.

Ebibuuzo ebikwata ku kunoonyereza:

Ennamba ya nansi akola ku kunoonyereza ebeerako essaawa yonna egenda kuweebwa abeetaba mu kunoonyereza bonna okumala essaawa 24 buli wiiki okukola ku nsonga zonna ze bagenda okuba nazo nga bafuna obujjanjabi mu kunoonyereza oba mu kiseera ky'okunoonyereza. Kino kigenda kusobozesa okwanukula mu budde eri ensonga zabeetaba mu kunoonyereza. Essimu y'Omunoonyereza omukulu, n'eya ssentebe wa School of Medicine and Ethics committee n'omuwandiisi wa Uganda National Science and Technology committee nazo zissiddwa mu kiwandiiko kino eky'okukkiriza (ekiwa olukusa).

Ebibuuzo ebikwata ku ddembe ly'abeetaba mu kunoonyereza:

Singa wabeerawo ebibuuzo by'oyagala okubuuza ku kikwata ku bigobererwa mu kunoonyereza okulondoolwa, okwebuuza kwonna ku bikozesebwa mu kunoonyereza oba ensonga zonna nga oli mu kunoonyereza, osobola okukubira Dr. Herbert Kayiga essimu ye ewandiikiddwa wammanga.
Singa obeera n'ensonga ku mpisa ekirizibwa, obutali bumativu bwonna ku kunoonyereza, osabibwa okukubira ssentebe w'Akakiiko akakwasisa empisa mu kunoonyereza oba omuwandiisi wa National Council of Science and Technology nga ennamba ze zissiddwa wansi. Ennamba z'essimu ze zino:

4. **Dr. Herbert Kayiga**
Department of Obstetrics and Gynecology,
Makerere University College of Health Services,
P.O. Box 7062, Kampala.
0777855063.
Email: hkayiga@gmail.com

5. **Prof Ponsiano Ocama**

Ssentebe:

SOMREC (School of Medicine Research & Ethics Committee)

0772421190

Email: ponsiano.ocama@gmail.com

6. **Dr. Peter Ndemere**

Executive Secretary- UNCST

+256414705500

Email: info@uncst.go.ug

Okukakasa obwa nakyewa:

Olina eddembe okugaana okwetaba mu kunoonyereza oba okukuvaamu ku kiseera kyonna eky'okubuuzibwa awatali kukosa mutindo gwa bujjanjabi obukuweebwa nga oli mu ddwaliro.

Okufulumya ebivuddemu:

Ogenda kuweebwa obubaka obukwata ku ntambula y'okunoonyereza oba ebizuulibwa mu kunoonyereza. Obubaka bwonna obupya obukwata ku kunoonyereza oba ebikuŋŋaanyizibwa nga bikulu eri obujjanjabi bw'abeetaba mu kunoonyereza (nga otwaliddemu n'ebirala ebizuulibwa) bigenda kukuweebwa ne/oba abasawo bo.

Okukkirizibwa mu mateeka:

Okukkirizibwa mu mateeka okw'okunoonyereza kuno kwafunibwa okuva ku SOMREC, UNCST, n'ekitongole ky'Ebyobulamu ekya Uganda. Olukusa olw'okuddukanya emirimu nalwo lwafunibwa okuva eri abakulira amalwaliro ag'enjawulo agakola omulimu n'ekitongole kya Obstetrics and Gynaecology, Makerere University College of Health Sciences ekikola ku by'okuzaalisa n'endwadde z'abakyala. Okunoonyereza era kwawandiisibwa ku ClinicalTrials.gov ne kwanjulwa okusinziira ku ndagiriro ya CONSORT.

OKUKAKASA OKUKKIRIZA/OLUKUSA

.... annyinnyonnyodde ekigenda okukolebwa, obuzibu, emiganyulo egirimu n'eddembe lyange ku kikwata ku kunoonyereza kuno. Ntegeera nti okusalawo kwange okwetaba mu kunoonyereza kuno tekijja kukyusa bujjanjabi bwange oba obw'omwana wange bw'afuna bulijjo. Mu kukozesa obubaka buno, erinnya lyange terijja

kwatulwa. Nkimanyi nti nsobola okuvaamu ekiseera kyonna. Ntegeera nti okuyita mu kuteeka omukono ku kiwandiiko kino, seggyako ddembe n'erimu erimpeebwa mu mateeka wabula ndaga bulazi nti ntegeezeddwa ebikwata ku kunoonyereza kwenzikiriza kyeyagalire okwetabamu. Kopi y'ekiwandiiko kino egenda kumpeebwa.

Erinnya ………………Omukono/Ekinkumu ky'ayeetabyemu …………Ennaku z'omwezi ……

Erinnya …………………Omukono gw'omuzadde/omukuza w'abaana (bwe kiba kyetaagisa)…Ennaku z'omwezi …........................

Erinnya…………Omukono gw'omujulizi (bwe kiba kyetaagisa)………Ennaku z'omwezi…….

Erinnya ………………Omukono gw'omusoyisoyi/Omuntu afuna olukusa nga otegedde ……… Ennaku z'omwezi ………….

APPENDIX XIII: LUGANDA CONSENT FOR UPTAKE

MAKERERE UNIVERSITY COLLEGE OF HEALTH SCIENCES

SCHOOL OF MEDICINE RESEARCH AND ETHICS COMMITTEE (SOM-REC)

Omutwe gw'okunoonyereza okugenda okukolebwa:

UPTAKE AND FACTORS ASSOCIATED WITH UTILIZATION OF POST ABORTION INTRAUTERINE CONTRACEPTION AFTER FIRST TRIMESTER MEDICAL MANAGEMENT OF INCOMPLETE ABORTION IN CENTRAL UGANDA

Omunoonyereza:

DR. HERBERT KAYIGA,
Department of Obstetrics and Gynecology,
Makerere University College of Health Services,
P.O. Box 7062, Kampala.
0777855063. Email: hkayiga@gmail.com
Abakolera awamu n'omunoonyereza:
ASSOC. PROF JOSAPHAT BYAMUGISHA,
Makerere University College of Health Sciences,
Department of Obs/Gyn,
P.O. Box 7062, Kampala.
0772580330. Email: jbyamugisha@gmail.com
DR. OTHMAN KAKAIRE
Makerere University College of Health Sciences,
Department of Obs/Gyn,
P.O. Box 7062, Kampala.
0772514616, Email: kakaireothman@gmail.com
ASSOC. PROF NAZARIUS .M. TUMWESIGYE
Makerere University College of Health Sciences,
Department of Epidemiology and Biostatistics
P.O. Box 7062, Kampala.
0782447771, Email: naz@musph.ac.ug
PROF KRISTINA GEMZELL-DANIELSSON
Karolinska Institutet and Karolinska University Hospital
Email: kristina.gemzell@ki.se

Ebyafaayo n'ensonga ezikwata ku kunoonyereza:

Oluvannyuma lw'okuvaamu embuto mu myezi esatu egisooka, obusobozi bw'okufuna olubuto bukomawo mu bbanga lya wiiki bbiri. Eby'embi abakyala abasinga obungi tebakomawo ku malwaliro kufuna nkola ey'okuziyiza okufuna olubuto nga olubuto lumaze okuvaamu oluvannyuma lw'ebyo. Kino kiviirako bangi ku bakyala bano okufuna embuto ze batagenderedde era ze batayagala era oluvannyuma ekyo kiyinza okuvaamu ebizibu ku lubuto. Mu Uganda, enkola ey'okuziyiza okufuna olubuto nga olubuto lumaze okuvaamu weeri wonna naye enkozesa esigadde nga ekyali wansi nnyo ku butundutundu 0.5%. Ensonga lwaki enkozesa eri wansi n'ebiviirako enkozesa y'enkola ey'okuziyiza olubuto okuba wansi tennaba kunoonyerezebwako.

Okunoonyereza kuno kunoonya okuzuula omuwendo gw'abakazi abasalawo okukozesa enkola ey'okuziyiza okufuna olubuto nga olubuto lumaze okuvaamu mu kugerageranya n'omugatte gw'abakazi bonna abafuna empeereza z'obujjanjabi nga olubuto lumaze okuvaamu mu kiseera ky'okunoonyereza mu bifo kkumi na bitaano (15) eby'okunoonyereza. Abakazi ng'abo bagenda kubeeramu abo abajjanjabiddwa n'amakerenda ga Misoprostol okumalamu ebintu ebivudde mu

kufuna olubuto oluvannyuma lw'emyezi esatu egisooka nga olubuto teruvuddemu bulungi. Okunoonyereza kuno era kunoonya okuzuula ebiremesa n'ebiyambako okusalawo okukozesa enkola ey'okuziyiza okufuna olubuto ey'okussibwamu akaweta mu nnabaana nga olubuto lumaze okuvaamu okumanyisa obulungi abateesiteesi b'enkola ku ngeri y'okukola endagiriro nga besigama ku bujulizi bwennyini obukwata ku mulamwa ku nkola ey'okuziyiza okufuna olubuto nga olubuto lumaze okuvaamu.

Ebikwata ku bawagira omulimu gw'okunoonyereza n'ebitongole ebikolagana n'abanoonyereza:

Nze Dr. Herbert Kayiga, nkola ku by'Okuzaalisa/endwadde z'abakyala (Obstetrician/Gynaecologist), era ndi Musomesa mu kitongole ky'Ebyokuzaalisa n'Endwadde z'abakyala (Obstetrics and Gynaecology), ekya Makerere University College of Health Sciences, omunoonyereza mu kunoonyereza okukulu "*Okukkirizika, n'Enkola n'Enkozesa y'Akaweta akaziyiza olubuto mu nnabaana nga bukyali oluvannyuma lw'okuvaamu olubuto mu kugerageranya n'enkozesa y'akaweta akaziyiza olubuto mu nnabaana eya bulijjo oluvannyuma lw'okufuna obujjanjabi bw'embuto ezitavuddemu bulungi mu myezi esatu egisooka egy'okufuna olubuto mu bitundu bya Uganda eya Wakati" ("Acceptability, Effectiveness and Uptake of Early versus Standard Post Abortion Intrauterine Contraception after First Trimester Medical Post Abortion Care for Incomplete Abortions in Central Uganda")*. Associate Professor Josaphat Byamugisha, Associate Professor Nazarius Tumwesigye, ne Dr. Othman Kakaire nabo bakolaganira wamu ne Makerere University College of Health Sciences. Bebannuŋŋamya ku mulimu guno ogw'okunoonyereza. Professor Kristina Gemzell-Danielsson, akolera wamu nange mu kunoonyereza ng'asinziira ku kitongole kya Karolinska Institutet ne Karolinska University Hospital, Sweden.

Okunoonyereza kuno kugenda kukolebwa mu bifo ewajjanjabirwa kkumi na bitaano (15) ebigenda okubeeramu eddwaliro lya Entebbe hospital, ne Mpigi HC IV, ne Masaka hospital, ne Gombe hospital, ne Nakaseke hospital, ne Luweero HC IV, ne Mengo hospital, ne Naguru hospital, ne Kawempe National referral hospital, ne Kawolo hospital, ne Wakiso HC IV, ne Kasangati HC IV, ne Kiganda Health Centre IV ne Kayunga hospital.

Okunoonyereza kugenda kussibwamu ekitundu ku ssente okuyita mu nkolagana wakati wa Makerere University ne Karolinska Institutet ne Karolinska University Hospital okuyita mu Prof Kristina Gemzell-Danielsson. Makerere University esazeeko ku bisale by'essomero ate Karolinska Institutet, egenda kuwaayo ezimu ku ssente z'okunoonyereza.

Ekigendererwa:

Mu kunoonyereza kuno, tugenda kukubuuza ebibuuzo ebikwata ku kusalawo kwo okw'akaweta akassibwa mu nnabaana ak'okukozesa okuziyiza olubuto lw'otayagala oluvannyuma lw'okuvaamu olubuto lw'obadde nalwo nga waweebwa amakerenda ga Misoprostol ogateeke wansi w'olulimi kikusobozese okufulumya ebintu ebyali bitavuddeeyo bulungi. Era tunoonye okutegeera lwaki wasazeewo okukozessa akaweta akassibwa mu nnabaana okusinga enkola endala ez'okuziyiza okufuna olubuto era oba nga owagira mukwano gwo akozese enkola ey'ekika kino okuziyiza olubuto mu kiseera eky'omu maaso. Okunoonyereza kuno era kunoonya okuzuula ebiremesa n'ebiyambako okusalawo okukozesa enkola ey'okuziyiza okufuna olubuto ey'okussibwamu akaweta mu nnabaana nga olubuto lumaze okuvaamu okumanyisa obulungi abateesiteesi b'enkola ku ngeri y'okukola endagiriro nga besigama ku bujulizi bwennyini obukwata ku mulamwa ku nkola ey'okuziyiza okufuna olubuto nga olubuto lumaze okuvaamu.

Ekiseera ekiteeberezebwa ayeetabye mu kunoonyereza ky'ajja okutwala ng'ali mu kunoonyereza:

Tuteekateeka okutwala eddakiika nga 30-45 mu bibuuzo ebibuuzibwa.

Engeri akaweta gye kakolamu:

Olina okweroboza ku kika ky'akaweta akassibwa mu nnabaana k'oyagala bakuteekemu. Akaweta aka copper IUD akassibwa mu nnabaana kasobola okukuyamba obutafuna lubuto okumala emyaka nga kkumi. Ekika kino kyennyini eky'akaweta akassibwa mu nnabaana kikola nga kireetera embeera eri mu nnabaana okuba nga tesobozesa kufuna lubuto nga kivaamu ekirungo ekiyitibwa copper ions. Copper ions kye zikola kisobola okukuleetera okulwa mu nsonga, okusinga ku kulumizibwa mu lubuto okwa bulijjo nga oli mu nsonga. Bulijjo obubonero buno busobola okukendeera mu bbanga lya myezi mukaaga. Enkola eno ekolerawo mangu oluvannyuma lw'okugiteekamu nga omaze okufuna obujjanjabi obw'olubuto olutavuddemu bulungi mu myezi esatu egisooka egy'okufuna olubuto.

Akaweta aka Levonorgestrel akassibwa mu nnabaana kasobla okukuyamba okulwawo okufuna olubuto okumala emyaka ettaano nga kamaze okussibwamu. Kakola nga kayita mu kufulumya ekirungo ekitonotono ekya Levonorgestrel ekiziyiza obusobozi bwo obw'okufulumya eggi erisobola okuwakisibwa. Enkola eno era ereetera amazzi go agabeera ku mumwa gwa nnabaana okubeera amakwafu ng'eminyira era mu kukola bwe gatyo, gaziyiza amazzi g'omusajja okuyitamu.

Ebigobererwa mu kunoonyereza:

Ogenda kubuuzibwa ebibuuzo ebikwata ku nsonga eziri emabega w'okusalawo kwo era oba nga owagira mikwano gyo okukozesa enkola eno oba n'okuddamu okukozesa enkola eno mu kiseera eky'omu maaso. Tugenda kukozesa obubaka bw'otuwa okuzuula ekiseera ekituufu eky'okuteekamu obuweta mu nnabaana oluvannyuma lw'obutavaamu bulungi lubuto mu wiiki 12 ezisooka okuziyiza embuto ezitayagalwa era ezitagendereddwa mu kiseera eky'omu maaso. Kino kiri bwe kiti kubanga obusobzi bw'okufuna olubuto olulala bukomawo mu bbanga lya wiiki bbiri oluvannyuma lw'okuggyamu ebiva mu kufuna olubuto.

Ani agenda okwetaba mu kunoonyereza?

Era tuteekateeka okubuuza abakyala abalala 738 abalinga ggwe ku nsonga eziri emabega w'okusalawo kwo era oba nga owagira mikwano gyo bakozese enkola eno oba n'okuddamu okukozesa enkola eno mu kiseera eky'omu maaso. Tugenda kukozesa obubaka bw'otuwa okutegeera ebiremesa n'ebiyambako ku kusalawo kwo okuteekebwamu obuweta mu nnabaana oluvannyuma lw'okuvaamu olubuto mu wiiki 12 ezisooka okuziyiza embuto ezitayagalwa era ezitagendereddwa.

Obuzibu/Okutawaanyizibwa: Obuweta obussibwa mu nnabaana (IUDs) aka copper T n'akavaamu ekirungo kya Levonorgestrel bukozesebwa mu Uganda mu nkola ey'ekizaala gumba (140). Mu kukozesa obuweta obussibwa mu nnabaana, akaweta ka copper IUD, kasobola okukuleetera okulwa mu nsonga oba okulumizibwa nga oli mu nsonga. Waliwo obuzibu bw'okuvaamu omusaayi wakati mu nsonga zo. Wabula obubonero buno busobola okukendeera mu bbanga lya myezi mukaaga oba busobola okujjanjabwa n'eddagala nga n'eddagala eddala. Era waliwo omukisa gwa butundutundu munaana ku buli kikumi ogw'okufuna olubuto nga okozesa enkola eno. Mu kukoesa akaweta ka Levonorgestrel IUD, wayinza okubeerawo okukendeera mu

mirundi gy'ogenda mu nsonga oba ebiseera ebimu obutalaba kugenda mu nsonga kwonna. Era waliwo obuzibu bwa katundutundu kamu ku buli kikumi obw'okufuna olubuto nga okozesa enkola eno. Newankubadde nga akaweta kakola mu ngeri eya bulijjo, ebiseera ebimu ekirungo kya Levonorgestrel kisobola okugenda mu musaayi gwo era kino kisobola okukyusa embeera yo. Tewali n'emu kun kola eya IUDs esobola okukuziyiza obutafuna kawuka ka siriimu (HIV) oba endwadde endala zonna ez'ekikaba naddala nga weetaba mu kikolwa eky'okwegatta nga tolina bukuumi.

Singa ofuna ekimu ku bino; okulwala okw'amaanyi oluvannyuma lw'okuggyamu ebibadde mu nnabaana, nga wetaaga okulongoosebwa (okusala mu lubuto okunoonyereza (exploratory laparotomy)) oluvannyuma lw'okuteekamu akaweta mu nnabaana nga omaze okuvaamu olubuto, nga wetaaga okussibwako eccupa y'omusaayi oluvannyuma lw'okuvaamu omusaayi okw'amaanyi, okuweebwa ekitanda okumala essaawa 72, okuyisibwa obubi okw'amaanyi okutali kwa bulijjo (anaphylactic reaction) oluvannyuma lw'okuteekamu akaweta ka IUD, oba okufa kwo oba okw'omwagalwa wo, bino bigenda kutwalibwa nga okuyisibwa obubi okw'amaanyi ennyo.Abeetabyemu abafunye okuyisibwa obubi bagenda kujjanjabibwa mangu abakola ku by'okujjanjaba abali ku malwaliro ag'enjawulo okusinziira ku nkola egobererwa bulijjo. Abo abetaaga okufuna obuweereza obw'okusindikibwa ewalala bagenda kuweebwa obujjanjabi obulungi nga busasulirwa abanoonyereza. Tugenda kukusaba otegeeze Omunoonyereza omukulu oba abakola ku kunoonyereza amangu ddala ng'ebintu ebyo bibaddewo wasobole okubeerawo ekikolebwa okutereeza embeera mu kiseera ekituufu.

Emiganyulo:

Abeetaba mu kunoonyereza abagenda kulondoolwa nnyo mu kujjanjabibwa kwabwe nga tebavuddemu bulungi lubuto mu myezi esatu egisooka egy'okufuna olubuto mu kugerageranya n'abo abafuna obujjanjabi obwa bulijjo. Enkola eno egenda kuziyiza embuto ezitagendereddwa ekigasa abeetabyemu. Mu ngeri y'okubaako kye tukola eri obulamu bw'abantu, okunoonyereza kuno kugenda kuyamba mu kukola enkola ey'okuziyiza olubuto ng'omaze okuvaamu olubuto oluvannyuma lw'emyezi esatu egisooka egy'okufuna olubuto nga embuto zivuddemu etaliiwo mu kiseera kino.

Tusuubira nti emiganyulo gy'okuziyiza okufuna olubuto nga lumaze okuvaamu oluvannyuma lw'emyezi esatu egisooka egy'okufuna olubuto nga embuto zivuddemu gisinga obuzibu obuyinza okuva mu kufuna embuto ezitagenderddwa n'ebizibu ebigenderako.

Abakola ku kunoonyereza bagenda kukunnyonnyola ku nkola eziriwo ez'ekizaala gumba, amaanyi gaazo, enkola yaazo n'emiganyulo gy'enkola eziriwo mu kugerageranya n'enkola endala.

Okukuuma ebyama:

Ebikwata ku mulwadde ebikuŋŋaanyizibwa bigenda kukuumibwa mu kompyuta ezirimu paasiwaadi. Ebikwata ku beetabyemu byonna (entambi z'amaloboozi, ebiwandiiko, kkopi ezissiddwa mu buwandiike n'obubaluwa) bigenda kuterekebwa mu kifo ekikuumibwa ekigenda okuggalwa era nga kituukibwamu abantu bokka abali obutereevu mu kunoonyereza. Abeetabye mu kunoonyereza bagenda kuyitbwa amannya agatali gaabwe mu lipooti esembayo. Abakola ku kunoonyereza bagenda kuteeka omukono ku biwandiiko by'okukuuma ebyama okukakasa nti banywerera ku kukuuma ebyama by'abetabyemu ekiseera kyonna mu kunoonyereza n'oluvannyuma lw'okunoonyereza.

Olw'okuba nga omunoonyereza omukulu n'Akakiiko akavunaanyizibwa ku by'Empisa mu kunoonyereza ak'omu kitundu (Research Ethics Committee (REC)) ne Uganda National Council for Science and Technology (UNCST) bagenda kulaba obubaka bwo obw'ekyma obukukwatako obwawula abeetabye mu kunoonyereza okusinziira ku linnya okukakasa nti okuumibwa obutafuna kabi okumalako ekiseera kyonna eky'okunoonyereza.

Okweroboza okulala:

Olina eddembe okugaana okwetaba mu kunoonyereza oba okukuvaamu ku kiseera kyonna eky'okubuuzibwa awatali kukosa mutindo gwa bujjanjabi obukuweebwa nga oli mu ddwaliro. Singa werobozako engeri endala mu kifo ky'obujjanjabi bw'olubuto olutavuddemu bulungi mu myezi esatu egisooka, ogenda kuweebwa engeri endala ey'okulongoosebwa baggyemu ebintu ebisigaddemu ebiva mu kufuna olubuto. Enkola endala ez'ekizaala gumba (140) nga oggyeeko ey'okussibwamu akaweta mu nnabaana akaziyiza olubuto nazo zijja kukuweebwa osalewo nga otegedde ku kisinga okukukolera.

Ebisale ebisasulwa:

Ebisale by'eddagala nga Misoprostol, eddagala eriziyiza okukula kw'obuwuka n'okubusaanyawo (antibiotics) n'eddagala erikendeeza obulumi mu kiseera eky'okujjanjabibwa n'obuweta obussibwa mu nnabaana bigenda kusasulwa abakola ku kunoonyereza. Obuzibu bwonna

obutasuubirwa nga buva ku bikozesebwa mu kunoonyereza obuyinza okukutuukako mu kunoonyereza bugenda kusasulirwa abakola ku kunoonyereza.

Okuliyirirwa olw'okwetaba mu kunoonyereza:

Ogenda kuweebwa 30,000/= (siringisi za Uganda emitwalo esatu) nga okuliyirirwa olw'obudde bwo okwetaba mu kunoonyereza. Singa wabeera okukosebwa okw'olubeerera kwonna oba ebisago eby'amaanyi byonna nga okunoonyereza kugenda mu maaso, ebisale by'obujjanjabi bw'abeetaba mu kunoonyereza bigenda kusasulwa abakola ku kunoonyereza.

Okuddiza:

Abeetaba mu kunoonyereza bonna bagenda kuliyirirwa olw'obudde bwabwe era bagenda kuddizibwawo ssente z'entambula.

Ebibuuzo ebikwata ku kunoonyereza:

Ennamba ya nansi akola ku kunoonyereza ebeerako essaawa yonna egenda kuweebwa abeetaba mu kunoonyereza bonna okumala essaawa 24 buli wiiki okukola ku nsonga zonna ze bagenda okuba nazo nga bafuna obujjanjabi mu kunoonyereza oba mu kiseera ky'okunoonyereza. Kino kigenda kusobozesa okwanukula mu budde eri ensonga zabeetaba mu kunoonyereza. Essimu y'Omunoonyereza omukulu, n'eya ssentebe wa School of Medicine and Ethics committee n'omuwandiisi wa Uganda National Science and Technology committee nazo zissiddwa mu kiwandiiko kino eky'okukkiriza (ekiwa olukusa).

Ebibuuzo ebikwata ku ddembe ly'abeetaba mu kunoonyereza:

Singa wabeerawo ebibuuzo by'oyagala okubuuza ku kikwata ku bigobererwa mu kunoonyereza, okwebuuza kwonna ku bikozesebwa mu kunoonyereza oba ensonga zonna nga oli mu kunoonyereza, osobola okukubira Dr. Herbert Kayiga essimu ye ewandiikiddwa wammanga. Singa obeera n'ensonga ku mpisa ekirizibwa, obutali bumativu bwonna ku kunoonyereza, osabibwa okukubira ssentebe w'Akakiiko akakwasisa empisa mu kunoonyereza oba omuwandiisi wa National Council of Science and Technology nga ennamba ze zissiddwa wansi. Ennamba z'essimu ze zino:

4. **Dr. Herbert Kayiga**

Department of Obstetrics and Gynecology,

Makerere University College of Health Services,

P.O. Box 7062, Kampala.

0777855063. Email: hkayiga@gmail.com

5. **Prof Ponsiano Ocama**

Chairperson:

SOMREC (School of Medicine Research & Ethics Committee)

0772421190

Email: ponsiano.ocama@gmail.com

6. **Dr. Peter Ndemere**

Executive Secretary- UNCST

+256414705500

Email: info@uncst.go.ug

Okukakasa obwa nnakyewa:

Olina eddembe okugaana okwetaba mu kunoonyereza oba okukuvaamu ku kiseera kyonna eky'okubuuzibwa awatali kukosa mutindo gwa bujjanjabi obukuweebwa nga oli mu ddwaliro.

Okufulumya ebivuddemu:

Ogenda kuweebwa obubaka obukwata ku ntambula y'okunoonyereza oba ebizuulibwa mu kunoonyereza. Obubaka bwonna obupya obukwata ku kunoonyereza oba ebikuŋŋaanyizibwa nga bikulu eri obujjanjabi bw'abeetaba mu kunoonyereza (nga otwaliddemu n'ebirala ebizuulibwa) bigenda kukuweebwa ne/oba abasawo bo.

Okukkirizibwa mu mateeka:

Okukkirizibwa mu mateeka okw'okunoonyereza kuno kwafunibwa okuva ku SOMREC, UNCST, n'ekitongole ky'Ebyobulamu ekya Uganda. Olukusa olw'okuddukanya emirimu nalwo lwafunibwa okuva eri abakulira amalwaliro ag'enjawulo agakola omulimu n'ekitongole kya Obstetrics and Gynaecology, Makerere University College of Health Sciences ekikola ku by'okuzaalisa n'endwadde z'abakyala.

OKUKAKASA OKUKKIRIZA/OLUKUSA

.... annyinnyonnyodde ekigenda okukolebwa, obuzibu, emiganyulo egirimu n'eddembe lyange ku kikwata ku kunoonyereza kuno. Ntegeera nti okusalawo kwange okwetaba mu kunoonyereza kuno tekijja kukyusa bujjanjabi bwange oba obw'omwana wange bw'afuna bulijjo. Mu kukozesa obubaka buno, erinnya lyange terijja kwatulwa. Nkimanyi nti nsobola okuvaamu ekiseera kyonna. Ntegeera nti okuyita mu kuteeka omukono ku kiwandiiko kino, seggyako ddembe n'erimu erimpeebwa mu mateeka wabula ndaga

bulazi nti ntegeezeddwa ebikwata ku kunoonyereza kwenzikiriza kyeyagalire okwetabamu. Kopi y'ekiwandiiko kino egenda kumpeebwa.

Erinnya ………………Omukono/Ekinkumu ky'ayeetabyemu …………Ennaku z'omwezi ……

Erinnya …………………Omukono gw'omuzadde/omukuza w'abaana (bwe kiba kyetaagisa)…Ennaku z'omwezi …........................

Erinnya…………Omukono gw'omujulizi (bwe kiba kyetaagisa)………Ennaku z'omwezi…….

Erinnya ………………Omukono gw'omusoyisoyi/Omuntu afuna olukusa nga otegedde ……… Ennaku z'omwezi ………….

APPENDIX XIV: LUGANDA CONSENT FOR HEALTHCARE PROVIDERS

EKIWANDIIKO KY'OKUKKIRIZA (EKIWA OLUKUSA) EKY'ABASAWO
MAKERERE UNIVERSITY COLLEGE OF HEALTH SCIENCES

SCHOOL OF MEDICINE RESEARCH AND ETHICS COMMITTEE (SOM-REC)

Omutwe gw'okunoonyereza okugenda okukolebwa:

"HEALTHCARE PROVIDERS' PERCEPTIONS TOWARDS POST ABORTION INTRAUTERINE CONTRACEPTION AFTER FIRST TRIMESTER MEDICAL MANAGEMENT OF INCOMPLETE ABORTION IN UGANDA: A QUALITATIVE STUDY"

Omunoonyereza:

DR. HERBERT KAYIGA,
Department of Obstetrics and Gynecology,

Makerere University College of Health Services,
P.O. Box 7062, Kampala.
0777855063. Email: hkayiga@gmail.com
Abakolera awamu n'omunoonyereza:
ASSOC. PROF JOSAPHAT BYAMUGISHA,
Makerere University College of Health Sciences,
Department of Obs/Gyn,
P.O. Box 7062, Kampala.
0772580330. Email: jbyamugisha@gmail.com
DR. OTHMAN KAKAIRE
Makerere University College of Health Sciences,
Department of Obs/Gyn,
P.O. Box 7062, Kampala.
0772514616, Email: kakaireothman@gmail.com
ASSOC. PROF NAZARIUS .M. TUMWESIGYE
Makerere University College of Health Sciences,
Department of Epidemiology and Biostatistics
P.O. Box 7062, Kampala.
0782447771, Email: naz@musph.ac.ug
PROF KRISTINA GEMZELL-DANIELSSON
Karolinska Institutet and Karolinska University Hospital
Email: kristina.gemzell@ki.se

Ebyafaayo n'ensonga ezikwata ku kunoonyereza:

Okunoonyereza kuno kunoonya okuzuula ekiseera ekirungi eky'okussibwamu akaweta akaziyiza olubuto mu nnabaana oluvannyuma lw'okufuna obujjanjabi bw'olubuto olutavuddemu bulungi mu myezi esatu egisooka egy'okufuna olubuto mu bitundu bya Uganda eya Wakati. Mu kiseera kino enkola ebadde yakuteekamu kaweta akaziyiza olubuto mu nnabaana (akavaamu ekirungo kya Copper oba Levonorgestrel) wakati wa wiiki 2-4 oluvannyuma lw'okuggibwamu kw'ebiva mu kufuna olubuto. Ebyembi, abakazi bangi tebatera kukomawo kussibwamu buweta olw'ensonga eziwerako. Kino kibateeka mu kabi ak'okufuna embuto ezitali ŋŋenderere kubanga obusobozi bw'okufuna olubuto bukomawo mangu mu nnaku nga ttaano ku kkumi oluvannyuma lw'okuvaamu embuto.

N'olw'ekyo kye tuva tusaba okukubuuza ebikwata ku ndowooza yo nga omusawo ku kuteekamu obuweta obussibwa mu nnabaana nga engeri y'okuziyiza embuto ezitayagalwa oluvannyuma lw'okufuna obujjanjabi bw'olubuto olutavuddeemu bulungi mu myezi esatu egisooka. Ebirowoozo byo bigenda kutuyamba mu kukola endagiriro nga twesigama ku bujulizi ku kikwata

ku kuziyiza okufuna olubuton oluvannyuma lw'okuvaamu olubuto n'okusalawo ekiseera ekituufu eky'okuteekamu obuweta mu nnabaana oluvannyuma lw'okufuna obujjanjabi bw'embuto ezitavuddemu bulungi mu myezi esatu egisooka. Era tunoonya okutegeera ebiremesa n'ebyanguya mu basawo ku kikwata ku kuwagira okuziyiza okufuna olubuto oluvannyuma nga olubuto luvuddemu naddala obuweta obussibwa mu nnabaana.

Ebikwata ku bawagira omulimu gw'okunoonyereza n'ebitongole ebikolagana n'abanoonyereza:

Nze Dr. Herbert Kayiga, nkola ku by'Okuzaalisa/endwadde z'abakyala (Obstetrician/Gynaecologist), era ndi Musomesa mu kitongole ky'Ebyokuzaalisa n'Endwadde z'abakyala (Obstetrics and Gynaecology), ekya Makerere University College of Health Sciences, omunoonyereza mu kunoonyereza okukulu "*Okukkirizika, n'Enkola n'Enkozesa y'Akaweta akaziyiza olubuto mu nnabaana nga bukyali oluvannyuma lw'okuvaamu olubuto mu kugerageranya n'enkozesa y'akaweta akaziyiza olubuto mu nnabaana eya bulijjo oluvannyuma lw'okufuna obujjanjabi bw'embuto ezitavuddemu bulungi mu myezi esatu egisooka egy'okufuna olubuto mu bitundu bya Uganda eya Wakati" (*"*Acceptability, Effectiveness and Uptake of Early versus Standard Post Abortion Intrauterine Contraception after First Trimester Medical Post Abortion Care for Incomplete Abortions in Central Uganda")*. Associate Professor Josaphat Byamugisha, Associate Professor Nazarius Tumwesigye, ne Dr. Othman Kakaire nabo bakolaganira wamu ne Makerere University College of Health Sciences. Bebannuŋŋamya ku mulimu guno ogw'okunoonyereza. Professor Kristina Gemzell-Danielsson, akolera wamu nange mu kunoonyereza ng'asinziira ku kitongole kya Karolinska Institutet ne Karolinska University Hospital, Sweden.

Okunoonyereza kuno kugenda kukolebwa mu ddwaliro lya Entebbe hospital, ne Mpigi HC IV, ne Masaka hospital, ne Gombe hospital, ne Nakaseke hospital, ne Luweero HC IV, ne Mengo hospital, ne Naguru hospital, ne Kawempe National referral hospital, ne Kawolo hospital, ne Wakiso HC IV, ne Kasangati HC IV, ne Kiganda Health Centre IV ne Kayunga hospital.

Okunoonyereza kugenda kussibwamu ekitundu ku ssente okuyita mu nkolagana wamzell-Danielsson. Makerere University esazeeko ku bisale by'essomero ate Karolinska Institutet, egenda kuwaayo ezimu ku ssente z'okunoonyereza.

Ekigendererwa:

Mu kunoonyereza kuno, tugenda kukubuuza ebibuuzo ebikwata ku ndowooza yo ku eky'okussibwamu akaweta akaziyiza olubuto mu nnabaana nga olubuto lumaze okuvaamu oluvannyuma lw'okufuna obujjanjabi nga olubuto teruvuddemu bulungi. Ebirowoozo byo bigenda kutuyamba okumanya oba okussaamu akaweta nga bukyali (mu bbanga lya wiiki emu) kikola bulungi nga enkola eya bulijjo ey'okuteekamu akaweta (wakati wa wiiki 2-4 oluvannyuma lw'okufuna obujjanjabi bw'olubuto olutavuddemu bulungi mu myezi esatu egisooka). Bwe kikakasibwa okuba nga kikola bulungi, olwo tugenda kuziyiza embuto ezitayagalwa mu bakyala abavuddemu embuto mu myezi esatu egisooka kubanga obusobozi bw'okuddamu okufuna olubuto bubaawo mu bbanga lya wiiki bbiri. Ebirowoozo byo ku kuziyiza okufuna olubuto nga olubuto lumaze okuvaamu bigenda kutusobozesa okuzuula ebiremesa n'ebiyambako mu basawo ku kuziyiza okufuna olubuto nga olubuto lumaze okuvaamu bwe kityo tuluŋŋamizibwe mu kukola endagiriro nga twesigama ku bujulizi bwennyini obukwata ku mulamwa ku nkola ey'akaweta akassibwa mu nnabaana okuziyiza okufuna olubuto nga olubuto lumaze okuvaamu.

Ekiseera ekiteeberezebwa ayeetabye mu kunoonyereza ky'ajja okutwala ng'ali mu kunoonyereza:

Ogenda kubuuzibwa wekka okufaanana n'abasawo abalala 20-30 abalinga ggwe newankubadde nga bayinza okuba ab'ebibinja eby'enjawulo. Tuteekateeka okutwala eddakiika nga 45-90 mu kiseera eky'okubuuzibwa.

Ebigobererwa mu kunoonyereza:

Ogenda kubuuzibwa ku biremesa n'ebiyambako ku nkola ey'okussibwa akaweta mu nnabaana okuziyiza okufuna olubuto nga olubuto lumaze okuvaamu oluvannyuma lw'okufuna obujjanjabi bw'olubuto olutavuddemu bulungi mu myezi esatu egisooka.

Ani agenda okwetaba mu kunoonyereza?

Tuteekateeka okubuuza abasawo abawa obuweereza obwekizaala gumba (140) ku biremesa n'ebiyambako ku nkola ey'okussibwa akaweta mu nnabaana okuziyiza okufuna olubuto nga olubuto lumaze okuvaamu oluvannyuma lw'okufuna obujjanjabi bw'olubuto olutavuddemu bulungi mu myezi esatu egisooka. Tubuuza abasawo 20-30 ab'ebibinja eby'enjawulo nga tweyambisa enkola ey'okubuuzibwa ekigenda ewala okumala eddakiika nga 45-90.

Obuzibu/Okutawaanyizibwa: Tusuubira obuzibu butono gy'oli mu kunoonyereza kuno.

Emiganyulo:

Tewaliiwo ky'oganyulwamu butereevu naye obubaka obukuŋŋaanyizibwa bugenda kutuyamba okuwa amagezi nga tutegedde ku kiseera eky'okuteekamu obuweta obuweta mu nnabaana obuziyiza okufuna olubuto nga embuto zimaze okuvaamu mu wiiki 12 ezisooka naddala oluvannyuma lw'okukozesa obujjanjabi okumaliriza okuggibwamu kw'ebintu ebiva mu kufuna olubuto.

Okukuuma ebyama:

Ebikwata ku beetabyemu ebikuŋŋaanyizibwa bigenda kukuumibwa mu kompyuta ezirimu paasiwaadi. Ebikwata ku beetabyemu byonna (entambi z'amaloboozi, ebiwandiiko, kkopi ezissiddwa mu buwandiike n'obubaluwa) bigenda kuterekebwa mu kifo ekikuumibwa ekigenda okuggalwa era nga kituukibwamu abantu bokka abali obutereevu mu kunoonyereza. Abeetabye mu kunoonyereza bagenda kuyitbwa amannya agatali gaabwe mu lipooti esembayo. Abakola ku kunoonyereza bagenda kuteeka omukono ku biwandiiko by'okukuuma ebyama okukakasa nti banywerera ku kukuuma ebyama by'abetabyemu ekiseera kyonna mu kunoonyereza n'oluvannyuma lw'okunoonyereza.

Olw'okuba nga omunoonyereza omukulu n'Akakiiko akavunaanyizibwa ku by'Empisa mu kunoonyereza ak'omu kitundu (Research Ethics Committee (REC)) ne Uganda National Council for Science and Technology (UNCST) bagenda kulaba obubaka bwo obw'ekyma obukukwatako obwawula abeetabye mu kunoonyereza okusinziira ku linnya okukakasa nti okuumibwa obutafuna kabi okumalako ekiseera kyonna eky'okunoonyereza.

Okweroboza okulala:

Olina eddembe okugaana okwetaba mu kunoonyereza oba okukuvaamu ku mutendera gwonna ogw'okubuuzibwa awatali kukosa mbeera ya mulimu gwo ku ddwaliro oba n'abakola ku kunoonyereza.

Ebisale ebisasulwa:

Waliwo ebisasulwa obutereevu okukuyingiza mu kunoonyereza kuno naye singa bibeerawo mu ngeri yonna, abakola ku kunoonyereza bagenda kubisasula.

Okuliyirirwa olw'okwetaba mu kunoonyereza:

Ogenda kuweebwa 30,000/= (siringisi emitwalo esatu) nga okuliyirirwa olw'obudde bwo okwetaba mu kunoonyereza.

Okuddiza:

Abeetabye mu kunoonyereza bagenda kuliyirirwa olw'obudde bwabwe.

Ebibuuzo ebikwata ku kunoonyereza:

Essimu y'Omunoonyereza omukulu, n'eya ssentebe wa School of Medicine and Ethics committee n'omuwandiisi wa Uganda National Science and Technology committee nazo zissiddwa mu kiwandiiko kino eky'okukkiriza (ekiwa olukusa).

Ebibuuzo ebikwata ku ddembe ly'abeetaba mu kunoonyereza:

Singa wabeerawo ebibuuzo by'oyagala okubuuza ku kikwata ku bigobererwa mu kunoonyereza, okwebuuza kwonna ku bikozesebwa mu kunoonyereza oba ensonga zonna nga oli mu kunoonyereza, osobola okukubira Dr. Herbert Kayiga essimu ye ewandiikiddwa wammanga. Singa obeera n'ensonga ku mpisa ekirizibwa, obutali bumativu bwonna ku kunoonyereza, osabibwa okukubira ssentebe w'Akakiiko akakwasisa empisa mu kunoonyereza oba omuwandiisi wa National Council of Science and Technology nga ennamba ze zissiddwa wansi. Ennamba z'essimu ze zino:

4. **Dr. Herbert Kayiga**
   Department of Obstetrics and Gynecology,
   Makerere University College of Health Services,
   P.O. Box 7062, Kampala.
   0777855063.
   Email: hkayiga@gmail.com
5. **Prof Ponsiano Ocama**
   Chairperson:
   SOMREC (School of Medicine Research & Ethics Committee)
   0772421190
   Email: ponsiano.ocama@gmail.com
6. **Dr. Peter Ndemere**
   Executive Secretary- UNCST
   +256414705500
   Email: info@uncst.go.ug

Okukakasa obwa nakyewa:

Olina eddembe okugaana okwetaba mu kunoonyereza oba okukuvaamu ku kiseera kyonna eky'okubuuzibwa awatali kukosa nkolagana yo n'abanoonyereza oba abakukozesa.

Okufulumya ebivuddemu:

Ogenda kuweebwa obubaka obukwata ku ntambula y'okunoonyereza oba ebizuulibwa mu kunoonyereza. Obubaka bwonna obupya obukwata ku kunoonyereza oba ebikuŋŋaanyizibwa nga bikulu eri obujjanjabi bw'abeetaba mu kunoonyereza (nga otwaliddemu n'ebirala ebizuulibwa) bigenda kubaweebwa nga abasawo.

Okukkirizibwa mu mateeka:

Okukkirizibwa mu mateeka okw'okunoonyereza kuno kwafunibwa okuva ku SOMREC, UNCST, n'ekitongole ky'Ebyobulamu ekya Uganda. Olukusa olw'okuddukanya emirimu nalwo lwafunibwa okuva eri abakulira amalwaliro ag'enjawulo agakola omulimu.

OKUKAKASA OKUKKIRIZA/OLUKUSA

.... annyinnyonnyodde ekigenda okukolebwa, obuzibu, emiganyulo egirimu n'eddembe lyange ku kikwata ku kunoonyereza kuno. Ntegeera nti okusalawo kwange okwetaba mu kunoonyereza kuno tekijja kukyusa mbeera ya mulimu gwange oba enkolagana gye nnina n'abakola ku kunoonyereza. Mu kukozesa obubaka buno, erinnya lyange terijja kwatulwa. Nkimanyi nti nsobola okuvaamu ekiseera kyonna. Ntegeera nti okuyita mu kuteeka omukono ku kiwandiiko kino, seggyako ddembe n'erimu erimpeebwa mu mateeka wabula ndaga bulazi nti ntegeezeddwa ebikwata ku kunoonyereza kwenzikiriza kyeyagalire okwetabamu. Kopi y'ekiwandiiko kino egenda kumpeebwa.

Erinnya ………………Omukono/Ekinkumu ky'ayeetabyemu …………Ennaku z'omwezi ……

Erinnya …………………Omukono gw'omuzadde/omukuza w'abaana (bwe kiba kyetaagisa)…Ennaku z'omwezi …......................

Erinnya…………Omukono gw'omujulizi (bwe kiba kyetaagisa)………Ennaku z'omwezi…….

Erinnya ………………Omukono gw'omusoyisoyi/Omuntu afuna olukusa nga otegedde ……… Ennaku z'omwezi ………….

APPENDIX XV: LUGANDA CONSENT FOR QUALITATIVE STUDY AMONG COUPLES

EBYONGERWAKO: EKIWANDIIKO KY'OKUKKIRIZA (EKIWA OLUKUSA) EKY'OKUBUUZIBWA EKIGENDA EWALA OKW'ABAKAZI

MAKERERE UNIVERSITY COLLEGE OF HEALTH SCIENCES

SCHOOL OF MEDICINE RESEARCH AND ETHICS COMMITTEE (SOM-REC)

Omutwe gw'okunoonyereza okugenda okukolebwa:

WOMEN AND THEIR SPOUSES' PERCEPTIONS TOWARDS POST ABORTION INTRAUTERINE CONTRACEPTION AFTER FIRST TRIMESTER MEDICAL MANAGEMENT OF INCOMPLETE ABORTIONS IN CENTRAL UGANDA: A QUALITATIVE STUDY

Omunoonyereza:
DR. HERBERT KAYIGA,
Department of Obstetrics and Gynecology,
Makerere University College of Health Services,
P.O. Box 7062, Kampala.
0777855063. Email: hkayiga@gmail.com
Abakolera awamu n'omunoonyereza:
ASSOC. PROF JOSAPHAT BYAMUGISHA,
Makerere University College of Health Sciences,

Department of Obs/Gyn,
P.O. Box 7062, Kampala.
0772580330. Email: jbyamugisha@gmail.com
DR. OTHMAN KAKAIRE
Makerere University College of Health Sciences,
Department of Obs/Gyn,
P.O. Box 7062, Kampala.
0772514616, Email: kakaireothman@gmail.com
ASSOC. PROF NAZARIUS .M. TUMWESIGYE
Makerere University College of Health Sciences,
Department of Epidemiology and Biostatistics
P.O. Box 7062, Kampala.
0782447771, Email: naz@musph.ac.ug
PROF KRISTINA GEMZELL-DANIELSSON
Karolinska Institutet and Karolinska University Hospital
Email: kristina.gemzell@ki.se

Ebyafaayo n'ensonga ezikwata ku kunoonyereza:

Okunoonyereza kuno kunoonya okutunuulira endowooza z'abakazi abalina oba abatalina baagalwa baabwe nga bakozesa enkola ey'akaweta akassibwa mu nnabaana okuziyiza okufuna olubuto nga olubuto lumaze okuvaamu oluvannyuma lw'okufuna obujjanjabi bw'olubuto olutavuddemu bulungi mu myezi esatu egisooka. Mu kiseera kino enkola ebadde yakuteekamu kaweta akaziyiza olubuto mu nnabaana (akavaamu ekirungo kya Copper oba Levonorgestrel) wakati wa wiiki 2-4 oluvannyuma lw'okuggibwamu kw'ebiva mu kufuna olubuto. Ebyembi, abakazi bangi tebatera kukomawo kussibwamu buweta olw'ensonga eziwerako. Kino kibateeka mu kabi ak'okufuna embuto ezitali ŋŋenderere kubanga obusobozi bw'okufuna olubuto bukomawo mangu mu nnaku nga ttaano ku kkumi oluvannyuma lw'okuvaamu embuto.

N'olw'ekyo kye tuva tusaba okukubuuza ebikwata ku kweroboza kw'okoze okukozesa akaweta akassibwa mu nnabaana nga engeri y'okuziyiza embuto ezitayagalwa oluvannyuma lw'okuvaamu olubuto lw'obadde nalwo nga oweereddwa amakerenda ga Misoprostol ogateeke wansi w'olulimi lwo okukusobozesa okuggyamu ebintu ebyali bitavuddeeyo bulungi. Ebirowoozo byo bigenda kutusobozesa okumanya oba okuteekamu enkola eno oluvannyuma lw'okuvaamu olubuto tekirina kabi era nga kikola bulungi nga essiddwamu mu bbanga lya wiiki emu ey'okuggibwamu kw'ebiva mu kufuna olubuto nga bw'essibwamu oluvannyuma lwa wiiki 2-4 nga y'ebadde enzijjanjaba eya bulijjoe. Era tunoonya okutegeera lwaki wasazeewo okuteekebwamu akaweta mu nnabaana mu kifo ky'enkola endala ez'okuziyiza okufuna olubuto era oba nga owagira mukwano gwo akozese

enkola ey'ekika kino okuziyiza olubuto mu kiseera eky'omu maaso. Kino kigenda kutusobozesa okuwa amagezi nga twesigama ku bujulizi ku kiseera eky'okussibwamu akaweta akaziyiza olubuto mu nnabaana oluvannyuma lw'okuvaamu olubuto oluvannyuma lw'okufuna obujjanjabi mu myezi esatu egisooka nga embuto zivuddemu.

Ebikwata ku bawagira omulimu gw'okunoonyereza n'ebitongole ebikolagana n'abanoonyereza:

Nze Dr. Herbert Kayiga, nkola ku by'Okuzaalisa/endwadde z'abakyala (Obstetrician/Gynaecologist), era ndi Musomesa mu kitongole ky'Ebyokuzaalisa n'Endwadde z'abakyala (Obstetrics and Gynaecology), ekya Makerere University College of Health Sciences, omunoonyereza mu kunoonyereza okukulu "*Okukkirizika, n'Enkola n'Enkozesa y'Akaweta akaziyiza olubuto mu nnabaana nga bukyali oluvannyuma lw'okuvaamu olubuto mu kugerageranya n'enkozesa y'akaweta akaziyiza olubuto mu nnabaana eya bulijjo oluvannyuma lw'okufuna obujjanjabi bw'embuto ezitavuddemu bulungi mu myezi esatu egisooka egy'okufuna olubuto mu bitundu bya Uganda eya Wakati" ("Acceptability, Effectiveness and Uptake of Early versus Standard Post Abortion Intrauterine Contraception after First Trimester Medical Post Abortion Care for Incomplete Abortions in Central Uganda")*. Associate Professor Josaphat Byamugisha, Associate Professor Nazarius Tumwesigye, ne Dr. Othman Kakaire nabo bakolaganira wamu ne Makerere University College of Health Sciences. Bebannuŋŋamya ku mulimu guno ogw'okunoonyereza. Professor Kristina Gemzell-Danielsson, akolera wamu nange mu kunoonyereza ng'asinziira ku kitongole kya Karolinska Institutet ne Karolinska University Hospital, Sweden.

Okunoonyereza kuno kugenda kukolebwa mu bifo ewajjanjabirwa kkumi na bitaano (15) ebigenda okubeeramu eddwaliro lya Entebbe hospital, ne Mpigi HC IV, ne Masaka hospital, ne Gombe hospital, ne Nakaseke hospital, ne Luweero HC IV, ne Mengo hospital ne Naguru hospital, ne Kawempe National referral hospital, ne Kawolo hospital, ne Wakiso HC IV, ne Kasangati HC IV, ne Kiganda Health Centre IV ne Kayunga hospital.

Okunoonyereza kugenda kussibwamu ekitundu ku ssente okuyita mu nkolagana wakati wa Makerere University ne Karolinska Institutet ne Karolinska University Hospital okuyita mu Prof Kristina Gemzell-Danielsson. Makerere University esazeeko ku bisale by'essomero ate Karolinska Institutet, egenda kuwaayo ezimu ku ssente z'okunoonyereza.

Ekigendererwa:

Mu kunoonyereza kuno, tugenda kukubuuza ku kusalawo kw'okoze okukozesa akaweta akassibwa mu nnabaana nga engeri y'okuziyiza olubuto olutayagalwa oluvannyuma lw'okuvaamu olubuto lw'obadde nalwo nga waweebwa amakerenda ga Misoprostol ogateeke wansi w'olulimi lwo okukusobozesa okuggyamu ebintu ebyali bitavuddeeyo bulungi. Ebirowoozo byo bigenda kutusobozesa okumanya oba okuteekamu enkola eno oluvannyuma lw'okuvaamu olubuto tekirina kabi era nga kikola bulungi nga essiddwamu mu bbanga lya wiiki emu ey'okuggibwamu kw'ebiva mu kufuna olubuto nga bw'essibwamu oluvannyuma lwa wiiki 2-4 nga y'ebadde enzijjanjaba eya bulijjo. Era tunoonya okutegeera lwaki wasazeewo okuteekebwamu akaweta mu nnabaana mu kifo ky'enkola endala ez'okuziyiza okufuna olubuto era oba nga owagira mukwano gwo akozese enkola ey'ekika kino okuziyiza olubuto mu kiseera eky'omu maaso. Era tunoonya okumanya engeri gye wayita ku biremesa

Okugeza nga empisa ez'obuwangwa okukozesa enkola ey'akaweta akassibwa mu nnabaana okuziyiza okufuna olubuto nga olubuto lumaze okuvaamu

Ekiseera ekiteeberezebwa ayeetabye mu kunoonyereza ky'ajja okutwala ng'ali mu kunoonyereza:

Tuteekateeka okutwala eddakiika nga 45-90 nga tukubuuza ebibuuzo wekka oba nga oli n'omwagalwa wo ku kikwata ku kweroboza ku nkola y'okuziyiza okufuna olubuto nga olubuto lumaze okuvaamu kw'osazeewo okukozesa oluvannyuma lw'okufuna obujjanjabi nga olubuto lwo luvuddemu mu myezi esatu egisooka.

Ebigobererwa mu kunoonyereza:

Ogenda kubuuzibwa ebibuuzo ebikwata ku nsonga eziri emabega w'okusalawo kwo era oba nga owagira mikwano gyo okukozesa enkola eno oba n'okuddamu okukozesa enkola eno mu kiseera eky'omu maaso. Tugenda kukozesa obubaka bw'otuwa okuzuula ekiseera ekituufu eky'okuteekamu obuweta mu nnabaana oluvannyuma lw'obutavaamu bulungi lubuto mu wiiki 12 ezisooka okuziyiza embuto ezitayagalwa era ezitagendereddwa mu kiseera eky'omu maaso. Kino kiri bwe kiti kubanga obusobozi bw'okufuna olubuto olulala bukomawo mu bbanga lya wiiki bbiri oluvannyuma lw'okuggyamu ebiva mu kufuna olubuto. Ogenda kubuuzibwa wekka oba wamu n'omwagalwa wo nga bw'onasalawo ku kikusingira.

Ani agenda okwetaba mu kunoonyereza?

Okunoonyereza era kugenda kubeeramu okubuuza abakazi oba abaagalana 20-30 abasalawo okukozesa enkola ey’akaweta akassibwa mu nnabaana okuziyiza okufuna olubuto nga olubuto lumaze okuvaamu nga tweyambisa okubuuza ebibuuzo ekigenda ewala okutegeera ebiremesa n’ebiyambako mu kukozesa enkola ze balonze ez’ekizaala gumba (140).

Obuzibu/Okutawaanyizibwa: Obuweta obussibwa mu nnabaana (IUDs) aka copper T n’akavaamu ekirungo kya Levonorgestrel bukozesebwa mu Uganda mu nkola ey’ekizaala gumba (140). Mu kukozesa obuweta obussibwa mu nnabaana, akaweta ka copper IUD, kasobola okukuleetera okulwa mu nsonga oba okulumizibwa nga oli mu nsonga. Waliwo obuzibu bw’okuvaamu omusaayi wakati mu nsonga zo. Wabula obubonero buno busobola okukendeera mu bbanga lya myezi mukaaga oba busobola okujjanjabwa n’eddagala nga n’eddagala eddala. Era waliwo omukisa gwa butundutundu munaana ku buli kikumi ogw’okufuna olubuto nga okozesa enkola eno. Mu kukoesa akaweta ka Levonorgestrel IUD, wayinza okubeerawo okukendeera mu mirundi gy’ogenda mu nsonga oba ebiseera ebimu obutalaba kugenda mu nsonga kwonna. Era waliwo obuzibu bwa katundutundu kamu ku buli kikumi obw’okufuna olubuto nga okozesa enkola eno. Newankubadde nga akaweta kakola mu ngeri eya bulijjo, ebiseera ebimu ekirungo kya Levonorgestrel kisobola okugenda mu musaayi gwo era kino kisobola okukyusa embeera yo. Tewali n’emu kun kola eya IUDs esobola okukuziyiza obutafuna kawuka ka siriimu (HIV) oba endwadde endala zonna ez’ekikaba naddala nga weetaba mu kikolwa eky’okwegatta nga tolina bukuumi.

Singa ofuna ekimu ku bino; okulwala okw’amaanyi oluvannyuma lw’okuggyamu ebibadde mu nnabaana, nga wetaaga okulongoosebwa (okusala mu lubuto okunoonyereza (exploratory laparotomy)) oluvannyuma lw’okuteekamu akaweta mu nnabaana nga omaze okuvaamu olubuto, nga wetaaga okussibwako eccupa y’omusaayi oluvannyuma lw’okuvaamu omusaayi okw’amaanyi, okuweebwa ekitanda okumala essaawa 72, okuyisibwa obubi okw’amaanyi okutali kwa bulijjo (anaphylactic reaction) oluvannyuma lw’okuteekamu akaweta ka IUD, oba okufa kwo oba okw’omwagalwa wo, bino bigenda kutwalibwa nga okuyisibwa obubi okw’amaanyi ennyo.Abeetabyemu abafunye okuyisibwa obubi bagenda kujjanjabibwa mangu abakola ku by’okujjanjaba abali ku malwaliro ag’enjawulo okusinziira ku nkola egobererwa bulijjo. Abo abetaaga okufuna obuweereza obw’okusindikibwa ewalala bagenda kuweebwa obujjanjabi obulungi nga busasulirwa abanoonyereza. Tugenda kukusaba otegeeze Omunoonyereza omukulu

oba abakola ku kunoonyereza amangu ddala ng'ebintu ebyo bibaddewo wasobole okubeerawo ekikolebwa okutereeza embeera mu kiseera ekituufu.
Mu kunoonyereza kuno kwennyini, wagenda kubeerawo obuzibu butono obusuubirwa mu kubuuzibwa.

Emiganyulo:

Abeetaba mu kunoonyereza abagenda kulondoolwa nnyo mu kujjanjabibwa kwabwe nga tebavuddemu bulungi lubuto mu myezi esatu egisooka egy'okufuna olubuto mu kugerageranya n'abo abafuna obujjanjabi obwa bulijjo. Enkola eno egenda kuziyiza embuto ezitagendereddwa ekigasa abeetabyemu. Mu ngeri y'okubaako kye tukola eri obulamu bw'abantu, okunoonyereza kuno kugenda kuyamba mu kukola enkola ey'okuziyiza olubuto ng'omaze okuvaamu olubuto oluvannyuma lw'emyezi esatu egisooka egy'okufuna olubuto nga embuto zivuddemu etaliiwo mu kiseera kino.
Tusuubira nti emiganyulo gy'okuziyiza okufuna olubuto nga lumaze okuvaamu oluvannyuma lw'emyezi esatu egisooka egy'okufuna olubuto nga embuto zivuddemu gisinga obuzibu obuyinza okuva mu kufuna embuto ezitagenderddwa n'ebizibu ebigenderako.
Abakola ku kunoonyereza bagenda kukunnyonnyola ku nkola eziriwo ez'ekizaala gumba, amaanyi gaazo, enkola yaazo n'emiganyulo gy'enkola eziriwo mu kugerageranya n'enkola endala.

Okukuuma ebyama:
Ebikwata ku mulwadde ebikuŋŋaanyizibwa bigenda kukuumibwa mu kompyuta ezirimu paasiwaadi. Ebikwata ku beetabyemu byonna (entambi z'amaloboozi, ebiwandiiko, kkopi ezissiddwa mu buwandiike n'obubaluwa) bigenda kuterekebwa mu kifo ekikuumibwa ekigenda okuggalwa era nga kituukibwamu abantu bokka abali obutereevu mu kunoonyereza. Abeetabye mu kunoonyereza bagenda kuyitbwa amannya agatali gaabwe mu lipooti esembayo. Abakola ku kunoonyereza bagenda kuteeka omukono ku biwandiiko by'okukuuma ebyama okukakasa nti banywerera ku kukuuma ebyama by'abetabyemu ekiseera kyonna mu kunoonyereza n'oluvannyuma lw'okunoonyereza.
Olw'okuba nga omunoonyereza omukulu n'Akakiiko akavunaanyizibwa ku by'Empisa mu kunoonyereza ak'omu kitundu (Research Ethics Committee (REC)) ne Uganda National Council for Science and Technology (UNCST) bagenda kulaba obubaka bwo obw'ekyma obukukwatako

obwawula abeetabye mu kunoonyereza okusinziira ku linnya okukakasa nti okuumibwa obutafuna kabi okumalako ekiseera kyonna eky’okunoonyereza.

Okweroboza okulala:

Olina eddembe okugaana okwetaba mu kunoonyereza oba okukuvaamu ku kiseera kyonna eky’okubuuzibwa awatali kukosa mutindo gwa bujjanjabi obukuweebwa nga oli mu ddwaliro. Singa werobozako engeri endala mu kifo ky’obujjanjabi bw’olubuto olutavuddemu bulungi mu myezi esatu egisooka, ogenda kuweebwa engeri endala ey’okulongoosebwa baggyemu ebintu ebisigaddemu ebiva mu kufuna olubuto. Enkola endala ez’ekizaala gumba (140) nga oggyeeko ey’okussibwamu akaweta mu nnabaana akaziyiza olubuto nazo zijja kukuweebwa osalewo nga otegedde ku kisinga okukukolera.

Ebisale ebisasulwa:

Ebisale by’eddagala nga Misoprostol, eddagala eriziyiza okukula kw’obuwuka n’okubusaanyawo (antibiotics) n’eddagala erikendeeza obulumi mu kiseera eky’okujjanjabibwa n’obuweta obussibwa mu nnabaana bigenda kusasulwa abakola ku kunoonyereza. Obuzibu bwonna obutasuubirwa nga buva ku bikozesebwa mu kunoonyereza obuyinza okukutuukako mu kunoonyereza bugenda kusasulirwa abakola ku kunoonyereza.

Okuliyirirwa olw’okwetaba mu kunoonyereza:

Ogenda kuweebwa 30,000/= (siringisi za Uganda emitwalo esatu) nga okuliyirirwa olw’obudde bwo okwetaba mu kunoonyereza. Singa wabeerawo okukosebwa okw’olubeerera kwonna oba ebisago eby’amaanyi byonna nga okunoonyereza kugenda mu maaso, ebisale by’obujjanjabi bw’abeetaba mu kunoonyereza bigenda kusasulwa abakola ku kunoonyereza.

Okuddiza:

Abeetaba mu kunoonyereza bonna bagenda kuliyirirwa olw’obudde bwabwe era bagenda kuddizibwawo ssente z’entambula.

Ebibuuzo ebikwata ku kunoonyereza:

Ennamba ya nansi akola ku kunoonyereza ebeerako essaawa yonna egenda kuweebwa abeetaba mu kunoonyereza bonna okumala essaawa 24 buli wiiki okukola ku nsonga zonna ze bagenda okuba nazo nga bafuna obujjanjabi mu kunoonyereza oba mu kiseera ky’okunoonyereza. Kino kigenda kusobozesa okwanukula mu budde eri ensonga zabeetaba mu kunoonyereza. Essimu y’Omunoonyereza omukulu, n’eya ssentebe wa School of Medicine and Ethics committee

n'omuwandiisi wa Uganda National Science and Technology committee nazo zissiddwa mu kiwandiiko kino eky'okukkiriza (ekiwa olukusa).

Ebibuuzo ebikwata ku ddembe ly'abeetaba mu kunoonyereza:

Singa wabeerawo ebibuuzo by'oyagala okubuuza ku kikwata ku bigobererwa mu kunoonyereza, okwebuuza kwonna ku bikozesebwa mu kunoonyereza oba ensonga zonna nga oli mu kunoonyereza, osobola okukubira Dr. Herbert Kayiga essimu ye ewandiikiddwa wammanga. Singa obeera n'ensonga ku mpisa ekirizibwa, obutali bumativu bwonna ku kunoonyereza, osabibwa okukubira ssentebe w'Akakiiko akakwasisa empisa mu kunoonyereza oba omuwandiisi wa National Council of Science and Technology nga ennamba ze zissiddwa wansi. Ennamba z'essimu ze zino:

4. **Dr. Herbert Kayiga**
Department of Obstetrics and Gynecology,
Makerere University College of Health Services,
P.O. Box 7062, Kampala.
0777855063.
Email: hkayiga@gmail.com
5. **Prof Ponsiano Ocama**
Chairperson:
SOMREC (School of Medicine Research & Ethics Committee)
0772421190
Email: ponsiano.ocama@gmail.com
6. **Dr. Peter Ndemere**
Executive Secretary- UNCST
+256414705500
Email: info@uncst.go.ug

Okukakasa obwa nnakyewa:

Olina eddembe okugaana okwetaba mu kunoonyereza oba okukuvaamu ku kiseera kyonna eky'okubuuzibwa awatali kukosa mutindo gwa bujjanjabi obukuweebwa nga oli mu ddwaliro.

Okufulumya ebivuddemu:

Ogenda kuweebwa obubaka obukwata ku ntambula y'okunoonyereza oba ebizuulibwa mu kunoonyereza. Obubaka bwonna obupya obukwata ku kunoonyereza oba ebikuŋŋaanyizibwa nga bikulu eri obujjanjabi bw'abeetaba mu kunoonyereza (nga otwaliddemu n'ebirala ebizuulibwa) bigenda kukuweebwa ne/oba abasawo bo.

Okukkirizibwa mu mateeka:

Okukkirizibwa mu mateeka okw'okunoonyereza kuno kwafunibwa okuva ku SOMREC, UNCST, n'ekitongole ky'Ebyobulamu ekya Uganda. Olukusa olw'okuddukanya emirimu nalwo lwafunibwa okuva eri abakulira amalwaliro ag'enjawulo agakola omulimu n'ekitongole kya Obstetrics and Gynaecology, Makerere University College of Health Sciences ekikola ku by'okuzaalisa n'endwadde z'abakyala. Okunoonyereza era kwawandiisibwa ku ClinicalTrials.gov ne kwanjulwa okusinziira ku ndagiriro ya CONSORT.

OKUKAKASA OKUKKIRIZA/OLUKUSA

.... annyinnyonnyodde ekigenda okukolebwa, obuzibu, emiganyulo egirimu n'eddembe lyange ku kikwata ku kunoonyereza kuno. Ntegeera nti okusalawo kwange okwetaba mu kunoonyereza kuno tekijja kukyusa bujjanjabi bwange bwenfuna bulijjo. Mu kukozesa obubaka buno, erinnya lyange terijja kwatulwa. Nkimanyi nti nsobola okuvaamu ekiseera kyonna. Ntegeera nti okuyita mu kuteeka omukono ku kiwandiiko kino, seggyako ddembe n'erimu erimpeebwa mu mateeka wabula ndaga bulazi nti ntegeezeddwa ebikwata ku kunoonyereza kwenzikiriza kyeyagalire okwetabamu. Kopi y'ekiwandiiko kino egenda kumpeebwa.

Erinnya ………………Omukono/Ekinkumu ky'ayeetabyemu …………Ennaku z'omwezi ……

Erinnya …………………Omukono gw'omuzadde/omukuza w'abaana (bwe kiba kyetaagisa)…Ennaku z'omwezi …........................

Erinnya…………Omukono gw'omujulizi (bwe kiba kyetaagisa)………Ennaku z'omwezi…….

Erinnya ………………Omukono gw'omusoyisoyi/Omuntu afuna olukusa nga otegedde ……… Ennaku z'omwezi ………….

APPENDIX XVI: LUGANDA CONSENT FOR SPOUSES

EBYONGERWAKO: EKIWANDIIKO KY'OKUKKIRIZA (EKIWA OLUKUSA) EKY'OKUBUUZIBWA EKIGENDA EWALA EKY'ABAAMI

MAKERERE UNIVERSITY COLLEGE OF HEALTH SCIENCES

SCHOOL OF MEDICINE RESEARCH AND ETHICS COMMITTEE (SOM-REC)

Omutwe gw'okunoonyereza okugenda okukolebwa:

WOMEN AND THEIR SPOUSES' PERCEPTIONS TOWARDS POST ABORTION INTRAUTERINE CONTRACEPTION AFTER FIRST TRIMESTER MEDICAL MANAGEMENT OF INCOMPLETE ABORTIONS IN CENTRAL UGANDA: A QUALITATIVE STUDY

Omunoonyereza:

DR. HERBERT KAYIGA,
Department of Obstetrics and Gynecology,
Makerere University College of Health Services,
P.O. Box 7062, Kampala.
0777855063. Email: hkayiga@gmail.com

Abakolera awamu n'omunoonyereza:

ASSOC. PROF JOSAPHAT BYAMUGISHA,

Makerere University College of Health Sciences,
Department of Obs/Gyn,
P.O. Box 7062, Kampala.
0772580330. Email: jbyamugisha@gmail.com
DR. OTHMAN KAKAIRE
Makerere University College of Health Sciences,
Department of Obs/Gyn,
P.O. Box 7062, Kampala.
0772514616, Email: kakaireothman@gmail.com
ASSOC. PROF NAZARIUS .M. TUMWESIGYE
Makerere University College of Health Sciences,
Department of Epidemiology and Biostatistics
P.O. Box 7062, Kampala.
0782447771, Email: naz@musph.ac.ug
PROF KRISTINA GEMZELL-DANIELSSON
Karolinska Institutet and Karolinska University Hospital
Email: kristina.gemzell@ki.se

Ebyafaayo n'ensonga ezikwata ku kunoonyereza:

Okunoonyereza kuno kunoonya okutunuulira endowooza z'abakazi abalina oba abatalina baagalwa baabwe nga bakozesa enkola ey'akaweta akassibwa mu nnabaana okuziyiza okufuna olubuto nga olubuto lumaze okuvaamu oluvannyuma lw'okufuna obujjanjabi bw'olubuto olutavuddemu bulungi mu myezi esatu egisooka. Mu kiseera kino enkola ebadde yakuteekamu kaweta akaziyiza olubuto mu nnabaana (akavaamu ekirungo kya Copper oba Levonorgestrel) wakati wa wiiki 2-4 oluvannyuma lw'okuggibwamu kw'ebiva mu kufuna olubuto. Ebyembi, abakazi bangi tebatera kukomawo kussibwamu buweta olw'ensonga eziwerako. Kino kibateeka mu kabi ak'okufuna embuto ezitali ŋŋenderere kubanga obusobozi bw'okufuna olubuto bukomawo mangu mu nnaku nga ttaano ku kkumi oluvannyuma lw'okuvaamu embuto.

N'olw'ekyo kye tuva tusaba okukubuuza ebikwata ku kweroboza kw'okoze okukozesa akaweta akassibwa mu nnabaana nga engeri y'okuziyiza embuto ezitayagalwa oluvannyuma lw'okuvaamu olubuto lw'obadde nalwo nga oweereddwa amakerenda ga Misoprostol ogateeke wansi w'olulimi lwo okukusobozesa okuggyamu ebintu ebyali bitavuddeeyo bulungi. Ebirowoozo byo bigenda kutusobozesa okumanya oba okuteekamu enkola eno oluvannyuma lw'okuvaamu olubuto tekirina kabi era nga kikola bulungi nga essiddwamu mu bbanga lya wiiki emu ey'okuggibwamu kw'ebiva mu kufuna olubuto nga bw'essibwamu oluvannyuma lwa wiiki 2-4 nga y'ebadde enzijjanjaba eya bulijjoe. Era tunoonya okutegeera lwaki wasazeewo okuteekebwamu akaweta mu nnabaana mu kifo ky'enkola endala ez'okuziyiza okufuna olubuto era oba nga owagira mukwano gwo akozese enkola ey'ekika kino okuziyiza olubuto mu kiseera eky'omu maaso. Kino kigenda kutusobozesa

okuwa amagezi nga twesigama ku bujulizi ku kiseera eky'okussibwamu akaweta akaziyiza olubuto mu nnabaana oluvannyuma lw'okuvaamu olubuto oluvannyuma lw'okufuna obujjanjabi mu myezi esatu egisooka nga embuto zivuddemu.

Ebikwata ku bawagira omulimu gw'okunoonyereza n'ebitongole ebikolagana n'abanoonyereza:

Nze Dr. Herbert Kayiga, nkola ku by'Okuzaalisa/endwadde z'abakyala (Obstetrician/Gynaecologist), era ndi Musomesa mu kitongole ky'Ebyokuzaalisa n'Endwadde z'abakyala (Obstetrics and Gynaecology), ekya Makerere University College of Health Sciences, omunoonyereza mu kunoonyereza okukulu "*Okukkirizika, n'Enkola n'Enkozesa y'Akaweta akaziyiza olubuto mu nnabaana nga bukyali oluvannyuma lw'okuvaamu olubuto mu kugerageranya n'enkozesa y'akaweta akaziyiza olubuto mu nnabaana eya bulijjo oluvannyuma lw'okufuna obujjanjabi bw'embuto ezitavuddemu bulungi mu myezi esatu egisooka egy'okufuna olubuto mu bitundu bya Uganda eya Wakati" ("Acceptability, Effectiveness and Uptake of Early versus Standard Post Abortion Intrauterine Contraception after First Trimester Medical Post Abortion Care for Incomplete Abortions in Central Uganda").* Associate Professor Josaphat Byamugisha, Associate Professor Nazarius Tumwesigye, ne Dr. Othman Kakaire nabo bakolaganira wamu ne Makerere University College of Health Sciences. Bebannuŋŋamya ku mulimu guno ogw'okunoonyereza. Professor Kristina Gemzell-Danielsson, akolera wamu nange mu kunoonyereza ng'asinziira ku kitongole kya Karolinska Institutet ne Karolinska University Hospital, Sweden.

Okunoonyereza kuno kugenda kukolebwa mu bifo ewajjanjabirwa kkumi na bitaano (15) ebigenda okubeeramu eddwaliro lya Entebbe hospital, ne Mpigi HC IV, ne Masaka hospital, ne Gombe hospital, ne Nakaseke hospital, ne Luweero HC IV, ne Mengo hospital, ne Naguru hospital, ne Kawempe National referral hospital, ne Kawolo hospital, ne Wakiso HC IV, ne Kasangati HC IV, ne Kiganda Health Centre IV ne Kayunga hospital.

Okunoonyereza kugenda kussibwamu ekitundu ku ssente okuyita mu nkolagana wakati wa Makerere University ne Karolinska Institutet ne Karolinska University Hospital okuyita mu Prof Kristina Gemzell-Danielsson. Makerere University esazeeko ku bisale by'essomero ate Karolinska Institutet, egenda kuwaayo ezimu ku ssente z'okunoonyereza.

Ekigendererwa:

Mu kunoonyereza kuno, tugenda kukubuuza ku kusalawo omwagalwa wo kw'akoze okukozesa akaweta akassibwa mu nnabaana nga engeri y'okuziyiza olubuto olutayagalwa oluvannyuma lw'okuvaamu olubuto lw'abadde nalwo nga yaweebwa amakerenda ga Misoprostol agateeke wansi w'olulimi lwe okumusobozesa okuggyamu ebintu ebyali bitavuddeeyo bulungi. Ebirowoozo byo bigenda kutusobozesa okumanya oba okuteekamu enkola eno oluvannyuma lw'okuvaamu olubuto tekirina kabi era nga kikola bulungi nga essiddwamu mu bbanga lya wiiki emu ey'okuggibwamu kw'ebiva mu kufuna olubuto nga bw'essibwamu oluvannyuma lwa wiiki 2-4 nga y'ebadde enzijjanjaba eya bulijjo. Era tunoonya okutegeera lwaki wasazeewo akaweta akassibwa mu nnabaana mu kifo ky'enkola endala ez'okuziyiza okufuna olubuto era oba nga owagira mukwano gwo akozese enkola ey'ekika kino okuziyiza olubuto mu kiseera eky'omu maaso. Era tunoonya okumanya engeri gye wayita ku biremesa okugeza nga empisa ez'obuwangwa okukozesa enkola ey'akaweta akassibwa mu nnabaana okuziyiza okufuna olubuto nga olubuto lumaze okuvaamu

Ekiseera ekiteeberezebwa ayeetabye mu kunoonyereza ky'ajja okutwala ng'ali mu kunoonyereza:

Tuteekateeka okutwala eddakiika nga 45-90 nga tukubuuza ebibuuzo wekka oba nga oli n'omwagalwa wo ku kikwata ku kweroboza ku nkola y'okuziyiza okufuna olubuto nga olubuto lumaze okuvaamu kw'osazeewo okukozesa oluvannyuma lw'okufuna obujjanjabi nga olubuto lwo luvuddemu mu myezi esatu egisooka.

Ebigobererwa mu kunoonyereza:

Ogenda kubuuzibwa ebibuuzo ebikwata ku nsonga eziri emabega w'okusalawo kwo era oba nga owagira mikwano gyo okukozesa enkola eno oba n'okuddamu okukozesa enkola eno mu kiseera eky'omu maaso. Tugenda kukozesa obubaka bw'otuwa okuzuula ebiremesa n'ebiyambako ku nkola ey'akaweta akassibwa mu nnabaana okuziyiza okufuna olubuto nga olubuto lumaze okuvaamu mu bafumbo. Obubaka nga obwo bugenda kutuyamba okuluŋŋamya mu kukola kw'endagiriro nga besigama ku bujulizi bwennyini obukwata ku mulamwa ku nkola ey'okuziyiza okufuna olubuto nga olubuto lumaze okuvaamu. Ogenda kubuuzibwa wekka oba wamu n'omwagalwa wo nga bw'onasalawo ku kikusingira.

Ani agenda okwetaba mu kunoonyereza?

Okunoonyereza era kugenda kubeeramu okubuuza abafumbo 20-30 abasalawo okukozesa enkola ey'akaweta akassibwa mu nnabaana okuziyiza okufuna olubuto nga olubuto lumaze okuvaamu

nga tweyambisa okubuuza ebibuuzo ekigenda ewala okutegeera ebiremesa n'ebiyambako mu kukozesa enkola ze balonze ez'ekizaala gumba (140).

Obuzibu/Okutawaanyizibwa: Obuweta obussibwa mu nnabaana (IUDs) aka copper T n'akavaamu ekirungo kya Levonorgestrel bukozesebwa mu Uganda mu nkola ey'ekizaala gumba (140). Mu kukozesa obuweta obussibwa mu nnabaana, akaweta ka copper IUD, kasobola okukuleetera okulwa mu nsonga oba okulumizibwa nga oli mu nsonga. Waliwo obuzibu bw'okuvaamu omusaayi wakati mu nsonga zo. Wabula obubonero buno busobola okukendeera mu bbanga lya myezi mukaaga oba busobola okujjanjabwa n'eddagala nga n'eddagala eddala. Era waliwo omukisa gwa butundutundu munaana ku buli kikumi ogw'okufuna olubuto nga okozesa enkola eno. Mu kukoesa akaweta ka Levonorgestrel IUD, wayinza okubeerawo okukendeera mu mirundi gy'ogenda mu nsonga oba ebiseera ebimu obutalaba kugenda mu nsonga kwonna. Era waliwo obuzibu bwa katundutundu kamu ku buli kikumi obw'okufuna olubuto nga okozesa enkola eno. Newankubadde nga akaweta kakola mu ngeri eya bulijjo, ebiseera ebimu ekirungo kya Levonorgestrel kisobola okugenda mu musaayi gwo era kino kisobola okukyusa embeera yo. Tewali n'emu kun kola eya IUDs esobola okukuziyiza obutafuna kawuka ka siriimu (HIV) oba endwadde endala zonna ez'ekikaba naddala nga weetaba mu kikolwa eky'okwegatta nga tolina bukuumi.

Mu kunoonyereza kuno kwennyini, wagenda kubeerawo obuzibu butono obusuubirwa mu kubuuzibwa.

Emiganyulo:

Enkola omwagalwa wo gy'akozesa egenda kuziyiza embuto ezitagendereddwa. Mu ngeri y'okubaako kye tukola eri obulamu bw'abantu, okunoonyereza kuno kugenda kuyamba mu kukola enkola ey'okuziyiza olubuto ng'omaze okuvaamu olubuto oluvannyuma lw'emyezi esatu egisooka nga embuto zivuddemu etaliiwo mu kiseera kino.

Tusuubira nti emiganyulo gy'okuziyiza okufuna olubuto nga lumaze okuvaamu oluvannyuma lw'emyezi esatu egisooka egy'okufuna olubuto nga embuto zivuddemu gisinga obuzibu obuyinza okuva mu kufuna embuto ezitagenderddwa n'ebizibu ebigenderako.

Abakola ku kunoonyereza bagenda kukunnyonnyola ku nkola eziriwo ez'ekizaala gumba, amaanyi gaazo, enkola yaazo n'emiganyulo gy'enkola eziriwo mu kugerageranya n'enkola endala.

Okukuuma ebyama:

Ebikwata ku mulwadde ebikuŋŋaanyizibwa bigenda kukuumibwa mu kompyuta ezirimu paasiwaadi. Ebikwata ku beetabyemu byonna (entambi z'amaloboozi, ebiwandiiko, kkopi ezissiddwa mu buwandiike n'obubaluwa) bigenda kuterekebwa mu kifo ekikuumibwa ekigenda okuggalwa era nga kituukibwamu abantu bokka abali obutereevu mu kunoonyereza. Abeetabye mu kunoonyereza bagenda kuyitbwa amannya agatali gaabwe mu lipooti esembayo. Abakola ku kunoonyereza bagenda kuteeka omukono ku biwandiiko by'okukuuma ebyama okukakasa nti banywerera ku kukuuma ebyama by'abetabyemu ekiseera kyonna mu kunoonyereza n'oluvannyuma lw'okunoonyereza.

Olw'okuba nga omunoonyereza omukulu n'Akakiiko akavunaanyizibwa ku by'Empisa mu kunoonyereza ak'omu kitundu (Research Ethics Committee (REC)) ne Uganda National Council for Science and Technology (UNCST) bagenda kulaba obubaka bwo obw'ekyma obukukwatako obwawula abeetabye mu kunoonyereza okusinziira ku linnya okukakasa nti okuumibwa obutafuna kabi okumalako ekiseera kyonna eky'okunoonyereza.

Okweroboza okulala:

Olina eddembe okugaana okwetaba mu kunoonyereza oba okukuvaamu ku kiseera kyonna eky'okubuuzibwa awatali kukosa mutindo gwa bujjanjabi obukuweebwa oba obuweebwa omwagalwa wo ng'ali mu ddwaliro. Enkola endala ez'ekizaala gumba (140) nga oggyeeko ey'okussibwamu akaweta mu nnabaana akaziyiza olubuto nazo zijja kukuweebwa osalewo nga otegedde ku kisinga okukukolera.

Ebisale ebisasulwa:

Ebisale by'eddagala nga Misoprostol, eddagala eriziyiza okukula kw'obuwuka n'okubusaanyawo (antibiotics) n'eddagala erikendeeza obulumi mu kiseera eky'okujjanjabibwa n'obuweta obussibwa mu nnabaana bigenda kusasulwa abakola ku kunoonyereza. Obuzibu bwonna obutasuubirwa nga buva ku bikozesebwa mu kunoonyereza obuyinza okukutuukako mu kunoonyereza bugenda kusasulirwa abakola ku kunoonyereza.

Okuliyirirwa olw'okwetaba mu kunoonyereza:

Ogenda kuweebwa 30,000/= (siringisi emitwalo esatu) nga okuliyirirwa olw'obudde bwo okwetaba mu kunoonyereza. Singa wabeerawo okukosebwa okw'olubeerera kwonna oba ebisago eby'amaanyi byonna nga okunoonyereza kugenda mu maaso, ebisale by'obujjanjabi bw'abeetaba mu kunoonyereza bigenda kusasulwa abakola ku kunoonyereza.

Okuddiza:

Abeetaba mu kunoonyereza bonna bagenda kuliyirirwa olw’obudde bwabwe era bagenda kuddizibwawo ssente z’entambula.

Ebibuuzo ebikwata ku kunoonyereza:

Ennamba ya nansi akola ku kunoonyereza ebeerako essaawa yonna egenda kuweebwa abeetaba mu kunoonyereza bonna okumala essaawa 24 buli wiiki okukola ku nsonga zonna ze bagenda okuba nazo nga bafuna obujjanjabi mu kunoonyereza oba mu kiseera ky’okunoonyereza. Kino kigenda kusobozesa okwanukula mu budde eri ensonga zabeetaba mu kunoonyereza. Essimu y’Omunoonyereza omukulu, n’eya ssentebe wa School of Medicine and Ethics committee n’omuwandiisi wa Uganda National Science and Technology committee nazo zissiddwa mu kiwandiiko kino eky’okukkiriza (ekiwa olukusa).

Ebibuuzo ebikwata ku ddembe ly’abeetaba mu kunoonyereza:

Singa wabeerawo ebibuuzo by’oyagala okubuuza ku kikwata ku bigobererwa mu kunoonyereza, okwebuuza kwonna ku bikozesebwa mu kunoonyereza oba ensonga zonna nga oli mu kunoonyereza, osobola okukubira Dr. Herbert Kayiga essimu ye ewandiikiddwa wammanga. Singa obeera n’ensonga ku mpisa ekirizibwa, obutali bumativu bwonna ku kunoonyereza, osabibwa okukubira ssentebe w’Akakiiko akakwasisa empisa mu kunoonyereza oba omuwandiisi wa National Council of Science and Technology nga ennamba ze zissiddwa wansi. Ennamba z’essimu ze zino:

4. **Dr. Herbert Kayiga**

   Department of Obstetrics and Gynecology,

   Makerere University College of Health Services,

   P.O. Box 7062, Kampala.

   0777855063.

   Email: hkayiga@gmail.com

5. **Prof Ponsiano Ocama**

   Chairperson:

   SOMREC (School of Medicine Research & Ethics Committee)

   0772421190

   Email: ponsiano.ocama@gmail.com

6. **Dr. Peter Ndemere**

Executive Secretary- UNCST

+256414705500

Email: info@uncst.go.ug

Okukakasa obwa nnakyewa:

Olina eddembe okugaana okwetaba mu kunoonyereza oba okukuvaamu ku kiseera kyonna eky'okubuuzibwa awatali kukosa mutindo gwa bujjanjabi obukuweebwa oba obuweebwa omwagalwa wo nga muli mu ddwaliro.

Okufulumya ebivuddemu:

Ogenda kuweebwa obubaka obukwata ku ntambula y'okunoonyereza oba ebizuulibwa mu kunoonyereza. Obubaka bwonna obupya obukwata ku kunoonyereza oba ebikuŋŋaanyizibwa nga bikulu eri obujjanjabi bw'abeetaba mu kunoonyereza (nga otwaliddemu n'ebirala ebizuulibwa) bigenda kukuweebwa ne/oba abasawo bo.

Okukkirizibwa mu mateeka:

Okukkirizibwa mu mateeka okw'okunoonyereza kuno kwafunibwa okuva ku SOMREC, UNCST, n'ekitongole ky'Ebyobulamu ekya Uganda. Olukusa olw'okuddukanya emirimu nalwo lwafunibwa okuva eri abakulira amalwaliro ag'enjawulo agakola omulimu.

OKUKAKASA OKUKKIRIZA/OLUKUSA

.... annyinnyonnyodde ekigenda okukolebwa, obuzibu, emiganyulo egirimu n'eddembe lyange ku kikwata ku kunoonyereza kuno. Ntegeera nti okusalawo kwange okwetaba mu kunoonyereza kuno tekijja kukyusa bujjanjabi bwa mwagalwa wange bw'afuna bulijjo. Mu kukozesa obubaka buno, erinnya lyange terijja kwatulwa. Nkimanyi nti nsobola okuvaamu ekiseera kyonna. Ntegeera nti okuyita mu kuteeka omukono ku kiwandiiko kino, seggyako ddembe n'erimu erimpeebwa mu mateeka wabula ndaga bulazi nti ntegeezeddwa ebikwata ku kunoonyereza kwenzikiriza kyeyagalire okwetabamu. Kopi y'ekiwandiiko kino egenda kumpeebwa.

Erinnya ………………Omukono/Ekinkumu ky'ayeetabyemu …………Ennaku z'omwezi ……

Erinnya …………………Omukono gw'omuzadde/omukuza w'abaana (bwe kiba kyetaagisa)…Ennaku z'omwezi …........................

Erinnya…………Omukono gw'omujulizi (bwe kiba kyetaagisa)………Ennaku z'omwezi…….

Erinnya ………………Omukono gw'omusoyisoyi/Omuntu afuna olukusa nga otegedde ………

Ennaku z'omwezi ………….

APPENDIX XVII: DATA COLLECTION TOOL

Part A: SECTION 1: SOCIO- DEMOGRAPHIC DATA

Question Number	Questions	Response categories		Skip to
**1.**	Date of case recruitment	…………………………………...		
**2.**	Register/Unit No.	Number..<br>…………………………………...		
**3.**	Patient's Age	…………………………………		
**4.**	What is your religious affiliation?	Catholic……………………….1<br>Anglican/Protestant…………..2<br>Moslem……………………….3<br>Pentecostal/Born Again……….4<br>Adventist……………………..5		

		Atheist………………………..6<br><br>Other (specify)………………..7		
**5.**	Gravidity	………………………………..		
**6.**	Prior Pregnancy outcome	Live birth………………………1<br><br>Spontaneous Abortion………….2<br><br>Induced abortion………………3<br><br>IUFD………………………….4<br><br>ENND…………………………5<br><br>No prior pregnancy……………6<br><br>Child with a disability…………7		
**7.**	How many children do you have currently?	1……………………………..1<br><br>2-3……………………………2<br><br>4-5……………………………3<br><br>>5……………………………4		
**8.**	Your Level of Education	Primary 7……………………..1<br><br>S1-S4………………………….2<br><br>S5-S6………………………….3<br><br>Tertiary ……………………….4<br><br>Other (specify)………………..5<br><br>Didn't go for formal education…6		

**9.**	Your spouse's level of Education	Primary 7……………………..1<br>S1-S4………………………….2<br>S5-S6………………………….3<br>Tertiary ……………………….4<br>Other (specify)………………..5<br>Didn't go for formal education…6		
**10.**	Occupation	Employment…………………1<br>House wife/unemployed……..2		
**11.**	How much do you earn monthly in shillings?	<50,000/=…………………..…1<br>50,000-<500,000/=……………..2<br>500,000-<1,000,000/=………….3<br>>1,000,000/=………………...4		
**12.**	How far is your residence from the health facility?	< 5 kilometer………………….1<br>5-10 Km………………………2<br>11-15 Km……………………..3<br>16-20Km………………………4<br>>20km…………………………5		
**13.**	Do you smoke?	Yes ……………………1<br>No…………………….......2		
**14.**	Do you use any substances of Abuse?	Yes……………………1<br>No…………………….2		**If No, skip to 16**

**15.**	Specify any the following that you use?	Alcohol…………………..1<br><br>Cocaine……………………2<br><br>Cannabis…………………3<br><br>Marijuana………………..4<br><br>Other (specify)……………5		
**16.**	Marital Status	Married………………………..1<br><br>Co-habiting……………………2<br><br>Single…………………………..3<br><br>Divorced………………………..4		
**17.**	Number of Sexual partners	1……………………………….1<br><br>2-3…………………………….2<br><br>4-5……………………………..3<br><br>>5……………………………..4		
**18.**	Were you accompanied by your spouse to the clinic?	Yes……………………………1<br><br>No…………………………….2		
**19.**	Does your spouse approve you to use post abortion contraception?	Yes………………………………<br>1<br><br>No………………………………2<br>.		**If No, skip to 21**

**20.**	Which method does he/she approve?	Condoms………………………..1<br>Copper IUD……………………2<br>Levonorgestrel IUS……………..3<br>Implants………………………..4<br>Pills…………………………….5<br>Injectables…………………….6<br>Withdraw……………………….7<br>Natural periodic abstinence…..8<br>Others (specify)………………..9		
**21.**	Do you have any post abortion contraceptive method of preference	Yes……………………………1<br>No…………………………….2		**If No, skip to 23**
**22.**	Specify the method of preference	Condoms………………………..1<br>Copper IUD……………………2<br>Levonorgestrel IUS……………..3<br>Implants………………………..4<br>Pills…………………………….5<br>Injectables…………………….6<br>Withdraw……………………….7<br>Natural periodic abstinence…..8		

		Others (specify)………………..9		
**23.**	Have you ever used any of the contraceptive methods before?	Yes……………………………… 1<br>No……………………………….. 2		
**24.**	Did you receive post abortion contraceptive counseling?	Yes……………………………..1<br>No………………………………2		**If No, Skip to 26**
**25.**	Has the post abortion counseling influenced the method of choice you have taken up?	Yes ……………………………1<br>No……………………………..2		
**26.**	Did you get any contact of any health worker in case you needed more clarity on any of the methods?	Yes……………………………… …1<br>No……………………………….. 2		
**27.**	Which of the methods have you used before?	Condoms………………………..1<br>Copper IUD…………………….2		

		Levonorgestrel IUD……………..3<br><br>Implants………………………..4<br><br>Pills…………………………….5<br><br>Injectables…………………….6<br><br>Withdraw………………………7<br><br>Natural periodic abstinence…..8<br><br>Others (specify)………………..9		
**28.**	What were the reasons for not using modern contraception before?	I felt too young to use the methods……………………1<br><br>My friends discouraged me from using the methods…………………2<br><br>I feared that the methods could make me barren………………………….3<br><br>The health care providers discouraged me from using the methods…………………………. 4<br><br>My partner refused me from using the method………………………5		

		I feared the side effects from the contraceptive methods………….6<br>My religion discourages me from using the available methods…….7<br>Others (specify)……………………………8		
**29.**	HIV status	Positive……………………1<br>Negative…………………..2		**If No, skip to 31**
**30.**	Does your HIV status affect the choice of contraception you want to use after the evacuation?	Yes…………………………1<br>No………………………….2		
**31.**	Which method would you prefer to use because of your HIV status?	Condoms………………………..1<br>Copper IUD…………………….2<br>Levonorgestrel IUS……………..3<br>Implants………………………..4<br>Pills…………………………….5<br>Injectables…………………….6<br>Withdraw………………………7		

		Natural periodic abstinence…..8<br>Others (specify)………………..9		
**32.**	Did the healthcare provider influence your choice of contraception?	Yes…………………………1<br>No……………………………2		**If No, skip to 34**
**33.**	How did the health workers affect your choice of contraception?	They made me to change from one choice to another…………1<br>They told me they couldn't insert my method of choice……………2<br>My choice wasn't available at the health facility……………….3<br>They told me my method of choice has many side effects…..4<br>I couldn't pay for my method of choice………………………..5<br>Other (specify)……………….6		
**34.**	Why did you opt to use post abortion intrauterine contraception?	I didn't want to conceive for at least 1 year……………………1<br>It was the only available option…………………………2<br>My friends encouraged me to use the method……………………3		

		I have used this method before and it was fine by me…………4<br><br>The health workers convinced me to use the method……………..5<br><br>My partner approved me to use it ………………………………6<br><br>The method was available for free……………………………..7<br><br>Other (specify)…………………8		
**35.**	Did you have to move from the place you had the abortion care to another place or facility to have your intrauterine device inserted?	Yes…………………………1<br><br>No…………………………..2		
**36.**	Would you use this post abortion intrauterine contraception in future?	Yes …………………………1<br><br>No…………………………….2		
**37.**	Would you recommend a friend to use this method?	Yes……………………………1<br><br>No……………………………..2		

**38.**	Does your partner approve you to use intrauterine contraception?	Yes……………………….1<br>No………………………..2<br>He/ She doesn’t know that I use the method…………………3		

APPENDIX XVIII: QUALITATIVE HEALTH WORKERS’ INTERVIEW GUIDE:

Aim: We seek to better understand the perception of Healthcare providers towards the provision of intrauterine contraception after medical management of first trimester incomplete abortion with particular emphasis on barriers and facilitators of the intrauterine contraception.

Research questions:

1. How do Healthcare providers perceive post abortion contraceptive counseling and provision?
2. How do Healthcare providers perceive post abortion long acting reversible contraceptive provision, IUD in particular?
3. What are the most important barriers to post abortion long acting reversible contraceptive provision and uptake?
4. What are the most important facilitators to post abortion long acting reversible contraceptive provision and uptake?

Interview Guide questions:

1. Please tell me a little about yourself (Age, education background, marital status, religious affiliation, occupation, number of children, health facility you work at, number of years at the facility, role in contraceptive provision etc.)
2. What has been your experience of providing post abortion intrauterine contraceptives

after medical management of first trimester incomplete abortion?

3. Has it been any different from the intrauterine contraceptive uptake after surgical evacuation? How different or not different has it been?
4. Do you think it is right to recommend post abortion intrauterine contraceptives within the first week as compared to 2-4 weeks after medical management of first trimester miscarriages? Why/Why not?
5. Do you think introduction of the insertion one week after the medical evacuation as compared with 2-4 weeks post evacuation will improve the uptake of post abortion intrauterine contraception among women seeking post abortion services? Why do you think so?
6. What barriers have you faced before this current study while offering post abortion counselling and intrauterine contraception thereafter?
7. What are the most important facilitators/motivators to your provision of post abortion intrauterine contraception?
8. What have been the main challenges that you have faced as you provide post abortion contraception? Probe for staffing, inadequate skill set for the service delivery, stock outs and supply chain for post abortion contraceptives as well as patient related challenges.
9. What recommendations would you give to improve the uptake of post abortion intrauterine contraception?
10. Any other comment.

Thank you

APPENDIX XIX: QUALITATIVE WOMEN AND THEIR SPOUSES' INTERVIEW GUIDE:

Aim: To explore the perception of women and their spouses on post abortion contraceptive uptake and use, LARC in particular. Specifically we want to gain a deeper understanding of post abortion family planning and contraceptive use, as well as how women who have chosen to use an IUC, navigate socio-cultural factors and gender norms and power relations that discourage use of the IUC.

Research questions:

1. What are women's and their spouses' perceptions surrounding LARC and the IUD in particular?
2. Considering the many socio-cultural beliefs and practices, gender norms and power relations that discourage modern contraceptive use, how do women who have chosen to initiate mPAC IUD assert their agency and negotiate use?
3. How do current social and gender norms including norms about sexuality and reproduction influence reproductive agency in relation to contraceptive use?

Interview Guide questions:

1. Please tell me a little about yourself (Age, education background, marital status, religious affiliation, occupation, number of children, where you stay, number of times they have sought post abortion services and post abortion intrauterine contraception etc.)
2. What has been your experience of post abortion intrauterine contraceptives after medical management of first trimester incomplete abortion?
3. Have you used such a method before? Explain when and source?
4. Do you think it is right to use post abortion intrauterine contraceptives within the first week as compared to 2-4 weeks after medical management of first trimester miscarriages? Why do you think so?
5. Do you think introduction of the insertion one week after the medical evacuation as compared with 2-4 weeks post evacuation will enable you to prevent unnecessary pregnancies in future? Why or why not?
6. What fears do you and your spouse have about post abortion intrauterine contraception?
7. Do you think the post abortion counselling given prior to choosing this type of contraception contributed in any way to the choice you are using? If ye why? If no why?
8. What did you like out the post abortion contraceptive counselling prior to taking on the intrauterine contraception?
9. What did you dislike about the post abortion contraceptive counselling prior to taking on the intrauterine contraception?
10. What are the most important facilitators/motivators that you and your spouse considered so as to take on post abortion intrauterine contraception?
11. What have been the main challenges that you have faced as you and your spouse took on post abortion intrauterine contraception?

    Probe for peer influence, inadequate skill set among the health workers, stock outs, and long distances to travel, cultural influence, power struggles on decision making, in regards to post abortion contraceptives.
12. What has been your experience about the impact of your culture on the choice of post

abortion contraception you chose to use?

Probe about the impact of norms, religious beliefs, spouse and relatives' influence on the women's choice of intrauterine contraception

How have you and your spouse managed to negotiate you way into using post abortion intrauterine contraception despite facing all the mentioned obstacles

13. What recommendations would you give to friends about post abortion intrauterine contraception?

APPENDIX XX: ELIGIBILITY SCREENING

MPAC IUD: RANDOMISED CONTROLLED NON-INFERIORITY TRIAL

YES NO Treated for incomplete abortion with Misoprostol

YES NO Uterine size at time of treatment <12 weeks

YES NO Age 15 years or older

YES NO Willing to have an IUD inserted following treatment?

IF **YES** to ALL → **Move on with screening**

YES NO Has had a surgical procedure to remove products of conception (this current pregnancy)

YES NO Fever >38.5 degrees Celcius

YES NO Suspected pelvic inflammatory disease

YES NO Ectopic pregnancy

YES NO Confirmed breast cancer

YES NO Cervicitis or endometritis

YES NO Confirmed cervical dysplasia

YES NO Known allergy towards copper

YES NO Known allergy towards Levonorgestrel

YES NO Confirmed or suspected uterine abnormality

YES NO Presence of any unexplained abnormal uterine bleeding

IF **YES** to any of these → **DO NOT INCLUDE**

APPENDIX XXI: CASE REPORT FORM (CRF)

MPAC IUD: A RANDOMISED CONTROLLED NON-INFERIORITY TRIAL COMPARING EARLY VERSUS STANDARD INSERTION OF INTRAUTERINE CONTRACEPTION AFTER FIRST TRIMESTER INCOMPLETE ABORTION IN CENTRAL UGANDA

CRF PART I: TREATMENT AND ENROLMENT VISIT

SECTION I: PARTICIPANT INFORMATION

A. STUDY SITE: _____________

B. STUDY ID:________________

1. **RANDOMISED TO (please circle study arm)**

   a) • EARLY IUD INSERTION (within one week)

   b) • STANDARD IUD INSERTION (2–4 weeks post medical abortion care)

2. **Today's date: ___:___:_____ (date/month/year)**

3. **Participant initials:_____**

SECTION II: SOCIO-DEMOGRAPHIC BACKGROUND

4. **How old are you? Years: _________**

5. **Are you currently married or in a relationship?**

a) • Yes, currently married

b) • Yes, currently in a relationship and living together

c) • Yes, currently in a relationship but not living together

d) • No, currently not in a relationship/never married

e) • No, I am divorced/separated

f) • No, I am widowed

6. **What is the highest level of school you have attended?**

a) • Never attended school

b) • Incomplete primary school (1st-7th grade)

c) • Complete primary school (1st-7th grade)

d) • Incomplete secondary school (1st-5th year)

e) • Complete secondary school (1st-6th year)

f) • University or Tertiary institution?

7. **What religion do you belong to?**

a) • Catholic

b) • Protestant

c) • Muslim

d) • Born again Christian

e) • Other (specify)……………………….

8. **What is your current employment status?**

a) • Unemployed looking for work

b) • Employed full time

c) • Employed part-time

d) • Self-employed

e) • Full time student

9. **What is your current average earning per month (salary and/or benefits)?**

a) • <50,000/=

b) • 50,000- <500,000/=

c) • 500,000-<1,000,000/=

d) • >1,000,000/=

SECTION III: REPRODUCTIVE HISTORY

10. **How many times have you been pregnant in your life, including any pregnancies that were not carried to term? (Including this current pregnancy)**

Number:________

11.1 **Have you ever had a pregnancy that ended in miscarriage?**

a) • Yes

b) • No (Go to Q12.1)

11.2 **How many times in your life have you experienced a miscarriage? Number:_____**

12.1 **Have you ever had an ectopic pregnancy?**

a) • Yes

b) • No (go to Q13.1)

12.2 **How many times in your life have you experienced an ectopic pregnancy? Number:________**

13.1 **Have you ever had a pregnancy that ended in induced abortion?**

a) • YES

b) • NO (Go to Q14.1)

13.2. **How many times in your life have you had an induced abortion? Number:______**

14.1 **How many times have you given birth in your life? Number:________**(If zero then go to Q15)

14.2 **How many of these births have been by caesarean section? Number:________**

14.3. **When was your most recent birth?**

a) • Within 6 months

b) • > 6–12 months

c) • >12–24 months

d) • >2 years

15. **Have you used any method of contraception in the past or ever done something to delay or avoid pregnancy?**

a) • YES

b) • NO, never (go to Q18)

16. **What contraceptive methods have you used/what have you done to delay or avoid pregnancy <u>in the past 12 months</u>? (Tick all that apply)**

a) • Male condom

b) • Female condom

c) • Pill

d) • Implant

e) • Hormonal IUD

f) • Copper IUD

g) • Injectable

h) • Vaginal ring

i) • Hormonal patch

j) • Emergency contraception

k) • Diaphragm or cervical cap

l) • Foam or jelly (spermicide)

m) • Withdrawal

n) • Calendar method (such as rhythm method or standard days' method)

o) • Lactation amenorrhea

p) • Fertility awareness approaches (such as cycle beads, billings ovulation method or use of devices to predict the fertile period)

q) • Female sterilization (tubes tied)

r) • Male sterilization (vasectomy)

s) • Abstinence

17. **What contraceptive method/s were you using to prevent a pregnancy at the time of conception (current pregnancy)?**

a) • Male condom

b) • Female condom

c) • Pill

d) • Implant

e) • Hormonal IUD

f) • Copper IUD

g) • Injectable

h) • Vaginal ring

i) • Hormonal patch

j) • Emergency contraception

k) • Diaphragm or cervical cap

l) • Foam or jelly (spermicide)

m) • Withdrawal

n) • Rhythm method

o) • Lactation amenorrhea

p) • Abstinence

q) • Female sterilization (tubes tied)

r) • Male sterilization (vasectomy)

18. **Are you currently breastfeeding?**

a) • Yes

b) • No

19. **What is your HIV status?**

a) • I am positive and on treatment

b) • I am positive but not on treatment

c) • I am negative

d) • I don't know (offer testing)

Regarding this current pregnancy:

20**. At the time of conception, did you want to become pregnant/had you planned to wait until later to become pregnant or did you not want to have any (more) children?**

a) • I wanted to become pregnant then

b) • I had planned to wait until later

c) • I did not want to have any children/any more children

21. **What happened with this pregnancy you are seeking care in relation to?**

a) • I had a miscarriage

b) • I had an induced abortion

SECTION IV: GESTATIONAL AGE AT TIME OF MISOPROSTOL TREATMENT

22. **On the day of Misoprostol treatment, how was the gestational age (length of pregnancy) determined?** *Tick all that apply and specify the length of the pregnancy in weeks*

a) • First day of last menstrual period, weeks: ______

b) • Obstetric ultrasound scan, weeks: _______

c) • Bi- manual palpation, weeks: ______

23. **Date scheduled for insertion visit: ___:___:_____ (date/month/year)**

Intervention group*: Insertion visit should be <7 days of the treatment visit*

Comparison group: *Insertion visit should be 2–4 weeks following the treatment visit*

<u>CRF PART II: INSERTION VISIT</u>

EARLY INSERTION GROUP <7 DAYS vs STANDARD INSERTION GROUP 2–4 WEEKS

24. **Did the participant attend this insertion visit?**

a) • YES

b) • NO (go to the End of Trial Form (at the end of the questionnaire)

SECTION I: PARTICIPANT INFORMATION:

A. STUDY SITE: _____________

B. STUDY ID:________________

25. **RANDOMISED TO (please circle study arm**)

a) • EARLY INSERTION

b) • STANDARD INSERTION

26. **Today's date: __:__:____ (date/month/year)**

27. **Number of weeks/days since first visit (day of treatment) to this facility:**

______Weeks ______days

SECTION II: SYMPTOMS SINCE TREATMENT WITH MISOPROSTOL

28. **Have you seen products of conception passed? (including larger blood clots that may or may not have been actual product of conception)**

a) • Yes

b) • No

c) • Unsure

29. **How much have you bled since treatment at this facility?**

a) • Less than a normal menstrual bleeding

b) • Same as a normal menstrual bleeding

c) • Somewhat more than a normal menstrual bleeding

d) • A lot heavier than a normal menstrual bleeding

30. **Since treatment have you experienced…?**

a) • Foul smelling vaginal discharge

b) • Fever

c) • Severe abdominal pain

d) • Heavy vaginal bleeding

e) • Other symptoms, specify:

31. **What is the highest level of pain you have experienced since treatment?**

Visual analogue scale

0 (no pain)____10 (worst possible pain)

32. **What is the level of pain you are currently experiencing?**

Visual analogue scale

0 (no pain)____10 (worst possible pain)

SECTION III: UNSCHEDULED VISITS

33.1 **Have you had any unscheduled visits since treatment at this facility?**

a) • Yes

b) • No (skip to Q34)

33.2 **What was the main reason for this unscheduled visit?**

a) • Heavy bleeding

b) • Abdominal pain

c) • Fever

d) • Other reason, please specify

33.3 **Have you received any additional treatment to empty the uterus apart from the initial treatment with Misoprostol, since your first visit to this facility?**

a) • No

b) • Yes, additional Misoprostol doses

c) • Yes, surgical evacuation, MVA (manual vacuum aspiration)

d) • Yes, surgical evacuation, curettage

e) • Other, please specify

SECTION IV: SYMPTOMS AND CLINICAL FINDINGS FROM PELVIC EXAMINATION

34. **Body temperature in degrees Celsius:________**

35. **Uterine size according to bimanual palpation (91):_______**

36. **Clinical findings at pelvic exam**

a) • Uterine tenderness

b) • Heavy bleeding

c) • Foul smelling discharge

d) • Severe abdominal pain

e) • None of the above

f) • Other, please specify: ____

37. **Based on your clinical examination, was the abortion complete?**

a) • Yes (Go to Q39.1)

b) • No

38. **If no, how was the incomplete abortion managed?**

b) • Re-evacuated using MVA

c) • Re-evacuated using additional Misoprostol

d) • Re-evacuated using curettage

e) • Expectant management

SECTION V: IUD INSERTION–HEALTHCARE PROVIDER EXPERIENCE OF INSERTING THE IUD (including unsuccessful and successful attempts)

39.1 **Was an ultrasound conducted prior to insertion?**

a) • YES (go to Q39.2)

b) • NO (go to Q40.1)

39.2 **If yes, what were the clinical findings from the ultrasound?**

a) • Retained foetal parts

b) • No foetal parts/endometrial thickness <30 millimeters

c) • No foetal parts/Endometrial thickness >30 millimeters

40.1 **Did you attempt to insert an IUD?**

a) • YES (go to Q40.3)

b) • NO

40.2 **What was the main reason for not attempting the IUD insertion?**

a) • There were signs of pelvic infection

b) • The participant was in too much pain

c) • Because of heavy bleeding

d) • Because an ultrasound showed that the pregnancy has not yet been expelled (i.e. foetal parts remained)

e) • The participant changed her mind about having IUD inserted

f) • Other reason, please specify

If there were no attempts to insert the IUD go to Q45 after filling in Q40.2

40.3 **Was the insertion attempt successful?**

a) • YES

b) • NO

40.4 **How many attempts were made to insert an IUD? Number:_______**

40.5 **How would you rate the ease of insertion?**

a) • Very easy

b) • Somewhat easy

c) • Difficult

d) • Extremely difficult

41.1 **Did you provide/had the patient taken pain medication prior to insertion?**

a) • YES

b) • NO (go to Q42)

41.2 **What pain medication/s did the patient take/was provided prior to insertion? (tick all that apply)**

a) • Ibuprofen

b) • Paracetamol

c) • Local anesthetic

d) • Diclofenac

e) • Opioids

f) • Other, please specify

SECTION VI: IUD INSERTION – PARTICIPANT´S EXPERIENCE OF THE INSERTION (including unsuccessful and successful attempts)

42. **Did you feel safe and calm at time of insertion?**

a) • Yes

b) • No

43. **How did you perceive the insertion?**

a) • Easier than expected

b) • As expected

c) • Worse than expected

44. **What was the level of pain you experienced during insertion?**

Visual analogue scale

0 (no pain)____10 (worst possible pain)

SECTION VII: CONTRACEPTIVE USE FOLLOWING THIS VISIT

45 **What method of contraception did the study participant *leave with*?**

Indicate the method the study participant left with i.e. the method she initiated during this visit.

Please note that 'initiate a method' means that a method is administrated, inserted or prescribed (pills, implant, IUD, patch, vaginal ring, emergency contraception, injectable) OR she plans to use it during next intercourse (condom, diaphragm, foam or jelly) OR sterilization is scheduled. If none of these apply then tick 'left without a method'.

a) • Male condom

b) • Female condom

c) • Pill

d) • Implant

e) • Hormonal IUD

f) • Copper IUD

g) • Injectable

h) • Vaginal ring

i) • Hormonal patch

j) • Emergency contraception

k) • Diaphragm or cervical cap

l) • Foam or jelly (spermicide)

m) • Scheduled for female sterilization (tubes tied)

n) • Left without a method

Date scheduled for next visit: ___:___:_____ (date/month/year)

Intervention group: Next visit should be within 2–4 weeks follow the treatment visit

Comparison group: Next visit should be 3 months following the treatment visit

CRF PART III: OUTCOME OF MISOPROSTOL TREATMENT (2–4 WEEKS FOLLOWING TREATMENT) – INTERVENTION GROUP ONLY!

Please note that this visit is for the intervention group only as the misoprostol treatment outcome is assessed at the insertion visit for those in the comparison group

46. **Did the participant attend this visit to assess treatment outcome?**

a) • YES

b) • NO (go to the End of Trial form at the end of the questionnaire, page)

SECTION I: PARTICIPANT INFORMATION

A. STUDY SITE: _____________

B. STUDY ID:________________

47. **RANDOMISED TO (please circle study arm)**

a) • EARLY INSERTION

b) • STANDARD INSERTION

48. **Today's date: __:__:____ (date/month/year)**

SECTION II: CURRENT CONTRACEPTIVE USE

49.1 **Are you currently using any method of modern contraception?**

Modern contraception include: condom, pill, implant, IUD, vaginal ring, hormonal patch, foam or jelly, diaphragm or cervical cap, emergency contraception

a) • YES

b) • NO (go to Q50.1)

49.2 **What method/s of modern contraceptive are you currently using? (tick all that apply)**

a) • Male condom

b) • Female condom

c) • Pill

d) • Implant

e) • Hormonal IUD

f) • Copper IUD

g) • Injectable

h) • Vaginal ring

i) • Hormonal patch

j) • Emergency contraception

k) • Diaphragm or cervical cap

l) • Foam or jelly (spermicide)

m) • Female sterilization (tubes tied)

n) • Male sterilization (vasectomy)

49.3 **What is your level of satisfaction with the method/s you are currently using?**

a) • Highly satisfied

b) • Somewhat satisfied

c) • Somewhat unsatisfied

d) • Highly unsatisfied

49.4 **Have you experienced any side effects since the IUD was inserted/other method of contraception was initiated?**

a) • No side effects (go to Q50.1)

b) • Heavy menstruation

c) • Painful menstruation

d) • Irregular menstruation

e) • Continuous bleeding/spotting

f) • Intermittent spotting

g) • No menstruation

h) • Cramping/pelvic pain

i) • Acne

j) • Headaches

k) • Mood swings

l) • Vaginal dryness

m) • Decrease in libido

n) • Other, please specify

49.5 **How would you describe these side effects?**

a) • Mild and tolerable

b) • Just tolerable

c) • Intolerable

SECTION III: UNSCHEDULED VISITS

50.1 **Did you have an unscheduled visit since your last visit to this facility?**

a) • YES

b) • NO (go to Q51)

50.2 **What was the main reason for the unscheduled visit?**

a) • Heavy bleeding

b) • Abdominal pain

c) • Fever

d) • IUD came out by itself

e) • Painful sexual intercourse

f) • Other reason, please specify

50.3 **Was the IUD removed at this unscheduled visit?**

a) • Yes

b) • No it was left inside uterus (go to Q51)

c) • No, there was no IUD to remove (go to Q50.5)

50.4 **What were the main reason/s for removing the IUD at the unscheduled visit?**

a) • Partial expulsion (IUD in cervix)

b) • Signs of pelvic infection

c) • Pregnancy - intrauterine

d) • Pregnancy - ectopic

e) • Painful intercourse

f) • Heavy menstruation

g) • Painful menstruation

h) • Irregular menstruation

i) • Continuous bleeding/spotting

j) • Intermittent spotting

k) • No menstruation

l) • Cramping/pelvic pain

m) • Acne

n) • Headaches

o) • Mood swings

p) • Vaginal dryness

q) • Decreased libido

r) • Partner disapproved

s) • Wanted to become pregnant

t) • Other, please specify:

50.5 **Was the IUD replaced with a new one at the unscheduled visit?**

a) • YES

b) • NO

SECTION IV: SYMPTOMS AND CLINICAL FINDINGS FROM PELVIC EXAMINATION (ALL STUDY PARTICIPANTS)

51. **Bleeding since last visit to this facility**

a) • Less than a normal menstrual bleeding

b) • Same as a normal menstrual bleeding

c) • Somewhat more than a normal menstrual bleeding

d) • A lot heavier than a normal menstrual bleeding

The following questions are answered <u>by the health care staff after examination</u> of the study participant

52. **At clinical examination, did the study participant have any of the following symptoms?**

a) • Uterine tenderness

b) • Heavy bleeding

c) • Foul smelling discharge

d) • Severe abdominal pain

e) • None of the above

f) • Other please specify

53.1 **Based on your clinical examination, was the abortion complete?**

a) • YES (go to Q54)

b) • NO

53.2 **If no, how was the incomplete abortion managed?**

a) • Re-evacuated using MVA

b) • Re-evacuated using additional Misoprostol

c) • Re-evacuated using curettage

d) • Expectant management

54. **Did the study participant have an IUD inserted at her last visit to this facility? (see Q45 if unsure)**

a) • YES

b) • NO (go to Q58.1)

55. **What did the clinical examination show?**

a) • IUD in uterus - threads were visible/palpable

b) • IUD in cervix – threads/IUD were visible/palpable

c) • No IUD in cervix or uterus

d) • Unsure (proceed with ultrasound if possible)

56.1 **Was an ultrasound conducted to determine if the IUD was correctly placed?**

a) • Yes

b) • No (go to Q57.1)

56.2 **What did the ultrasound show?**

a) • IUD in situ (intra uterine)

b) • IUD partially expelled (in cervix)

c) • There was no IUD in uterus or cervix (IUD expelled)

d) • IUD perforation, partial

e) • IUD perforation, total

57.1 **Was the IUD removed at this visit?**

a) • Yes

b) • No (go to Q58.1)

57.2 **What was the main reason for removing the IUD at this visit?**

a) • Partial expulsion (IUD in cervix)

b) • Signs of pelvic infection

c) • Pregnancy - intrauterine

d) • Pregnancy - ectopic

e) • Painful intercourse

f) • Heavy menstruation

g) • Painful menstruation

h) • Irregular menstruation

i) • Continuous bleeding/spotting

j) • Intermittent spotting

k) • No menstruation

l) • Cramping/pelvic pain

m) • Acne

n) • Headaches

o) • Mood swings

p) • Vaginal dryness

q) • Decreased libido

r) • Partner disapproved

s) • Wanted to become pregnant

t) • Other, please specify:

SECTION V: CONTRACEPTIVE USE FOLLOWING THIS VISIT

58.1 **Did the study participant change OR initiate a new contraceptive method during this visit?**

Please note that 'initiate a method' means that a method is administrated, inserted or prescribed (pills, implant, IUD, patch, vaginal ring, emergency contraception, injectable) OR she plans to use it during next intercourse (condom, diaphragm, foam or jelly) OR sterilization is scheduled.

a) • YES

b) • NO

58.2 **What method of contraception did the study participant *leave with*?**

Indicate the method the study participant left with i.e. the same method she used when arriving for this visit OR the method she initiated during this visit. If none of these apply then tick 'left without a method'.

a) • Male condom

b) • Female condom

c) • Pill

d) • Implant

e) • Hormonal IUD

f) • Copper IUD

g) • Injectable

h) • Vaginal ring

i) • Hormonal patch

j) • Emergency contraception

k) • Diaphragm or cervical cap

l) • Foam or jelly (spermicide)

m) • Scheduled for female sterilization (tubes tied)

n) • Left without a method

59. **Date scheduled for next visit: ___:___:_____ (date/month/year)**

Next visit should be 3 months follow the treatment visit

<u>CRF Part IV: THREE–MONTH FOLLOW UP</u>

60. **Did the participant attend this 3-month visit?**

a) • YES

b) • NO (go to the End of Trial form at the end of the questionnaire)

SECTION I: PARTICIPANT INFORMATION

A. STUDY SITE: _____________

B. STUDY ID:________________

61. **RANDOMISED TO (please circle study arm)**

a) • EARLY INSERTION

b) • STANDARD INSERTION

62. **Today's date: ___:___:_____ (date/month/year)**

SECTION II: PREGNANCIES - ALL STUDY PARTICIPANTS

63. **Have you had unprotected sex since your last visit to this facility?**

a) • YES

b) • NO

64. **Have you experienced any pregnancies since the last visit to this facility? Number:____**

(If 0 pregnancies go to Q 69.1)

65. **Have you experienced any miscarriages since last visit to this facility? Number:____**

66. **Have you had any induced abortions since last visit to this facility? Number:_____**

67. **Have you experienced any ectopic pregnancies since last visit to this facility? Number:____**

68. **Are you currently pregnant?**

a) • YES

b) • NO

SECTION III: UNSCHEDULED VISITS

69.1 **Did you have an unscheduled visit since your last visit to this facility?**

a) • YES

b) • NO (go to SECTION IV, Q70.1)

69.2 **What was the main reason for the unscheduled visit?**

a) • Abdominal pain

b) • Vaginal bleeding

c) • IUD came out by itself

d) • Painful sexual intercourse

e) • Other reason, please specify

69.3 **Was the IUD removed at this unscheduled visit?**

a) • Yes

b) • No, it was left inside the uterus (go to Q70.1)

c) • No, there was no IUD to remove (go to Q69.5)

69.4 **What were the main reasons for removing the IUD at the unscheduled visit? (tick all that apply)**

a) • Partial expulsion (IUD in cervix)

b) • Signs of pelvic infection

c) • Pregnancy - intrauterine

d) • Pregnancy - ectopic

e) • Painful intercourse

f) • Heavy menstruation

g) • Painful menstruation

h) • Irregular menstruation

i) • Continuous bleeding/spotting

j) • Intermittent spotting

k) • No menstruation

l) • Cramping/pelvic pain

m) • Acne

n) • Headaches

o) • Mood swings

p) • Vaginal dryness

q) • Decrease in libido

r) • Partner disapproved

s) • Wanted to become pregnant

t) • Other, please specify:

69.5 **Was the IUD replaced with a new one at the unscheduled visit?**

a) • YES

b) • NO

SECTION IV: CURRENT CONTRACEPTIVE USE

70.1 **Are you currently using any method of modern contraception?**

Modern contraceptive include: condom, pill, implant, IUD, vaginal ring, hormonal patch, foam or jelly, diaphragm or cervical cap, emergency contraception

a) • YES

b) • NO (go to Q74.1, SECTION VI)

70.2 **What contraceptive method/s are you currently using? (tick all that apply)**

a) • Male condom

b) • Female condom

c) • Pill

d) • Implant

e) • Hormonal IUD

f) • Copper IUD

g) • Injectable

h) • Vaginal ring

i) • Hormonal patch

j) • Emergency contraception

k) • Diaphragm or cervical cap

l) • Foam or jelly (spermicide)

m) • Female sterilization (tubes tied)

n) • Male sterilization (vasectomy)

70.3 **What is your level of satisfaction with the method/s you are currently using?**

a) • Highly satisfied

b) • Somewhat satisfied

c) • Somewhat unsatisfied

d) • Highly unsatisfied

70.4 **Are you experiencing any side effects from the contraceptive method/s you are currently using? (tick all that apply)**

a) • No side effects (go to Q71)

b) • Heavy menstruation

c) • Painful menstruation

d) • Irregular menstruation

e) • Continuous bleeding/spotting

f) • Intermittent spotting

g) • No menstruation

h) • Cramping/pelvic pain

i) • Acne

j) • Headaches

k) • Mood swings

l) • Vaginal dryness

m) • Decrease in libido

n) • Other, please specify

70.5 **How would you describe these side effects?**

a) • Mild and tolerable

b) • Just tolerable

c) • Intolerable

SECTION V: FOR STUDY PARTICIPANTS WHO REPORT THAT THEY ARE CURRENTLY USING AN IUD (Non-IUD users go to section VI below)

The following questions are answered by the health care staff after examination of the study participant

71. **What did the clinical examination show?**

a) • IUD in uterus - threads were visible/palpable

b) • IUD in cervix – threads/IUD were visible/palpable

c) • No IUD in cervix or uterus

d) • Unsure (proceed with ultrasound if possible)

72.1 **Was an ultrasound conducted to confirm if the IUD was correctly placed in the uterus?**

a) • YES

b) • NO (go to Q73.1)

72.2 **What did the ultrasound show?**

a) • IUD in situ (intra uterine)

b) • IUD partially expelled (in cervix)

c) • There was no IUD in uterus or cervix (IUD expelled)

d) • IUD perforation, partial

e) • IUD perforation, total

73.1 **Was the IUD removed at this visit?**

a) • YES

b) • NO (go to question 75.1)

73.2 **What was the main reason for removing the IUD at this visit?**

a) • Partial expulsion (IUD in cervix)

b) • Signs of pelvic infection

c) • Pregnancy - intrauterine

d) • Pregnancy - ectopic

e) • Painful intercourse

f) • Heavy menstruation

g) • Painful menstruation

h) • Irregular menstruation

i) • Continuous bleeding/spotting

j) • Intermittent spotting

k) • No menstruation

l) • Cramping/pelvic pain

m) • Acne

n) • Headaches

o) • Mood swings

p) • Vaginal dryness

q) • Decrease in libido

r) • Partner disapproved

s) • Wanted to become pregnant

t) • Other, please specify:

SECTION VI: FOR STUDY PARTICIPANTS WHO REPORT THAT THEY ARE NOT CURRENTLY USING AN IUD (at the time they arrive at the facility)

74.1 **How come you are currently not using an IUD?**

a) • The IUD was never inserted (go to Q75.1)

b) • I had the IUD removed and it was not replaced

c) • The IUD came out by itself and it was not replaced

74.2 **What were the main reason/s for removing/not replacing the IUD? (tick all that apply)**

a) • Partial expulsion (IUD in cervix)

b) • Signs of pelvic infection

c) • Pregnancy - intrauterine

d) • Pregnancy - ectopic

e) • Painful intercourse

f) • Heavy menstruation

g) • Painful menstruation

h) • Irregular menstruation

i) • Continuous bleeding/spotting

j) • Intermittent spotting

k) • No menstruation

l) • Cramping/pelvic pain

m) • Acne

n) • Headaches

o) • Mood swings

p) • Vaginal dryness

q) • Decrease in libido

r) • Partner disapproved

s) • Wanted to become pregnant

t) • Other, please specify:

SECTION VII: CONTRACEPTIVE USE FOLLOWING THIS VISIT - ALL STUDY PARTICIPANTS

75.1 **Did the study participant change OR initiate a new contraceptive method during this visit?**

Please note that 'initiate a method' means that the method is administrated, inserted or prescribed (pills, implant, IUD, patch, vaginal ring, emergency contraception, injectable) OR she plans to use it during next intercourse (condom, diaphragm, foam or jelly) OR sterilization is scheduled.

a) • YES

b) • NO

75.2 **What method of contraception did the study participant *leave with*?**

Indicate the method the study participant left with i.e. the same method she used when arriving for this visit OR the method she initiated during this visit. If none of these apply then tick 'left without a method'.

a) • Male condom

b) • Female condom

c) • Pill

d) • Implant

e) • Hormonal IUD

f) • Copper IUD

g) • Injectable

h) • Vaginal ring

i) • Hormonal patch

j) • Emergency contraception

k) • Diaphragm or cervical cap

l) • Foam or jelly (spermicide)

m) • Scheduled for female sterilization (tubes tied)

n) • Left without a method

76. **Date scheduled for next visit: ___:___:_____ (date/month/year)**

Next visit should be 6 months after the first visit (treatment visit)

<u>CRF PART V: SIX–MONTH FOLLOW UP</u>

77. **Did the participant attend this 6-month visit?**

a) • YES

b) • NO (go to the End of Trial form at the end of the questionnaire)

SECTION I: PARTICIPANT INFORMATION

A. STUDY SITE: _____________

B. STUDY ID:________________

78. **RANDOMISED TO (please circle study arm)**

a) • EARLY INSERTION

b) • STANDARD INSERTION

79. **Today's date: ___:___:_____ (date/month/year)**

SECTION II: PREGNANCIES - ALL PARTICIPANTS

80. **Have you had unprotected sex since your last visit to this facility?**

a) • YES

b) • NO

81. **Have you experienced any pregnancies since last visit to this facility? Number**:___ _

(If 0 pregnancies go to Q86.1)

82. **Have you experienced any miscarriages since last visit to this facility? Number:____**

83. **Have you had any induced abortions since last visit to this facility? Number:_____**

84. **Have you experienced any ectopic pregnancies since last visit to this facility? Number:______**

85. **Are you currently pregnant?**

a) • YES

b) • NO

SECTION III: UNSCHEDULED VISITS

86.1 **Did you have an unscheduled visit since your last visit to this facility?**

a) • YES

b) • NO (go to Q87.1)

86.2 **What was the main reason for the unscheduled visit?**

a) • Abdominal pain

b) • Vaginal bleeding

c) • IUD came out by itself

d) • Painful sexual intercourse

e) • Other reason, please specify

86.3 **Was the IUD removed at this unscheduled visit?**

a) • Yes

b) • No, it was left inside the uterus (go to Q87.1)

c) • No, there was no IUD to remove (go to Q86.5)

86.4 **What were the main reasons for removing the IUD at the unscheduled visit? (tick all that apply)**

a) • Partial expulsion (IUD in cervix)

b) • Signs of pelvic infection

c) • Pregnancy - intrauterine

d) • Pregnancy - ectopic

e) • Painful intercourse

f) • Heavy menstruation

g) • Painful menstruation

h) • Irregular menstruation

i) • Continuous bleeding/spotting

j) • Intermittent spotting

k) • No menstruation

l) • Cramping/pelvic pain

m) • Acne

n) • Headaches

o) • Mood swings

p) • Vaginal dryness

q) • Decrease in libido

r) • Partner disapproved

s) • Wanted to become pregnant

t) • Other, please specify:

86.5 **Was the IUD replaced with a new one at the unscheduled visit?**

a) • YES

b) • NO

SECTION IV: CURRENT CONTRACEPTIVE USE

87.1 **Are you currently using any method of modern contraception?**

Modern contraceptives include: condom, pill, implant, IUD, vaginal ring, hormonal patch, foam or jelly, diaphragm or cervical cap, emergency contraception

a) • YES

b) • NO (skip to Q 91.1 SECTION V)

87.2 **What modern contraceptive method/s are you currently using? (tick all that apply)**

a) • Male condom

b) • Female condom

c) • Pill

d) • Implant

e) • Hormonal IUD

f) • Copper IUD

g) • Injectable

h) • Vaginal ring

i) • Hormonal patch

j) • Emergency contraception

k) • Diaphragm or cervical cap

l) • Foam or jelly (spermicide)

m) • Female sterilization (tubes tied)

n) • Male sterilization (vasectomy)

87.3 **What is your level of satisfaction with the method/s you are currently using?**

a) • Highly satisfied

b) • Somewhat satisfied

c) • Somewhat unsatisfied

d) • Highly unsatisfied

87.4 **Are you experiencing any side effects from the contraceptive method/s you are currently using? (Tick all that apply)**

a) • No side effects (go to Q88)

b) • Heavy menstruation

c) • Painful menstruation

d) • Irregular menstruation

e) • Continuous bleeding/spotting)

f) • Intermittent spotting

g) • No menstruation

h) • Cramping/pelvic pain

i) • Acne

j) • Headaches

k) • Mood swings

l) • Vaginal dryness

m) • Decrease in libido

n) • Other, please specify

87.5 **How would you describe these side effects?**

a) • Mild and tolerable

b) • Just tolerable

c) • Intolerable

SECTION V: FOR STUDY PARTICIPANTS WHO REPORT THAT THEY ARE CURRENTLY USING AN IUD (Non-IUD users go to section VI below)

88. **What did the clinical examination show?**

a) • IUD in uterus: - threads were visible/ palpable

b) • IUD in cervix – threads/IUD were visible/palpable

c) • No IUD in cervix or uterus

d) • Unsure (proceed with ultrasound if possible)

89.1 **Was an ultrasound conducted to confirm if the IUD was correctly placed in the uterus?**

a) • YES

b) • NO (go to Q90.1)

89.2 **What did the ultrasound show?**

a) • IUD in situ (intra uterine)

b) • IUD partially expelled (in cervix)

c) • There was no IUD in uterus or cervix (IUD expelled)

d) • IUD perforation, partial

e) • IUD perforation, total

90.1 **Was the IUD removed at this visit?**

a) • Yes

b) • No it was left inside the uterus (go to Q92.1)

c) • No there was no IUD to remove (go to Q92.1)

90.2 **What were the main reasons for removing the IUD? (Tick all that apply)**

a) • Partial expulsion (IUD in cervix)

b) • Signs of pelvic infection

c) • Pregnancy - intrauterine

d) • Pregnancy - ectopic

e) • Painful intercourse

f) • Heavy menstruation

g) • Painful menstruation

h) • Irregular menstruation

i) • Continuous bleeding/spotting

j) • Intermittent spotting

k) • No menstruation

l) • Cramping/pelvic pain

m) • Acne

n) • Headaches

o) • Mood swings

p) • Vaginal dryness

q) • Decreased libido

r) • Partner disapproved

s) • Wanted to become pregnant

t) • Other, please specify:

SECTION VI: FOR STUDY PARTICIPANTS WHO REPORT THAT THEY ARE NOT CURRENTLY USING AN IUD (at the time they arrive at the facility)

91.1 **How come you are currently not using an IUD?**

a) • The IUD was never inserted (go to Q92.1)

b) • I had the IUD removed and it was not replaced

c) • The IUD came out by itself and it was not replaced

91.2 **What were the main reason/s for removing/not replacing the IUD? (Tick all that apply)**

a) • Partial expulsion (IUD in cervix)

b) • Signs of pelvic infection

c) • Pregnancy - intrauterine

d) • Pregnancy - ectopic

e) • Painful intercourse

f) • Heavy menstruation

g) • Painful menstruation

h) • Irregular menstruation

i) • Continuous bleeding/spotting

j) • Intermittent spotting

k) • No menstruation

l) • Cramping/pelvic pain

m) • Acne

n) • Headaches

o) • Mood swings

p) • Vaginal dryness

q) • Decreased libido

r) • Partner disapproved

s) • Wanted to become pregnant

t) • Other, please specify:

SECTION IV: CONTRACEPTIVE USE FOLLOWING THIS VISIT - ALL STUDY PARTICIPANTS

92.1 **Did the study participant change OR initiate a new contraceptive method during this visit?**

Please note that 'initiate a method' means that a method is administrated, inserted or prescribed (pills, implant, IUD, patch, vaginal ring, emergency contraception, injectable) OR she plans to use it during next intercourse (condom, diaphragm, foam or jelly) OR sterilization is scheduled.

a) • YES

b) • NO

92.2 **What method of contraception did the study participant *leave with*?**

Indicate the method the study participant left with i.e. the same method she used when arriving for this visit OR the method she initiated during this visit. If none of these apply then tick 'left without a method'.

a) • Male condom

b) • Female condom

c) • Pill

d) • Implant

e) • Hormonal IUD

f) • Copper IUD

g) • Injectable

h) • Vaginal ring

i) • Hormonal patch

j) • Emergency contraception

k) • Diaphragm or cervical cap

l) • Foam or jelly (spermicide)

m) • Scheduled for female sterilization (tubes tied)

n) • Left without a method

93. **Date scheduled for next visit: ___:___:_____ (date/month/year)**

Next visit should be 12 months after the first visit (treatment visit)

<u>CRF PART VII: TWELVE–MONTH FOLLOW UP</u>

94. **Did the participant attend this 12-month follow up visit?**

a) • YES

b) • NO (go to the End of Trial form at the end of the questionnaire)

SECTION I: PARTICIPANT INFORMATION

A. STUDY SITE: _____________

B. STUDY ID:________________

95. **RANDOMISED TO (please circle study arm)**

a) • EARLY INSERTION

b) • STANDARD INSERTION

96. **Today's date: ____:____:_______ (date/month/year)**

SECTION II: PREGNANCIES - ALL STUDY PARTICIPANTS

97. Have you had unprotected sex since your last visit to this facility?

a) • YES

b) • NO

98. **Have you experienced any pregnancies since last visit to this facility? Number:____**

(If 0 then go to Section III, Q103.1)

99. **Have you experienced any miscarriages since last visit to this facility? Number:____**

100. **Have you had any induced abortions since last visit to this facility? Number:____**

101. **Have you experienced any ectopic pregnancies since last visit to this facility? Number:____**

102.1 **Have you given birth since your last visit to this facility?**

a) • YES

b) • NO

102.2 **Are you currently pregnant?**

a) • YES

b) • NO

SECTION III: UNSCHEDULED VISITS

103.1 **Did you have an unscheduled visit since your last visit to this facility?**

a) • Yes

b) • No (go to SECTION IV, Q104.1)

103.2 **What was the main reason for the unscheduled visit?**

a) • Abdominal pain

b) • Vaginal bleeding

c) • IUD came out by itself

d) • Painful sexual intercourse

e) • Other reason, please specify

103.3 **Was the IUD removed at this unscheduled visit?**

a) • Yes

b) • No, it was left inside the uterus (go to Q104.1)

c) • No, there was no IUD to remove (go to Q103.5)

103.4 **What were the main reasons for removing the IUD at the unscheduled visit?**

a) • Partial expulsion (IUD in cervix)

b) • Pelvic infection

c) • Pregnancy - intrauterine

d) • Pregnancy - ectopic

e) • Painful intercourse

f) • Heavy menstruation

g) • Painful menstruation

h) • Irregular menstruation

i) • Continuous bleeding/spotting

j) • Intermittent spotting

k) • No menstruation

l) • Cramping/pelvic pain

m) • Acne

n) • Headaches

o) • Mood swings

p) • Vaginal dryness

q) • Decreased libido

r) • Partner disapproved

s) • Wanted to become pregnant

t) • Other, please specify:

103.5 **Was the IUD replaced with a new one at the unscheduled visit?**

a) • YES

b) • NO

Section IV: CURRENT CONTRACEPTIVE USE

104.1 **Are you currently using any method of modern contraception?**

Modern contraceptives include: condom, pill, implant, IUD, vaginal ring, hormonal patch, foam or jelly, diaphragm or cervical cap, emergency contraception

a) • YES

b) • NO (go to Q105, SECTION V)

104.2 **What contraceptive method/s are you currently using? (Tick all that apply)**

a) • Male condom

b) • Female condom

c) • Pill

d) • Implant

e) • Hormonal IUD

f) • Copper IUD

g) • Injectable

h) • Vaginal ring

i) • Hormonal patch

j) • Emergency contraception

k) • Diaphragm or cervical cap

l) • Foam or jelly (spermicide)

m) • Female sterilization (tubes tied)

n) • Male sterilization (vasectomy)

104.3 **What is your level of satisfaction with the method/s you are currently using?**

a) • Highly satisfied

b) • Somewhat satisfied

c) • Somewhat unsatisfied

d) • Highly unsatisfied

104.4 **Are you experiencing any side effects from the contraceptive method/s you are currently using? (Tick all that apply)**

a) • No side effects (go to Q105)

b) • Heavy menstruation

c) • Painful menstruation

d) • Irregular menstruation

e) • Continuous bleeding/spotting

f) • Intermittent spotting

g) • No menstruation

h) • Cramping/pelvic pain

i) • Acne

j) • Headaches

k) • Mood swings

l) • Vaginal dryness

m) • Decrease in libido

n) • Other, please specify

104.5 **How would you describe these side effects?**

a) • Mild and tolerable

b) • Just tolerable

c) • Intolerable

SECTION V: FOR STUDY PARTICIPANTS WHO REPORT THAT THEY ARE CURRENTLY USING AN IUD (Non-IUD users go to question Q108.1 in section VI)

105 **What did the clinical examination show?**

a) • IUD in uterus - threads were visible/ palpable

b) • IUD in cervix – threads/IUD were visible/palpable

c) • No IUD in cervix or uterus

d) • Unsure (proceed with ultrasound if possible)

106.1 **Was an ultrasound conducted to confirm if the IUD was correctly placed in the uterus?**

a) • YES

b) • NO (go to Q107.1)

106.2 **What did the ultrasound show?**

a) • IUD in situ (intra uterine)

b) • IUD partially expelled (in cervix)

c) • There was no IUD in uterus or cervix (IUD expelled)

d) • IUD perforation, partial

e) • IUD perforation, total

107.1 **Was the IUD removed at this visit?**

a) • YES

b) • NO (go to Q109.1)

107.2 **What were the main reasons for removing the IUD?**

a) • Partial expulsion (IUD in cervix)

b) • Signs of pelvic infection

c) • Pregnancy - intrauterine

d) • Pregnancy - ectopic

e) • Painful intercourse

f) • Heavy menstruation

g) • Painful menstruation

h) • Irregular menstruation

i) • Continuous bleeding/spotting

j) • Intermittent spotting

k) • No menstruation

l) • Cramping/pelvic pain

m) • Acne

n) • Headaches

o) • Mood swings

p) • Vaginal dryness

q) • Decreased libido

r) • Partner disapproved

s) • Wanted to become pregnant

t) • Other, please specify:

SECTION VI: FOR STUDY PARTICIPANTS WHO REPORT THAT THEY ARE NOT CURRENTLY USING AN IUD (at the time of this follow-up visit)

108.1 **How come you are currently not using an IUD?**

a) • The IUD was never inserted (go to Q109.1)

b) • I had the IUD removed and it was not replaced

c) • The IUD came out by itself and it was not replaced

108.2 **What were the main reason/s for removing/not replacing the IUD? (Tick all that apply)**

a) • Partial expulsion (IUD in cervix)

b) • Pelvic infection

c) • Pregnancy - intrauterine

d) • Pregnancy - ectopic

e) • Painful intercourse

f) • Heavy menstruation

g) • Painful menstruation

h) • Irregular menstruation

i) • Continuous bleeding/spotting

j) • Intermittent spotting

k) • No menstruation

l) • Cramping/pelvic pain

m) • Acne

n) • Headaches

o) • Mood swings

p) • Vaginal dryness

q) • Decreased libido

r) • Partner disapproved

s) • Wanted to become pregnant

t) • Other, please specify:

SECTION VII: CONTRACEPTIVE USE FOLLOWING THIS VISIT - ALL STUDY PARTICIPANTS

109.1 **Did the study participant change OR initiate a new contraceptive method during this visit?**

Please note that 'initiate a method' means that a method is administrated, inserted or prescribed (pills, implant, IUD, patch, vaginal ring, emergency contraception, injectable) OR she plans to use it during next intercourse (condom, diaphragm, foam or jelly) OR sterilization is scheduled.

a) • YES

b) • NO

109.2 **What method of contraception did the study participant *leave with*?**

Indicate the method the study participant left with i.e. the same method she used when arriving for this visit OR the method she initiated during this visit. If none of these apply then tick 'left without a method'.

a) • Male condom

b) • Female condom

c) • Pill

d) • Implant

e) • Hormonal IUD

f) • Copper IUD

g) • Injectable

h) • Vaginal ring

i) • Hormonal patch

j) • Emergency contraception

k) • Diaphragm or cervical cap

l) • Foam or jelly (spermicide)

m) • Scheduled for female sterilization (tubes tied)

n) • Left without a method

When finalized go to the End Of Trial form below

<u>END OF TRIAL FORM: ALL RANDOMISED STUDY PARTICIPANTS</u>

110. **Did the participant complete all the follow up visits?**

a) • YES

b) • NO

111. **Trial outcome**

a) • Participant never had IUD inserted

b) • Participant had the IUD removed (for reasons other than partial expulsion)

c) • The IUD was expelled fully

d) • The IUD was expelled partially (IUD in cervix)

e) • An IUD was inserted and was neither expelled nor removed throughout the study period

(ALL PARTICIPANTS)

53. Bleeding since last visit to this facility

a) • Less than a normal menstrual bleeding

b) • Same as a normal menstrual bleeding

c) • Somewhat more than a normal menstrual bleeding

d) • A lot heavier than a normal menstrual bleeding

54. At clinical examination, did the women have any of the following symptoms…?

a) • Uterine tenderness

b) • Heavy bleeding

c) • Foul smelling discharge

d) • Severe abdominal pain

e) • None of the above

f) • Other please specify

55. Was the abortion complete?

a) • Yes

b) • No, re-evacuated using MVA

c) • No, re-evacuated using additional Misoprostol

d) • No, re-evacuated using curettage

e) • No, expectant management

56. Did the woman have an IUD inserted at her last visit to this facility? (check with Q45 if unsure)

a) • Yes

b) • No (go to Q59)

57. What did the clinical examination show?

a) • IUD in uterus - threads were visible/palpable

b) • IUD in cervix – threads/IUD were visible/palpable

c) • No IUD in cervix or uterus

d) • Unsure (proceed with ultrasound if possible)

58.1 Was an ultrasound conducted to determine if IUD was correctly placed?

a) • Yes

b) • No

58.2 What did the ultrasound show?

a) • IUD in uterus

b) • IUD in cervix

c) • No IUD in uterus or cervix

59.1 Was the IUD removed at this visit?

a) • Yes

b) • No (go to Q59)

59.2 What was the main reason for removing the IUD at this visit?

a) • Partial expulsion

b) • Signs of pelvic infection

c) • Pregnancy - intrauterine

d) • Pregnancy - ectopic

e) • Painful intercourse

f) • Heavy menstruation

g) • Painful menstruation

h) • Irregular menstruation

i) • Continuous bleeding/spotting

j) • Intermittent spotting

k) • No menstruation

l) • Cramping/pelvic pain

m) • Acne

n) • Headaches

o) • Mood swings

p) • Vaginal dryness

q) • Decreased libido

r) • Partner disapproved

s) • Wanted to become pregnant

t) • Other, please specify:

SECTION V: CONTRACEPTIVE UPTAKE

60. What method of contraception did the woman leave with?

Only check a method if the women received this method on the same day of the visit (for IUD and implant if the woman had this method inserted, injectable if it was administered, and other methods if they left with them in hand)

a) • Male condom

b) • Female condom

c) • Pill

d) • Implant

e) • Hormonal IUD

f) • Copper IUD

g) • Injectable

h) • Vaginal ring

i) • Hormonal patch

j) • Emergency contraception

k) • Diaphragm

l) • Foam or jelly (spermicide)

m) • Scheduled for female sterilization (tubes tied)

n) • Left without a method

61. Date scheduled for next visit: ___:___:_____ (date/month/year)

Intervention group: Next visit should be 3 months follow the treatment visit

62. Did the participant attend the 3 month visit?

a) • YES (go to the EOT form at the end of the CRF)

b) • NO

CRF Part IV: THREE MONTH FOLLOW UP

SECTION I: PARTICIPANT INFORMATION

A. STUDY SITE: _____________

B. STUDY ID:________________

63. **RANDOMISED TO (please circle study arm)**

a) • EARLY INSERTION

b) • STANDARD INSERTION

64. Today's date: ___:___:_____ (date/month/year)

SECTION II: PREGNANCIES - ALL PARTICIPANTS

65. Have you had unprotected sex since your last visit to this facility?

a) • Yes

b) • No

66. Have you experienced any pregnancies since last visit to this facility? Number:____

(If 0 pregnancies go to Q 71.4)

67. Have you experienced any miscarriages since last visit to this facility? Number:____

68. Have you had any induced abortions since last visit to this facility? Number:_____

69. Have you experienced any ectopic pregnancies since last visit to this facility?

Number:_____

70. Are you currently pregnant?

a) • Yes

b) • No

SECTION III: UNSCHEDULED VISITS

71.4 Did you have an unscheduled visit since your last visit to this facility?

a) • Yes

b) • No (go to SECTION IV Q72.1)

71.2 What was the main reason for the unscheduled visit?

a) • Abdominal pain

b) • Vaginal bleeding

c) • IUD came out by itself

d) • Painful sexual intercourse

e) • Other reason, please specify

71.3 Was the IUD removed at this unscheduled visit?

a) • Yes

b) • No it was left inside uterus (go to Q72.1)

c) • No, there was no IUD to remove (go to Q72.1)

71.5 What were the main reasons for removing the IUD at the unscheduled visit? (check all that apply)

a) • Partial expulsion

b) • Signs of pelvic infection

c) • Pregnancy - intrauterine

d) • Pregnancy - ectopic

e) • Painful intercourse

f) • Heavy menstruation

g) • Painful menstruation

h) • Irregular menstruation

i) • Continuous bleeding/spotting

j) • Intermittent spotting

k) • No menstruation

l) • Cramping/pelvic pain

m) • Acne

n) • Headaches

o) • Mood swings

p) • Vaginal dryness

q) • Decrease in libido

r) • Partner disapproved

s) • Wanted to become pregnant

t) • Other, please specify:

71.6 Was the IUD replaced with a new one?

a) • Yes

b) • No

SECTION IV: CURRENT CONTRACEPTIVE USE

72.1 Are you currently using any method of modern contraception?

These include: condom, pill, implant, IUD, vaginal ring, hormonal patch, foam or jelly, diaphragm or cervical cap, emergency contraception

a) • Yes

b) • No (**skip to Q76.1 in SECTION VI**)

72.2 What modern contraceptive method/s are you currently using? (check all that apply)

a) • Male condom

b) • Female condom

c) • Pill

d) • Implant

e) • Hormonal IUD

f) • Copper IUD

g) • Injectable

h) • Vaginal ring

i) • Hormonal patch

j) • Emergency contraception

k) • Diaphragm or cervical cap

l) • Spermicidal agents e.g. gel, foam, jelly, cream

m) • Female sterilization (tubes tied)

n) • Male sterilization (vasectomy)

72.3 What is your level of satisfaction with the method/s you are currently using?

a) • Highly satisfied

b) • Somewhat satisfied

c) • Somewhat unsatisfied

d) • Highly unsatisfied

72.4 Are you experiencing any side effects from the contraceptive method/s you are currently using? (check all that apply)

a) • No side effects (go to Q73)

b) • Heavy menstruation

c) • Painful menstruation

d) • Irregular menstruation

e) • Continuous bleeding/spotting

f) • Intermittent spotting

g) • No menstruation

h) • Cramping/pelvic pain

i) • Acne

j) • Headaches

k) • Mood swings

l) • Vaginal dryness

m) • Decrease in libido

n) • Other, please specify

72.5 How would you describe these side effects?

a) • Mild and tolerable

b) • Just tolerable

c) • Intolerable

SECTION V: FOR WOMEN WHO ARE CURRENTLY USING AN IUD (Non-IUD users go to section VI below)

73. What did the clinical examination show?

a) • IUD in uterus:- threads were visible/palpable

b) • IUD in cervix – threads/IUD were visible/palpable

c) • No IUD in cervix or uterus

d) • Unsure (proceed with ultrasound if possible)

74.1 Was an ultrasound conducted to confirm if IUD was correctly placed in uterus?

a) • Yes

b) • No (go to Q75.1)

74.2 What did the ultrasound show?

a) • IUD in uterus

b) • IUD in cervix

c) • No IUD in uterus or cervix

75.1 Was the IUD removed at this visit?

a) • Yes

b) • No (go to Q77 Section VII)

75.2 What was the main reason for removing the IUD at this visit?

a) • Partial expulsion

b) • Signs of pelvic infection

c) • Pregnancy - intrauterine

d) • Pregnancy - ectopic

e) • Painful intercourse

f) • Heavy menstruation

g) • Painful menstruation

h) • Irregular menstruation

i) • Continuous bleeding/spotting

j) • Intermittent spotting

k) • No menstruation

l) • Cramping/pelvic pain

m) • Acne

n) • Headaches

o) • Mood swings

p) • Vaginal dryness

q) • Decrease in libido

r) • Partner disapproved

s) • Wanted to become pregnant

t) • Other, please specify:

SECTION VI: FOR WOMEN WHO ARE NOT CURRENTLY USING AN IUD (at the time they arrive at the facility)

76.1 How come you are currently not using an IUD?

a) • The IUD was never inserted (go to Q78)

b) • I had the IUD removed (go to Q77.2)

c) • The IUD came out by itself (go to Q78)

76.2 IF you have had the IUD removed what were the main reasons for the removal? (check all that apply)

a) • Partial expulsion

b) • Signs of pelvic infection

c) • Pregnancy - intrauterine

d) • Pregnancy - ectopic

e) • Painful intercourse

f) • Heavy menstruation

g) • Painful menstruation

h) • Irregular menstruation

i) • Continuous bleeding/spotting

j) • Intermittent spotting

k) • No menstruation

l) • Cramping/pelvic pain

m) • Acne

n) • Headaches

o) • Mood swings

p) • Vaginal dryness

q) • Decrease in libido

r) • Partner disapproved

s) • Wanted to become pregnant

t) • Other, please specify:

SECTION VII: CONTRACEPTIVE UPTAKE - ALL PARTICIPANTS

77. What method of contraception did the woman leave with?

Only check a method if the women received this method on the same day of the visit (for IUD and implant if the woman had this method inserted, injectable if it was administered, and other methods if they left with them in hand)

a) • Male condom

b) • Female condom

c) • Pill

d) • Implant

e) • Hormonal IUD

f) • Copper IUD

g) • Injectable

h) • Vaginal ring

i) • Hormonal patch

j) • Emergency contraception

k) • Diaphragm

l) • Foam or jelly (spermicide)

m) • Scheduled for female sterilization (tubes tied)

n) • Left without a method

78. Date scheduled for next visit: ___:___:_____ (date/month/year)

Next visit should be 6 months after the first visit (treatment visit)

79. Did the participant attend the 6 month visit?

a) • YES (go to the EOT form at the end of the CRF)

b) • NO

CRF PART V: SIX MONTH FOLLOW UP

SECTION I: PARTICIPANT INFORMATION

A. STUDY SITE: _____________

B. STUDY ID:________________

80. **RANDOMISED TO (please circle study arm)**

a) • EARLY INSERTION

b) • STANDARD INSERTION

81. Today's date: ___:___:_____ (date/month/year)

SECTION II: PREGNANCIES - ALL PARTICIPANTS

82. Have you had unprotected sex since your last visit to this facility?

a) • Yes

b) • No

83.1 Have you experienced any pregnancies since last visit to this facility? Number:___ (If 0 pregnancies go to Q87.1)

84. Have you experienced any miscarriages since last visit to this facility? Number:____

85. Have you had any induced abortions since last visit to this facility? Number:_____

86. Have you experienced any ectopic pregnancies since last visit to this facility?

Number:________

87. Are you currently pregnant?

a) • Yes

b) • No

SECTION III: UNSCHEDULED VISITS

88.1 Did you have an unscheduled visit since your last visit to this facility?

a) • Yes

b) • No (go to Q88.1)

88.2 What was the main reason for the unscheduled visit?

a) • Abdominal pain

b) • Vaginal bleeding

c) • IUD came out by itself

d) • Painful sexual intercourse

e) • Other reason, please specify

88.3 Was the IUD removed at this unscheduled visit?

a) • Yes

b) • No, it was left inside uterus (go to Q89.1)

c) • No, there was no IUD to remove (go to Q89.1)

88.4 What were the main reasons for removing the IUD at the unscheduled visit? (tick all that apply)

a) • Partial expulsion

b) • Signs of pelvic infection

c) • Pregnancy - intrauterine

d) • Pregnancy - ectopic

e) • Painful intercourse

f) • Heavy menstruation

g) • Painful menstruation

h) • Irregular menstruation

i) • Continuous bleeding/spotting

j) • Intermittent spotting

k) • No menstruation

l) • Cramping/pelvic pain

m) • Acne

n) • Headaches

o) • Mood swings

p) • Vaginal dryness

q) • Decrease in libido

r) • Partner disapproved

s) • Wanted to become pregnant

t) • Other, please specify:

88.5 Was the IUD replaced with a new one?

a) • Yes

b) • No

SECTION IV: CURRENT CONTRACEPTIVE USE

89.1 Are you currently using any method of modern contraception?

These include: condom, pill, implant, IUD, vaginal ring, hormonal patch, foam or jelly, diaphragm or cervical cap, emergency contraception

a) • Yes

b) • No (skip to Q90 in SECTION V)

89.2 What modern contraceptive method/s are you currently using? (tick all that apply)

a) • Male condom

b) • Female condom

c) • Pill

d) • Implant

e) • Hormonal IUD

f) • Copper IUD

g) • Injectable

h) • Vaginal ring

i) • Hormonal patch

j) • Emergency contraception

k) • Diaphragm or cervical cap

l) • Spermicidal agents e.g. gel, foam, jelly, cream

m) • Female sterilization (tubes tied)

n) • Male sterilization (vasectomy)

89.3 What is your level of satisfaction with the method/s you are currently using?

a) • Highly satisfied

b) • Somewhat satisfied

c) • Somewhat unsatisfied

d) • Highly unsatisfied

89.4 Are you experiencing any side effects from the contraceptive method/s you are currently using? (Tick all that apply)

a) • No side effects (go to Q89)

b) • Heavy menstruation

c) • Painful menstruation

d) • Irregular menstruation

e) • Continuous bleeding/spotting

f) • Intermittent spotting

g) • No menstruation

h) • Cramping/pelvic pain

i) • Acne

j) • Headaches

k) • Mood swings

l) • Vaginal dryness

m) • Decrease in libido

n) • Other, please specify

89.5 How would you describe these side effects?

a) • Mild and tolerable

b) • Just tolerable

c) • Intolerable

SECTION V: FOR WOMEN WHO ARE CURRENTLY USING AN IUD (Non-IUD users go to section VI below)

90. What did the clinical examination show?

a) • IUD in uterus: - threads were visible/ palpable

b) • IUD in cervix – threads/IUD were visible/palpable

c) • No IUD in cervix or uterus

d) • Unsure (proceed with ultrasound if possible)

91.1 Was an ultrasound conducted to confirm if IUD was correctly placed in uterus?

a) • Yes

b) • No (go to Q91.1)

91.2 What did the ultrasound show?

a) • IUD in uterus

b) • IUD in cervix

c) • No IUD in uterus or cervix

92.1 Was the IUD removed at this visit?

a) • Yes

b) • No it was left inside uterus (go to Q93.1)

c) • No there was no IUD to remove (go to Q93.1)

92.2 What were the main reasons for removing the IUD? (Tick all that apply)

a) • Partial expulsion

b) • Signs of pelvic infection

c) • Pregnancy - intrauterine

d) • Pregnancy - ectopic

e) • Painful intercourse

f) • Heavy menstruation

g) • Painful menstruation

h) • Irregular menstruation

i) • Continuous bleeding/spotting

j) • Intermittent spotting

k) • No menstruation

l) • Cramping/pelvic pain

m) • Acne

n) • Headaches

o) • Mood swings

p) • Vaginal dryness

q) • Decreased libido

r) • Partner disapproved

s) • Wanted to become pregnant

t) • Other, please specify:

SECTION VI: FOR WOMEN WHO ARE NOT CURRENTLY USING AN IUD (at the time they arrive at the facility)

93.1 How come you are currently not using an IUD?

a) • The IUD was never inserted (go to Q94)

b) • I had the IUD removed and it was not replaced (go to Q93.2)

c) • The IUD came out by itself and it was not replaced

93.2 IF you have had the IUD removed what were the main reasons for the removal? (Tick all that apply)

a) • Partial expulsion

b) • Signs of pelvic infection

c) • Pregnancy - intrauterine

d) • Pregnancy - ectopic

e) • Painful intercourse

f) • Heavy menstruation

g) • Painful menstruation

h) • Irregular menstruation

i) • Continuous bleeding/spotting

j) • Intermittent spotting

k) • No menstruation

l) • Cramping/pelvic pain

m) • Acne

n) • Headaches

o) • Mood swings

p) • Vaginal dryness

q) • Decreased libido

r) • Partner disapproved

s) • Wanted to become pregnant

t) • Other, please specify:

SECTION IV: CONTRACEPTIVE UPTAKE - ALL PARTICIPANTS

94. What method of contraception did the woman leave with?

Only tick a method if the women received this method on the same day of the visit (for IUD and implant if the woman had this method inserted, injectable if it was administered, and other methods if they left with them in hand)

a) • Male condom

b) • Female condom

c) • Pill

d) • Implant

e) • Hormonal IUD

f) • Copper IUD

g) • Injectable

h) • Vaginal ring

i) • Hormonal patch

j) • Emergency contraception

k) • Diaphragm

l) • Foam or jelly (spermicide)

m) • Scheduled for female sterilization (tubes tied)

95. Date scheduled for next visit: ___:___:_____ (date/month/year)

Next visit should be 12 months after the first visit (treatment visit)

96. Did the participant attend the 12 month follow up visit?

a) • YES (If YES go to the EOT form at the end of the CRF)

b) • NO

CRF PART VII: TWELVE MONTH FOLLOW UP

SECTION I: PARTICIPANT INFORMATION

A. STUDY SITE: ______________

B. STUDY ID:_________________

97. **RANDOMISED TO (please circle study arm)**

a) • EARLY INSERTION

b) • STANDARD INSERTION

98. Today's date: ____:____:_______ (date/month/year)

SECTION II: PREGNANCIES - ALL PARTICIPANTS

99. Have you had unprotected sex since your last visit to this facility?

a) • Yes

b) • No

100. Have you experienced any pregnancies since last visit to this facility? Number:____

(If 0 the go to Q100.1 Section III)

101. Have you experienced any miscarriages since last visit to this facility? Number:____

102. Have you had any induced abortions since last visit to this facility? Number:____

103. Have you experienced any ectopic pregnancies since last visit to this facility?

Number:____

104.1 Have you given birth since your last visit to this facility?

a) • Yes

b) • No

104.2 Are you currently pregnant?

a) • Yes

b) • No

SECTION III: UNSCHEDULED VISITS

105.1 Did you have an unscheduled visit since your last visit to this facility?

a) • Yes

b) • No (go to Q101.1 Section IV)

105.2 What was the main reason for the unscheduled visit?

a) • Abdominal pain

b) • Vaginal bleeding

c) • IUD came out by itself

d) • Painful sexual intercourse

e) • Other reason, please specify

105.3 Was the IUD removed at this unscheduled visit?

a) • Yes

b) • No, it was left inside the uterus

c) • No, there was no IUD to remove (go to Q101.1)

105.4 What were the main reasons for removing the IUD at the unscheduled visit?

a) • Partial expulsion

b) • Pelvic infection

c) • Pregnancy - intrauterine

d) • Pregnancy - ectopic

e) • Painful intercourse

f) • Heavy menstruation

g) • Painful menstruation

h) • Irregular menstruation

i) • Continuous bleeding/spotting

j) • Intermittent spotting

k) • No menstruation

l) • Cramping/pelvic pain

m) • Acne

n) • Headaches

o) • Mood swings

p) • Vaginal dryness

q) • Decreased libido

r) • Partner disapproved

s) • Wanted to become pregnant

t) • Other, please specify:

105.6 Was the IUD replaced with a new one?

a) • Yes

b) • No

Section IV: CURRENT CONTRACEPTIVE USE

106.1 Are you currently using any method of modern contraception?

These include: condom, pill, implant, IUD, vaginal ring, hormonal patch, foam or jelly, diaphragm or cervical cap, emergency contraception

a) • Yes

b) No (go to Q106.1 section V)

106.2 What modern contraceptive method/s are you currently using? (Tick all that apply)

a) • Male condom

b) • Female condom

c) • Pill

d) • Implant

e) • Hormonal IUD

f) • Copper IUD

g) • Injectable

h) • Vaginal ring

i) • Hormonal patch

j) • Emergency contraception

k) • Diaphragm or cervical cap

l) • Spermicidal agents e.g. gel, foam, jelly, cream

m) • Female sterilization (tubes tied)

n) • Male sterilization (vasectomy)

106.3 What is your level of satisfaction with the method/s you are currently using?

e) • Highly satisfied

f) • Somewhat satisfied

g) • Somewhat unsatisfied

h) • Highly unsatisfied

106.4 Are you experiencing any side effects from the contraceptive method/s you are currently using? (Tick all that apply)

a) • No side effects (go to Q)

b) • Heavy menstruation

c) • Painful menstruation

d) • Irregular menstruation

e) • Continuous bleeding/spotting

f) • Intermittent spotting

g) • No menstruation

h) • Cramping/pelvic pain

i) • Acne

j) • Headaches

k) • Mood swings

l) • Vaginal dryness

m) • Decrease in libido

n) • Other, please specify

106.5 How would you describe these side effects?

a) • Mild and tolerable

b) • Just tolerable

c) • Intolerable

SECTION V: FOR WOMEN WHO ARE CURRENTLY USING AN IUD (Non-IUD users go to section VI Q)

107 What did the clinical examination show?

a) • IUD in uterus - threads were visible/ palpable

b) • IUD in cervix – threads/IUD were visible/palpable

c) • No IUD in cervix or uterus

d) • Unsure (proceed with ultrasound if possible)

108.1 Was an ultrasound conducted to confirm if IUD was correctly placed in uterus?

a) • Yes

b) • No (go to Q109.1)

108.2 What did the ultrasound show?

a) • IUD in uterus

b) • IUD in cervix

c) • No IUD in uterus or cervix

109.1 Was the IUD removed at this visit?

a) • Yes

b) • No

109.2 What were the main reasons for removing the IUD?

a) • Partial expulsion

b) • Signs of pelvic infection

c) • Pregnancy - intrauterine

d) • Pregnancy - ectopic

e) • Painful intercourse

f) • Heavy menstruation

g) • Painful menstruation

h) • Irregular menstruation

i) • Continuous bleeding/spotting

j) • Intermittent spotting

k) • No menstruation

l) • Cramping/pelvic pain

m) • Acne

n) • Headaches

o) • Mood swings

p) • Vaginal dryness

q) • Decreased libido

r) • Partner disapproved

s) • Wanted to become pregnant

t) • Other, please specify:

SECTION VI: FOR WOMEN WHO ARE NOT CURRENTLY USING AN IUD (at the time of this follow-up visit)

110.1 How come you are currently not using an IUD?

a) • The IUD was never inserted (go to Q111)

b) • I had the IUD removed (go to Q110.2)

c) • The IUD came out by itself (go to Q111)

110.2 What were the main reason/s for the removing the IUD? (Tick all that apply)

a) • Partial expulsion

b) • Pelvic infection

c) • Pregnancy - intrauterine

d) • Pregnancy - ectopic

e) • Painful intercourse

f) • Heavy menstruation

g) • Painful menstruation

h) • Irregular menstruation

i) • Continuous bleeding/spotting

j) • Intermittent spotting

k) • No menstruation

l) • Cramping/pelvic pain

m) • Acne

n) • Headaches

o) • Mood swings

p) • Vaginal dryness

q) • Decreased libido

r) • Partner disapproved

s) • Wanted to become pregnant

t) • Other, please specify:

SECTION VII: CONTRACEPTIVE UPTAKE - ALL PARTICIPANTS

111. What method of contraception did the woman leave with?

Only tick a method if the women received this method on the same day of the visit (for IUD and implant if the woman had this method inserted, injectable if it was administered, and other methods if they left with them in hand)

a) • Male condom

b) • Female condom

c) • Pill

d) • Implant

e) • Hormonal IUD

f) • Copper IUD

g) • Injectable

h) • Vaginal ring

i) • Hormonal patch

j) • Emergency contraception

k) • Diaphragm

l) • Foam or jelly (spermicide)

m) • Scheduled for female sterilization (tubes tied)

n) • Left without a method

When finalized go to the End Of Trial form below

END OF TRIAL FORM: ALL RANDOMISED WOMEN

112. Did the participant complete all the follow up visits?

a) • YES

b) • NO

113. Trial outcome

a) • Participant never had IUD inserted

b) • Participant had the IUD removed (for reasons other than partial expulsion)

c) • The IUD was expelled fully

d) • The IUD was expelled partially

e) • The IUD was neither expelled nor removed

APPENDIX XXII: POST-TRIAL ACCESS: TRIAL CLOSE AND CONTINUATION ACCESS MANUAL

Purpose:

The purpose of this Manual of Operations (MOP) is to streamline the entire close out procedures that will be required at the 15 sites prior to ending the trial.

Close-out refers to the steps we shall undertake at the 15 trial sites in regards to the activities (administrative, data, and study materials) and responsibilities in accordance with the trial protocol, the GCP and the relevant regulatory requirements.

Study Continuation Phase: Whereas the trial is aimed to report the expulsion and continuation rates six months after insertion of intrauterine devices after medical management of first trimester incomplete abortions, study participants who will consent to continue in the study will be followed up to one year from the recruitment into the study. The aim of the continuation phase will be to assess whether the level of satisfaction, effectiveness and continuation rates of the post abortion intrauterine contraception will remain comparable in the two arms.

Timeline:

The Table below highlights the milestones we plan to achieve in execution of the close out phase of the trial.

Event/Milestone	Deadline

CRF completion and queries	31st January 2023
Last participant recruited	1st February 2023
Final Study visits	1st February, 2024
Data cleaning	Ongoing till the database closes
Queries in the STATA in the 2 groups	Responses/Resolution by 1st March 2024
Database lock	1st April 2024
Initial Results	1st May 2024
Result presentation	June 2024
Results submission	July 2024
Submission for Publication of study findings	August 2024

END OF TRIAL:

At all the study sites, the trial CRF forms will be labeled “EOT” and key aspects will be summarized by the different study teams in regards to the pregnancy rates, continuation rates, expulsion rates, or any adverse events that will necessitate follow up. The infection rates following the different timing and type of IUD inserted post medical management of first trimester incomplete abortion. The participants requesting to change from one intrauterine contraception type to another will be evaluated as per the study protocols.

ADVERSE EVENTS:

FOLLOW UP OF UNRESOLVED EVENTS:

Any adverse event that will occur during the study will be followed up as highlighted under the adverse events section in the protocol. If such events do not resolve by the recruitment of the last participant into the study, the trial team will continue to follow up such participants till resolution or when the participants stabilize. When the events don’t stabilize or resolve at the close of the trial, the End of Trial committee will decide on whether more time needs to be given to follow the participants or to refer to such occurrences as “unresolved”. This evaluation could be rendered till the database locks. If resolution of the adverse events occurs between end of trial and database locking, the safety committee will report such events to the ethics review board using the appropriate reporting systems.

PREGNANCY:

All participants who conceive will be followed up even beyond the trial closure to determine the pregnancy outcomes. Measures such as face-to-face interface, phone calls will be conducted to assess this aspect in both groups. The outcomes will be documented and presented according to the protocol.

LOSS TO FOLLOW:

Participants that will have exited early in the study (consent withdrawal) and those that get lost to follow up will be documented at each of the 15 study sites. These will be assessed at the end of the trial. These participants will be noted in logs and as well as CRF forms. Every effort shall be put underway to reach out to such participants for a face-to-face interface for their scheduled visits. If such efforts are futile, phone interviews shall be put underway to reach out to such participants. At the close of the trial, these CRFs will be analyzed and presented accordingly.

ACCOUNTABILITY:

At all of the 15 study sites, measures will be put in place to ensure that all intrauterine devices, drugs, and materials are fully accounted for. The amount of drugs and supplies received, used and expired, will be fully accounted for at the end of the trial. Study monitors will assess these aspects. Proper inventory of drugs, intrauterine devices and supplies will be kept up to 12 months post-trial. These records shall be kept at the different study sites for future reference. All materials like intrauterine devices that might not be issued out will be donated to the different family planning units at the health facilities with consent from the trial team and the sponsors as will be streamlined in the memorandum of understanding.

FINAL STOCK REVIEW:

All drugs and supplies (expired, non-expired, used and unused) will be physically accounted for by the study monitors where feasible in the presence of the trial manager or local PI. Where this is not feasible, an onsite pharmacist will evaluate the stock using the inventory logs, destruction logs and participant registers. Once the accountability is verified, the trial manager or PI will sign the forms and expired drugs and supplies will be destroyed in accordance to the NDA protocols.

DATA CLEANING:

Prior to database locking on 1st April 2024, all efforts will be put underway to clean the data. Inconsistency logs and queries will be generated and addressed by the trial team to minimize errors. Emails and phone communication will be used to verify any missing records in this regard. The data cleaning process will be on going throughout the trial period. Measures to ensure 100% closures of queries in the database will be put in place before the locking of the database. Planned timelines for data cleaning and query reports will be ensured by the biostatistician every month prior to the closure of the trial.

DATA QUALITY CONTROL AT THE TRIAL SITES:

All CRF forms will be filled in completely at all the visits according to the manual of procedures. All missed visits will be documented in the subsequent notes and kept track of throughout the trial period. The PI will keep a track of all these occurrences and a record of all of them will be presented at the end of the trial. Loss to follow up participants will be reported in logs and the reasons behind their loss to follow up will be documented and presented at the end of the trial.

Different forms will be used to capture details of those lost to follow up. Measures to ensure that all CRFs are completed and handed in two weeks to the data entry teams will be in place prior to closure of the trial.

All Data entry will be ensured two weeks after recruitment of the last participant. Measures to ensure that completeness is achieved in all the data entered will be ensured by all the concerned data entry teams. Outstanding CRFs and queries will be aggressively pursued and rectified prior to closure of the trial.

MONITORING:

On site monitoring of all the trial activities will be ensured by the monitors. Such monitoring visits will be continually planned with onsite teams throughout the trial period. Efforts to achieve all the preset targets will be put underway by the trial teams at the different facilities. For targets which are under achieved or not clearly set will be evaluated and addressed using risk based approaches

as deemed appropriate. At the close of trial, a reflection and reevaluation of the trial activities will be conducted.

INVESTIGATOR'S TRIAL FILE:

At all study sites, an investigator's file containing up-to-date CRFs, GCP certificates, staff CVs, Ethical reporting and corresponding forms will be observed. These will be evaluated at the end of the trial and efforts will be in place to ensure completeness in the documentation and filing of the required paperwork. The monitoring plans and self-evaluation by the study team will be assessed periodically and at the end of the trial for feedback on the trial run.

SITE CLOSE OUT:

The trial team plans to close out all sites at the same time when feasible. When deemed feasible, the local monitor and the on-site teams would close the sites when the preset targets are met in accordance to the manual of procedures. A close out checklist will be used to assess the readiness of the sites to close. Once the targets are achieved for the individual sites according to the preset guidelines as highlighted in the protocol, and a confirmation report certifying the completion, the individual sites will be closed. The trial team will thereafter report to the Ethics review boards and regulatory authorities of the closure of the trial with submission of the closure report according to the local guidelines.

TRIAL INVOICES:

All payments to the trial team and participants shall be ensured prior to closure of the trial. All outstanding invoices applicable to the main study will be paid by 1st March 2024.

ARCHIVING OF TRIAL DOCUMENTS:

The trial documents will be archived as mandated for 15-20 years after the main trial. Local guidelines in regards to the archiving will also be followed appropriately. Archiving guidelines will be shared for all the 15 study sites.

DISSEMINATION OF STUDY FINDINGS:

A dissemination plan will be disclosed once the results are presented and discussed to the doctoral committee. The Thesis will be presented to a preliminary committee and thereafter discussed in the Makerere University College of Health Sciences for examination.

As feedback, measures are underway to ensure that participants at the different study sites are convened and the results disseminated to the participants highlighting the implication of such findings to the participants as well as to the local and international community. We hope that we shall have this avenue to have the participants give their experiences during the study period to the researchers as well. For participants that might require letters summarizing the study findings, with guidance from the IRBs, we shall devise ways to avail them the required information.

CONTINUATION PHASE ACCESS TO TRIAL MATERIALS AND REPORTING:

After closure of the trial, we shall ensure that the unused materials are provided by the health facilities to the family planning units. We shall ensure ongoing trainings periodically for the different health facilities where our study will be conducted to build capacity and ensure continuity of care even after closure of the trial.

At the interim analysis, once one of the intrauterine devices (Copper or Levonorgestrel IUD) is noted to be superior to the other, the participants will be updated and will be availed the opportunity to chance their contraceptive options whenever they opt to change at no cost to the participants.

With consent from the participants, the registry of participants will be given to the family planning units at the different health facilities for continuation of care even after the trial closes. Any participants who might opt for another contraceptive method other than one offered after medical management of 1st trimester incomplete abortion will be noted and database updated at the health facility but not in the trial.

Participants, who consent to continue in the study, will be followed up six months after assessment of the primary and secondary outcomes, to assess expulsion, continuation rates and pregnancy outcomes at one year after insertion of intrauterine contraceptive devices.

We shall link the different health facilities where the trial will be running to the sponsor of the Levonorgestrel intrauterine devices to avail this option of contraception which is not readily available to all women in Central Uganda.

We plan to have a mobile access for the study participants to keep consulting in regards to their choice of contraception even after the trial.

Accountability of the continuation phase will be managed by the different health facility administration.

APPENDIX XXIII: COVID 19 RISK MANAGEMENT PLAN FOR THE EFFECTIVENESS, ACCEPTABILITY AND UPTAKE OF EARLY VERSUS STANDARD INTRAUTERINE CONTRACEPTION FOLLOWING PROVISION OF FIRST TRIMESTER MEDICAL POST ABORTION CARE IN CENTRAL UGANDA (Mak-SOMREC-2021-133)

INTRODUCTION

Coronavirus 2 (SARS-CoV-2) is a new virus which has not been identified in humans previously and therefore, no population-level immunity exists. It is highly transmissible through droplet infections that attack the respiratory, intestinal and brain tissues. Infection from Corona virus 2 results in coronavirus disease (COVID-19) (MOH 2020).

SYMPTOMS OF COVID-19

COVID-19 disease symptoms include; fever, cough myalgia or fatigue, shortness of breath, sore throat and headache. Other COVID-19 symptoms may include: flu-like symptoms, diarrhoea and nausea, muscle ache, pneumonia and Acute Respiratory Distress Syndrome (ARDS), renal failure, pericarditis and Disseminated Intravascular Coagulation (DIC) (MOH 2020). However it is reported that some COVID-19 patients may be asymptomatic and therefore strict Infection Prevention and Control (IPC) measures have to be put in place to protect people at all times (MOH 2020).

OBJECTIVES OF THE RISK MANAGEMENT PLAN

The objectives of this risk management plan entail:

1. To provide guidance on safe conduct of all trial activities during the COVID-19 pandemic

2. To describe in detail the measures that will be taken to protect the trial staff and study participants from COVID-19.

PROTECTIVE/SAFETY MEASURES TO BE TAKEN AT THE STUDY SITES

Protective measures to be taken on arrival at the Study sites:

Hand Hygiene:

Sanitisers, water and soap will be put at the entrance of the study site clinic where every study staff member and study participant will be required to wash or sanitise their hands before entering the different health facilities where the study will be conducted.

Body temperature measurement:

After sanitisation, the trial staff and participants' body temperatures will be taken on a daily basis by one of the study staff member on duty using the infrared temperature monitor provided by the study. Any person whose temperature will be above the normal body temperature range (> 37.5 degrees Celsius) will be isolated immediately and referred for further investigations at the designated treatment areas by the Ministry of Health or District COVID-19 task force.

Use of Personal protective equipment:

All trial staff will be required to put on medical face masks at all times with the nose, mouth and chin covered to avoid direct contact with study participants' blood, body fluids, secretions (including respiratory secretions) and non-intact skin. A box of disposable medical masks will always be available at the different health facilities where the study will be undertaken to be dispensed to all study staff as soon as they reach the study site. Medical gowns and gloves will also be worn where appropriate to minimize any transmission of COVID-19 among the study team and also to the participants.

Study participants will be required to put on face masks and will be provided with medical masks by the trial team in case they do not come with any mask or if they are wearing face masks that are not recommended by Ministry of Health.

Ensuring social distancing at the study sites:

At the study sites, in waiting areas, a bench will be shared by only two study participants sitting two metres apart with each wearing a medical mask in the proper way with the nose and mouth and chin completely covered.

Chairs will be placed at least two metres apart to ensure social distancing in the clinic when consenting and interviewing study participants. We shall ensure that appointments will be given to ensure that there's minimal congestion at any one time at the different study sites. We plan on not having more than ten participants at any one visit at any of the designated health facilities. This will ensure minimal risk of transmission of COVID-19.

Vaccination of study staff and Participants:

The study will only hire staffs that are fully vaccinated as recommended by the Ministry of Health of Uganda. Study participants will be advised to undertake the COVID-19 vaccination at the designed areas by the Ministry of Health of Uganda.

Protective measures to be taken during study participant screening and enrolment in the examination rooms:

Hand hygiene:

In the examination room, before and after screening and enrolling any study participant, the study staff and the study participants will be required to sanitise their hands again.

All study staff and participants will have constant reminders in place to avoid touching their eyes, nose or mouth areas.

Protective wear:

The study staff will be required to put on medical masks, disposable aprons and gloves to avoid any possibility of contamination when carrying out the screening and enrolment procedures on the study participants. A new pair of gloves will be used for each participant. The study staff will be required to hand wash/sanitise before and after putting on gloves. The study staff will use face shields whenever undertaking insertion of the intrauterine devices after medical management of

first trimester incomplete abortions or when dealing with any participants suspected to have any symptoms suggestive of COVID-19.

All trial participants will be required to enter the examination room with properly worn face mask.

Environmental control measures:

Social distancing when consenting and interviewing study participant:

The interviewer's and study participants' chairs will be placed at least two metres apart to ensure social distancing when consenting and interviewing study participants. During this time both the study staff and participant must be putting on their face masks properly.

Keeping the working environment clean:

The chairs, tables and pens that will be used during the consenting and interviewing process, will be sanitised before and after handling each study participant.

Proper ventilation of the rooms will be ensured by keeping the windows and ventilators wide open.

All rooms where study activities take place will be kept clean through mopping floors, dusting and sanitizing tables, examination beds, chairs, rails, sinks, door locks, filing cabins using water, JIK and alcohol solutions each morning plus before and after working on each study participant.

Cleaning and disinfection of equipment used in the trial will be done regularly and according to the trial standard operating procedures. The cleaning and disinfecting equipment/solutions will always be available on site.

Dealing with participants or study staff suspected to have COVID-19:

Study participants and staff with symptoms suggestive of COVID-19 such as high grade fever, nasal congestion or difficulty in breathing, will be advised to be in isolation and linked into care at the designated treatment COVID-19 treatment areas.

Safe waste management:

Used masks, gloves and aprons will be removed safely and disposed appropriately in infectious waste bins that are on site to avoid transmissions.

Communication:

Visual aids on will be in place at all the study areas to remind the study staff and the participants on the symptoms of COVID-19, appropriate measures to prevent or minimize transmission of COVID-19 and where to seek help from in case they are suspected of having the COVID-19 during the study period. There will be displayed on doors and notice boards at the different study sites.

Refresher training will be offered to all study staff on how to identify persons with COVID-19 during the study period. Persons presenting with flu like symptoms, difficulty in breathing, fever, or with prior travel history in COVID-19 stricken areas will be identified, isolated immediately and referred to designated COVID-19 treatment areas.

Conclusion:

The above risk management plan is going to be effected after having a practical training session with all study staff and before commencement of any trial activities.

References

Ministry of Health 2020, National guidelines for management of COVID 19
