# Supplementary material for: Healthcare providers’ perceptions on post abortion intrauterine contraception: A qualitative study in central Uganda
Source: PLoS One. 2024 Dec 5;19(12):e0301748. doi: 10.1371/journal.pone.0301748 (PMC11620438; doi:10.1371/journal.pone.0301748)
Supplement: Attachment — Submitted filename: feedback PONE.docx [file pone.0301748.s003.docx]

**Healthcare Providers’ Perceptions on Post Abortion Intrauterine Contraception: A qualitative study in central Uganda**

**Summary of the Research**

Despite access to post abortion intrauterine contraception, the uptake of Intrauterine devices (IUDs) in Uganda is below four percent. Whether the perceptions of healthcare providers towards IUDs have a role in the provision of post abortion IUDs remains unclear. The aim of study to explore the perceptions of healthcare providers towards post abortion IUDs after medical management of first trimester incomplete abortions. Methods: Between 1st August 2022 and 30th September 2022, forty-five in-depth interviews were conducted among healthcare providers of different cadres in sixteen public health facilities in central Uganda. The interviews were primarily to help us understand the perceptions of healthcare providers towards IUDs. All interviews were audio-recorded and transcribed verbatim. Themes were identified using inductive content analysis. From the content analysis of the interviews, three themes emerged. Theme one covered health facility related barriers such as healthcare providers’ and health system challenges towards provision of post abortion IUDs. The second theme was around the challenges in reaching and counselling women to use the post abortion IUDs. The third theme covered the motivating factors and participants’ views on how to scale up IUD uptake and provision within postabortion care in Uganda. Even with the availability of the necessary materials and equipment required for post abortion IUDs, and improved logistics such as timing of insertion to overcome some of the barriers faced by patients, service delivery hinges on the healthcare providers’ perceptions. Measures to address healthcare providers’ knowledge gaps and IUD skill sets should be enhanced. Healthcare providers’ misconceptions need to be addressed with provision of evidence-based information to enable correct information to patients. There is a need to address health system barriers like patient overload, stock-outs of equipment and materials for IUD insertion, and privacy. Provision of on-job refresher trainings, community sensitization on IUDs, financial incentives, and support supervision are key motivators that can be utilized in supporting healthcare providers towards post abortion IUD provision.

**Areas for improvement**

**Title:**

Good title.

**Abstract:**

Clear and good abstract.

Key words: limits the words to the most important, 5-6 words is enough.

**Order of the authors:**

Mention all authors of this review please.

Make sure that all necessary information for all authors are available in this document.

**Introduction Section:**

In one sentence explain if maternal death is associated with not using birth spacing methods.

The authors should revise introduction section for grammar issues and language to improve readability.

Join paragraphs that discussing the same concepts.

The authors should indicate what they wanted the readers to understand.

**Overall**

Good introduction but the authors need to arrange introduction section.

**Material and Methods:**

**Study design,**

Clear.

**Setting,**

Clear.

**Participants recruiting,**

Clear.

**Inclusion and exclusion criteria,**

Clear.

**Quality Assessment,**

The authors should explain the how the maintained validity of data.

**Data collection and analysis,**

Clear, however topic guild should be explained in detail with the questions.

**Ethical consideration,**

Clear.

**Results:**

The authors should revise the language and grammar to improve readability.

**Discussion:**

The authors should make clear for the readers about the themes that they were discussing.

The authors should discuss each themes from different angle and in detail.

The authors should revise the language and grammar to improve readability.

**References:**

The authors should revise all references according to the guidelines provided.

**Overall:** Good manuscript, all the best.
