# Supplementary material for: Healthcare providers’ perceptions on post abortion intrauterine contraception: A qualitative study in central Uganda
Source: PLoS One. 2024 Dec 5;19(12):e0301748. doi: 10.1371/journal.pone.0301748 (PMC11620438; doi:10.1371/journal.pone.0301748)
Supplement: Attachment — Submitted filename: Response to reviewer comments_finale.docx [file pone.0301748.s004.docx]

P.O. Box 7072, Kampala, Uganda

Phone: +256-414-534361

e-mail: [obsgyn@chs.mak.ac.ug](mailto:obsgyn@chs.mak.ac.ug)

## COLLEGE OF HEALTH SCIENCES

## SCHOOL OF MEDICINE

## DEPARTMENT OF OBSTETRICS AND GYNAECOLOGY

15^th^ July 2024

RESPONSE TO REVIEWS COMMENTS:

***Object:* PONE-D-24-05509: “HEALTHCARE PROVIDERS’ PERCEPTIONS ON POST ABORTION INTRAUTERINE CONTRACEPTION: A QUALITATIVE STUDY IN CENTRAL UGANDA”**

With great pleasure, I’m thankful for your comments towards our manuscript. In response to the reviewers’ comments sent to us on 11^th^ June 2024, we have revised the manuscript accordingly.

| **Comment** | **Response to Comment** | **Page No. and Line** |
| --- | --- | --- |
| **Journal Requirements:**  When submitting your revision, we need you to address these additional requirements.  1. Please ensure that your manuscript meets PLOS ONE's style requirements, including those for file naming. The PLOSONE style templates can be found at  <https://journals.plos.org/plosone/s/file?id=wjVg/PLOSOne_formatting_sample_main_body.pdf> and  <https://journals.plos.org/plosone/s/file?id=ba62/PLOSOne_formatting_sample_title_authors_affiliations.pdf>  2. Thank you for stating the following financial disclosure:  “ I received part of the funding from Kalorinska Institutet in partnership with Makerere University for the datacollection and analysis but not in towards the publication and dissemination. I would be grateful if I can be availed a full publication waiver towards my publication. I have in the past reviewed peer articles for free for this highly reputable journal and would be grateful if my request for a publication waiver is given your kind consideration."  Please state what role the funders took in the study.  If the funders had no role, please state: ""The funders had no role in study design, data collection and analysis, decision to publish, or preparation of the manuscript.""  If this statement is not correct you must amend it as needed.  Please include this amended Role of Funder statement in your cover letter; we will change the online submission form on your behalf.  3. When completing the data availability statement of the submission form, you indicated that you will make your data available on acceptance. We strongly recommend all authors decide on a data sharing plan before acceptance, as the process can be lengthy and hold up publication timelines. Please note that, though access restrictions are acceptable now, your entire data will need to be made freely accessible if your manuscript is accepted for publication. This policy applies to all data except where public deposition would breach compliance with the protocol approved by your research ethics board. If you are unable to adhere to our open data policy, please kindly revise your statement to explain your reasoning and we will seek the editor's input on an exemption. Please be assured that, once you have provided your new statement, the assessment of your exemption will not hold up the peer review process.  4. Please include captions for your Supporting Information files at the end of your manuscript, and update any in-text citations to match accordingly. Please see our Supporting Information guidelines for more information: <http://journals.plos.org/plosone/s/supporting-information>.  Reviewers' comments:  Reviewer's Responses to Questions  **Comments to the Author**  1. Is the manuscript technically sound, and do the data support the conclusions?  The manuscript must describe a technically sound piece of scientific research with data that supports the conclusions. Experiments must have been conducted rigorously, with appropriate controls, replication, and sample sizes. The conclusions must be drawn appropriately based on the data presented.  Reviewer #1: Yes  Reviewer #2: Partly  Reviewer #3: Yes  2. Has the statistical analysis been performed appropriately and rigorously?  Reviewer #1: Yes  Reviewer #2: No  Reviewer #3: No  3. Have the authors made all data underlying the findings in their manuscript fully available?  The [PLOS Data policy](http://www.plosone.org/static/policies.action#sharing) requires authors to make all data underlying the findings described in their manuscript fully available without restriction, with rare exception (please refer to the Data Availability Statement in the manuscript PDF file). The data should be provided as part of the manuscript or its supporting information, or deposited to a public repository. For example, in addition to summary statistics, the data points behind means, medians and variance measures should be available. If there are restrictions on publicly sharing data—e.g. participant privacy or use of data from a third party—those must be specified.  Reviewer #1: Yes  Reviewer #2: No  Reviewer #3: Yes  4. Is the manuscript presented in an intelligible fashion and written in standard English?  PLOS ONE does not copyedit accepted manuscripts, so the language in submitted articles must be clear, correct, and unambiguous. Any typographical or grammatical errors should be corrected at revision, so please note any specific errors here.  Reviewer #1: Yes  Reviewer #2: Yes  Reviewer #3: Yes  5. Review Comments to the Author  Please use the space provided to explain your answers to the questions above. You may also include additional comments for the author, including concerns about dual publication, research ethics, or publication ethics. (Please upload your reviewas an attachment if it exceeds 20,000 characters)  Reviewer #1: Summary of the Research Despite access to post abortion intrauterine contraception, the uptake of Intrauterine devices (IUDs) in Uganda is below four percent. Whether the perceptions of healthcare providers towards IUDs have a role in the provision of post abortion IUDs remains unclear. The aim of study to explore the perceptions of healthcare providers towards post abortion IUDs after medical management of first trimester incomplete abortions. Methods: Between 1st August 2022 and 30th September 2022, forty-five in-depth interviews were conducted among healthcare providers of different cadres in sixteen public health facilities in central Uganda. The interviews were primarily to help us understand the perceptions of healthcare providers towards IUDs. All interviews were audio-recorded and transcribed verbatim. Themes were identified using inductive content analysis. From the content analysis of the interviews, three themes emerged. Theme one covered health facility related barriers such as healthcare providers’ and health system challenges towards provision of post abortion IUDs. The second theme was around the challenges in reaching and counselling women to use the post abortion IUDs. The third theme covered the motivating factors and participants’ views on how to scale up IUD uptake and provision within postabortion care in Uganda. Even with the availability of the necessary materials and equipment required for post abortion IUDs, and improved logistics such as timing of insertion to overcome some of the barriers faced by patients, service delivery hinges on the healthcare providers’ perceptions. Measures to address healthcare providers’ knowledge gaps and IUD skill sets should be enhanced. Healthcare providers’ misconceptions need to be addressed with provision of evidence-based information to enable correct information to patients. There is a need to address health system barriers like patient overload, stock-outs of equipment and materials for IUD insertion, and privacy. Provision of on-job refresher trainings, community sensitization on IUDs, financial incentives, and support supervision are key motivators that can be utilized in supporting healthcare providers towards post abortion IUD provision. Areas for improvement Title: Good title. Abstract: Clear and good abstract. Key words: limits the words to the most important, 5-6 words is enough.  Order of the authors: Mention all authors of ffthis review please. Make sure that all necessary information for all authors are available in this document.  Introduction Section: In one sentence explain if maternal death is associated with not using birth spacing methods.  The authors should revise introduction section for grammar issues and language to improve readability. Join paragraphs that discussing the same concepts. The authors should indicate what they wanted the readers to understand.  Overall Good introduction but the authors need to arrange introduction section.  Material and Methods: Study design, Clear. Setting, Clear. Participants recruiting, Clear. Inclusion and exclusion criteria, Clear.  Quality Assessment, The authors should explain the how the maintained validity of data.  Data collection and analysis, Clear, however topic guild should be explained in detail with the questions.  Ethical consideration, Clear.  Results: The authors should revise the language and grammar to improve readability.  Discussion: The authors should make clear for the readers about the themes that they were discussing.  The authors should discuss each themes from different angle and in detail.  The authors should revise the language and grammar to improve readability.  References: The authors should revise all references according to the guidelines provided.  Overall: Good manuscript, all the best.  Reviewer #2: This study describes the perceptions of healthcare providers in Central Uganda on post-abortion intrauterine contraception. However, the inclusion of only 45 interviewees does not adequately represent the broader situation in Central Uganda.  The study employs inductive content analysis, but this method also has some shortcomings, particularly in terms of replicability, bias impact, and theoretical depth. The subjectivity of the researchers may affect which data are coded and how they are coded, thereby influencing the objectivity and reliability of the research results. Researchers may tend to select data that supports their anticipated hypotheses while ignoring or undervaluing data that does not fit their expectations. This could lead to the research results being biased or incomplete.  Reviewer #3: The manuscript is entitled: "Healthcare Providers’ Perceptions on Post-Abortion Intrauterine Contraception: A Qualitative Study in Central Uganda'".  The authors explored the healthcare providers' perceived barriers and facilitators to post-abortion IUDs after medical management of first-trimester incomplete abortions in Central Uganda.  General Comment: The research topic is relevant to the field and the authors thoroughly explored the research topic and demonstrated a deep understanding of the subject matter. However, during a review of the manuscript, the following areas were identified as a major concern  1. The authors clearly described the gap in low uptake of IUDs in Uganda, however, they failed to define the evidence gap. Specifically, evidence gap on the health providers' perceptions of post-abortion IUD. What others presented on the topic needs to be well presented. So, What were the gaps in the literature?  2. The title of the manuscript describes healthcare providers' Perceptions, however, what is presented in the result, discussion and even in the conclusion part is entirely about the barriers and facilitators to post-abortion IUD uptake?? It lacks coherence. The ideas should flow logically from one section to the next, with clear transitions. Each section should build upon the previous one, guiding the reader through the manuscript.  3. The reviewer can not say the methodology is sound and replicable with confidence 3.1. Despite having a clear study design, the authors exhaustively discussed the philosophical perspective that aligns with this study. "A qualitative interview study" is not specific. Which kind of qualitative study design was employed to answer the research question???  3.2. The authors assigned two independent researchers whose task was to ensure rigor in the study according to the Lincoln–Guba criteria. That's excellent. However, how was trustworthiness (rigor) ensured in this study? Specifically, what techniques were used to establish credibility, confirmability, dependability, and transferability?  Additionally, the following minor revisions/comments were identified: 1. Abstract 1.1. Methods: what was analyzed and the type of analysis conducted?  1.2. Results: Content analysis appeared in the result part. wouldn't it be repeating the methods in the result section?  2. Results 2.1. the title of Table 1: why Baseline characteristics?  2.2. Table 2: is not self-explanatory and is very long  3. Discussion: Unnecessary details were observed in this section, therefore, the authors need to be more focused while discussing only the main findings. Moreover, very long sentences need to modified again.  6. PLOS authors have the option to publish the peer review history of their article ([what does this mean?](https://journals.plos.org/plosone/s/editorial-and-peer-review-process#loc-peer-review-history)). If published, this will include your full peer review and any attached files.  If you choose “no”, your identity will remain anonymous but your review may still be made public.  **Do you want your identity to be public for this peer review?** For information about this choice, including consent withdrawal, please see our [Privacy Policy](https://www.plos.org/privacy-policy).  Reviewer #1: **Yes:** Zalikha Khamis Al-Marzouqi  Reviewer #2: No  Reviewer #3: **Yes:** Andamlak Gizaw Alamdo [NOTE: If reviewer comments were submitted as an attachment file, they will be attached to this email and accessible via the submission site. Please log into your account, locate the manuscript record, and check for the action link "View Attachments". If this link does not appear, there are no attachment files.]  While revising your submission, please upload your figure files to the Preflight Analysis and Conversion Engine (PACE) digital diagnostic tool, <https://pacev2.apexcovantage.com/>. PACE helps ensure that figures meet PLOS requirements. To use PACE, you must first register as a user. Registration is free. Then, login and navigate to the UPLOAD tab, where you will find detailed instructions on how to use the tool. If you encounter any issues or have any questions when using PACE, please email PLOS at [figures@plos.org](mailto:figures@plos.org). Please note that Supporting Information files do not need this step. | The manuscript has been revised in accordance to the PLOSONE style templates as recommended.  "The funders had no role in study design, data collection and analysis, decision to publish, or preparation of the manuscript."  All the necessary data and materials underlying the findings described have been provided as part of this manuscript. The authors agree to the free data sharing plan of the materials. In case any more data or materials are needed, they are readily accessible in the Department of Obstetrics and Gynecology library, Makerere University College of Health Sciences, Kampala, Uganda.  The manuscript has been revised in accordance to the PLOSONE style templates. We have added a caption for the supporting information as recommended.  Thanks to the reviewers for considering the manuscript to be technically sound.  We appreciate the reviewers for the comments in this regard. We have availed the revised manuscript, the approved protocol, ethical approval forms from the School of Medicine, Makerere University Ethic Review, and the Uganda National Committee of Science and Technology, informed consent and interview guide for the in-depth interviews. We have also attached the descriptive characteristics of the study sites without exposing the identity of the participants. The authors agree to the data sharing plan.  Thanks to the reviewers for considering the manuscript worthy in this regard.  The key words used in the revised manuscript are now five. They are as follows; *First-trimester abortion, Intrauterine-contraception, Perceptions, Healthcare providers, and central Uganda*  Thanks for comment. All the other names and contributions are mentioned in the revised manuscript. The write up appears as follows; *Conceptualization: HK, EL, AC, KG*  *Formal analysis: HK, JB, KO, EL*  *Data collection: HK*  *Funding acquisition: KGD, JB, OK, AC*  *Methodology: HK, EM, KS, AC, NT, KO*  *Project administration: DAG, HK, DN*  *Resources: KG, HK, NT, EL*  *Supervision: KO, AC, JB, NT, KG*  *Writing original draft: HK,*  *Writing review & editing: AC, KO, RM, EL, NT, KG, JB*  HK- Herbert Kayiga, EL- *Emelie Looft-Trägårdh, AC- Amanda Cleeve, KO- Kakaire Othman, NT- Nazarius Mbona Tumwesigye, JB-Josaphat Byamugisha, KG-Kristina Gemzell-Danielsson*  Maternal deaths follow unsafe abortions that result from the induced abortions. The induced abortions follow unintended pregnancies that result from the high unmet need for contraception in low- and middle-income countries.  Thanks for the recommendation. The introduction section has been revised accordingly. The grammar has been improved as recommended by the reviewer. The first paragraph now appears as *“Unintended pregnancies continue to be a global public health issue especially in low-and middle-income countries (LMICs) [1, 2]. As a result of the high unmet need for contraception in LMICs between 20 to 58%[3], an estimated 45% of unintended pregnancies end up as induced abortions[4]. About 75.6% in Africa and 76.1% in East Africa of these induced abortions are unsafe, contributing to 5-13% of the preventable maternal mortality [5]. In Sub-Saharan Africa, the high unmet need for contraception is driven by the healthcare providers’ knowledge gaps in contraception, inaccessibility to modern contraceptive methods especially in rural areas, minimal resource allocation for family planning services and limited skills among healthcare providers [6].*  The write up has been revised to cover the global and local Ugandan perspective of the unintended pregnancies. We have acknowledged the drivers of the unintended pregnancies in Uganda. An account of the existing low IUD uptake in Uganda is mentioned. We conclude by stating the objective of exploring the perception of healthcare providers towards the provision of intrauterine contraception after medical management of first trimester incomplete abortion in central Uganda.  We appreciate the reviewer for the kind comments.  Thanks for the comment. We ensured validity in the study by purposively sampling the study participants with maximal variation. We explicitly described the context of the study setting and ensured that we achieved data saturation prior to stopping the data collection. More than one individual assessed the data to ensure that the results were similar. We sampled healthcare providers from urban, peri-urban and rural setting to have representative sample of healthcare providers in central Uganda. We ensured triangulation as well in the study.  The manuscript has however been revised accordingly. The write up now appears as “Rigor:  *To ensure rigor in the data collection, we used Guba and Lincoln criteria [25], that included, data credibility, confirmability, transferability and dependability. Triangulation was by interviewing different cadres of healthcare providers from different health facilities and later on comparing their perspectives.Triangulation was ensured by group three (HK, RM) that was devoted to continued reading through of the transcripts to ensure ongoing comparison of the key information generated from one health facility to another. We ensured prolonged data collection. Prolonged engagement with the data was observed thereafter to make appropriate interpretation. A journal where constant reflections of our experiences, potential biases and assumptions was kept. In so doing we kept the findings in perspective with the study assumptions from the outset of the study. We also observed the individuals’ posture and body language and made notes that we compared with the other data from the interviews. Dependability was observed by the stringent coding procedure and inter-coder corroboration. Key concepts and themes arising from the data were organized into codes through an iterative process of reading the interview transcripts for recurring patterns. From the insights, initial codes were developed that were later on transformed, and labelled. A codebook from the codes was generated thereafter. Data confirmability was observed by ensuring that participants’ statements were captured with barely any modifications made. Data transferability was ensured by the research team so that a rich, condensed description of the study process was documented to enable replicability in a similar context elsewhere[28].*  *.*  Thanks for the comment. We have taken note of this and the manuscript has been revised accordingly.  The grammar in the results’ section has been revised as recommended by the reviewer.  The three themes as obtained from the results have been discussed extensively as recommended by the reviewer.  Each of the themes have been given different faces as recommended by the reviewer.  The grammar in the discussion section has been revised as recommended by the reviewer.  The refences have been revised as recommended by the reviewer and journal.  Thanks so much for the kind remarks.  Thanks so much for taking off time to review our manuscript. Since this is a qualitative study, we were not aiming for representativeness.  It’s true that 45 interviewees are not representative of the central Uganda. We however sampled 16 health public facilities that are the biggest providers of family planning services yet with established research infrastructure in central Uganda. The selected sites also had higher abortion rates as compared to the national rate of 62 vs. 39 per 1,000 live deliveries. We purposively sampled with maximal variation 2-3 participants from each of the health facilities. The health facilities were located in urban, peri-urban and rural setting. We collected data until data saturation was achieved. Taking into account that qualitative studies are not mainly about the number of participants but rather the richness of the data collected, we are convinced that the approach we used was appropriate to answer the study objective.  We appreciate the concerns raised about the use of inductive content analysis especially with the researchers’ subjectivity and potential bias. We however set out to understand the perceptions of the healthcare providers in regards to provision of post abortion intrauterine contraception with an open mind. We anticipated that with this approach, we could gather multiple perspectives or viewpoints that could explain the uptake of IUDs in central Uganda. We anticipated building stronger theories to explain the low IUD uptake of less than four percent in Uganda. The aspects which cover how we minimized bias are covered under the write up on trustworthiness under rigor in the revised manuscript.  The manuscript has been revised as follows; *‘To strengthen trustworthiness, we built rapport with the participants and also assured them of confidentiality. We also had variation in the cadres interviewed to assess whether our findings were in agreement or not. We ensured that the data collection team was different from those actively involved in the implementation of the study. We used interview probes in the interviews and ensured that the settings of the interview rooms were appropriate to minimize these potential biases from the participants”.*  Thanks so much for the kind remarks.  We appreciate the comment on the evidence gap. From literature, it is evident that IUDs are readily available in 90% of the public facilities in Uganda. Despite their availability, less than four percent of Ugandan women utilize IUDs according to Twesigye. It’s to this end that we sought to explore the perception of healthcare providers towards the provision of post abortion intrauterine contraception to better understand the barriers and facilitators of this uptake. We anticipated if we could identify these factors, it could guide in formulation of evidence-based practices to scale up the contraceptive prevalence of IUDs in Uganda and beyond. The manuscript has been revised to address these gaps.  The write up is as follows; *“With a high unmet need for contraception of 30.4%, 56% of the pregnancies in Uganda are unintended. About ten percent of the maternal mortality in Uganda are due to complications from unsafe abortions, that follow the induced abortions in the face of restrictive abortion laws [10, 11]. Uganda’s contraceptive prevalence rate of 30.4% is lower than that of her neighbors in the East African community; Kenya (46%), Rwanda (52%) and Tanzania (34%) [12]. Despite the availability of intrauterine devices in 90% of the health facilities, the national contraceptive prevalence rate of IUDs in Uganda is below four percent[13, 14].*  Thanks for the observation. We have revised the result section to make it more coherent and added to the objective of the study. Specifically, we wanted to improve understanding of their perspectives on the barriers and possible solutions in relation to provision of good quality postabortion contraceptive counselling and provision in Uganda, focusing on the IUD.  We appreciate the concern raised by the reviewer. The purpose of elaborating more about the approach we used was literally to describe with as much detail as possible the context of where the study was conducted and the aspects we considered as pertinent as we carried the study out. We believe that with this contextual background, other researchers could replicate the study. We used the case study design and conventional content analysis to answer the research question. We have revised the manuscript to address the concern. It now appears as *“We used the case study design and inductive conventional content analysis approach to answer the study question[33].*  We appreciate the comment raised by the reviewer. The write up has been revised to add the aspect of rigor. The revision in the manuscript is as follows; “Rigor:  *To ensure rigor in the data collection, we used Guba and Lincoln criteria [25], that included, data credibility, confirmability, transferability and dependability. Triangulation was by interviewing different cadres of healthcare providers from different health facilities and later on comparing their perspectives.Triangulation was ensured by group three (HK, RM) that was devoted to continued reading through of the transcripts to ensure ongoing comparison of the key information generated from one health facility to another. We ensured prolonged data collection. Prolonged engagement with the data was observed thereafter to make appropriate interpretation. A journal where constant reflections of our experiences, potential biases and assumptions was kept. In so doing we kept the findings in perspective with the study assumptions from the outset of the study. We also observed the individuals’ posture and body language and made notes that we compared with the other data from the interviews. Dependability was observed by the stringent coding procedure and inter-coder corroboration. Key concepts and themes arising from the data were organized into codes through an iterative process of reading the interview transcripts for recurring patterns. From the insights, initial codes were developed that were later on transformed, and labelled. A codebook from the codes was generated thereafter. Data confirmability was observed by ensuring that participants’ statements were captured with barely any modifications made. Data transferability was ensured by the research team so that a rich, condensed description of the study process was documented to enable replicability in a similar context elsewhere[28].*  We analyzed the perception of healthcare providers towards provision of post abortion intrauterine contraception in central Uganda. We used the case design and the conventional content analysis approach in the exploration of the healthcare providers’ perceptions. The write up has been revised accordingly. It now appears as *“We used the case study design to explore the healthcare providers’ perceptions. All interviews were audio-recorded and transcribed verbatim. Themes were identified using conventional inductive content analysis”.*  Content analysis has been deleted from the results section as recommended by the reviewer. The write in the revised manuscript now appears as *“From the analysis, three themes emerged. Theme one covered health facility related barriers such as healthcare providers’ and health system challenges towards provision of post abortion IUDs”.*  We have changed the title from Baseline characteristics to Socio-demographic characteristics.  The title of Table 2 has been edited to *“****Overview of themes and categories of the Healthcare providers’ perceptions on the uptake of Post Abortion intrauterine contraception in central Uganda:”.*** We hope this will make it more informative. More revision has been added to it as well.  We appreciate the comment. We have edited out the unnecessary details and eliminated what appears as unnecessary. The sentences have been made shorter as recommended by the reviewer.  I take this privilege to appreciate the reviewers for taking off time to review our manuscript. Your comments are highly appreciated. | Page 29, line 642-648  Page 30, line 655-660  Page 37, line 824-832  Page 37, line 824-832  Page 3, line 54-55  Page 30, line 661-674  Page 4, line 57-66  Page 4-5, line 57-104  Page 4, line 65-77  Page 8-9, line 168-187  Page 31-36, line 675-823  Page 6, line 122-135  Page 9, line 187-193  Page 4, line 70-77  Page 6, line 111-112  Page 8-9, line 168-187  Page 2, line 30-32  Page 2, line 33-36  Page 11-12  Page 12, line 247 |

Yours truly,

**Dr Herbert Kayiga**

Lecturer/Corresponding Author,

Department of Obstetrics and Gynaecology

Makerere University College of Health Sciences

+256777855063, [hkayiga@gmail.com](mailto:hkayiga@gmail.com)
